# Supplementary material for: Beliefs, perceptions, and behaviors impacting healthcare utilization of Syrian refugee children
Source: PLoS One. 2020 Aug 7;15(8):e0237081. doi: 10.1371/journal.pone.0237081 (PMC7413502; doi:10.1371/journal.pone.0237081)
Supplement: S1 File — (DOCX) [file pone.0237081.s001.docx]

| Semi- Structured Interview Guide |
| --- |
| **Medical Problems**   1. What health concerns and medical problems do you and your family have?   ما هي الاهتمامات الصحية والمشكلات الطبية لديك ولدى عائلتك ؟   1. What medical problems are common among Syrian refugees? Among children?   ما هي المشكلات الطبية الشائعة بين اللاجئين السوريين ؟ وبين الأطفال خاصة منهم ؟   1. How do you feel your medical problems impact your daily life?   كيف تؤثر مشكلاتك الطبية على حياتك اليومية ؟   1. Do you feel you have control over your disease after immigrating here?   هل تشعر أنك تستطيع معالجة مرضك بصورة أفضل بعد الهجرة إلى أمريكا ؟   1. How do you feel you can better manage your disease here in Cincinnati?   كيف تشعر أنك تستطيع أن تعالج مرضك بشكل أحسن هنا في مدينة سينسيناتي ؟   1. Do you feel your children’s medical problems are well controlled?   هل ترى أن مشكلات أولادك الطبية تعالج بشكل جيد ؟   1. How do you feel you can better manage your child’s existing medical problems?   كيف ترى أنك تستطيع معالجة مشكلات أولادك الطبية الراهنة بشكل أحسن ؟  **Mental Health**   1. What social issues affect your family’s health and wellbeing?   ما هي المشكلات الاجتماعية التي تؤثر على صحة عائلتك؟   1. How do you address your own mental health concerns?   كيف تهتم بصحتك النفسية ؟   1. How do you feel about your children’s mental wellness?   كيف ترى صحة أولادك النفسية ؟  **Health Behaviors**   1. What tools and resources do you utilize to maintain your health?   ما هي الوسائل والموارد التي تستخدمها للحفاظ على صحتك ؟   1. What behaviors do you share with other Syrian refugees?   ما هي التصرفات المشتركة بينك وبين اللاجئين السوريين ؟   1. In what preventative health behaviors do you engage?   ما هي المعالجات الوقائية التي تتبعها لنفسك ؟   1. In what preventative health behaviors do you do for your children?   ما هي المعالجات الوقائية التي تتبعها لأولادك ؟  **Healthcare Access**   1. How do you address health problems at home, especially when you cannot come to the doctor or hospital?   كيف تتصرف مع المشكلات الصحية في بيتك ، خاصة عندما لا تستطيع الذهاب إلى الطبيب أو إلى المستشفى ؟   1. What prompts you to seek medical care for yourself or your children?   ما الذي يدفعك على البحث عن العناية الطبية لك أو لأولادك ؟   1. Where do you go to seek medical care: a clinic, hospital, emergency room?   أين تذهب للبحث عن العناية الطبية : عيادة أو مستشفى أو غرفة الإسعاف ؟   1. What means do you use to access the healthcare system? (internet, friends, rides, bus, taxi, EMS)   ما هي الوسائل التي تستخدمها للصول إلى مكان العناية الطبية : الإنترنت أو أصدقاء أو الركوب مع أحد أو ركوب حافلة أو ركوب سيارة أجرة أو الإسعاف ؟  **Healthcare Expectations**   1. What are your biggest health needs since coming to Cincinnati?   ما هي أهم احتياجاتك الصحية منذ مجيئتك إلى مدينة سينسيناتي ؟   1. What do you feel the Syrian refugee community needs here?   في رأيك ، ما هي احتياجات اللاجئين السوريين هنا ؟   1. What do you expect from your emergency room visit?   ماذا تتوقع عند ذهابك إلى غرفة الإسعاف ؟   1. What expectations do you have for you clinic visits and your hospital visits?   ما هي توقعاتك عند زيارة العيادة أو المستشفى ؟   1. Have you ever interacted with doctors or hospitals here in America?   هل تعاملت مع أطباء أو مستشفيات هنا في أمريكا من قبل ؟   1. How did your expectation compare with your actual experience?   كيف تقارن توقعاتك بما حدث معك بالفعل ؟   1. What do you feel could have improved your experience with the doctor or hospital system?   ما هي الأشياء التى ترى لو أنها كانت موجودة لحسنت تجربتك مع الطبيب أو نظام المستشفيات ؟  **Barriers**   1. What barriers do you face in accessing the healthcare system here in America? How is different than healthcare back home?   ما هي العوائق التي تواجهها في الوصول إلى العناية الطبية هنا في أمريكا ؟ كيف تختلف هذه العناية الطبية عن العناية الطبية في بلدك ؟   1. What barriers do you face in implementing treatment plans determined by your or your children’s healthcare providers?   ما هي العوائق التي تواجهها عند اتباع المعالجات التي ينصحك أو أولادك بها الممارسون الصحيون ؟ |
