# Supplementary material for: Beliefs, perceptions, and behaviors impacting healthcare utilization of Syrian refugee children
Source: PLoS One. 2020 Aug 7;15(8):e0237081. doi: 10.1371/journal.pone.0237081 (PMC7413502; doi:10.1371/journal.pone.0237081)
Supplement: S2 File — Statement of positionality of the authors. (DOCX) [file pone.0237081.s002.docx]

Statement of Positionality

The primary author is an emergency medicine physician, specialized in pediatric emergency medicine. Daughter of Syrian immigrants, Dr. Alwan was born and raised in the Midwest, where she has clinically cared for immigrants and refugees for a decade. Her ability to speak the Syrian dialect of Arabic fluently built trust and rapport with participants of this study. Many members of Dr. Alwan’s extended family were displaced by the Syrian war. This experience allows her to empathize with the refugee struggles of attachment and displacement. Moreover, Dr. Alwan designed and implemented this study in an effort to understand and improve the refugee health experience. Her worldview attests that there are duplicities of meaning and relativity of truth. And Dr. Alwan adheres to the transformative research framework. The transformative research paradigm centers studies on the experiences of marginalized communities, analyzing power differentials and disparities in care. In effect, this paradigm links research findings to actionable items that will mitigate disparities, thereby addressing a community’s direct needs. The work highlighted in this manuscript laid groundwork for future interventions directly addressing the refugee health needs elicited in this study. Qualitative research, as an interpretive framework, was adopted to best explore the health needs and frustrations of the Syrian refugee community, directly empowering the parents’ words and experiences.

Dr. Ahmed Beydoun was raised in Syria and was displaced due to the Syrian war. Also fluent in Arabic, his cultural concordance with participants allowed for facilitation of trust and expression of candor from the Syrian refugee participants during data collection.

Sevsem Cicek-Okay is a sociologist raised in Turkey and immigrated to Cincinnati. She focuses her research on the experiences of Middle Eastern immigrants in Americas. Her expertise in qualitative research and Middle Eastern immigration patterns facilitated the data analysis of this study.

The remaining researchers have various experiences with immigrant and refugee health. All researchers are well versed in qualitative research. All researchers were living in the city of Cincinnati during the time of research.
