# Supplementary material for: Beliefs, perceptions, and behaviors impacting healthcare utilization of Syrian refugee children
Source: PLoS One. 2020 Aug 7;15(8):e0237081. doi: 10.1371/journal.pone.0237081 (PMC7413502; doi:10.1371/journal.pone.0237081)
Supplement: S3 File — (DOCX) [file pone.0237081.s003.docx]

Interview 1A

Riham: Okay, I am Riham speaking with a young man, participant number is 01A. Today is May 6, 2017. So, first of all, we are going to begin with questions as I explained to you before we turned on the

recorder. That these questions are a little bit specific, but you answer them at your ease. Okay?

Participant: Okay.

Riham: As in, based on what you know.

Time – 00:30

RESEARCHER REQUESTED NO TRANSLATION

Time – 10:00

Ahmed: The goal of the questions is just to have a starting point we can begin our conversation from, so if you have anything you would like to--. We just want to hear about your experience.

Riham: Okay, so we do not have to ask the questions one after the other but rather can ask in general. So, even though we previously spoke before the recorder was turned on, we are going to begin as if we are getting to know each other for the first time. Okay?

Participant: Okay.

Riham: How many children do you have?

Participant: Two.

Riham: Okay, and are your children young or old?

Participant: No, they are young.

Riham: Young, okay. When coming to America--before you came--did they have any medical problems?

Participant: No.

Riham: Okay, thank God. Did they have any problems--not problems--did they have any things you told yourself that "I have to take them to the doctor to examine them for something"--.

Participant: Of course.

Riham: Okay.

Participant: Certainly.

Riham: What are these things?

Participant: What?

Riham: What are the things on your mind, like things that you thought about with regards to your children's health?

Participant: He had an issue with his tonsils. Now, when he was in Jordan his tonsils used to always get inflamed

Riham: Okay, as in, get infected?

Participant: Yeah, get infected. All the time. But here I feel that he has gotten better without even going to the doctor, thank God.

Riham: Thank God! Could it perhaps be due to the change in climate or something?

Participant: Yeah. It is possible.

Riham: Did you give him any medical treatment there?

Participant: We did not do anything--over there, honestly--may God be pleased with the doctor--he said it was his tonsils so they can remove them for him.

Riham: Yes?

Participant: But we did not remove them. We were afraid to, but since the day we came here, thank God, they stopped getting infected.

Riham: Thank God. How old is he now?

Participant: Four.

Riham: Four years old. Okay. Sometimes children--am I allowed to? Okay. Sometimes children, when they get bigger their tonsils also get smaller, so there are some children that need to have their tonsils removed--.

Participant's Wife: That is right.

Participant: This year, no month passed without his tonsils getting infected.

Riham: How sad.

Participant: Every two weeks they used to get infected. Every two weeks they used to get infected. He got an infection in Jordan--.

Riham: There are a lot of infections.

Participant: The infection he got--maybe about 50, 60 bottles of medication for the infection.

Riham: Wow!

Participant: Yeah.

Riham: Okay.

Ahmed: You did not face such problems here.

Participant: Here? No. Honestly, thank God, no.

Riham: Okay.

Participant: Since he first arrived, thank God, not at all.

Riham: Thank God.

Participant: He did not get infected.

Riham: Does he have any--I mean both of them--do they have a general practitioner they go to?

Participant: Now, as in currently? No.

Riham: Okay. When you arrived here, did they take them to any doctors or not?

Participant: When we first arrived? It has been a while--it has not even been two weeks since they have gone. They took them to the doctor and gave them vaccinations.

Riham: You did not face any problems with these matters--when you went there for the vaccinations or --.

Participant: No, honestly, no.

Riham: Okay, thank God.

Ahmed: You did not face any problems with the language or anything --?

Participant: Language? Yes, we encountered problems with the language. When their mother took them to the hospital, there was no interpreter.

Riham: Oh.

Ahmed: Okay.

Riham: They did not have an Arabic interpreter?

Participant: There was no interpreter, no.

Riham: So, then how did you know that --.

Participant: They called a lady on the phone. She then started speaking with him, and he started speaking with her.

Riham: Oh, a phone interpreter.

Participant: Yes.

Riham: Not an on-site interpreter.

Participant: No.

Riham: Okay. Okay.

Ahmed: Why were the kids going to the hospital?

Participant: For vaccinations. They had--.

Ahmed: For vaccinations.

Participant: They had an appointment for vaccinations. They wanted to vaccinate them.

Riham: Yeah. Because he is four years old, and she is--.

Participant: Six.

Riham: Six. Yeah, around this time they give vaccinations. It is normal.

Participant: No, here, when they first come—vaccinations--the whole world, here, generally speaking--.

Riham: Oh.

Participant: Everyone gets vaccinated when they first come because they give you the appointment. They do.

Riham: Okay.

Participant: They call you and tell you to come on such and such day.

Riham: Okay, how do you get to the hospital?

Participant: Honestly, right now, currently, they--the organization is taking us, but now we are responsible for ourselves--I mean we have to go.

Riham: Okay, but how do you go?

Participant: You have to go by bus, but we do not know how to get around by bus.

Riham: It is hard for you to learn how to use them?

Participant: It is hard!

Riham: Did they give you anything to teach you how to use them?

Participant: They did. They told us how to come and go, but is there someone who is going to learn immediately (lit. in a day and night)?

Riham: No, not at all. Everything takes time.

Participant: For example, every bus has a different look--has a different route. You are afraid if you take a bus that it will take you to a different area.

Riham: And you will not know where you are.

Participant: No, you will not know how to come and go.

Riham: Okay, yeah, and the buses--between me and you--the buses here are hard.

Participant: I mean, they are really hard!

Riham: Some cities are better than others, but here in this area the buses are hard.

Participant: They are hard. They are hard. They are hard for someone to learn.

Riham: But, God willing, with time you will be able to figure them out. Everything takes --.

Participant: You need to know the language like everyone else--.

Riham: Yeah.

Participant: For the buses.

Riham: Yeah, are you finding any problems with transportation when you want to take them to the store or anything else that you need? Like--.

Participant: No-- The shopping area is close to us here, but some days you get a little bothered.

Riham: Okay.

Ahmed: Okay.

Riham: When you think about the problems that are bothering you--domestic problems--okay? Problems, for example, as a father. What are the things that-- how do you say “Wear heavy on your head?” Like, you get bothered from them; they worry you--.

Ahmed: Things that preoccupy you.

Riham: Yeah, things that preoccupy you. What are domestic problems that, like, worry you? Did you understand what I mean or not?

Participant: Domestic problems that I am worried about?

Riham: Yeah, like--.

Participant: The most thing I am worried about is the children. That is it.

Riham: What are those things?

Participant: I mean them getting sick, getting confused about their sickness. How are you going to take them and bring them back?

Riham: Yeah.

Participant: For example, if his temperature suddenly goes up and it's nighttime, where are we going to take them and bring them back?

Riham: So what do you do?

Participant: I did not do anything. I put him his “kammadat” [a cool compress] and waited till the morning so I could take him to the doctor.

Riham: Okay, “kammadat” you said? What does that mean?

Participant: "Kammadat" are --.

Participant's Wife: Water.

Riham: Oh, water.

Ahmed: Like water on a towel.

Riham: Oh, okay, okay. Yes, yes, yes. You mean something cold--.

Participant: Yes.

Riham: ...on his head.

Participant: Yeah.

Riham: Okay, I understand. So, for example, when that happened, did you think about calling an ambulance, calling a friend, or something like that?

Participant: Yes, but as for the ambulance, I am afraid that it will not come for something small. They might tell you--.

Riham: Okay.

Participant: “For something like a fever, it does not come.”

Riham: Okay, okay. So that means--as I am talking to you, it seems that you do know the American system well.

Participant: Yes. They talked to us about it. They informed us at the organization.

Riham: Yeah, “mashallah,” that is something good.

Participant: Yeah..

Riham: Of course, you will find that as time passes you will be able to get to know the country better and everything--.

Participant: Of course.

Riham: It will become easier.

Participant: Of course.

Riham: God willing.

Participant: That is for sure.

Ahmed: Is there anything related to medicine here that you feel you do not know? Or, for example, they did not teach you about?

Participant: No, there is nothing.

Riham: They give you, like, classes in Catholic Charities, right?

Participant: Mm.

Riham: Do they teach you in these classes how to use the ambulance or how to--.

Participant: They tell us, yes.

Riham: Do you find this--how do you say information?

Ahmed: Information.

Riham: … this information complete? Or does it need something else?

Participant: Well, honestly, it is lacking.

Riham: Okay.

Participant: It is not complete.

Riham: So, tell us then. What are the things that are lacking so that we can improve them for refugees--.

Participant: Honestly, I do not know.

Riham: ...that are coming after you.

Participant: Hold on, I need to remember. Just a minute.

Riham: Think about it. For example, is there something you learned later and thought to yourself, “I wish someone would have told me about that.”

Participant: Yes, of course, there are a lot of centers here that you do not know about.

Riham: Okay.

Participant: Someone wants to go there, but, like, nobody knows about them.

Riham: For example?

Participant: Medical centers.

Riham: What do you mean?

Participant: I mean we do not know--we do not know--now, if a child gets sick, we do not know where we would take them. We do not know which medical center we would take him to.

Riham: I understand.

Participant: For example, since I first came here I have never taken my children to a center.

Riham: Okay.

Participant: I treat him. If his temperature goes up, I treat him at home.

Riham: Mm.

Participant: And lowering his temperature.

Riham: Mm.

Participant: As for taking him to the doctor, I still do not know.

Riham: Mm. So they do not give you a list, for example, these doctors--.

Participant: They did not give us anything, no.

Riham: At all?

Participant: I do not know.

Riham: So, do they tell you, for example, what you should do if your son's temperature goes up?

Participant: That is known. In every household, it is known that if a child’s temperature goes up you put some cold water on it.

Riham: Okay.

Participant: That is something known, but as for taking him to the doctor I do not know--where are there clinics? Where is there such and such? I do not know.

Ahmed: Okay.

Participant: But if they give us appointments from the center and say, “Come.” We go. They take us--the organization. It takes us and brings us back.

Riham: But these appointments, like, it is not for something like if he gets sick.

Participant: No--.

Riham: Right?

Participant: The appointments are from them.

Riham: Yes, okay.

Participant: Not from us. The appointments are not from us. They are from them.

Riham: Set up beforehand.

Participant: Exactly, set up beforehand.

Riham: So, if there is a problem now, you do not know what you are going to do.

Participant: Now, if I have a problem now, and the kids get sick, where am I going to take them? I do not know.

Riham: Okay.

Participant: [Indistinct]

Riham: Okay, if, God forbid, God forbid, for example, in the evening--today, you find he has difficulty breathing. He is not able to breathe well.

Participant: I would call the ambulance in that case.

Riham: Okay. That means you have knowledge.

Participant: I would call the ambulance--if something happens bigger than that, I would call the ambulance.

Riham: Okay. Thank God that they are in good health, but--.

Participant: No, thank God. It is a great blessing from God.

Riham: Yes, what am I going to tell you? Are there things that you do with your son or daughter to ensure that their health is good?

Participant: I am always trying to use tricks to get them to eat and drink.

Riham: Good.

Participant: Yeah.

Riham: So you plan what they eat and drink?

Participant: Yes, all the time. I use tricks on them. For example, if they want to go out somewhere, I tell them, “You have to each such and such food so I can take you.”

Riham: Okay. What are the foods that you--.

Participant: Bananas, oranges, apples, things like that.

Ahmed: Fruits and vegetables?

Riham: You mean healthy food.

Participant: Mm. Yes.

Riham: Okay. Is there anything--?

Participant: Also, I have a daughter that does not eat too much. She eats a little--.

Riham: Really?

Participant: Yeah. She eats very little. So, I play a trick on her, and I tell her, "Finish this plate, and I will buy you something."

Riham: Then she finishes them.

Participant: She eats them. She finishes them, whether she likes it or not.

Riham: Are you finding the food here is different than the food you are used to? Are there any difficulties or problems?

Participant: No, there are no difficulties.

Riham: Like, the children--your daughter who does not like food too much, was she like that back in your home country--the same thing?

Participant: Yes, she has been that way since she was back home.

Participant's Wife: She never eats any food at school.

Riham: Oh.

Participant's Wife: From the school.

Participant: We want to find a solution for this thing here.

Riham: That she is not eating school food--?

Participant: No, not school food. She eats little.

Riham: She eats little.

Ahmed: In general.

Riham: Yea.

Participant: Mm.

Riham: Did you speak to the doctor about this, for example?

Participant: Not yet. We did not speak about it.

Riham: Okay.

Participant: I am telling you, it is all just one appointment that they give you.

Time – 20:00

Participant's Wife: They vaccinated them--.

Riham: Oh.

Ahmed: Until now?

Participant: Until now.

Riham: Four months and--.

Riham: ...only one appointment?

Participant: Yes, one appointment.

Riham: Now,

Riham: Oh.

Participant's Wife: And they did it at school.

Riham: Okay. Okay. Not that they took her to a doctor.

Ahmed: Okay.

Riham: Interesting. Yeah, God willing, I can give you --.

Participant: We are missing appointments because of language difficulties.

Riham: Okay, then how do you know you missed an appointment?

Participant's Wife: There is a paper they give us.

Participant: An interpreter calls us and tells us that we missed an appointment.

Riham: Oh.

Ahmed: Oh.

Riham: Is that at Millville?

Participant: Yes,

Participant: We are always missing appointments.

Riham: So they wanted to see you but between --.

Participant: They call, but I do not know how to speak with them [in English].

Riham: They call in English?

Participant: Yeah, they speak in English. I do not understand anything they are saying.

Riham: So how are you going to understand them?

Participant: I do not know. They send papers here. She translates these papers --.

Participant's Wife: He had some doctor's appointments --.

Participant: I had an appointment, and I missed it.

Participants Wife: They told him to return their phone call so that he can reschedule.

Riham: Okay. At least you are taking care of their nutrition. Do you do any exercises at home or outside?

Participant: No, but from time to time we take them to the park right here.

Riham: That's nice. Do you have a park next to your house?

Participant: Yeah.

Riham: That way they can move around. They seem really healthy, praise be to God, but things are different here than back in your home country. There isn’t a lot of walking here. The food here is more fattening. These are things we have to watch out for when it comes to children. I asked you earlier what things you were doing to take care of your children’s health. Is there anything else you can think of that can help promote good health? For example, a gym or being able to go to a bigger park. Are there things you wish you had?

Participant: Yeah, I think about things like that …but now my children are still young.

Riham: When they get older, what are some things in your opinion?

Participant: It will depend on each child.

Ahmed: Okay.

Riham: I also wanted to ask you something. Your wife is currently pregnant, right?

Participant: Yeah.

Riham: Are there things you feel like you need to know after your baby is born? A baby has different needs than a five-year-old. Right? So, are there things you wish you can learn more about regarding the medical system here when it comes to your baby?

Participant: Of course. We need to know what the system is like here for a newborn.

Riham: Okay, please explain some more.

Participant: When a newborn comes we are not going to know how to deal with it. My mom used to --. Do you know the umbilical cord? The umbilical cord scares us. My mom used to deal with it. I personally do not know how to deal with a newborn.

Riham: Okay. When you were in your home country, your mother used to help you a lot with these things.

Participant: Yeah, a lot. We do not know anything about how to deal with newborns. A newborn that is a day or two, for example.

Riham: Right.

Participant's Wife: The umbilical cord is the only thing that really used to scare us.

Riham: Don’t be scared of it. God willing, it will fall off and not cause any problems.

Participant: God willing.

Riham: It does look scary though, you’re right.

Participant: Of course, it is scary.

Riham: Yeah, it looks really weird.

Participant: A child’s life depends on it.

Riham: Yeah, of course. This isn’t one of the questions but I am just curious. Were you in the delivery room?

Participant: Where?

Riham: When they deliver babies there [in Syria], are fathers usually present?

Participant's Wife: No, he would be at home.

Riham: Oh, they do not allow the fathers to be there?

Participant: No, it is not allowed.

Participant's Wife: No.

Riham: Wow.

Participant's Wife: They allow it here.

Riham: Here, the father has to be there.

Participant: [indistinct].

Riham: Also, the father is usually the one that cuts the umbilical cord.

Participant: What do you mean, he cuts it!

Riham: They give you some scissors and tell you where to cut it.

Participant: No, it is prohibited in our home country.

Riham: All the fathers wait outside?

Participant: All of them. No one is allowed to go in.

Riham: How about her mother? Would she be with her?

Participant: Not even her mother. No one is allowed in there. Everyone has to wait outside. Not just outside, but all the way outside of the hospital even.

Riham: Wow.

Participant: When they take her out to another room then they would be able to see her.

Riham: That’s really tough.

Participant: Sometimes you won’t be able to see her till an hour or two after.

Riham: Oh no!

Participant: Yeah.

Riham: Okay. Here they will give him to you immediately. Just so you know what to expect.

Riham: The baby will be a little covered in blood and fluids and they will say, “Here you go, father”.

Participant: In our home country, they clean the baby all the way and wrap him, then give him to the parents.

(1A -- 25:06)

Riham: Oh, wow. It is a different system.

Participant: It is really different.

Riham: Hopefully, she will have a doctor that --.

Participant: We do not know what to expect. How is the system here when a woman gets pregnant? Do they have a special care or not? We do not know.

Riham: They do care for her, of course. Would you like me to explain it to you or not?

Participant: In the system here, do they offer special care for the pregnant?

Riham: Yeah, they care for the pregnant women a lot. They would first see her when she is 12 weeks. Meaning between 8 - 12 weeks along. That would be approximately 2 or 3 months into the pregnancy. Then they would follow up with her every month at least once. Then, near the end, they become weekly visits. The last three months of the pregnancy, she would be seen every week. They observe her closely. They would only do the ultrasound --.

Participant: What is an ultrasound? Oh, an ultrasound! I understand now.

Riham: They do the ultrasound at 18 weeks. Between the 18th and 20th week. That way they can see how the baby is doing. How are you finding the obstetric/gynecologists?

Participant's Wife: My doctor is in [indistinct].

Riham: Where?

Participant's Wife: Millville.

Riham: Oh, in Millville.

Participant: Yeah, in Millville.

Riham: Yeah, yeah. I hope you find that your experience changes after 6 or 7 months. By that time you would have gone a few times and experienced different doctors. Maybe by then, you will notice things that you felt were missing and also things that you liked about your experience.

Participant: God willing.

Riham: God willing.

Ahmed: God willing. From our conversation, it seems that the most thing you are finding difficulty in is the language barrier.

Participant: Yeah.

Ahmed: The second biggest problem was the transportation, right?

Participant: That is right.

Ahmed: Is there a third thing you can think of similar to these issues?

Participant: A third thing, you mean?

Ahmed: Yeah. Anything you can think of. If you feel like these are the only problems, then no worries. We just want to make sure we understand all the problems you are facing. No matter how small they may seem, please share them.

Participant: Honestly, the language and getting around are the biggest issues.

Riham: The transportation you mean.

Participant: Yeah.

Riham: Do you use the internet? For example, if you find that your son is coughing or vomiting.

Participant: Yeah.

Riham: My apologies [regarding the use of an Arabic word for vomiting]. I worked with a lot of Yemeni patients at the hospital I used to work at. They used to tell me the word "tarrish" [vomiting], so I started using it. It isn't a Syrian word, though.

Participant: Yeah, it isn’t.

Riham: If your son is vomiting a lot, do you open a book, go online, or maybe call a friend? Do you call your mother, your sister, or your brother? What do you do?

Participant: If I find that my child is vomiting a lot, I would immediately treat him myself.

Riham: How would you treat him?

Participant: We have some herbs. We boil them and give the child some to drink.

Riham: What kind of herbs?

Participant: “Khalil al-jabal”, for example.

Riham: What is “khalil al-jabal”?

Participant: It is a type of herb.

Riham: Do you know what it is? [Asking Ahmad]

Ahmed: No, I don’t.

Riham: Does it resemble the chamomile?

Participant: No. It is thin.

Riham: Can you find it here?

Participant: Yeah you can.

Participant's Wife: Only at the Arab market.

Riham: Does it have a strong smell?

Participant's Wife: Yeah, that is it.

Riham: Do you boil it?

Participant: Yeah.

Participant's Wife: Yeah, you boil it like herbal tea.

Riham: Maybe it is sage.

Ahmed: Yeah, maybe.

Riham: You can also cook it with the food?

Participant: Yes, exactly.

Riham: Okay, yeah, I know what you’re talking about. My mom used to use it as well. Do you find that these herbs help?

Participant: Yes, they help. That is only if his vomiting is normal. If it is due to an infection, then he needs to see a doctor.

Riham: Okay.

Participant: Up until now, we have not experienced anything like that, though.

Riham: Okay, good thank God.

Participant: Thank God.

Riham: Okay, let’s see what else. You said that you took your children before to get vaccinated. How did you find the treatment of the doctors and nurses?

Participant: I didn’t go with them. I was in [indistinct].

Ahmed: May God give you strength.

Riham: Can you think of anything else?

Ahmed: Yeah. I know that you did not have the need to go to the emergency room till now but what are your expectations if you do go?

(1B -- 30: 41)

Participant: The emergency room is for emergencies, right?

Riham: Yes.

Participant: I would want them to really care for the patient.

Ahmed: Tell us a little bit more about your expectations. Do you expect there to be an interpreter, for example? Maybe you would expect the doctor to speak Arabic, for example. Give us specific examples, please.

Participant: I would expect that there be an interpreter. It is a must.

Ahmed: Okay.

Participant: They should bring one immediately upon the arrival of the patient.

Ahmed: Okay. We just want to see what your expectations are to see if they match with reality.

Riham: How long do you expect to wait in the waiting room.

Participant: I would expect it to be immediately.

Riham: That is the ambulance. What about when you get to the emergency room?

Participant: It needs about five or six hours to even leave the hospital now.

Riham: It will never be immediate, I’ll tell you that much.

Participant: Last time, I left work and went to the hospital at around 8 or 9. I left at 1 pm and that was without even seeing the doctor.

Riham: Oh, no!

Participant: I just left.

Riham: Where did you go? Which hospital?

Participant: I went home. The hospital that is right here.

Riham: Which one is that?

Participant: It is before the 11. That 11 market.

Riham: Is it Good Sam?

Participant: Someone from the organization took me.

Riham: Oh, you called them and they came and took you?

Participant: No, I didn’t call them. She came here to have me sign some papers and saw that I was sick. She told me she wanted to take me to the hospital. She took me to the hospital and we sat waiting from 9 till 1.

Riham: They didn’t call you in?

Participant: They didn’t call me in. They asked me some questions first then had me wait outside till the doctor was ready to see me. This is “wish al dayf”.

Riham: What does "wish al dayf" mean?

Participant: They didn’t even call me in or update me. I just got up and went home.

Riham: What day of the week was it?

Participant: I don’t remember what day it was.

Participant's Wife: I think it was a Tuesday or a Wednesday. It was Wednesday.

Participant: Yeah, it was a Wednesday.

Riham: What was wrong with you?

Participant: You know how I work in a freezer? I work with chlorine and in cold conditions. It agitated me.

Riham: Were you coughing?

Participant: I was coughing and had pain in my throat. I got even more sick from waiting at the hospital. I came home and treated myself with honey and lemon. I was fine after that, thank God.

Riham: Thank God. Do you have any medical problems?

Participant: No, I don’t.

Riham: That is good, thank God. Do you take any medications daily, for example?

Participant: No.

Riham: That is a blessing. I am not going to mention any names, but a lot of refugees that came here have medical problems.

Participant: A lot of them do. Most of the refugees here have something. There are the ones that have heart issues and others that have diabetes. The ones that came with us, a lot of them have illnesses. We know about four families.

Riham: Yeah. This is all new to me but it seems like the ones that have medical issues, are the ones that are being brought here.

Participant: They are the ones being brought here.

Riham: Yeah, it seems like that is the reason they are able to come. Isn’t that right?

Participant: Yeah, the organization that is bringing the refugees are bringing the ones with health issues. [humanitarian cases].

Riham: The ones with a health issues. What about the ones with children? Are they taking that into account?

Participant: They are bringing them, but the most ones being brought are the ones with humanitarian cases. Everyone that they are bringing here has something. Maydah, for example, has the liver problem. There is another one [a woman] close to here that needs kidney dialysis.

Participant's Wife: The Abu Saroor family also has kidney dialysis.

Participant: The Abu Saroor family needs kidney dialysis as well. Most of them that come here have an illness.

Riham: Thank God for health. I have a question that is not for the research but out of my curiosity. Do you pick --.

Participant: The city?

Riham: Yes, the city.

Participant: No, they choose it for us.

Riham: Then how were you and your brother able to come to the same place?

Participant: They chose it for both of us. We are both under one file, that is why.

Riham: When you came, you requested to be together then? That is a blessing.

Participant: We were supposed to also come with my family, but they did not bring our parents.

Riham: They are still there?

Participant: Yeah, they are still there in Jordan.

Riham: May God make it easy on them. Did they hear anything about whether or not they will be coming here?

Participant: They want to make them [indistinct]. Making them [indistinct] takes a long time.

Riham: What is it?

Ahmed: If you are living here and your relatives are living abroad you can apply --.

Riham: Oh, yeah. I understand what you mean now. It is a very long process.

Participant: Very long.

Participant's Wife: They also have a humanitarian case. His brother is paralyzed.

Participant: My father is “majloot” [recovered from a stroke?]

Riham: Yeah, it is a long process.

Participant: That is a problem. When someone from the organization spoke to us before we came they told us that if someone from our family had a critical case, then we would be able to bring them after three months.

Riham: After three months?

Participant: Yes. When we got here though, the matter was different than what we were told.

Ahmed: Did you feel like they gave you a lot of misinformation or false expectations before coming?

Participant: That was it.

Ahmed: Just with that.

Participant: They said that we would be able to bring our family after three months but when we got here we saw that it was actually a really long process.

Riham: When we first entered your house, you said something about your daughter's education. You also mentioned how you didn’t want to stay in this house. When speaking to a lot of the other refugees, we found that many of them did not like the houses they were placed in when they first arrived.

Participant: Yeah, a lot of them did not like their houses.

Riham: When you were saying you wanted to move out of your house, was it because of the house itself or because of the neighborhood?

Participant: They raised the rent on us. It was $800 and they wanted to increase it to $900.

Riham: Oh wow. So, you want to find another house.

Participant: Yes.

Participant's Wife: We still have 6 more months here.

Participant: I signed a year-long contract.

Riham: Yeah, that is how it works here. It is usually a one year contract. Is the charity organization helping you find another place?

Participant: After three months, the charity organization tells us they are not responsible for anything at all. Even the benefits they gave us will be cut off. When someone starts working, they cut off the food stamps and benefits.

Riham: Even the food stamps?

Participant: Yeah. They left about $250 of the food stamps. They were going to cut it off completely.

Riham: Even with having two children?

Participant: Yes. They cut off all the benefits. When we start working we have to reapply and go to court to be able to get the benefits back. It is a long process.

Riham: Are you finding any help from the Rahma organization? Jackie and --.

Participant: Honestly, yeah they are helping a lot.

Riham: Thank you very much,

Interview 1B

Riham: Just like I said, we will now begin. I am with a mother that has two children. Her participant number is 01B. Okay, how are you today?

Participant: Thank God, I am doing well. How are you?

Riham: Thank God. I am glad we have met.

Participant: It was our pleasure.

Riham: I have some questions that I would like to start off with. These questions are a bit specific. After that, we will have a general conversation. Okay?

Participant: Sure, go ahead.

Riham: These questions are going to ask you to choose a number between 0 and 4. The 0 means never and the 4 means always. Number 2 would mean sometimes. It’s a scale. The first question is, how many times did you take your children to see a doctor in the past three months?

Participant: I took my son once and my daughter hasn’t been at all.

Riham: Okay, so one time.

Participant: Yes.

Riham: So would you give it a 0 or a 4?

Participant: I would say sometimes [a 2].

Riham: Okay, I’ll mark it in the middle. Did you have any problems getting to the doctor?

Participant: Any problems? No, we called our neighbors and they took us.

Riham: Okay, then choose a number from the scale.

Participant: Until now there aren’t any problems.

Riham: Okay, so which number should I mark?

Participant: Put never.

Riham: Okay, were there any problems with communication at the doctors?

Participant: Any problems? No, there was an interpreter.

Riham: Okay, so there was an interpreter. 0 means never and 4 means always.

Participant: There were no problems.

Riham: Okay, there were no problems. Did you feel like you were waiting for a long time to get an appointment?

Participant: Yes, I waited for a long time that day and at the end of it all, they didn’t even give me any medicine.

Riham: Okay, then you can choose any number from 0 to 4.

Participant: What does 4 mean?

Riham: 4 means always.

Participant: It was a lot. I got really upset.

Riham: Okay, if your children were to get sick on a Saturday or Sunday or after 5 o'clock, did you find any problems getting them to see a doctor or to the hospital? 0 means never and 4 means always.

Participant: Always. My son was in a bad state at 4 o'clock.

Riham: In the morning or evening?

Participant: In the morning! He was struggling to breathe and had a high fever. I was really upset for him that day, and I had no one to call. At 9 o'clock I called my friend and she came and took me.

Riham: You weren’t able to call anyone?

Participant: I couldn’t call anyone. Everyone was asleep so I couldn’t call.

Riham: When you found that he could not breathe well, what did you do?

Participant: I gave him a cool compresses and gave him honey. He wasn’t able to speak. He would say “mommy” and cry. He wasn’t even speaking! He couldn’t breathe.

Riham: At all?

Participant: At all. You know that cream? [Indistinct] I rubbed some on his chest.

Riham: Yes, I know it. It has a smell like mint or something.

Participant: Yes. I rubbed some on his chest and gave him some honey. He felt a little better and went to sleep. At 9 o'clock in the morning, I took him in. [indistinct, noises]

Riham: We were speaking about your son when he got sick. When you took him in, how were you able to communicate with the doctor and understand what he was saying?

(1B -- 5:21)

Participant: They brought me an interpreter over the phone. That day, I stayed there for too long though. My son was also vomiting at the time. They brought him a pill for his vomiting.

Riham: Did it help?

Participant: They also gave him something to reduce his fever. Yes, it did help. That was all though. They did not write me a prescription for some medicine to take home.

Riham: Why not?

Participant: They said it wasn’t necessary.

Riham: Okay, it wasn’t necessary. Did he get better?

Participant: Yes he got better after a few days.

Riham: Okay. Did you find that you trusted the treatment they gave him?

Participant: Yeah, but I was hoping they would give me a fever reducer to take home with me. He woke up at night with a fever. They didn’t give me one though.

Riham: Did you ask them for this medication?

Participant: I did tell them that I wanted a fever reducer, and they said it wasn’t necessary.

Riham: Why wasn't it necessary? Didn't they tell you?

Participant: They said that they gave him a dose in the ER.

Riham: You told me that you had two children. Are they young or old?

Participant: My daughter is six and my son is 4. The boy will turn five soon.

Riham: How about the girl?

Participant: The girl is six.

Riham: Oh, that’s right. You said that already. Did they have any medical problems when you lived in your country?

Participant: No, but as their dad said, it was just his tonsils that were a problem.

Riham: Okay, when you arrived here you were --.

Participant: Thank God, he got better. It was only that one situation that I told you about. It was really hot and then suddenly became cold. That is when that situation happened to him.

Riham: You think it was because of the weather change?

Participant: Yeah, it was from the weather.

Riham: They do not take any medication daily?

Participant: No, I do not give them anything. Only when they get a fever do I give them a fever reducer. I bought it for them from the pharmacy.

Riham: Which one do you use? Is it Tylenol or ibuprofen?

Participant: Something like that.

Riham: Something like that. Do you find any trouble when reading about the proper dosage of these medications?

Participant: Yeah, my friend got it for me. She speaks English.

Riham: Oh, good. So she told you how much medication to give them based on their weight?

Participant: Yes, that’s right.

Riham: We have a few more questions in this survey, but we can finish them later. Actually, let’s just finish them now…. Okay. Nevermind we will finish them later because they are very specific. You told me that your friend helps you, is there anyone else you turn to for help with medical matters?

Participant: No, honestly those two friends are the most ones that help. They know English and I turn to them a lot. I depend on them. Especially if there is a paper that I can’t read. I send it to them. Everything.

Riham: Okay.

Participant: The other day, about four or five days ago, I was at the eye doctor.

Riham: Okay.

Participant: They wanted to change the appointment because they did not find an interpreter for me. I called my friend and she was the one that interpreted for me, May God reward her.

(1B -- 9:01)

Riham: By phone, then.

Participant: Yeah, if it wasn’t for her, they would have rescheduled my appointment.

Riham: Were you able to see the doctor after that?

Participant: Yes, I did. I had a surgery called “zara qamiya” done to my eye.

Riham: A “zara qamiya”?

Participant: Yeah, it is for the white part of the eye on the surface. I had to put another one.

Riham: Oh, were you born with this problem?

Participant: This happened to me after I delivered my daughter.

Riham: Oh, so you did not have this problem when you were young?

Participant: They told me this happened to me when I was young but the effects of it showed after my pregnancy. My condition worsened.

Riham: Can you see well with it now?

Participant: Yeah, thank God, I can see but they prescribed some glasses for me. I’ve had them for two weeks.

Riham: In one eye or both?

Participant: No, in both eyes.

Riham: Oh, wow.

Participant: The doctors here told me that it is good that I had this surgery.

Riham: You had this surgery done in Syria or in Jordan?

Participant: In Syria.

Riham: Okay. That means you have experience with doctors in Syria and here.

Participant: And in Jordan.

Riham: Also in Jordan.

Participant: I really struggled with my eye problem. I would have stitches. Once a month I would have to go to the doctor because the stitches came loose or to add one in there.

Riham: Oh no.

Participant: I have about 4 stitches in each eye.

Riham: Do you feel pain?

Participant: Yes. If it comes apart it starts to poke my eye. My eye would start tearing and I would know that it came apart.

Riham: Then you would go have it done. Is this a genetic condition? Are you worried about your kids or is it just your side of the family?

Participant: No, no, no. No one else has it. It’s just me.

Riham: They don’t know the cause of it?

Participant: I don’t know. The doctor here just said that it was from my childhood.

Riham: But he didn’t tell you if it was genetic or not.

Participant: No, he didn't tell me. I don’t have anyone else in my family that has it.

[children interrupt]

Participant: Do you have kids?

Riham: No, I do not.

Participant: Are you married?

Riham: No I am not.

Participant: I hope you will get married and have many children soon!

Riham: Thank you. I do love these kids. As disruptive as they are!

Participant: You are good with the kids.

Riham: Yes, remember I am a pediatric doctor. And I have a niece and nephew I miss so much in Michigan. My mom also had a baby when I was 16 years old, so I have many experiences with children.

Participant: Wow, you must have raised your sister then?

Riham: Yes…Yes, alright, I was going to ask you something. I am also new here. I am born here [in the US] but new to Cincinnati. Are you facing any problems --. Of course, you will be facing some problems in terms of knowing how the medical system works here. Tell me a little bit about how you are finding the system here.

Participant: I found a lot of difficulty, especially with the doctors. Since the 25th of March, I have not seen a doctor. Till now I have not been able to go and get examined by the doctor.

Riham: Why not?

Participant: Because of the appointments. It is not till the 12th of this month.

Riham: Okay, what is this appointment for?

Participant: A doctor’s appointment to see the baby.

Riham: Oh, you are pregnant?

Participant: Yes. I am pregnant.

Riham: Okay, do you have any concerns about being pregnant in this country? I mean as compared to your previous pregnancies. You’ve been pregnant twice before this time so do you have any concerns or issues on your mind? Tell me about them.

Participant: No, I don’t have any. I am worried about the delivery part the most.

Riham: Okay, the delivery.

Participant: Every woman feels this way.

Riham: Yes, of course.

[pause to deal with children in the background]

Riham: Okay, we were talking to your husband earlier about things he does to promote good health in your children. What are some things you do as a mother and as one that is always with them at home?

Participant: If I feel like my child is sick, I would sleep next to them and care for them more during that time. I would not be able to close my eyes without watching over them closely. I worry a lot about them.

Riham: Are there some things you do before they get sick? Things that will prevent them from getting sick.

Participant: I give them honey and herbs. It eases their symptoms. Before it gets worse, I give them these things.

Riham: Do you find that these things help?

Participant: Yes, instantly. The other day I gave my daughter a lot of honey. I would dissolve some honey in warm water and give it to her in the morning and evening. She got better right away. It was so that her cough wouldn’t get worse.

(1B -- 15:07)

Riham: Thank God. Is there anything else you use other than honey and herbs?

Participant: Yes, if I feel like they have a fever, I give them a fever reducer immediately before it gets high.

Riham: Okay, that’s good. Do you give them any type of food or just fluids?

Participant: No, I just give them fluids.

Riham: Okay. That is really good. What are some things that you notice in your children that cause you to take them to see a doctor?

Participant: An infection.

Riham: Okay. How would you know if they have an infection?

Participant: When my son has an infection, he starts vomiting. He vomits a lot. If he even drinks water, he throws up. If he eats anything, he immediately vomits. When that happens I know that the boy has an infection.

Riham: When he starts to vomit, what do you do?

Participant: I would go [to the hospital]. I told you it happened just once.

Riham: Just once. Thank God that it only happened once. If it happens again, what would you do?

Participant: I would take him to the doctors.

Riham: How would you take him?

Participant: I would call my friends. Hopefully, we would have a car by then.

Riham: God willing. Do you know how to drive?

Participant: No, I don’t. My husband is learning how to.

Riham: That is good. Hopefully, when you get a car, how would you feel your situation would be?

Participant: I would feel like I would be in a much better situation. I can take my children in immediately.

Riham: Okay, I am hearing from you that the transportation issue is really important to you.

Participant: Yes, it is. Especially when one is sick.

Riham: How are you able to get groceries?

Participant: Honestly, I go walking. It’s right here next to us.

Riham: Oh, that is really good.

Participant: Yeah. Look the supermarket is right there. It’s right next to us.

Riham: That is really nice.

Participant: Yeah.

Riham: That way you can go walking and buy some things.

Participant: Yeah, I go walking and get the stuff.

Riham: Is there a pharmacy here?

Participant: Yes, there is a pharmacy near us as well.

Riham: Okay nice, are you going to it?

Participant: When I need to go, I go. The other day I needed medicine for my headache so I went and got it.

Riham: Okay, nice.

Participant: I showed them by using my phone and they gave it to me.

Riham: Okay, it seems like you are really smart and are able to manage.

Participant: Yeah, we have everything nearby. The supermarket, the pharmacy, and even the bank are close.

Riham: That is really nice. You don’t have any doctors nearby that you can walk to, right?

Participant: No.

Riham: I hope you don’t find yourselves needing the doctors to begin with.

Participant: God willing.

Riham: God willing. Can you tell me a little bit about how the medical system works in Syria for children? Do parents take their children in to get checked at a certain age? For example, do they take them at six months, then at 9 months, or is just when they get sick?

Participant: No, only when they would get sick.

Riham: Here in America, they specify certain times to bring in your children based on their age. They don’t do that in Syria?

Participant: No, they don’t. Only when they get sick.

Riham: For example, if a child is not growing properly --.

Participant: They would take him in, then. If a child is not getting taller or isn’t walking normally, then they would take him in.

Riham: Okay, this is when the parents notice it.

Participant: Yes, when the parents notice it.

Riham: Okay, that is good. It is a different environment. Here, when talking about children’s medicine, a child is able to do certain things at different ages. For example, at four months, he should be able to do so and so. At six months they do this or that. We know these things but it’s good because we sometimes may not notice.

Participant: It will show when they get older. For example, if you don’t feed a small child well, the effects will show when the child is older and it will harm him.

Riham: Exactly.

Participant: We would have harmed the child without knowing.

Riham: Yes, exactly. Things like that. Did they give you a specific doctor that you can go to for these types of appointments?

Participant: The same doctor that gave us the vaccines is our primary doctor. This is only for me and my husband. My children were vaccinated at the school.

Riham: Oh, so they do not currently have a doctor.

Participant: No, they do not have a doctor.

Riham: If you were to have a problem--. For example, you mentioned before I started recording that your daughter doesn’t eat much.

Participant: Yes, that is true. She eats very little.

Riham: If you had a question about this and wanted to speak to a doctor about this issue, what would you do?

Participant: I don’t know. I would need to see a doctor.

Riham: How will you see a doctor?

Participant: I don’t know. I would try by asking our doctor and he will refer me to another doctor. That can also work.

Riham: Okay. Does your doctor treat children?

Participant: I don’t think so. I go to the clinic.

Riham: What does that mean?

Participant: My doctor is in a clinic and not in a public hospital.

Riham: Yes, you mean in a clinic. The one in Millville?

Participant: Yes, Millville.

Riham: Okay, and they don’t see children there?

Participant: No, I don’t think so. I didn’t take my children yet so I don’t know.

Riham: Okay. If you need any doctors, I can give you a list of doctors that I know and they can care for your children’s health.

Participant: Okay.

Riham: In pediatrics, we aim to see a child at least once a year if they are the age of your kids. That way we can follow up on their eating habits and developmental skills. We also see how they are doing in school as well. Medicine isn’t just for the body, it is for everything. Do you know what I mean?

Participant: Yes, everything.

(1B -- 21:11)

Riham: I am going to ask you some more questions regarding your children. Of course, your children are young, one is 4 and one is 6. Did you find that their psychological state changed when they came here?

Participant: No, honestly. It is quite the opposite.

Riham: Thank God. Do you feel like they are at ease?

Participant: Yes, they are at ease.

Riham: Okay, do they have any psychological needs?

Participant: A few days ago, when my daughter’s tooth fell out, she got dizzy and fell. Another time, her brother flipped over and fell on her head and again she got dizzy and fell. There was also a time when we gave her a shot, she also got dizzy and fell on the ground. She turns blue and gets dizzy. I don’t know if it’s because she started getting afraid or what.

Riham: That is really scary.

Participant: It really is. Even if I use water she doesn't wake up. She falls to the ground and turns blue.

Riham: Wow. Did you tell the doctor this?

Participant: I did. I went to the clinic and told them that my daughter gets scared of the shot and gets dizzy. They said it wasn’t a problem we will wake her up immediately.

Riham: At least she woke up, thank God.

Participant: They gave it to her and she did not get dizzy, thank God. Just yesterday she had three shots.

Riham: Oh, this was yesterday? She didn’t get dizzy?

Participant: No thank God, but she got afraid. I was soothing her and distracting her with my words. If I did not do that, she would have fainted.

Riham: When I said that I was going to give her a shot, she didn’t get scared though. Maybe I should bring some gloves with me during the next interview. [laughter] Once the kids see the gloves, they get scared. I always tell them at the hospital [the children], “don’t look at me, the nurses are the ones that give the shots. You can yell and scream when you see a nurse”.

Participant: Yeah. [laughter]

Riham: It is interesting but when I was young I never used to get afraid of getting shots.

Participant: Really? Lucky you.

Riham: I used to like it actually. I don't know why. I must be strange. Look at me now, I became a doctor.

Participant: When I took the vaccine I got dizzy. I didn’t fall to the ground like my daughter does, but I did get dizzy.

Riham: I am the same way. Now, when I first see a needle, I get upset. Once the shot is given to me though, I wonder why the patients react the way they do. It’s really not that bad.

Participant: It can really be scary.

Riham: Anything but a needle, right?

(1B -- 24:12)

Participant: I wish you the best in finishing your studies and becoming a doctor soon.

Riham: I completed my program in medicine, thank God. It is a long process here. In your country, once a person completes their bachelors, they immediately go into medicine. Here, it is different. Once we finish our bachelors, we spend 4 to 5 years in med school. Just like Ahmad who is downstairs. I finished five years of med school. Then it was followed by 4 years of specialization. I completed those as well. Now I am doing my fellowship in pediatrics. It is another specialization basically.

Participant: Sounds like such a long process.

Riham: Yes, it is really long. I have two more years left.

Participant: God willing, you finish soon.

Riham: Pray for us.

Participant: God willing.

Riham: I wanted to ask you another question. When times get tough, what do you turn to? For example, I told you that I have a long way to go to finish my studies and so I deal with a lot of stress. How can I describe what stress is in Arabic? I believe it is “daghit” [pressure]

Participant: Do you mean at home?

Riham: As in mentally and emotionally. When a person has a lot on their mind --.

Participant: What do they do about it, you mean?

Riham: Yes, how do you deal with it?

Participant: I eat.

Riham: There is nothing like eating. [laughter]

Participant: When I get upset, I go and eat.

Riham: That’s good.

Participant: When I get upset, I cry and feel better. I hope I don’t get upset though.

Riham: God willing. Is there anything besides eating and crying?

Participant: That is it.

Riham: Are there things you turn to? Perhaps by searching online or talking to someone.

Participant: I go outside. I get myself out of the house if I can’t take it anymore.

Riham: That is nice.

Participant: I take it out on my children.

Riham: What else?

Participant: That is it.

Riham: Okay, that is good. I know your children are young, but what are some things you teach them to help them cope with their anger or sadness?

Participant: When they get older, you mean?

Riham: Even at this age. Don’t you find that they get upset?

Participant: Yes, they get upset now. I give them something that they like then they calm down.

Riham: Let me just review the questions I have here. Are there things you use that help promote good health in your husband and children?

Participant: Like what kind of things?

Riham: Like a gym, for example. Maybe a particular type of food.

(1B -- 28:11)

Participant: You mean do I take care of my husband and children’s health through food?

Riham: That is just an example.

Participant: No, there isn’t anything.

Riham: Okay, is there anything you feel like you need to help promote good health in your family?

Participant: No, thank God. There isn’t anything.

Riham: Okay, what are some social problems that you feel are affecting the health of your family members?

Participant: Medical problems?

Riham: Social problems.

Participant: There isn’t anything.

Riham: Okay, what are some things you learned from your parents--.

Participant: I learned how to cook from my mother.

Riham: What are some things you learned from your parents that help promote good health?

Participant: In terms of health, I would call my mom if my children had a fever or vomit. Sometimes I would call my mother-in-law. They usually tell me what to do.

Riham: What are some things they tell you to do?

Participant: If they have a high fever, they tell me to give them water and vinegar. Do you know what vinegar is?

Riham: Yeah, of course.

Participant: They said that immediately the fever will go down if I give them water and vinegar.

Riham: Okay.

Participant: Do you know the peel of the pomegranate?

Riham: No. I don’t know what that is.

Participant: The pomegranate.

Riham: Yes, I know what the pomegranate is.

Participant: The peel of it. You boil it and give it to them.

Riham: You drink it like a tea, then.

Participant: Yes, it is bitter though.

Riham: It’s bitter.

Participant: You let it boil a lot then you let it brew.

Riham: You mean the color turns red?

Participant: Exactly. This is really good for diarrhea. Boiled potatoes are also good for diarrhea. Cumin is good for constipation.

Riham: That is really good.

Participant: Yeah, they help me a lot.

Riham: That is really good. Are you able to easily reach them?

Participant: Yes. I speak to my mother-in-law more than I speak to my family. It is kind of hard to reach my parents now. They are in Syria.

Riham: May God protect them.

Participant: Thank you.

Riham: When you came to this country with your husband, was there anyone else here that you knew?

Participant: No. My brother-in-law is here as you know. They came in a different plane than us, though.

Riham: Oh, so you did not arrive at the same time.

Participant: No, we didn’t. They arrived before us by two hours.

Riham: Oh, but you arrived at the same time.

Participant: We came home and found them waiting for us.

Riham: That is a blessing to have someone here that you know.

Participant: We really keep each other company.

Riham: That is good, thank God.

Participant: Yeah.

Riham: I know you said you haven't been to an emergency room here, but let's say you were to go to an emergency room. What are some things you imagine would happen if you were to go?

Participant: I haven’t been to the emergency room here. I only went to the clinic as I told you. I left at 9 am and came home at 2 pm.

Riham: Wow.

Participant: They put you in a room and they go and sleep or I don’t know what. Maybe they go to eat. I don’t know what they do.

Riham: I wish we get to sleep. What else?

Participant: Every hour they come and do something different. They might measure the blood pressure and another time they come in to say a few words and then leave. When they come back, they say a few words, then leave again.

Riham: Okay, is there anything that can be improved that will make you happier?

Participant: Yeah, in Jordan when I would go to the doctor’s, it would only take 15 minutes.

Riham: That’s it?

Participant: Yeah. If there are a lot of people then we would wait an hour at most. It is not like here at all. The wait here is way too long. I find a lot of difficulty in going to the doctor’s here.

Riham: Was this in the emergency room or the clinic?

Participant: No, a regular doctor’s appointment.

Riham: In a clinic.

Participant: No, they put us in a room.

Riham: Okay, this is at Millville?

Participant: I don’t know what Millville is.

Riham: Was it at your primary doctor’s office?

Participant: Yes, my primary doctor.

Riham: Okay, not the gynecologist.

Participant: No, not the gynecologist.

Riham: Okay, is there anything else you can think of that can be improved?

(1B -- 33:20)

Participant: No, there is nothing. We cannot improve anything, anyway.

Riham: Yes, we can. That is the purpose of conducting this research.

Participant: I would like that when I go to the doctor’s office, that I do not spend longer than two hours there.

Riham: Okay, the time spent waiting. What else?

Participant: The time and the transportation as I told you.

Riham: Okay. The time and the transportation. What else?

Participant: That is it. I would also like for them to give me medicine.

Riham: Okay, you would like to be given medicine. What else?

Participant: That is it.

Riham: There is nothing else? You mentioned a lot of great points. I would like to thank you for that.

Participant: It was our pleasure to meet you.

Riham: Thank you. I feel like we finished all the questions. It has only been 35 minutes. It was supposed to be an hour-long interview so is there anything else you would like to add regarding the topics of health, or the environment here? This a chance for you to share your thoughts.

Participant: No, there is nothing else. I feel like I said everything I needed to say. Thank you. I feel like we troubled you.

Riham: Not at all. We are going to turn the recorder off and continue with some of these questions, then.

Participant: No problem.

Riham: That might be easier.

Participant: Okay.

Riham: Thank you very much.

Interview 2A

(2A.I)

Riham: Today is May 12, 2017. My name is Riham and I am here with a father of two children. This is participant number 02A. How old are your children?

Participant: My son is 5 years old and my daughter is 2 years old.

Riham: May God protect them for you.

Participant: Thank you.

Riham: How long have you been in this country?

Participant: I am now starting my fifth month here

Riham: Okay, the fifth month. Just like I explained to you before starting the recorder, the purpose of this research is to allow the voice of a Syrian refugee to be heard and know what they are struggling with in this country. We also want to know what the medical needs of the children are. We are also trying to elicit the expectations of both parents. We want to hear their different experiences. I have some questions but I will not be asking them one after the other. We will answer them in a form of a conversation. I am going to start off by asking you a question. [laughter]

(2A.2)

Riham: Once again, I am with participant 02A. In what ways do you care for your health --.

Participant: This question seems to be about the cigarettes. Cigarettes are really harmful to one's health. I do not advise --. [laughter, indistinct]

Riham: Tell me a little bit more about smoking.

Participant: A father should not harm himself or his children. There is hookah too.

Riham: Do you have a hookah here as well? The translator might not know what a hookah is. Tell me what are the things you care about in terms of your health and your family’s health?

Participant: I care about things like nutrition, and treating illnesses. Things like that.

Riham: Okay, then tell me about nutrition.

Participant: All illnesses come from lack of proper nutrition. If someone eats healthy nutritious food --. Now you have those that eat processed food, fried food, and pre-prepared food that has almost no nutritional value. It does not compare to someone that buys vegetables and fruit and fresh ingredients from the market that you can cook at home. This promotes a person's health. They will not get as sick because they will have a strong immune system. Naturally, the body gets stronger. At that point, if that person was to catch a virus, they could fight it off easily. If a person has a weak immune system then they will need to go to the hospital.

Riham: Other than nutritious fresh food, what are some other things that you bring for your family to use to promote good health? Or maybe your wife does certain things to preserve your children’s good health.

Participant: Yes, we have herbs as well. We first turn to herbs for remedies.

Riham: Which herbs do you use?

Participant: Margoum is good for diarrhea. Cumin and garlic are good for fevers. Lemon and garlic are good for the flu and a cold. You also have ginger for the chest.

Riham: Are you finding it difficult to find these types of herbs here in Cincinnati?

Participant: No, I am not finding it difficult to find them here. I am having trouble finding “bizr el-khili”. This is what we call it back home.

Riham: Tell me a little bit more about it.

Participant: These are seeds

Riham: Black seeds?

Participant: No. It is like a dark beige in color. Do you know the color “jardouni”?

Riham: It is not black but --.

Participant: It’s the color of oil. Oil that is dark in color. Dark olive oil.

Riham: Okay

(2A.2 -- 3:54)

Participant: This “bizr el-khili” [Ammi Seeds] is really good for you.

Riham: Is it a liquid?

Participant: No it is a seed. It comes really small and you need to boil it. It tastes bitter. Medicine can be bitter as we know. This is a natural medicine. They take it for the kidneys.

Riham: Can you say the name of it one more time?

Participant: We call it “bizr el-khili” [called Ammi Seed and comes from the Ammi Visnaga plant].

Riham: Okay so “Bidhr Al-Khali”, “Khali” would be the seed or the plant?

Participant: “Khali” is a plant. I will show it to you on my phone.

Riham: Yeah, show it to me when we end because I did not hear about it before.

Participant: Exactly. This seed --.

Riham: You could not find it here.

Participant: If I can get the scientific name for it, I can find it. For example, we call this plant "khaleel aljabal". In Jordan, they call it "hasbaran". In America, they call it --.

Riham: It is called lemon verbena as I told you.

Participant: Yeah. Now if I had the scientific name, I could find it anywhere in the world.

Riham: Yeah.

Participant: Now, if you were to go to a pharmacy that sells herbs, if you gave them the scientific name, they would help you find it.

Riham: Hopefully we find it for you. I don’t know what it is.

Participant: Yeah, God willing.

Riham: You mentioned the importance of nutritious food. What else do you use to promote good health for you and your family? Other than smoking a cigarette that is.

Participant's Wife: How do you like your coffee?

Participant: Get out!

Riham: I honestly don’t drink coffee. Thank you. I don’t like how it tastes.

Participant: Leave! Get outta here! [shouting at an animal?]

Riham: I like tea though. Do you have any tea left?

Participant: I use herbs.

Riham: Herbs that are called tobacco [said jokingly]?

Participant: I like it.

Riham: Only twice a day?

Participant: Yeah. What herbs did we use in the past?

Riham: We finished that topic. Let's talk about smoking. I would like to talk to you about it. Were you smoking for a long time?

Participant: I started when I was 14 years old because it was what “real men” did and I just never stopped after that.

Riham: Are you trying to stop or reduce the number of times you smoke?

Participant: Yes, honestly, I am trying to not buy one pack.

Riham: You would buy ten? [laughter]

Participant: I am trying to buy one pack and have it last me the whole week.

Riham: Okay, so rather than one pack a day.

Participant: Yeah.

Participant's Wife: He is trying to limit it a lot.

(2A.3)

Riham: We had a couple of guests come in and interrupt. We are going to continue now. This is participant 02A.

Participant: We were talking about smoking.

Riham: Yeah, we were talking about smoking.

Participant: I am only trying to smoke a pack a week now.

Riham: That is really good. That means you have reduced it by a lot.

Participant: In America, it is prohibited to smoke at the workplace. We can only smoke during the break. This has helped me cut down smoking.

Riham: That is really good. May God reward you. Ramadan [the month of fasting for Muslims] will be here soon anyway.

Participant: Exactly, it a good time to cut down because Ramadan is coming. I heard they have ways of treating smoking addictions.

Riham: Yeah, there are.

Participant: I heard that there were some patches of some sort.

Riham: Yeah.

Participant: Till now, we have not seen any doctors.

Riham: Why not? You’ve been here for a while though.

Participant: We didn’t meet with any doctor.

Riham: Do you have a primary doctor?

Participant: No, I do not currently have one.

Riham: Okay, weren’t you supposed to be given one during the first three months at least?

Participant: The charity organization that is responsible for me --. What do they call them? My caseworker --.

Riham: Which one? Catholic Charities?

Participant: Yes, that is the one. I may have missed a lot of appointments. Normally, those that come new to this country, go to see a doctor immediately. They would need vaccines and what not. They get evaluated. I have not seen a doctor yet.

Riham: Yes.

Participant: Till now I have not been seen by a doctor once.

Riham: Legally you were supposed to.

Participant: Legal or not, I should have at least gotten the vaccines. We did not go even once.

Riham: You should tell them.

Participant: God willing, I will tell them.

Riham: What do they say when you tell them that it's been a long time and you still haven't seen a doctor.

Participant: The organization that has my documents and file should be the one responsible for taking care of this. There are shortcomings. Negligence. I have not been to the doctor.

Riham: That means your children have also not been to the doctor, right?

Participant: My children were vaccinated within a week.

(2A.3 -- 2:45)

Riham: You have been here for five months.

Participant: Yeah we are starting the fifth month, and till now I have not seen a doctor.

Riham: Why did it take long for your gets to get vaccinated?

Participant: It’s been the organization’s shortcoming. I have missed many appointments because of them. They are not informing me.

Riham: They do not even call and tell you?

Participant: Exactly. They do not tell me and the appointment passes. Now the way it works is that the hospital calls the organization and the organization calls the hospital. The organization would call the hospital and tell them that a new family arrived and that they need appointments. The hospital then makes appointments for this new family and calls the organization back to inform them of the dates and times. The organization does not inform us and so we miss the appointment. When you miss an appointment like this, you need months till they can reschedule.

Riham: Okay, I get what you’re saying. In your opinion, is there anything we can do to improve the situation so that this does not reoccur?

Participant: This is important for me and for others of course! Because we are talking about vaccines.

Riham: Is there anything we can do to improve the situation?

Participant: You could call the hospital and make an appointment for me. You tell me…

Riham: Okay, so if the hospital talks to you directly without going through the organization --.

Participant: I’ll need the information in Arabic, though. They speak in English and I do not understand them.

Riham: If they called and spoke to you in Arabic and informed you of everything, the appointments, will that solve this problem? The purpose of this research project that I told you about is to improve these matters for the Syrian refugees, right? I can think of some things but I want you to give me your suggestions.

Participant: I did a lot of things. I went and talked to the director of this organization. There were others that also talked to them from the Rahma organization at the Mosque. They talked to them and sent emails to the charity organization. I went to the hospital even. The hospital told me that I missed all my appointments. I told them that I had absolutely no idea of these appointments. In fact, I was actually waiting for them. It’s been five months and I have been getting sick and have still not been able to see a doctor nor had any vaccines. This also caused an issue with the children. When we tried to register them at the school, the school told us that we were to blame and that we did not want to put our kids in school. A child is not able to start school without getting vaccinated. So the children aren’t vaccinated. This time that we had to register them for school has passed as well. They did not blame the vaccines, they blamed us.

(2A.3 -- 6:06)

Riham: So this was due to missed appointments?

Participant: Of course.

Riham: Okay, if we were able to improve the matters, what do you think about this idea? Let’s say once a refugee family arrives, Catholic Charities informs the hospital and gives the hospital a number to reach the family. Then the hospital directly contacts the family without going through Catholic Charities. Do you get what I mean?

Participant: Yes, that I agree with. We speak directly to each other.

Riham: In order to improve the situation. The people are saying they did not hear about the appointments and the hospital is saying that the people are not showing up to their appointments.

Participant: You are suggesting that this matter is taken care of from the very beginning of the situation.

Riham: Yes, to involve the hospital or the health department from the very beginning.

Participant: In that case, you will need to arrange for transportation as well. If a refugee has only been in the country for 2 days, they will not know how to go and where to go for the appointment.

Riham: No, this wouldn’t be something the refugee is responsible for. It would be the opposite because we want to help the refugees. This project is so that we can help the refugees, right?

Participant: Right.

Riham: One solution we are thinking about after hearing other people’s opinions, is that what if we were to make a rule that the hospital should receive the contact information of any refugee child and that the Children’s Hospital should then take care of this matter? I don’t know if you know this about Children’s Hospital, but their goal is to really help every child.

Participant: Exactly. That would be much better. You just pinpointed the problem. You have found the wound. These charities are in high demand and their employees cannot keep up. Twenty families are coming and they do not have enough employees or workers to keep up with them all. It is like one person is taking care of ten families. It is hard to help them all. Since they cannot help with everything, it all stops.

(2A.3 -- 8:31).

Riham: Every family has their own issues and their own needs.

Participant: This would be even better. If the hospitals were the ones to get the files of the refugees, it would be a lot better. They should take over the health records of refugees.

Riham: If your son was to get sick, what would be a sign for you to go to the hospital or to see a doctor?

Participant: A fever.

Riham: Okay, a fever. What else?

Participant: A fever.

Riham: Is there anything else besides a fever?

Participant: The first and foremost thing is the fever.

Riham: How do you measure a fever?

Participant: By touching. If a child has a high fever, I would take him to the hospital. If he is not getting better along with a fever, then I would take him.

Riham: Okay. In these five months, have you taken your kids to the hospital?

Participant: I took my daughter. She spent three days with a high fever. We used a cold compress and gave her some ibuprofen. The fever would go down and back up then down and back up again. That means there is a problem. The girl has a problem clearly.

(2A.3 -- 10:36)

Riham: That’s right.

Participant: That means there is something wrong with the girl. We took her at 11 pm at night.

Riham: Where did you take her?

Participant: What is the name of the hospital?

Participant's Wife: Children’s Hospital.

Participant: Children’s Hospital.

Riham: The same hospital then.

Participant: Yes, Children's. That day the doctor came along with an interpreter. I brought her in to the emergency room. That day we had a problem with the hospital. When you bring a child there, they take a long time till they call them in.

Riham: They take longer than you can wait?

Participant: Exactly. They take too long.

Riham: How long did you wait?

Participant: I told the woman that she had a high fever. She told me to sit and wait and I said, “No”. I said I wanted her to see a doctor right away.

Riham: In Syria, is there any waiting when you go to see a doctor in an emergency room?

Participant: No. You would go in immediately in Syria.

Riham: Immediately.

Participant: Yes, immediately. If it’s an emergency, there is no way you would wait.

Riham: We have a completely different system then. Here people wait longer.

Participant 05A: I would like to say something. I am the most one that suffered from this hospital situation because I have nine children. One of them is my son and we came here for an emergency medical reason. They gave him a shot and the illness caused him to become paralyzed. First of all, the phone calls are always automated. I keep trying to say, "No English, Arabic". I really struggled with this issue. If I had an appointment, I would not know anything about it. Secondly, when they talk to us they do not tell us which department. I would be sitting next to a guy who speaks English and he would translate for me that I needed to bring my son Ahmad back to the hospital. When I would try to call the hospital, they would tell me that I needed to know the right department. How should I know which department? I would tell them that a number called me and told me this and that and that I needed an interpreter so that I can understand what was said. They would keep asking which department they should transfer me to. Which department, I don’t know! They said they had an Arabic interpreter but that I needed to know the department. I told them I do not know which department because I have nine kids. This is something we are really finding difficulty in. I have been in this country for three months now. I missed many appointments and I do not understand anything. Same thing with the schools. Every day, at around 4 or 5, they call me. I do not understand a word. Bla Bla Bla. If they left a voicemail I would still not understand. The second thing is that I am sick. It's been four days now. I fell down in the organization itself. I had a stroke. They took me to the ambulance. By the way, the UC hospital had all my paperwork and everything set from the day I arrived. Today, however, I broke out in a rash all over my body. Maybe it was from the medicine or shot. They told me I had to go see my doctor. How am I supposed to know how to make an appointment with my doctor? Then there is the option of the emergency room but no one can go to the emergency room unless they are in critical condition or something. I don't know how to make an appointment with a doctor. Some people might come and offer to help with translating, but they say that legally they cannot talk to us outside of work.

Participant 2A: To put it in simpler terms, no one in the hospital is telling us how we can navigate the system. No one is helping us diagnose our problems. How to get treated for the illness we have, for example. Abu Ahmad, did they give you a diet to follow or tell you what you should or shouldn’t eat for your illness? A patient needs to understand their illness in order to get treated.

Riham: Of course.

Participant: Now if I were to tell you what your illness is and I would give you medicine that is part of the treatment. It’s half of the treatment even.

Riham: Of course.

Participant: If I were just to leave you, ignore you, without telling you anything – there becomes an uproar in the body. These spots on his body could cause him a psychological problem, for example.

(2A.3 -- 15:35)

Riham: Of course.

Participant 04A: My daughter has stomach migraines. When it comes to the patient, they don’t give them the right medicine that can cure them. They are just experimenting. It is not just experimenting. They are just giving out pain relievers. They are not giving the patient the medicine they need to cure the illness, they are just giving out pain relievers. My daughter now has migraines because of this problem. They were just giving her pain relievers and not medicine to treat the migraine itself. Every day I have to go pick up my daughter from school. She is complaining of stomach pains. Almost every day I have to go and pick her up. They are not giving her the proper medicine for her illness. They wait until the illness becomes worse and difficult to treat. Only then do they give her medicine. After what? After a person isn’t able to bear it anymore. On top of that, the medicine they give at that point doesn't even cure the illness. My daughter is 20 years old and she had to pay the price for this. Her migraine has now increased to 60% in her intestines. Every few days you would find her on the floor in pain. They told her that there is no cure for her illness. We asked them, "how is possible that there is no cure?" If cancer has a cure, then how is there no cure for this? How is that possible? They found a cure for cancer so how come this illness doesn't have a cure? It wasn't until after my wife made a big deal about it that they cared. They just give the patients pain relievers. They don’t seek a complete cure. Just pain relievers.

Participant 05A: The same thing happened to me.

Riham: Excuse me, I just wanted to ask a question. In your opinion, what do you consider to be a complete cure?

Participant: In my opinion, if a person has an illness, then their illness needs to be diagnosed. The doctor needs to explain to them what they have or do not have. He has to tell the patient what will harm them and what will benefit them. He should tell the patient what medication to take. He should ask the patient what they are eating. What their diet is. The doctor needs to join the patient on his journey to treating his illness. So much that the patient becomes the doctor. To heal, the patient has to understand his own illness as if he is a doctor.

(2A.3 -- 18:07)

Riham: Yes.

Participant: In Syria, if you would go to the doctor for a tooth pain, for example, he would ask what did you eat, what have you tried, then he would fix it for you. He might tell you that you have an infection that spread to this area. He would mention the reasons as well. He will find out what you ate and drank to cause it.

Riham: Do they ask you questions like that here?

Participant: No, did they ask you these types of questions?

Participant 04A: They would do these things but they just give pain relievers in the end. The important thing is that they cure the illness completely.

Participant: That man is talking based on his own experience.

Riham: We will speak to him, God willing. Sir, I am planning to speak to you for a longer time so that I can understand your situation.

Participant 05A: The same thing happened to my daughter. Today, we took her to a hospital called Millville. [indistinct]. My daughter could not sleep at night from her stomach pain.

Participant 04A: You should speak to my wife as well. My wife is very eloquent. She has had many experiences with hospitals and the medical system because of our many kids as well.

Riham: Yes, I will plan to speak to you both and your wives. Let me stop the recorder. Give me a minute, please.

(2A.4)

Riham: The goal of this research is to allow the voices of the Syrian refugees to be heard. To know what they are struggling with and be able to improve the situation by working together. Just like you said about the doctor joining the patient on his journey. We need to work together so that we can find a solution. I hear you saying you don't want to even come to the emergency room, to begin with. It is an inconvenience to spend four or five hours waiting. At the same time, I, as a doctor, don't want to --.

Participant: I got sick!

Riham: Yes, I am sure you did. You are going to get sick because everyone around you is sick.

Participant: When I came to the emergency room and sat waiting for 5 or 6 hours, I got sick. I got emotionally and physically ill. Psychological illnesses increase the physical illnesses.

Riham: For a person to sit and wait, it can be a headache. On top of that, they leave the hospital without feeling like there is a cure. That is a real problem.

Participant: The more a patient knows what they have and how it can be treated, the better they will heal.

Riham: Of course.

Participant: Like I told you earlier, this all goes back to nutrition. Processed food lacks nutrition. The doctors need to ask what a patient is eating. Abu Ahmad, if you were eating store bought food, then, of course, you would get sick. If you were to buy something fresh though, and you cook it at home, it will be a different case. All these things lack nutrition and it is all we eat.

Riham: Is there anything else that you do to promote your psychological well being?

Participant: Yes.

Riham: What are some things you do that help you deal with psychological problems. That help you deal with the stress that you feel. Other than your cigarette [laughter]

Participant: The other day I cut my hand with the knife. I was doing something and cut my hand. I bandaged it up and forgot about the pain. If I keep thinking about it, it’s going to keep hurting me and the pain will be more severe than it already is. We should not think a lot about our pain.

Riham: That is good. My other question is --.

Participant: If we do not leave the wound alone – then it keeps hurting us!

Riham: Of course. My other question is what are some things that you do to help your mental and emotional health? Let us say you are upset, and you are dealing with a lot of stress. The bills keep piling up, for example.

Participant: Exactly.

Riham: When things are not working out for you. What can you do about it?

Participant: I start to smoke and I start to “inhirif” [deviate].

Riham: What do you mean by that?

Participant: Nothing is working out for me.

Riham: What does the word “inhirf” mean?

Participant 04A: It means he's going to smoke weed.

Participant: Take it easy, man. Let’s starting with alcohol first [laugher]. If I lose control of things I am going to have no choice but to turn to drinking and smoking. I would have nothing motivating me to resume my life or to live. On one side I have the illnesses and on the other side, I have the house and responsibilities. When I am suffering physically and mentally, all these pressures, I'm going to explode.

Riham: Is there anything you do that helps you cope with it?

Participant: Are you asking seriously or hypothetically [jokingling]?

Riham: Seriously. For example, by talking to your brother or to your wife? You might go running outdoors, for example. What do you do when things build up?

Participant: For me. We are Muslims. “If you get sick then He will cure you” [verse from the Quran]. There is no cure but it is in God’s hands.Some people may not turn to their faith, or pray, or say, “Thank God”. “If any hardship afflicts you --”. [Pause]

Riham: Is that a verse from the Quran? I don’t know it.

Participant 05A: We got to the mosque. We pray together. We meet one another.

Participant: Yes. Basically, it means that nothing afflicts you except what God has written for you. Everything is destined. Some people are not able to do this. Some people might deviate and turn to drugs and alcohol and other problems, and they might ruin their own health more and more.

Riham: So you turn to the mosque and your faith? To your faith and prayer.

Participant: Yeah, we are Muslims. We don't need to get into this right now.

Riham: It is a good question though. If a doctor knows this thing then they can give you something that helps you regarding this matter. For example, if a refugee was to come to the hospital and he doesn't know anyone, if we were to know that this refugee turns to his faith for healing --.

Participant: Of course. You just clarified it for me --.

Riham: Do you understand why I am asking you this question?

Participant: Of course, because the doctor can give this patient some relief by only using a word or two!

Riham: Exactly.

Participant: He can say this “If you get sick then He will cure you” [verse from the Quran].

Riham: Okay.

Participant: “If you get sick then He will cure you.” This is a verse from the Holy Quran. A patient will find relief when hearing this. If only the doctor could say this.

Riham: Okay.

Participant: The patient will get cured.

Riham: If a doctor or a nurse knows this belief system then they would be able to help the patient feel more at ease?

Participant: Of course.

Riham: If they were to remind them of their God and what their life means.

Participant: “We belong to Allah, and to Him, we shall return” [verse from the Quran]. There are many things. Look, the psychological state affects healing. It is not just the medicine. The medicine makes up a fourth of the cure. Three fourths of it is the mental state of the person. When a person feels at ease with his doctor and feels like their doctor really cares about them, and that this doctor is looking into everything that might be harming this patient, this will put the patient at ease. This will hasten healing.

(2A.4 -- 6:45)

Riham: Yes.

Participant: When a patient feels like the doctor genuinely cares. That the doctor is really trying to find the cure. This will speed up the recovery process for the patient and put them at ease mentally and emotionally. Before prescribing medicine, it is important to give the patient this.

Riham: Thank you for sharing that point. It is really helpful. This will help the doctors and the refugees.

Participant: Of course. If a patient sees that his doctor cares, then that will put them at ease. Healing ones emotional and mental state is the first step to fully recovering. The second part of healing would be the medication itself. They go hand in hand.

Riham: Is there anything else that comes to your mind that you would like to share? What your wishes are for the medical system here and for the refugees, for example. This is an opportunity to share your thoughts.

Participant: Things that will improve the situation is having mental health treatment. A refugee is not in the same psychological state as a native, is he? His mental state is not the same as one that is settled or a visitor even. There is a huge difference. We are talking about a refugee of war. He isn’t just visiting. He is not coming for business or anything like that. You cannot compare the two. This refugee will have mental illnesses due to the war and having to migrate. He would be someone that was separated from his family and his brother. They need to ease things on this person.

*04A: They need to help these refugees heal mentally. When stress piles up a person can lose it. A man explodes. With all these pressures.*

Participant: The most dangerous thing would be to overlook this aspect of this person’s humanity. If attention is not given to this matter, he will only get sicker. He needs to feel cared for and to feel empathized with. They would need some compensation for that pain and to feel like they will not be left to deal with it on their own. If this aspect is neglected, it can ruin his life. Mentally, physically, he will deteriorate. Nothing in his life will be stable. Not for him or for the whole society. The whole society will get negatively affected by this negligence.

Participant 04A: When we first arrived, we were placed in a really bad home. It was filled with snakes, bugs, creatures, and raccoons. My kids woke up to find a raccoon at the window. In just the first month, the children all became depressed. My daughter got sick for that reason. She became sick because of depression. It was because of the depression. She wouldn't dare go out to the backyard. They were just locked up in the house all day. We already had to deal with the war and now there was this added stress? We need to put refugees at ease and remove stress from their lives. In order for the refugee to not feel like they are refugees.

Riham: Okay. Is there anything you would like to add before we end?

(2A.4 -- 11:56)

Participant: Yes, there is so much to say. Let these men speak.

Participant 04A: Yes, yes! Speak! They are placing us in jobs that are really hard for us. They are beyond what our bodies and age can handle.

Riham: Let me just end with the participant 02A and then your turn will come.

Participant 04A: It makes no difference to me. I am just reminding him so that he can speak. Go ahead.

Participant: Am I going to be on Facebook featured? [indistinct] [laughter]

Riham: You can speak about work too.

Participant: Like others are saying, they are placing them in homes that are terrible. Abu Ahmad, for example, had shots fired at his house. There were drug fights in the area and shots fired. He spent three months in that home like a prisoner. It is worse than a prisoner because a prisoner knows that he is locked up. However, to have your door open and the ability to be free but to feel like a prisoner and live in fear --. Don't mind me, my brother.

Participant 05A: No, it’s not a problem, go ahead.

Riham: God willing, we will have session recorded for you both. I assure you both.

Participant 05A: If I spent three days speaking, I will not finish.

Participant: The point is that when these refugee families are coming to America, they are being placed in poor housing situations. They are cut off from life and others. Even if it had people around, they aren't the type of people you would want to interact with. They had critters.

Participant 05A: We want to get it all off our chest through your project.

Riham: What does “fadfid” mean?

Participant: To empty it all. To take it off our chests. They forced him to stay in that home for three months just because the contract says three months.

Riham: Tell me about your job.

Participant: Originally, my job was a baker. I make and bake bread. I deal with the oven and the fire. Now they placed me to work in a freezer.

Riham: They took you from the fire and placed you in ice.

Participant: A freezer, and water, and cutting up veggies. It is not working out for me. I have no choice though. We did not learn the language yet. It has been four months and all we know how to say is "Good morning" and "No problem". The other one is "How are you". Every day I use those phrases. [indistinct laughter]

Riham: You do not take any English Lessons?

Participant: What English lessons? Who is going to give me English lessons?

Riham: I thought the organization was offering them?

Participant: The organization took care of us for two, three months then they told us to go and manage on our own. They said they were no longer responsible for us and that we had to pay our own rent.

(2A.4 -- 15:21)

Riham: They don’t offer lessons though?

Participant: If they place you in a job, how are you going to have time to study?

Riham: That means you are not going to your lessons.

Participant: No, I can’t go anymore. I leave my home at 6 am and come back home at 7 pm. All work. I attended the lessons for one month but it was filled with holidays. I went the second month for a bit, but no one understands those lessons anyway. They have two teachers that are old in age. They are volunteers and may God reward them. [laughter, appears to imitate them] They speak to each other and we are just sitting there watching them. They are speaking and we are like “a deaf person at a wedding party” [Arabic saying]. Even if you translate this deaf person will not understand a thing at all.

Riham: Hopefully, the translator understands what you just said.

Participant: For example, the phrase “open the door.” Is it “close”?

Riham: “Close” means to close the door.

Participant: Mr. Fizer pulled his hair out with me. He kept telling me to close the door and I did not understand him. Over and over and over. He is telling me to close the door.

Riham: They are not starting with the letters and the basics, then.

Participant: Exactly. For example, let's say you are a foreigner. If I want to teach you Arabic, I wouldn’t tell you to “go and close the door”.

Riham: I wouldn’t understand a thing.

Participant: If I break it down for you slowly by saying each letter, slowly, you will understand it that way.

Riham: Yes

Participant: If the teacher would tell me a lot of things, I wouldn't understand. I would need the letters, the words, and the basics. I need to learn the pronunciation. My tongue is used to the Arabic language. It's a muscle. The Americans here they make a nasal sound when they speak. I noticed they did that. I tried to speak from my nose, and boogers came out. I stopped wanting to speak from my nostrils.

Riham: The people from Damascus also kinda speak that way.

Participant: Not completely though [speaks through his nostrils]. [laughter]

Riham: You mean the pronunciation then.

Participant: The Arabic language comes from the heart. When you speak in Arabic, you speak from the depth of your throat. It has to do with your muscles and physics. We are not used to speaking through our noses. They need to teach it to us. In the way of physics. For example, like the letter “P”. I spent a long time till I learned it.

Riham: If you spent your whole life trying, you will not learn it. It is really hard for Arabs [to pronounce].

Participant: “P”. “P”. “P”.

Riham: It seems like you learned it.

Participant: It comes out as a “B”.

Riham: My dad has been here for 30 years and he still says “B” instead of “P”.

Participant: I know how to pronounce it when it is alone. “P”. If it comes in the middle of a sentence though, I don’t know how to. Who knows how it will come out. Maybe some gum will come out with it. One time I was trying to pronounce it while having a cigarette in my mouth. The cigarette fell out with the letter. It was an embarrassment. [laughter]

Riham: Try saying the word “parking”.

Participant: This word “parking”, will come out as “barking”. I told a guy, “I need parking”, he replied by saying, “go barking wherever you want”. I had an Arab with me and he said that I was telling him that I wanted to bark. He said, “go bark wherever you want”.

[laughter]

Participant: That is it! After all this, we have become dogs barking in streets. [laughter]

Riham: Is there anything else you would like to say before we end?

Participant: No, there isn’t anything else. May God give us good health and calm the world down and make it easy and [breaks out into singing].

Interview 2B

(2B.1)

Riham: This is Riham. I am here with participant 02B. She is a mother with two children. Okay, let us begin. Hello.

Participant: Hello.

Riham: Okay, i will leave the recorder here. How are you?

Participant: I am doing well, thank you.

Riham: Thank you for welcoming us on such a short notice.

Participant: Don’t mention it. This is our pleasure.

Riham: As we mentioned earlier, this research includes asking parents questions about their kids and their experiences with the medical system in Cincinnati. Alright, so how long have you been here?

Participant: I have been here for five months.

Riham: How are you feeling.

Participant: Thank God, I am trying to acclimate to it slowly.

Ahmad: It takes time.

Riham: Yeah, everything takes time. Before coming to this country, did any of you have any medical problems?

Participant: No, thank God, we did not have any. I am hard of hearing though.

Riham: In one ear, or both?

Participant: Both. I am wearing a hearing aid.

Riham: Oh, I see. That is great that you have that. Other than this problem, do you have any other medical problems? What about your children?

Participant: No.

Riham: Okay, thank God. Have you been to any doctors here or not yet?

Participant: No, I have not been to see a doctor.

Riham: Did your kids go?

Participant: Yeah, I took them twice or three times.

Riham: Okay, where did you take them?

Participant: To the clinic. In Mill--….

Riham: Millville?

Participant: Yeah.

Riham: Then that was from the appointments they made for you when you first arrived?

Participant: Yes.

Riham: Was it a general appointment or you went when they were sick?

Participant: It was just a general appointment. It was the vaccine appointment. They got vaccinated. It was a general checkup at first. They wanted to examine them when we first arrived. They made the appointment and we went to it.

Riham: Okay then let us speak about your experience at these appointments. When you went there, did you find any problems with communication?

Participant: They gave us an interpreter from the start. They immediately called the interpreter and he came right away. We did not have any problems, thank God.

Riham: Did you find yourself understanding everything they said?

Participant: Yes, thank God.

Riham: Did they prescribe to you any medication?

Participant: No.

Riham: Okay, did your kids get sick at all during the 4 months of being here?

Participant: No, thank God. One time my daughter got a high fever and she had trouble breathing. I took her to the emergency room. They measured her fever and gave her some medicine.

Riham: What happened next?

Participant: She felt better immediately, thank God.

Riham: Did you face any problems when you were in the emergency room?

(2B.1 -- 3:15)

Participant: No, thank God. It went smoothly and quickly.

Riham: Thank God. Which hospital did you go to?

Participant: The hospital for children.

Riham: Children’s Hospital?

Participant: Yes.

Riham: Okay, because they have several branches. Did you go to the main one?

Participant: I went to the emergency room. Honestly, I don't know which branch.

Riham: Was it really big or small?

Participant: It was small.

Riham: Was it close to your home or far?

Participant: I don’t know.

Riham: Okay, no worries. How did you go there?

(2B.1 -- 3:40)

Participant: We went with my husband’s friend. It was only 20 minutes away I think. It wasn’t too far.

Riham: Okay, so it wasn’t that far. The reason I am asking is because there is one for critical cases and another one that is called urgent care that deals with non-critical cases.

Participant: No, it wasn’t a critical case.

Riham: Okay, did you find that the wait was long?

Participant: Yes, we waited for a long time. We left at 7 and came back at 12.

Riham: At night?

Participant: Yes. It did take a long time. The kids started to cry at the end because of the long wait. We waited a very long time. The doctors would be in and out. I lost count of how many doctors examined her. First one doctor then another doctor then one above that one! It felt like all the doctors at the hospital examined her.

Riham: Thank God.

Participant: Yes, thank God.

Riham: You aren’t used to this kind of thing?

Participant: No, I am not. It is not like that in our country.

Riham: How is it in your country? I don’t really know, honestly.

Participant: If they see that a child has a fever, they give them a pain reliever and send them home.

Riham: Just one doctor sees the child?

Participant: Yes, just one. It would be the primary doctor. Here they keep coming until the whole hospital comes and sees her.

Riham: Is there a difference in the waiting time back home?

Participant: I just didn’t like the waiting time here, honestly.

Riham: You are not used to it then.

Participant: No, I am not used to it. In our country, you don’t wait longer than ten minutes.

Riham: That’s it?

Participant: Yeah.

Riham: This is at the hospital?

Participant: Yes, at the hospital. If it is a fever, they quickly see the patient and send them on their way. Here they do not let you leave till they examine the patient completely. From their head to their toes.

(2B.1 -- 5:43)

Riham: They do not examine the patients the same way there [participant’s homeland]?

Participant: No, they do not. They are not as precise as they are here.

Riham: How is that? Tell me what you mean.

Participant: Here, they worry more about the child. In our country, they don’t do that.

Riham: Does the doctor just look at her from afar and tell you that there is nothing wrong with her?

Participant: No, he will examine her but then he will write me a prescription and send us on our way.

Riham: Did they give you a prescription here?

Participant: Yeah, they prescribe a pain reliever. They gave me two, honestly.

Ahmad: Did they have an interpreter?

Participant: Yes, they did. There was one already at the hospital.

Ahmad: Oh, okay.

Riham: In person then, not on the phone?

Participant: Yeah, in person.

Riham: Sometimes they do it over the phone.

Participant: Yeah, sometimes at the clinic they would call them over the phone. That other day that we went, there wasn’t one available in person so they called an interpreter for us.

Riham: Do you find one easier than the other?

Participant: Yeah, I felt like the one in person is easier to understand than the one over the phone.

Riham: Of course. The body language also plays a role.

Participant: Yes.

Riham: Let me see. You took your daughter, right?

Participant: Yes.

Riham: What are some things that made you want to take her in?

Participant: She had a really high fever. She could barely breathe. I felt like she really needed to go to the hospital. I didn’t have any medicine at home. She was also very congested and had a difficult time breathing. They noticed that in the hospital as well. They told me that she is not able to breathe easily. They gave her medicine and she immediately got better, thank God.

Riham: Thank God. So trouble breathing and the high fever were the things that caused you to take her to the hospital.

Participant: Yes.

(2B.2)

Riham: Continuing with 02B.

Participant: I didn’t like the weather here, though. One day it’s cold and the next day it’s hot.

Riham: Yeah. It seems like this whole year has been like that.

Ahmad: I was just saying that this whole year has been like that.

Riham: Two weeks ago it was really really hot. I had to turn the A.C on.

Participant: We also turned the A.C. on.

Riham: Now, it is really cold.

Participant: Yeah.

Ahmad: Maybe it’s just this weekend. It is supposed to get better during the week.

Riham: I don’t know honestly. Many people are coming to the hospital with a cough and congestion.

Ahmad: It should get warmer this week.

Riham: What is it going to be?

Ahmad: In the 70’s.

Riham: That is really nice.

Ahmad: Yeah, it is warmer but still doesn’t feel like Summer.

Riham: Yeah. Your kids are still too young to go to school, right?

Participant: My daughter goes to school now. She is turning six.

Riham: Your son didn’t start though.

Participant: No, he didn't’ but he will.

Riham: He will start the upcoming school year then.

Participant: That’s right.

Riham: Do you put him in school during the summer or only during the fall?

Participant: I wish there was a 24 hour school! [laughs]

Riham: That means you are giving your mom some trouble. May God protect them.

Participant: Thank you. I put my daughter in daycare too.

Riham: Currently?

Participant: I got some cards [ads or brochures] about a daycare. They didn’t accept them there because they were not vaccinated. I tried to put them in from the start but it didn’t work.

Riham: Okay. What will you do with your time when they start going?

Participant: I will start going to the organization for the English lessons.

Riham: That is really nice.

Participant: That way if I would like to work, I can. I can’t work till I learn the language.

Riham: Yeah, you are still young so you will learn quickly.

Participant: We are going every day. I am picking up a lot of the language.

Riham: Where are you going every day?

Participant: To the Catholic Charity organization.

Riham: They teach you every day?

Participant: Yeah, every day. It is from 9 to 1:30.

Riham: That is really good. Do they offer English lessons?

Participant: No, we have an English lesson for an hour then we have a lesson on practical living. In that lesson, they teach us about health, life here, and how to solve any problems we face.

Riham: That is really good. Are you finding the medical information they are giving you helpful?

Participant: It is really helpful. I benefited a lot from it.

Riham: What are some things they inform you about in terms of health?

Participant: They are telling us that if we need to go to the doctors office, we need to make an appointment beforehand. We have to call ahead of time to make the appointment. They told us that when we have an appointment, that we should go on time. We can’t be five minutes late or half an hour late. Things like that.

Riham: It is different in our culture. For us, it's rude to come on time (said jokingly).

(2B.2 -- 3:00)

Participant: Yes. Here people are more punctual. They also treat their patients well. The doctors treat us well. That is what they teach us.

Riham: Do they teach you specifically about children. What each age group needs, for example? Or is it just general information?

Participant: You mean regarding school?

Riham: No, here the children's medical system differs slightly from what they do in Syria. Children go to the doctors at certain ages like at 6 months, 9 months, and so on. There's a bit of an organization to it.

Participant: No, we don’t have that. I don’t always attend that second lesson. I mostly go to the English one. They also taught us how to use the medication here. How much medicine to give for each child. And to check the expiration date, for example.

Riham: That is really good then. It strange though, because there is a specific way to measure children’s medicine. It is based on the weight of the child. How are you supposed to know?

(2B.2 -- 4:19)

Participant: Yeah, they told us about the weight. I think this was regarding how to measure a fever.

Riham: Oh, as in how to measure it? Okay, I get what you are saying. That is really good.

Participant: It is good. If a person sets their mind to it, they will learn. I am trying.

Riham: You have young children though. It can be hard. May God protect them for you.

Participant: Thank you.

Riham: Is there anything else you would like to learn more about regarding health?

Participant: I am able to manage thank God. I know pretty much what I need to.

Riham: If you have any medical questions, how do you get them answered?

Participant: If I am at the doctor's office, then I ask the doctor.

Riham: Let's say you don't have an appointment.

Participant: What question will I need to ask? I don’t really have any questions.

Riham: For example, your son is vomiting a lot.

Participant: I’ll go online. I’ll call and ask my mom because she has more experience than me.

(2B.3 -- 5:34)

Riham: Okay, so you ask your mom.

Participant: Yes, I ask my mom.

Riham: What website do you use when you search online for answers?

Participant: I use Google.

Riham: Google.

Participant: Or I go on youtube.

Riham: You find stuff on youtube?

Participant: I’ll search whatever it has to get more information. If I can’t find anything, then I use cumin.

Riham: Cumin can really help. What else do you use besides cumin?

Participant: I use some plants and herbs. I use mint. If a child has a cold then all these things help.

Riham: Are there any other things? Tell me all of them. I am learning here. You said cumin, herbs, mint, and what else?

Participant: Honey helps a lot with a cold. My mom taught me to use honey with hot water and a squeeze of lemon for a cold. If a child is coughing it will help immediately.

Riham: Okay, do you give it to the child once or twice?

Participant: Once in the morning and once in the evening.

Riham: Okay, do you use these things daily or just when a child has a cough, for example?

Participant: Only when they have a cough.

Riham: That is really great.

Participant: They eat honey every day though because it has many benefits. If they are in pain though or has a cough then you would add honey to some hot water.

Riham: Okay, but every day they have --.

Participant: Honey. Every day they eat honey.

Riham: How does the honey help?

Participant: It is really good for the body. It has millions of benefit. It is good for the body, for the mind, the memory, and everything.

Riham: Yeah, of course, we know that, but some people do not eat honey.

Participant: Yeah, I know people that do not eat honey. I personally don’t eat extra honey but I do give it to my children. Against their will [laughs]. It is good for them and strengthens their immune system.

Riham: That is true.

Participant: If the weather changes, they do not get sick immediately. They would have a strong immune system. They won’t catch a cold immediately.

Riham: What are some other things that you believe strengthen the immune system?

Participant: I know that honey does. Food in general. Olive oil.

Riham: Is there anything else?

Participant: This is all I know.

Riham: Okay so food and herbal tea.

Participant: Yes, herbs. Sometimes tea can be harmful. It absorbs the milk from the body. Tea is not good for little kids. However, if you get them in the habit of drinking flowers in their tea, that is better for them.

Riham: You would boil the milk and then add some flowers to it?

Participant: Yes, with milk. Or with milk and honey as well.

Riham: Are you finding it difficult to find these things here? The herbs that you are used to using, for example. The cumin.

Participant: No. It’s the opposite actually. It is more available here.

Riham: Thank God.

Participant: There are also a lot of Arab markets. It's true that they are more expensive than other markets, but they are still available.

Riham: Yeah. Do you go to the one in West Chester?

Participant: Yes, the one in West Chester.

Riham: Yeah that one is clean and nice.

Participant: Yeah but it is expensive. There is also Jerusalem [market].

Riham: I do not know that one.

Participant: I don’t know the English name of it.

Riham: Is it near you or is it far?

Participant: No, it is on the other side of Halal Market. We do not have any Arab stores here.

Riham: I just know the one that is next to the mosque that is far from here.

Participant: Yeah, it is not that far. It is about 14 or 15 minutes from here.

Riham: Only?

Participant: Yeah, it is not far.

Riham: Maybe because you live far away from where I live. I live more south from here. It took me half an hour to get here, I think. Maybe it's closer to you then.

Participant: Yeah, when we go to the mosque it just takes less than 20 minutes.

Riham: Do you go to the West Chester mosque or Clifton?

Participant: No, we go to the West Chester. Where the Rahma organization is.

Riham: Yeah, that is the one. The big one.

Participant: Yes, the big one.

(2B.2 -- 10:22)

Participant: Do you know the small mosque? We’re going to move next to it now.

Riham: You’re going to move there?

Participant: Yeah.

Riham: When?

Participant: Maybe this week.

Riham: You will be my neighbor. Why are you moving?

Participant: What is the name of the area?

Riham: Clifton.

Participant: I don’t recall the name, honestly. Maybe it’s Mason or something like that.

Riham: It’s Clifton, I think.

Participant: Maybe, I don’t know.

Riham: In Cincinnati, right?

Participant: Yeah, in Cincinnati. You were just talking about West Chester. It’s on the other side of it. It’s really close to it.

Riham: Honestly, I do not know. Oh, maybe it’s Mason.

Participant: Yes, that is it.

Riham: Oh, you are moving next to Mason mosque, then.

Participant: Yeah.

Riham: That is far.

Participant: There isn’t much of a distance between that mosque and this mosque.

Riham: The West Chester one and the Mason one. You’re right. I am more south.

Participant: You are in a different area?

Riham: Yeah. Since we are students --.

Ahmad: We are close to the University.

Riham: Yeah, the mosque is next to the University. It is close to us. I have never been to the Mason mosque but West Chester is really nice.

Participant: Yeah it is. That is where I go to learn English.

Riham: Oh, I thought the English lessons were at Clifton.

Participant: No.

Riham: No, okay. When you go to these lessons, do you find them beneficial?

Participant: Yeah, when the teacher teaches us, we understand well. He knows Arabic and English. If we don't understand something in English, then he writes it in Arabic. He makes it easy for us to comprehend the language. He is a good teacher and we are learning a lot.

Riham: Thank God. Are you finding any problems with transportation?

Ahmad: What transportation do you use to get to the mosque or market?

Participant: My husband has a car.

Ahmad: Oh, he has a car?

Participant: He just got his license.

Ahmad: Congratulations.

Riham: Congratulations to you guys.

Participant: Thank you.

Riham: Are you also going to learn how to drive?

Participant: God willing.

Riham: God willing. That is really good.

Participant: His brother, Ahmad, is also learning now. He will also get his license.

Riham: That is really good. Otherwise, it is difficult to get around here.

Participant: Yeah, it helps a lot. The community also helps a lot with this matter.

Riham: Okay, let's say you want to go get groceries. What do you do?

Participant: My husband takes me now. Before we he got the car, they [community members] would help. They would come and take us.

Riham: How would you ask them? Was it by phone or text?

Participant: By phone or text. I would write that I needed a few things and if someone can come take me. They would come immediately.

Riham: That is really nice.

Participant: Yeah, thank God.

Riham: Do they specify a time like every Monday, for example? Or is it on a need to need basis?

Participant: No, it is not a specific time. Whenever they are free because I do not have anything.

Riham: Okay. That is really good, thank God.

Participant: Yeah, thank God. They really help with these things.

Riham: You two were speaking a little about the community and I interrupted. How are you finding the community?

Participant: They are really good. The Arab community here is really good. They are better than this organization that is working with us. They do not leave us on our own. If there is anything we need, they immediately respond and help. They help a lot, thank God.

(2B.2 -- 13:44)

Riham: What are some things that they do that differs from the other organization?

Participant: A lot of things because the organization doesn’t really help with anything.

Riham: Like what?

Participant: When we first arrived, they took us to see our doctors but now they are saying they are not responsible and that we should go alone…. We had to go by bus.

Riham: Did you try to go by bus?

Participant: Yes, I did. One time I came back alone from the Children’s Hospital by bus.

Riham: Were you able to figure out how to use them?

Participant: Yeah, if I get on the bus number 45 I think, it drops me off right in front of the house.

Riham: Okay. Was it easy or hard?

Participant: I didn't know how to use the busses at first, but this one woman helped me. There was a bus near the Children's Hospital but it was quite a walk. A woman shows me the way. I understood what she was saying and I found the bus stop. When the bus came, I got on. It dropped me off right next to the house. I didn’t get lost.

Riham: Okay, then you were able to understand how to use them well because its hard to use the bus system.

Participant: There were some other busses that take you to the downtown area, and then you have to take another bus. The bus from the hospital was very easy though. Maybe going there is more difficult but that one time that I took it back home it was easy. I don’t know. Usually my husband comes to pick me up.

Riham: If your kids were to get sick and your husband is at home and unable to take you, what would you do?

Participant: I would call an ambulance. If they were in a critical condition I would call. If it was a matter of a fever or a cold, they wouldn't come and will tell you to treat the child at home. If it is something urgent, then I would call the ambulance.

(2B.2 -- 15:14)

Riham: What are some things that you consider to be urgent?

Participant: If God forbid, a child breaks his arm or is wounded. If they were to have a really high fever then that is something that needs an ambulance as well. Just like that. Those things are scary and need an ambulance.

Riham: What do you consider to be a high fever?

Participant: They told us 110, I think.

Riham: No, a child would have died at 110.

Participant: One time my daughter had a fever of 104.

Riham: yes, 104 is really high.

Participant: Yes, it was 104 and I took her in. They called us in immediately. What is considered a high fever?

Riham: We consider a fever to be anything over 100.4F. 104 is really high. 100.4 is what is considered a fever.

Participant: Okay, I understand now. That is normal then?

Riham: Normal would be 98.

Ahmad: If it is 100.4 you should treat it.

Participant: My daughter was 104. That is why I took her to the emergency room immediately.

Riham: That is when you took her to the emergency room.

Participant: Yeah, I took her immediately.

Riham: How do you measure her fever?

Participant: I place my hand on her head and see how it feels. If it feels like she has a high fever, I use a cold compress. That usually brings down the fever right away.

Riham: You find that it helps?

Participant: Yeah, it helps a lot.

Riham: That is really good. Did you learn these things from when you were a child?

Participant: Yeah, I already knew it. My mom tells me about these things as well.

Riham: That is really good. Parents know a lot.

Participant: Yeah, they do.

Ahmad: When I was talking to you and Mohamad earlier, you told me how they would give you appointments but the organization was not informing you about these appointments. Can you elaborate a little for Riham?

(2B.2 -- 17:40)

Participant: Yeah. These appointments were not for me, honestly speaking. The appointment was for our neighbor. They would tell the caseworker, but the caseworker would not tell us. Our caseworker wasn’t in the office at the time. They told them that my appointment was canceled, and I should not go to the clinic. We didn’t know because the organization didn’t tell us. We went to the clinic and found out our appointment was canceled. We found an interpreter there that came to interpret for our neighbor. She asked if we knew him. We said, “yes, he is our neighbor”. She said that he had an appointment now. We called him and told him that he has an appointment and if he knew about it. He said he didn’t. No one told him that he had an appointment. We asked him if he could come now and he said that he couldn’t. There is negligence on the part of the organization. Our caseworker is currently studying at the University. She is also responsible for more than one family. She is not keeping up. She would see the Syrians here and helps them and then goes to the University. You never find her in the office. She is either here or at the University. She doesn’t have time.

Riham: This is the caseworker, right? The one from Catholic Charities.

Participant: Yes. She is the employee that is responsible for us. They have several employees and each one is responsible for some families. Our caseworker’s name is Stephanie. Our neighbor had an appointment and he didn’t even know. She didn’t tell him.

Riham: That is tough. They don’t communicate with you by phone to tell you when you have an appointment?

Participant: Do you mean the clinic?

Riham: Yeah.

Participant: They do, but I do not know English. They don't provide you with an interpreter. It happened to us the other day as well. They called us speaking in English and my husband said, "No speaking English", and he hangs up. They called again, and again he says, "No speaking English", and he hangs up. They should either leave a message in Arabic, or have an interpreter call.

Riham: Of course. How are you supposed to understand them?

Participant: That is why they call the organization because they speak English there. There is negligence there though. There is a level of negligence. A while ago, I had an appointment with for my children and she didn't tell me. In fact, I was the one that asked her. I always ask her if there were any appointments for me or the kids. The other day she told me that yes, we had an appointment for vaccination. This was May 3rd. It was just a few days ago. I always ask her. She tells me that she forgets. I called and reminded her. I asked her if it was at 10:00 and she said no it was at 10:15.

Riham: It seems like you are an organized person.

Participant: Yeah, I pay attention to these things. I can tell she is careless. I have not been to a doctor yet, though.

Riham: You have not been to see a doctor till now?

Participant: Till now my husband and I have not been. My children went only because of the schools. We talked to them about this issue numerous times. My neighbor always talks to the person that is responsible at the Rahma organization. They have been here for 5 months now and have still not been seen by a doctor.

Riham: To my knowledge, it was supposed to be during the first 30 days.

Participant: That is right. It is supposed to be that way.

Riham: That is the system here.

Participant: My brother-in-law went. Ahmad went with his whole family for appointments. My kids have been because we kept stressing the school issue. My husband and I still haven't been, though.

Riham: Till now.

Participant: Till now.

Riham: Do you have an appointment?

Participant: No. There were several families that came after we came and they have all been.

Riham: That is really strange. I wonder why that is.

Participant: I don’t know. When we were at the clinic we spoke about it.

Riham: Maybe you missed the appointment or something?

Participant: Honestly, I don’t know. I have hearing problems and want to treat it. I had my hopes up that when I got here, I would get it treated immediately.

Riham: What is it that you have?

Participant: The decrease in my hearing.

Riham: Oh, that is right.

Participant: They should treat it immediately. They still didn’t tell me when. I had hopes I would get treatment here in the U.S.

Riham: Yeah, that is not good at all. For refugees, they must see a doctor during the first 30 days of arriving.

Participant: Yeah, that is how it is supposed to be. They have to give us shots immediately. They need to give the vaccines and [indistinct]. We always bring it up to them but we have not gone yet.

Riham: You should continue to be insistent and follow up with them

Participant: I always tell them. They keep saying that it is not from their part. Then why can’t she follow up with them?

Riham: It seems like you are dealing with a few problems. These are what I consider to be problems. It seems like it is hard to find a doctor firstly, and secondly, it's hard to even get an appointment with a doctor. Right?

Participant: Yes. Definitely.

(2B.2 -- 22:40)

Riham: You were saying that now your husband has a car, thankfully, but before that the transportation was difficult.

Participant: Yes, the transportation was difficult. Even now my husband is at work and will need to take off a whole day of work just to take me if I had an appointment. The other day I went to the clinic. The caseworker took me but she was not able to bring me back home. My husband had to leave work to come bring me back home.

Riham: That is really good. These things you are dealing with can really cause anxiety at times.

Participant: I told you that I returned by bus that day, but I was scared the whole time. Scared. It is true that I came back alone but I was afraid the whole time. Very afraid.

Riham: It was nighttime as well, right?

Participant: No, it was during the day to be honest. It was around 1 pm. I would never go back alone at night. It was during the day, and my husband wasn't able to leave work to take me back home. It was hard for him to leave work. Thankfully I came back easily and didn't get lost.

Riham: That is good that you learned how to get back on your own.

Participant: I had my kids with me as well. They needed some chest scans done at the hospital.

Riham: What were they for?

Participant: They said that instead of giving them the shot, doing a scan was much easier. I don’t know honestly.

Riham: Yeah, I don't know either, honestly. Oh, maybe it was because of tuberculosis. What is it called in Arabic? Sorry, I do not know what it’s called. It is a type of infection in the lungs.

Participant: Yeah, exactly.

Riham: They either do a shot and check it in two days, or they do an x-ray of the chest to see evidence of the infection.

Participant: Yeah they call it “sil”.

Riham: It’s called “sil” or “still”?

Participant: They take your arm and prick it a little. If it swells that means you have the disease. If nothing happens, then you do not have the disease.

Riham: Right.

Ahmad: Yeah, it is called “sil” [in Arabic}.

Riham: Yeah, because research shows that many refugees have this disease. They try to check for it so that they can treat it quickly.

Participant: Do you mean the people that are coming from Syria?

Riham: No, not just Syrians. I mean any refugees in the world.

Participant: Really? I don't know. In Jordan, they would frequently vaccinate the children. Almost every month they would get a vaccine. Thank God, none of us have anything.

Riham: Thank God. Did they test you then?

Participant: No, here I have not been to the doctor at all! Remember? No one checked us for anything.

Riham: Let me see what other questions we have. We were talking about the home remedies you use when your children get ill. Let’s say they are fine and healthy, are there any things you do to prevent illnesses?

Participant: The first thing is cleanliness.

Riham: Tell me what that means to you.

Participant: I always shower and clean them. I clean the house as well. If it wasn’t clean they could get ill immediately. The first and foremost thing is cleanliness.

Riham: Okay. What are some other things you do at home?

Participant: That is it.

Riham: Just cleanliness?

Participant: Yeah, that is all in my opinion.

Riham: It’s a good opinion. You mentioned that you give them some things to drink. Do you feed them anything in particular as well?

Participant: They eat what we eat.

Riham: Basically, whatever healthy food you choose for yourself, you give it to your kids as well.

Participant: Yes.

Riham: Okay, good. Do you take them to any gym or to play outside?

Participant: Yes, we go out and play and we have a park close by that I take them to.

Riham: Oh, nice. You have a park next to your house.

Participant: Yes, we have a park right next to our house.

Riham: That is really nice. They run and play.

Participant: Yes, they run and play. If a child stays locked up in a house, they would find a way to release their energy. They might fight with their sibling. If they do not go out to play, they have nowhere to place their energy and they will get depressed. We have to give them their freedom and relaxation.

(2B.2 -- 27:47)

Riham: Let’s say the weather does not permit to go out, are there things you can do at home to calm them down?

Participant: I keep them entertained. I play with this one and then that one. I might put them to sleep as well.

Riham: That is nice.

Participant: Or I might make them a food that they like. Sometimes I turn on the television for them.

Riham: That is really nice. Do you read stories to them or anything?

Participant: I do not like reading.

Riham: Some people have tolerance for it and others don’t.

Participant: Yeah, and all the stories here are in English.

Riham: Oh, you do not have any in Arabic?

Participant: No, I do not. I tell them the story “Leila and the Wolf”. They know that one by heart.

Riham: That is really nice. Other than your in-laws here, do they have other children that they play with?

Participant: We have our neighbor. Her kids are a little older, but they play with them a lot.

Riham: Are you finding this environment good for your peace of mind?

Participant: Yeah, I am really at peace here.

Riham: It got better?

Participant: Yes, thank God.

Riham: Thank God.

Participant: It is not just the Arabs here, but even the westerners are kind. We thought they would be cold and frowning all the time. They all greet us constantly. When I see them they would say “assalamu alaykum” [Muslim greeting] and start laughing.

Riham: That is really nice.

(2B.2 -- 29:20)

Participant: They started to know us. When they see us with a scarf on our heads, they say, “assalamu alaykum”.

Riham: That is really nice. Is this just in your neighborhood, or everywhere?

Participant: No, everywhere. When people see me they say, “assalamu alaykum”, or “hi”. My kids say hi back. They learned it. So far I am enjoying life here.

Ahmad: I pray it gets better and better.

Riham: God willing. Are there things you are doing to help your psychological wellbeing? I know you said you are doing well but we all have those difficult moments in life. Are there things that you do that help you?

Participant: I speak to my mom. My mom is the first person that helps me if I am upset.

Riham: How long has it been since you last saw her?

Participant: It has been five years. I left Syria in 2013.

Riham: You left immediately, then.

Participant: Yes, we left immediately. We did not stay there during the war.

Riham: Did you go to Jordan first?

Participant: Yes, we went to Jordan first.

Riham: Okay. You spent that whole time without seeing your mom.

Participant: I saw here on the phone.

Riham: Oh, that is really nice. By video, right?

Participant: Yes, by video. Thank God they are okay.

Participant: She is staying in a safe area in Egypt. She is not in Syria or else I would fear for them and not know if they are okay or not or if they ate. Thank God, I am getting updates from them.

Riham: That is good, thank God.

Participant: I hope we can get our residence permit so we can go visit them.

Riham: You have to wait for a year?

Participant: Yes, we have to.

Riham: Okay, before you are able to leave, you mean.

Participant: Yeah, we can not leave without getting the Green Card. Unless Trump changes things.

Riham: Yeah, be careful. Honestly, this isn’t my area of expertise so I do not know the laws or how much you are supposed to stay here and what not.

Participant: We are slowly learning about the laws.

Riham: Ahmad had a really good question that he asked earlier. The one about expectations.

Ahmad: Oh, right. Yes, we have been hearing from other refugees that before coming here to America, that they are told certain things but when they come here, their expectations are not met. Did something like that happen to you?

Participant: It happened to my husband and my husband's side of the family. When we were in Jordan, they said that we would be able to bring our family with us. His family members have a lot of health issues. My father in law has a neurological problem and can not speak. I have my brother in law is also disabled. His mother has diabetes. They only have one young member that is able to help. Now they are telling him that it is hard to bring his family.

Riham: Is there anyone that you can turn to when you have these questions or problems?

(2B.2 -- 33:13)

Participant: I tell our caseworker because she also knows Arabic.

Riham: Stephanie?

Participant: Yes, she speaks Arabic.

Riham: Oh, she can speak Arabic. That is really good.

Participant: Yeah, she helps us a lot. If anything comes in English, she translates it for us.

Riham: How long does she help you for?

Participant: She constantly helps us.

Riham: No, I mean for a month or two months?

Participant: No, she will keep helping us.

Riham: Is it for a year or two?

Participant: No, it just all the time. Anything we need, she will help us with it. We do not know anything about this country and they are responsible for us. The Arab community here helps us as well.

Riham: That is really good.

Participant: Yeah it is.

Riham: Others have felt like there was no one to turn to for help. I was wondering if you felt the same way.

Participant: No, we are getting help from our caseworker and the Arab community here as well. If we had any question, the Arab community answers it for us. They help even more.

Riham: That is really nice.

Participant: Yeah, thank God.

Riham: When you first arrived here in Cincinnati, what were the major health needs you had? Like the insurance or other big needs you felt like you had.

Participant: I didn’t really understand the question. Do you mean the most thing that helped me?

Ahmad: It is talking about the major health needs you had. I know you mentioned that your children did not have any illnesses. You and your husband also don’t really have any illnesses. Are there any health problems that you faced when you came here to Cincinnati? Maybe issues with the insurance, for example.

(2B.2 -- 35:44)

Participant: No, we didn’t. Everything was processed quickly and we received health insurance immediately. We did not face any problems of this sort.

Ahmad: When you first arrived, what were the most important things for you to gain from here in terms of your health needs?

Participant: I don’t know.

Riham: The house or the air conditioning are some examples. What were the most important things?

Participant: All those things were provided for us. Anything we needed, we received immediately. There was nothing that we needed and we didn’t get, thank God. We came to this house as you can see. Everything was provided. As soon as we arrived, they brought us food. They even gave us money in case we needed anything. We got our insurance right away and IDs. We received everything right away and weren’t missing anything, thank God. Everything was provided.

Riham: That is really good.

Ahmad: Yeah. Did you have any other experiences that we did not cover and you would like to share? Is there anything that happened to you that we did not mention?

Participant: No.

Ahmad: No. Okay, how did you find the treatment of the doctors and nurses here?

Participant: I really liked their treatment. They respect their patients and treat them well. They are really good.

Riham: Thank you so much for sharing your thoughts and opening your home to us.

Participant: We enjoyed our time with you.

Riham: We did as well. Okay, I will now turn the recorder off. Thank you so much.

Interview 3A

Time (3A.I) – 0:00

Ahmed: I started. I am Ahmed Beydoun. Today is May 8^th^, 2017. I am with participant 03A, and we're going to start right now. So, we're going to start right now as if we didn't speak before and—.

Participant: Yup, go ahead.

Riham: Okay. And I, Riham, am also here at this gathering.

Ahmed: So, let's start. Tell us about your experience with the health system here in Cincinnati just to start with, and we'll get into the details later.

Participant: As a health system, thank God, things are good.

Ahmed: Things are good? What are the ways in which you interacted with the health system? For example, are you going to doctor's appointments? Did you have to go to the hospital?

Participant: Of course. Appointments—. I have children getting appointments, and we slept in the hospital. We stayed for about a week in the children's hospital.

Ahmed: Children's?

Participant: Yea, children's. [Indistinct] Service. Excellent service.

Ahmed: Excellent? Were there any big problems? Any problems you faced? Any difficulties?

Participant: The difficulties—. The hospital is equipped to handle such matters—. [Indistinct] was immediately taken care of. I mean an interpreter. If there's an interpreter, everything can be solved.

Ahmed: Are you finding the interpreter—. [Indistinct] you mentioned that in the clinic you go to, there's an interpreter there anyways, right?

Participant: Yea.

Ahmed: Even in the hospital, for example, when you went and spent the night there, was there an interpreter?

Participant: For sure.

Ahmed: Did you find any problems with the interpreter or was it something simple?

Participant: No, some interpreters—. The language is a little different. We're not able to correctly understand each other.

Riham: Like they don't speak the Syrian dialect? Or they don't speak—?

Participant: No they speak, for example, like someone who speaks Algerian, Moroccan, or Libyan.

Riham: Yea, honestly, that's difficult!

Participant: It's happening sometimes that understanding each other—. Like, I've interacted with them before. I'm able to understand them, but when my wife goes she's not able to understand anything they're saying. Like, she finds difficulty in the translation. He speaks to her, for example, in the Libyan dialect. I mean, it's still Arabic, but the dialect is different.

Riham: Yea, anyways they add French words to the Arabic.

Participant: Yea, exactly. Their mother tongue is mixed up anyways. So that's the thing where there's a little bit of confusion.

Riham: When you guys feel that or when there's someone whom you can't understand easily, do you try to tell them—

Participant: Of course!

Riham: we want someone Syrian or Lebanese or Jordanian—?

Participant: No, we can't impose conditions on them like “We want an interpreter from [Indistinct].” We, for example, make an appointment. We go, and there's an interpreter there.

Riham: Okay, he's already there.

Participant: Already there. For example, to tell them, “I don't want this interpreter.” I mean, this interpreter already came. “You want a different interpreter? There's no other interpreter.”

Riham: I understand.

Participant: They request the interpreter because “He has an appointment for interpretation.” He comes and meets you, finishes, and has other appointments, for example. If, for example, I'm understanding him, and he doesn't understand me. Now, it happened with my wife where she told him, “I'm not able to understand him.” She was with him on phone and said, “I'm not understanding anything.”

Riham: Yea, and then what did they do?

Participant: They switched him.

Riham: Oh, okay.

Participant: I mean, they switch him. If he's on the phone, he can shut it and—what's it called, but if the translator came and is there, no one can say, “You're not understanding me. Go, God be with you!” A person is a little bit—[Indistinct]—like—eh.

Riham: Yea, okay. You said your daughter was in the hospital. You spent a week there?

Participant: Yea, that's right.

Riham: That's when I met you, right?

Participant: Yea, when we saw each other.

Riham: Last summer?

Participant: I think it was at the beginning of August.

Riham: Yea.

Participant: The beginning of August.

Riham: So, it's been about 10 months since then.

Participant: Yea, about 10 months.

Riham: Yea, so tell me about the first time you went. Tell me a little bit about what happened according to your perspective. How did you feel in the ER and then upstairs. Tell me a little bit about how you felt.

Participant: To be honest, I felt that they have concern, and they monitor that this patient came in. My daughter's state—. I used to explain to them that “My daughter's situation is such and such. She used to be in Turkey. She had a bone marrow disease, and she needs surgery. That's the reason why I brought her to the US. Our selection came when—through the UN to New York. When we arrived at New York, they put us in a hotel for a night because of your daughter's circumstances. They transferred us to Cincinnati Children's Hospital.

Riham: So you thought you were going to stay in New York?

Participant: That's what they told me when I was in Turkey. “You are first going to the State of New York.” [Indistinct] When we got there, they said, “No,”—what's it called—“because you have two sick children, Cincinnati Hospital is—”

Riham: [Indistinct]

Participant: “better prepared to take on these things.”

Riham: How did you feel when you thought you were going to stay in New York then went to a totally different city?

Participant: Of course, in the beginning it was like, “Why did you change us?” They said, “It's for your kids.” Anyways, the whole reason I left was completely for the kids. I mean for their medical treatment. So we came and suffered a bit with the—what's it called—the organization that's here with regards to the housing and whatnot.

Riham: Tell me a little bit about it. I remember a little but for the recording. Tell me when you first arrived—.

Participant: They put us—. When we arrived, they put us in a place [either house or apartment; most probably apartment]—. Of course, when we first came, news of our arrival for sure reached them that “The reason he came is because he has sick children.” So they put me in a place that was in a bad neighborhood. I mean, in terms of the neighbors, there were problems. They come and knock on the door wanting food, wanting something to drink. They're always outside getting high and whatnot. The place was completely full of bugs, and it was a dirty place. When I first entered it after they first brought me from the airport, I held my nose and said, “What's this? Where did you bring me?” They said, “Stay.” I said, “I'm not going inside. I'm not staying here.” They said, “Go inside. We'll switch it for another place for you.” I said, “Fine.” I put the stuff down and didn't sleep. Our luggage stayed as I left it. They kept delaying the issue by always telling us us they would take care of it the next day. Then the girl—. I mean the house's smell—. The kids—. [Participant's daughter] got sick [Indistinct] because of it. She went back and relapsed. We stayed in the hospital for a week because of it. And I said in the hospital—. I said in the hospital that “Such and such is the situation.” We all got [the flu/a cold?]. We started scratching. I mean, we went to the doctor. They wrote us prescriptions for ointments. Bugs! Inside them—. Bedbugs—what do they call them—bedbugs. All the carpet on the floor was old. Pardon me for saying this [literally “May God honor you”], but the bathroom was all leaking to the kitchen. Flies were coming up. I mean, it wasn't something normal.

Riham: Nope.

Participant: So the—what's his name—that guy came, the official [one responsible] for the environment when I spoke in the hospital. He came, saw the situation, and wrote 10 reports about the matter.

Riham: Yea, good.

Participant: The basement had a [Indistinct] that's completely full of bugs. I mean the same—. When it would rain, it would come inside and flood from underneath. I mean, it was an uninhabitable place. So, they took us out of it with great difficulty. They brought us here to this place.

Riham: How long did you spend in that place?

Participant: We spent about 25 days in it.

Riham: Twenty-five days?!

Participant: Yea, but of course, all of it was filled with suffering and whatnot until we got out. So, of course, with great difficulty they—. They had a three-month lease with the landlord. I told them, “It's not my fault. You want me to stay in a house that you immediately sign a contract for?” Of course, that's also from my [Indistinct] that's in—what's it called. They took at that time—. They wrote me that the place's rent is $2,800.

Riham: $2,800?!

Participant: They wouldn't return the deposit. I told them, “It's not my fault.” At most, I'll give him his place's rent and pay for water and electricity so that it's doesn't become a matter of “halal or haram” [so that I'm sure I'm not doing anything wrong]. But I'm not—. The deposit I—. When I first went in the place I told them, “My problem with you is with regards to the housing.” If I didn't have sick kids—. I mean, the kids got into a bad mental state. They can't go outside. Neighbors that, I mean, aren't normal. At the same time, an uninhabitable place. Not even glass—. I mean, broken glass. It was like an abandoned house, not like a place where you could put—what's it called—inside. So they—. I talked to the organization again and had to go back and forth until in the end they returned $750 of them to me.

Riham: So, basically you lost half of it.

Participant: Yea, they took about $1,300. They took it—. He took it as rent for the—what's it called. I told them, “You call this a place? I didn't sign the contract, and I didn't want to stay in it anyways.” So, they came back and with a lot of difficulty found this here place for us.

Ahmed: How are finding this place?

Participant: To be honest, this place—. We had some problems for a while with, I don't know, maybe people who [Indistinct] when they first [Indistinct]. They would come and knock on the door—kick the door—at night, during the day.

Ahmed: Are these problems still there or—?

Participant: No, after we complained—. We called the police. I wasn't here at home. [Indistinct] there were 10 kids 17/18 years old throwing rocks and sticks at the door, swearing, and whatnot. So my wife called me and said, “There's something. They're breaking the door.” “What am I supposed to do?” The problem is I'm far away. Even if I do come, what am I supposed to do to them? I told her, “That's it. Call the cops.” So, they called the police, but by the time they showed up, the kids had already left. So, the officer came and saw the rocks and sticks—what's it called—

Time (3A.I) – 10:00

the sticks and whatnot. So, he came back after some time and held a—. In the—what's it called—there's an athletic club for football [could be “soccer”] here—. So, he held a meeting for the locals here. Most people in this area are refugees.

Riham: Ah, okay. They put refugees in this area?

Participant: No, they—. There are currently maybe four or five Syrian families here in this area,—

Ahmed: How many?

Participant: in this here city. And what's seems to be the case [Indistinct] is that they're usually close to the organization, and the organization is here.

Riham: Oh, Okay.

Participant: There are some, of course, that are spread out, so they're not necessarily here. It depends on if housing is available here. So, everyone spoke to him about what problems they were having, and many experienced what we had. It's not because you're Syrian or anything. [Indistinct] There are reasons for them banging on doors, swearing, or causing them harm.

Riham: In your opinion, why are they doing that? Why this behavior?

Participant: Honestly, I think it's childish foolishness, not that anyone is doing it on purpose or anything. They're kids, you know? For example, my daughter saw them from here—from the window—and said they go to school with them. They know them, for example, that they're maybe Arabs or something else. So after the police started coming here—. After that, the police started stopping here every day.

Riham: Oh, that's great.

Participant: The officer dealt with the issue really seriously with regards to the park [or the front yard]here. It's a pretty run-down park. You see, for example, I don't take the children to the park. If I'm not with them, I don't let them go out to the park. Even if you go out—

Riham: Why?

Participant: It's all problems, people getting high and whatnot. The police comes every day—. He's going to come in a little bit. He parks a car here and a one down the street.

Ahmed: [Indistinct]

Riham: Do you think these things put a lot of pressure on the kids' psychology [literally “mental state”]? I mean pressure on the kids. Pressure on you, too, but, of course [indistinct]—

Participant: Of course, there's been pressure. Even in school, there's the kids' teacher. They told her, “Such and such. We're getting scared.” They told her, for example, “I'm not even able to go out.” If I have to be out of the house for work or to run an errand if I have something at night, I can't keep my wife home alone. So she setup an alarm system for the—what's it called—the police.

Riham: Good.

Participant: She provided the funds [or “raised the funds”] from herself. It's even $50 a month.

Ahmed: [Indistinct]

Participant: Because she came and saw when [Participant's wife] called her and told her “ Such and such. They're banging on the door. [Participant] is not here. [Participant] is not home. What should I do?” She opened the door, and they rushed toward her, so she closed it. I mean, she gets afraid. She doesn't understand the language, and she doesn't know what to do.

Riham: Of course!

Participant: So she came, and she even came immediately. After that, she set up this here device. “If anything happens, immediately call the cops.” The same officer came by anyways and investigated what happened. After that, the police started to—. Maybe they saw some of their actions. They got scared. They stopped doing these things. They stopped doing them.

Riham: Thank God. Do you think the kids have relaxed a little bit, too?

Participant: Yea, the kids started to get a little better. We had a time when the kids—. If someone, for example, if a guest comes over and knocks on the door, they would get frightened. They would get afraid because they don't know what's going on. They just hear. “Is it a crime?” There's fear. “Is someone going to attack us?” When we were in that old place, they used to knock on the door at 12 AM, 1 AM wanting food. The problem is we don't know English and don't know what to tell them. I became like a guard, staying up late at night. [Indistinct] Like, it's a big place with a forest behind it. They were always sitting out on the street. It was like they were blocking the street, sitting down on the street, drinking, yelling at each other, and the police would come. It—

Ahmed: wasn't a good area.

Participant: wasn't a good area. At the same time, you have someone that's new to the country and doesn't the country. How are the people? How's the world? So, thank God.

Riham: You said you put the alarm system to help your kids feel a little bit more at ease?

Participant: Of course, if, God forbid, something bad is going to happen, this device isn't going to prevent anything from happening, but at the same time, for one's psychology, it provides peace of mind. Maybe the robber or the person coming—. That person is going to consider that it might be there, maybe also as a mental state of mind. For example, maybe he's going to consider it might be there. [Indistinct] As a person, he's taking precautionary measures.

Riham: Are there other things that you or your wife tried to do for them to relax more, mentally? Because what I hear from what you're saying is that your daughter not only has a physical sickness but also this state—

Participant: Of course, one's psychology—. For children, you'd say they were adults, [Indistinct] I don't know. I mean, I have this child here, [Participant's son #1], when I brought him also to Turkey, he also had Wilson's disease, so here—

Riham: Sorry, what disease?

Participant: Wilson's.

Riham: Okay.

Participant: So I told them he has such and such disease—he has Wilson's disease. So I went back and forth to the hospital. We spent about seven months until it was clear to them that he had the disease.

Ahmed: Seven months!

Participant: He stopped taking his medication. The problem is that his chest is sensitive. He has [Not clear. Kahraba atfal? Epilepsy?]. We took him to the clinic when—. For the vaccinations and tests here, and they said he has tuberculosis.

Riham: He has what?

Ahmed: Tuberculosis.

Riham: Okay.

Participant: My son left, and I know him, ever since we left Turkey. The UN did some tests and took x-rays of the whole chest. If a person has tuberculosis, it's clear, and they wouldn't even let them come here anyways. So we went, and he said “No, a specialist in these contagious diseases has to see him.” So, he transferred us to the hospital of—what's it called—

Riham: Children's?

Participant: Children's. They took x-rays and did tests and gave me a report that says he doesn't have any of these diseases. A month later—about a month and a half—another clinic informed me, “Your son, [Participant's son #1], has the tuberculosis disease, and we want to confirm that he has it.” I told me, “Look man, my son doesn't have a disease. Where did you get he has a disease from? What? Every two or three days someone tells me 'Bring your son so we can examine him so we can see what's going on with him?'” He said, “No, if you don't bring him, we're going to have to tell the school to remove him.” We go. Again. I took the papers with me because they said “If anybody else asks to see your son or anything again, show them that we examined him.” I took it to them. They looked at the report, and said “Okay.” Two months later, the same thing happened again. Once more they told us we had to bring him in. I told them, “The kid has a chest allergy and doesn't have tuberculosis. Where did you get he has tuberculosis from? Every few months someone tells me that.” He said, “No way. You have to come in.” I went there and told the doctor, “I have two hospitals that have written down that he doesn't have this disease. Who's the one telling you—who's the one basically making appointments at your clinic?”

Riham: What kind of doctor? His primary care physician?

Participant: It turned out that his primary car physician was the one who was asking, “Examine him.”

Riham: But, it seems that she's not understanding that he's already been examined and—

Participant: I don't know. They're not coordinating with each other. They're not [Indistinct] the reports—

Riham: You mean they're not talking to each other?

Participant: We're not making appointments, you got me? We're not asking for anyone to examine him for his chest or that he has a disease.

Riham: I got you.

Participant: They're telling us “You have to come [Indistinct]—” There's no translator. “My son doesn't have—” “No, he has to come in.” “We're going to have to write a report to the hospital—to the school to remove him.” So I went and said, “It's not like I have nothing to do but to go back and forth to the clinic.. You've left me really worried. The boy doesn't have tuberculosis! We can't believe you've been searching for this thing. If he has tuberculosis then treat him, man!”

Riham: Yea, that's right.

Participant: It's not like I don't have other kids. Even his friends at school, God forbid. This is a disease from God. “If he didn't [does?] have this disease, let him get treated or give him the treatment tuberculosis patients get. Treat him.” He looked at his test results, looked at the x-rays, and said, “We're sorry. Your son doesn't have it. That's it. We're going to close this whole case.” And from then onwards—

Ahmed: [indistinct]

Riham: So this whole story lasted four months? Five months?

Participant: For about five months they left us worried sick. And we were following up on the—what's it called—for his sickness, for Wilson's. In the end, the doctor confirmed that—what's it called—the same one [Participant's daughter] goes to—

Riham: You knew that from Turkey.

Participant: I knew that from Turkey, but they weren't finding it here in the beginning. The doctor was saying he didn't have it.

Riham: Sorry, was [Participant's son #1] born in Turkey?

Participant: No, he was born in Syria.

Riham: In Syria. So you guys found in Turkey? What happened with him that made you find out?

Participant: We didn't know. According to all the tests, [Participant's son #1] didn't have anything. So [Participant's wife]—. He used get the flu/catch a cold in the winter, and we'd take him to the clinic. In Turkey, even in the clinic, they would always do blood tests. The first thing they do is draw blood.

Time (3A.I) – 20:00

Participant: They did a blood test and found that his [liver rater/percentage?] isn't normal. So the doctor in the clinic told her that she has to go consult a hospital. It was in the city of Malatya. There's a special hospital in Turkey for liver transplants and liver [Indistinct]. He said, “Go consult that hospital. They know more than me. There's a specialist doctor there.” So she went—what's it called—[Participant's wife] told me “Such and such happened.” I told her, “We're still not done with [Participant's daughter]. He needs to take x-rays.” He hadn't complained about anything. The boy has normal health and acts normal. He doesn't have anything that's clearly up with him. So she spoke with the doctor that we used to take [Participant's daughter] to. She used to go every two weeks to a doctor. She told her “Such and such happened.” She said, “Why don't you bring him in? Let's take a look at him and do some [regular/systematic?] tests. God willing he doesn't have it.” So [Indistinct] [Participant's wife]. They requested to do a biopsy on him. Maybe “biopsy” is also in English—

Riham: Yea, “biopsy.” It's the same word.

Participant: Yea, so they can see and whatnot—. So they can confirm [Indistinct]. They did the biopsy and said “He has the disease.” Thank God, the Master of all creation. I was signed up before, of course, signed up for [Participant's daughter]'s trip to America. So I started telling the UN that I now have two sick children in these circumstances. At the same, with regards to the pills they were taking in Turkey, Turkey wasn't paying for them. They were coming from Germany. Through the—. Of course, they were coming through the government/public pharmacies, but the state doesn't pay for it. It comes from you. You pay with your own funds. So there was a lot of stress. Now, thank God, here I'm being provided with the medicine.

Riham: Thank God.

Ahmed: Thank God.

Participant: From the pharmacy of the—what's it called. But it's not always there. I go and request it. The next day they give me a part [of the prescription], and two or three days later they give me the rest.

Riham: So both of them are always taking medication?

Participant: They have to take this medicine for the rest of their lives.

Riham: All their lives? Okay.

Participant: So from when they identified [Participant's son #1]'s disease, I spoke with the doctor. I told him, “Doctor, for example, in Turkey”—. I mean, she didn't understand that I wouldn't find better medicine than in America. God, the Master of all creation, the Healer. “Give me something so I know what the kids' future is. I mean, what this medicine is.” They said, “Currently this disease doesn't have a cure. There's no medicine that cures it. You need to continue taking this medicine, the pills he's taking, because they break up the copper substance that is gathering in the liver and [Indistinct]—what's it called. However, its symptoms are a little tough. Maybe he's in a good state now. After a while, what's going to happen. Its symptoms don't affect memory or sight. I said, “With regards to all of this, doctor, we'll do what we have to do.”

Riham: Of course.

Participant: [Indistinct] We're relying on God and doing what we can.

Riham: So, when they're always taking this medication it means you have to go the pharmacy a lot.

Participant: Of course.

Riham: Does it come every six months or three months or do you have to get a prescription every month?

Participant: Every month.

Riham: Okay, do you face any difficulties at the pharmacy?

Participant: Well, I don't know the language, so I have to take [Participant's daughter]. She learned a few words. Yes, so she can explain things to him. And sometimes there will be someone working—what's he called—an Arab. He's Egyptian. He works at the pharmacy.

Riham: At Cincinnati Children's, right?

Participant: Mm-hm.

Riham: Yea, I know him. Anyways, we're not a lot of people.

Participant: [Indistinct] Yea, sometimes he's there. Things work out easily. Sometimes he's not there. For example, if [Participant's daughter]—[Participant's wife] tells me that their out of medicine. Like, there's only a week's worth left [as of today?]. I go there and with hand signals and whatnot I manage to get things done.

Riham: Anyways, you learned Turkish—.

Participant: Turkish? I used to know Turkish from Syria.

Riham: Oh, you learned from Syria.

Participant: Ah. We are originally Turkish.

Riham: Like, my family is also originally Turkish, but they don't know Turkish. My grandma used to speak it, but how did you learn?

Participant: [Participant's wife]'s mother [Indistinct].

Riham: Oh, like they're Turkish Turkish.

Participant: They're Turkish and from on the border. There's only a hill between their house and Turkey. The lands are right next to each other.

Riham: Ah, I understand.

Participant: For my wife's family, their mother tongue spoken at home is Turkish.

Riham: Oh, that's very nice. That means when you went to Turkey it was totally different than when you went to America.

Participant: Of course, when I went to Turkey—when I took [Participant's daughter] to the emergency room, Turkey's hospitals weren't prepared for Syrians. It was at the beginning of the events in Syria.

Riham: Right.

So they didn't have anything other than the [refugee] camps. They were putting people in the camps.

Riham: Did you guys stay in a camp?

Participant: No, we didn't stay in a camp, but the first time I went I just took [Participant's daughter]. She got scared, and her face turned yellow. I took her to many hospitals in—what's it called—in Aleppo. Of course, I didn't go to the government-controlled areas because it was our area, and we had relatives in it that were wanted by the government for—. I went to the countryside areas and the liberated areas. She spent the night in the hospital. They used to put her bags of plasma. I mean, white blood [cells].

Riham: I understand.

Participant: Her face so yellow, she was the color of lemons. With the plasma, everything was alright, but it wasn't widely available. We'd go from place to place and search for it [while willing to pay for it] with money, but it just wasn't available. So one of our friends that lives with us in our area advised me, “Go take her to Turkey.” So we went to Turkey. In Turkey, they weren't prepared to take in a Syrian and provide them with medical treatment. And I used to be able to know how to speak Turkish. I went to Kilis. From Kilis I went to Gaziantep. I talked to the hospital in Gaziantep. “You don't have any ID, but you want to pay out-of-pocket?” “Yes.” “But if it's out-of-pocket, the expenses are astronomical!”

Riham: They don't let you in?

Participant: They don't let you in. So I went to a camp in a county called Adıyaman. I went to the camp and told them I want an ID and whatnot. They said, “No, you're coming for two days then are later going to say 'I'm bringing my kids and—.' It's just a pretense.” I told them “I'm not going to the camp. Anyways, I'm not coming to stay in Turkey.” So, he agreed. He also saw that the girl's health was bad. Her stomach had gathered a lot of water and was puffing out. [Indistinct] So he immediately went in, took our pictures, and we took our IDs to the clinic. The clinic transferred us over to the hospital. The hospital—. You know, there are tests and x-rays and—. So we stayed a week in the hospital. At night the doctor came and told me, “Your daughter's test results came back. She needs a liver transplant. Would do donate?” I said, “I'll do it.” So my family wasn't really in Turkey other than my sister, who lives in another county about seven hours away. So I told them “Such and such happened. [Participant's daughter]'s test results came back, and she needs an urgent surgery.” I told them I'm going—. The ambulance came to take us—. I'm going to donate.” [Indistinct]We arrived at the hospital in the morning. Tests and whatnot. The doctor said “She doesn't need a surgery or whatnot. We're going to do a few tests. We're going to take you up to the wing, and they took us to the—what's it called—wing. So after—what's it called—they started the tests the next day, of course. They made an incision in her back.

Riham: Excuse me, could you please repeat that?

Participant: Tests and x-rays. They requested making an incision.

Riham: Oh, biopsy.

Participant: Not in the liver. An incision from her back.

Riham: Oh, bone marrow! Bone marrow? Aspiration. I understand you.

Ahmed: [Indistinct]

Riham: From the bone?

Participant: Yea, from the bone.

Riham: Yea, bone marrow aspiration.

Participant: They make an incision in her bone.

Riham: So they can do tests on it?

Participant: So they can do tests on that liquid—what's it called. The test results came back. They started requested a lady—. It was the pediatrics department. They wanted a lady to stay with her in the department. I told them “I can't. There's nobody in this whole county that can.” Of course, this talk of tests and whatnot took about a week. They started saying “You have to bring a lady to stay with her.” I called my sister. She came. Right when she arrived, they told her—. My sister, [Participant's sister], doesn't know [or “also knows”] Turkish. They told her “This girl

Time (3A.I) – 30:00

Participant: has—what's it called—cancer in her spinal cord. They transferred her to the department—what's it called—of oncology. They took her to the next department. They said she's not allowed to go without someone accompanying her and a mask. They're all children. They gave her the first dose of chemotherapy. [Participant's daughter] was not—. She wasn't responding at all. They said that's normal in the beginning of the—what's it called. She started. Three or four days the same thing continued. My sister would come out and say, “Your daughter—.” She was talking to the doctor, but he wasn't responding. [or “The doctor used to talk her, and she wouldn't respond.”] “The girl isn't eating or drinking anymore.” She even started to put diapers on her and clean her. So another doctor came. I told him, “Doctor, such and such happened. Her state—. You guys [Indistinct] her. What happened to her” So he took us down, looked at her, did tests—what's his name. “She needs [intensive] care.” They took her to the intensive care unit. They had blood bags, plasma, and whatnot. You know, my sister's staying here, but she also has kids and a family. She left them and came. She said “[Indistinct] The county I'm in”—the state—“has a hospital, too. Would it be okay if they gave her the chemical doses there?” They said, “Yes, but her state right now doesn't allow it. You have to wait a little.” We waited a few days. They said, “Now her state allows it.” We took her, of course, by ambulance. They put her in an ambulance. They sent her. We went there. I told her—. My sister went with [Participant's daughter]. I went down to Syria. [Participant's wife]—. There weren't any telephones. They had [cut?] all the lines. I went down to get her. She didn't even know what had happened to her, to [Participant's daughter]. So, I went down to Aleppo. I stayed a night. I told her, “[Participant's daughter], thank God, is in a good state.” and such things.

Riham: You didn't tell her anything?

Participant: Yea, I took her, and we went out to the countryside.

Riham: Do you mind if I ask you why you didn't tell her?

Participant: Because she thinks that there's nothing wrong with her daughter when she left. If I tell her it was a liver problem, which became cancer, she's going to spend the time wailing and crying and whatnot. We still have a long road ahead of us. I mean, we still have a long road ahead of us to—what's it called—to Turkey. [Indistinct] We're going to be smuggled in.

Riham: Yea, of course.

Participant: And children, too, and luggage and whatnot. So, thank God, the trip worked us for us—what it's called—going up. I took her to—. We got to my sister's house. “Where's [Participant's daughter]?” They said, “[Participant's daughter] is in the hospital.” From here to there, over there we told her, and she started crying. “Take me to her.” “Relax, you just arrived from”—what's it called. We had left at 4 AM and didn't arrive until 12 PM. You know, a mother is [Indistinct] with smuggling and whatnot. So she went to [Participant's daughter]. I told her, “Of course, her hair [Indistinct].” [Indistinct] for the mother. There, in that there hospital. Also, of course, as the custom is known, no hospital agrees with another's diagnosis.

Riham: Just like [Indistinct].

Participant: Yea, like in the whole world! Nobody's convinced. [It's like they're talking about their money?] They started the tests and x-rays. They came and told me—. I had, of course, left the kids at my sister's place [either house or apartment; most probably apartment]. I found something for me to do to pass the time, something to do as work. There are expenses, you know? Of course, we're talking about going from hospital to hospital for a month and a half until I got [Participant's wife]. It was all expenses. I can't eat from the hospital's food. So, honestly, [Participant's wife] called me at night—about 7:00 or 7:30 PM. She said, “[Participant's daughter]'s test results came back from the hospital. They said, 'This girl doesn't have cancer, and she doesn't need chemotherapy either.'” “Yea?” “This girl has liver failure. She needs liver surgery, and they just called the ambulance and said, 'Your daughter is urgently sick.' They wanted to return us to the medical center in Turkey—in Malatya, Turgut Özal Hospital.” It's also a university hospital. “They put her in the ambulance, and we went.” I looked—. I also got a ticket [bus or plane] first thing that evening and caught up with them. I left work. I left the kids at my sister's. “Who's the donor?” “I'm the donor.” We go to the hospital. Of course, how did they find out that she doesn't have a—. They made another incision [biopsy] in her, from her back. Between biopsy and biopsy there's wasn't even a month, less than a month . We get to the hospital. She's already registered with them. So they did tests. They said, “This girl—. How did they say she needed liver surgery?” We started to say, “The liver is better than the cancer.”

Riham: Of course!

Participant: A person starts , but we said, “It's good it's the liver and not cancer!”

Riham: Cancer!

Participant: Each one is worse than the other, but we said, “It's good that it turned out to be the liver and—“

Riham: Not cancer.

Participant: “not cancer!” They came and made a third incision [biopsy]. They said, “What do you mean? How wasn't it clear from the first biopsy? It wasn't clear to them?” They did a biopsy. “The others don't know what they're talking about. This girl doesn't have”—what's it called—“cancer.” I'm asking here and there. They tell me this hospital's good. This was a hospital from its huge size, from the equipment inside, but at that time they didn't have any interpreters. Like, for example, if a Syrian comes in, they would say, “Go and help him” or [Participant's wife] would help. There aren't any interpreters.

Riham: Yea, but you speak Turkish.

Participant: Yea, I speak Turkish, but I'm helping them just like that for the sake of God. I used to get—. I mean, I used to get strange, strange cases, like people shot or burnt and whatnot. So, the chemotherapy is supposed to take a year. The year passes. She supposed to need a transplant after it. We tell the doctor, “She has done chemotherapy for a year—more than a year. How like—? What's the solution?” Of course for this whole year [Participant's daughter] stayed in the hospital. Like, her situation was such that for a time she lost consciousness. She lost her memory. She stopped being able to recognize me. For two months and some. [Indistinct] her memory. There was a time when she also stopped being able to see. “Well, why is this happening?” “From the chemotherapy.” Of course, in the hospital. [Indistinct] in the hospital. Then the doctor—after a year and three or four months passed—said “We have to perform a transplant on her. Does she have any siblings or relatives?” I said, “She has siblings.” I got the kids and brought them. They did tests on the kids and me and [Participant's wife], and it didn't work out. There's a one percent chance for it to work, the—what's it called. “What's the solution?” They said, “Bring some of your relatives.” And there were some of my siblings that came. I'm not going to lie to you. For example, I told my siblings, each one—. I mean they had no clue what the bone marrow transplant surgery was. Each one said, “Surgery? I'm going to give bone marrow?” They started getting scared. Some of them got scared. Some of them agreed. They thought about it and consulted each other. I said, “Doctor, I got a few siblings.” “The tests are costing a lot of money, and the government doesn't have the ability to do so many tests for one patient.” “So, what's the solution, doctor?” “We're going to see. Maybe we can find another solution.” After some time, I heard about a county called Antalya.

Riham: What's that?

Participant: Antalya.

Riham: Antalya.

Participant: Mm. There they have a bank for—what's it called—bone marrow. For bone marrow. And a special hospital—a public hospital—for bone marrow transplants.

Riham: I got you.

Participant: For children.

Riham: Like a bone marrow registry.

Participant: So I spoke to the doctor. I told him “There's such and such hospital and whatnot. Transfer me there. Maybe there they'll do—. With a waiting list, it might quickly become our turn.” He said, “No, leave your daughter here. We'll give you—. We'll talk to the hospital. If they accept, we'll immediately record the—what's it called—biopsy [perhaps he meant bone marrow] that's needed.” Indeed, I went there. I went by [bus] in 17/18 hours.

Riham: Seventeen hours?

Participant: Yea, by [bus?]. I went and signed up with was needed, and the doctor gave me a [Indistinct], of course. Then I came back. A few months later, they told me “The biopsy [again, perhaps he meant bone marrow] you requested for [Participant's daughter] [Participant's last name]—what's it called—is here. It become available.” I told them, “Okay.” We come to the hospital. I said, “Such and such happened, and this is the number

Time (3A.I) – 40:00

Participant: for the doctor so you can talk to each other.” He said, “The problem is that the decision has been changed.” “Okay, what decision changed?” “That the government doesn't pay for such a surgery.” Every county is like a state. It has a budget. As long as my ID is from this state, if they want to transfer the patient to another state, the expense is going to be taken from this state's budget. Then a decision came out of the—not from this county—for all of Turkey that a surgery that costs $350,000 is not going to be paid for. No one is going to cover it. “Okay, then what's the solution, doctor?” “The solution? [Indistinct] on the [Indistinct] that's available.” She took the chemotherapy. Anyways, she stayed in the—what's it called. After two years had passed, we took her out of the hospital. I rented a place [either house or apartment; most probably apartment], and it became weekly that she had a hospital visit for these things. So, I heard from a friend of mine that in Gaziantep they're signing up people who have diseases to travel abroad. So I went and signed up in it—the [Indistinct]—what's it called—America. Of course over the span of many visits. “Come and go, come and go, and come and go.” It took two or three years until we got out, we got here.

Riham: So she never got the transplant?

Participant: She didn't get the transplant. She got chemotherapy for three years over there—.

Riham: When you came—. Sorry, I interrupted you. When you came here, I remember that she needed a transplant.

Participant: She needed a transplant. The reports—. All the reports I have from the Turkish hospital—. I brought them. The doctor here looked at them.

Riham: Is she a doctor of—

Participant: Dr. [Marsh?]

Riham: I mean is she a hematologist?

Participant: Yea, a hematologist-oncologist.

Riham: Yea, I meant hematologist-oncologist.

Participant: The one we used to go to in the oncology department when we stayed overnight in the hospital. So now her condition is good. She doesn't need—what's it called—a surgery. So in the future if anything changes, she'll go back and consult us.

Riham: Okay.

Participant: So here, of course, they flipped and said she has a liver, too, and she has a kidney infection. When I took her she had a swollen kidney and a swollen pancreas in the [Indistinct]. So they confirmed the—what's it called. So she's also seeing a nephrologist. So, she's also going there, but here they're work like—. I noticed the hospital here in Cincinnati—. They work very slowly. They don't confirm—. They don't say that a person has a disease until they confirm it and are 100% sure.

Riham: That's right.

Participant: One or two months or five pass, and they're not in a hurry.

Riham: But isn't it better than a person telling you something is the case then changing it?

Participant: Of course! To be sure, when a person knows—. They, of course, asked me “Are you guys coming here for a visit? Just to treat the girl and go? Or have you settled in this state?” I told them “I've settled down in this state.” They said, “In that case, leave it to us to do our work, taking our time. Not like”

Riham: In a rush.

Participant: in a rush or whatnot.” I told them “I used to mix up and whatnot in Turkey and Syria—.

Riham: Yea.

Ahmed: [Indistinct]

Participant: I don't have any other choice. You're the only ones. Do what you want.” So, thank God, when we go to the hospital, we get treated well by the doctor. And the doctor is very cheerful.

Riham: And she has a lot of appointments.

Participant: She has a lot of appointments, but that problem is that—. For the appointments, it's not like there's—. There's an old lady volunteer that wanted to help us because of kids' situation. I'd never be able to keep up. I mean, [Participant's daughter]'s appointments, [Participant's son #1]'s appointments, or [Participant's wife]'s appointments. When [Participant's wife] first came here, she started getting dizzy.

Riham: Mm-hm. [Participant's wife] is your wife.

Participant: Yea. She started getting dizzy and would fall down. The organization took her—what's it called—a few times to the ER. [Indistinct] She stopped being able to attend classes with the organization. And the organization is determined that we attend them, the classes whose attendance was a condition for us. They put a condition that “You have to attend classes for three months.” After that, we have to start working even if—. So she kept falling and getting dizzy. She goes to the hospital. She goes to the doctor.

Riham: Was she pregnant at that time?

Participant: It wasn't clear. [Indistinct] Maybe she was pregnant. They would say, “No, you're not pregnant.” It took two months for them to confirm that she's—what's it called—until they were sure that she was pregnant. So that's a story. The organization sent a report to the government.

Riham: Is the food done?

Participant: They had sent a report to the government saying “This lady didn't attend the classes,” so they sent us letter saying they were stopping the food stamps and our aid because “These people didn't follow our report.”

Riham: If you want, we'll stop for—

Ahmed: [Indistinct]

Time (3A.II) – 00:00

Ahmed: Hello, I am Ahmed Beydoun. I am with participant 03A. We just got back from eating dinner. And Riham is with me here.

Riham: I also ate food.

Ahmed: Yea, so let's start.

Riham: Put the recorder between you two.

Ahmed: We had a few questions. You told us a few stories.

Participant: We spoke about—.

Ahmed: You told us what you went through. The question is how are you—. Don't you have a lot of appointments and whatnot? How do you take the kids to the appointments? How do you go to them? How do you return? You don't have a car, right?

Participant: I have a car.

Ahmed: You have a car? Ah, okay.

Participant: Sometimes we're missing some appointments. There's a volunteer—an old American lady. So she's trying to help with these things, the transportation and whatnot—.

Ahmed: With work? Does she help when you're at work and not here? Or—

Participant: Yea, for example, when I'm at work there's nobody to take them. So she's trying to help. I have Mondays and Sundays off. So she tries to make appointments on Monday when I'm off so I can go to the hospitals and take care of what's necessary for the house [run errands].

Ahmed: You told me that you missed a few appointments. Were those only because there wasn't anybody to give a ride or—

Participant: Yea.

Ahmed: Okay.

Participant: It was—. For example, in the beginning, when the kids were first asked for the urine collection tests—. So we collected urine over the span of 24 hours, and we want to take it in. There's nobody to—. Even one month, [Participant's friend], may God grant him good health—. Like, I don't know him, but I got his number when I saw him by chance. I called him and told him, “Honestly, such and such happened,” even though he was living in West Chester. So I told him, “Honestly, pardon me, maybe you have work and whatnot. If possible, the situation here is that kids were asked to collect urine by the hospital. We collected the urine over 24 hours, but there's no one to take it, and I don't know how to get there by bus or whatnot.” The guy, may God increase his blessings, came, and we dropped it off and came back. So, thank God, Master of all creation, currently things are—. Like, we're adjusting to the situation, and we're trying for it to be, for life here to be better than the one we lived in other countries.

Ahmed: God willing. When did you get the car? How long have you had a car?

Participant: The car—. It has been about six months.

Ahmed: Six months. Thank God. Did you feel more relaxed after you got the car?

Participant: Of course!

Ahmed: Because you for sure have other appointments, for example, produce and—. How relaxed did you feel? What did you used to do before if you needed produce, if you needed to shop for your place [buy groceries]?

Participant: As is normal, for example, you ask someone you know. Like, you ask a person you know. If they're free—. You know, like someone—. Thank God, Master of all creation, things would work out. We would get the produce for the whole week. We would try to take care of everything for the whole week so we won't keep—

Ahmed: Coming and going.

Participant: Yea. And we [Indistinct]. The people are good. They help people

Ahmed: I have question about something else. A little bit ago you said, when we were talking before dinner, that your son, [Participant's son #1], when he came couldn't take his medication for seven months because they would call him—

Participant: Yea.

Ahmed: They didn't identify him [his disease yet] here. Could you tell me a little bit more about what happened?

Participant: To be honest—

Ahmed: They didn't do his reports or—

Participant: No, they didn't touch the reports I brought with me. I mean they didn't care about them. They did some tests. They used to do some blood and urine tests as we were just talking about. Urine collection—diuresis so they can test it. They weren't seeing anything with the tests. So here they—. The doctor said, “He doesn't have [Indistinct[.” So I got happy and said “Thank God, maybe in Turkey they couldn't identify the disease. God willing, he doesn't have it.” I said to the doctor, “Doctor, I want to know. The kid is not taking his medicine. The medicine we brought with us from Turkey finished, and we have to find a solution for him. I mean 'Yes' or 'No.' If he's left like that—. I mean he took the medicine then stopped taking it then something is going to happen. Now, I'm not a doctor or anything but some mix-ups are going to happen with him. I'm not willing for a mix-up to happen with him. If a child is spending the night in the hospital— Like, we're really going to suffer. I mean, we have a family.” So the doctor said, “The problem is I'm trying to do all these here things”—the tests, blood, and whatnot—“I don't want to be forced to do a biopsy”—to make an incision in him. In Turkey they made an incision [biopsy] in him. Like, “It's not right for me to make another incision again. I'm trying to look for a solution without making an incision.” So after a while he told us that they confirmed that he does have—what's it called—the disease. So he first wrote him a prescription for a liquid to drink, zinc is what they were giving [Participant's son #1]. He started throwing up when they drank it. It didn't help him. We went back and consulted the doctor. We told him “Such and such happened.”

Ahmed: Quickly, just to interrupt you. Just a question. When [Participant's son #1] started throwing up, how did you get to the doctor? For example, did you speak with the—

Participant: I spoke to that person, that lady that—what's it called—we spoke with a lady that speaks Arabic to that lady—what's it called—the American. So she told the hospital. Because sometimes I—. It's happened to me for the appointments for [Participant's daughter] or what's happening with [Participant's daughter]. They gave me the doctor's number, the doctor's secretary's number. I call, and the secretary answers.”I want the”—what's it called—“department of Dr. [Kanun?] for the liver.” Her doctor—her nurse is [Rachel?]. I call, and they transfer me from department to department, but nobody answers. So at first explained—. I felt went to the hospital. At that time I had a lady from the organization with me that told us “Whatever happens with you, this is the number. Call it.” So I called and call, and nothing happens. For example, this girl, for a while, her stomach hurts her. I didn't know anymore. Should I take her to the hospital or? I didn't know what to do anymore. So I call, and they don't answer. So we go to the hospital—at that time she had [Indistinct]. So went to the—what's it called—department. I told them “Such and such is happening with me. You basically tell me 'Make a phone call, and you can speak to us through this direct line.' Nobody answered.” They said, “Impossible.” I said, “Here's the number. I'll call back right in front of you.” I really did call right in front of them, and nobody answered. They said, “Maybe she's not in the office or has something else.” So I stopped like making appointments and whatnot. I'm not able to do that. If I don't call someone that can speak American and whatnot, you end up calling in Arabic someone that speaks to someone that speaks to someone that speaks to someone, and nothing ends up happening. So there's some difficulty with regards to my not having the language. There's some difficulty speaking on the phone and him understanding anything on the phone. It's hard.

Riham: Is there something that can make things easier? Of course, you're not going to learn English tomorrow, right?

Participant: For sure.

Riham: God willing, you'll learn it some day, but is there something that can make things easier on you at this time? In your opinion?

Participant: Honestly, in my opinion, currently we're trying to find a way, a solution for these things. There's no way. For example, either someone learns the language or learns by himself or the kids learn and for them to take care of things for us. Excuse me, but it's like you're ask someone once, twice to help you with something. Maybe he's not free for me, for example. People, may God grant them good health, but a person gets embarrassed to ask “Translate what's happening or what you want.” For example, you want to go to the grocery store or fill up gas or go if something happened with someone. You're going to spend it all on the phone calling people. So a while ago, about two or three weeks ago, my youngest son, [Participant's son #2], [Indistinct] he had difficulty breathing.

Time (3A.II) – 10:00

Participant: He wasn't able to take a breath. He would take a breath—[Participant's son #2]—He would take a breath—

Riham: Sorry, [Participant's son #1] is the one with Wilson's.

Participant: Yea.

Riham: And [Participant's son #2] has no problem.

Participant: No, he doesn't have anything.

Riham: He doesn't have—

Participant: It was like he was choking. He's crying and saying, “I'm choking!” He was breathing like a rooster. Anyways, I didn't know what to do anymore. So I called—we called [Indistinct] at the hospital—the ambulance. The ambulance comes, sees him, and immediately takes him. It took him to the children's hospital [Indistinct]. So his mom went with him. They sat down. They did tests, took out, took x-rays, and said, “There's nothing.” I mean, the kid has a fever. It wasn't until 2:00 PM—they took him at seven o'clock—it wasn't until 2:00 PM that they transferred him to—what's it called—they discharged him. They wrote him a prescription. So the boy—they wrote him what—. I went and got the medicine that they prescribed for him. One of it is called a pain-reliever—what's it called—a fever-reducer. The kid's temperature doesn't change, and his cough doesn't change. He took the [liquid] medicine the first time, the second time, the same thing. The temperature isn't going down. At that time, I had called—. Maybe she was busy. So she said there's another lady that is also Syrian. She said she's a friend. I told her “Such and such, [Participant's son #2] is sick.” She tells me, “There's a doctor.” I had—the day before—I'm opening up the conversation—I had taken him again the day before—she called me when I was at work. She said, “[Participant's son #2]'s temperature is burning up like fire, and we put him some cold wet cloths, and it didn't go down.” I took him and went to the hospital. I consulted. It was now 6:30/7:30 PM. They took a look at him. His temperature's 39 C. Of course, his mom had given him [to drink]—. She had finished the bottle giving him the fever-reducing medicine.

Participant's Wife: [Indistinct]

Participant: She put him some wet cloths. His teacher called me and said, “Where are you?” I hadn't told her. I said, “Honestly,”—whats it called—“we're at the hospital.” She came to me even being that she's a teacher and knows English and whatnot. They let us in, took a look at him, took his pressure, gave him some more [liquid] medicine, too. They said, “Wait outside.” It was seven o'clock. We were still waiting until 11:30 PM/12:00 AM. “What? [Indistinct] left? Okay, but until when?” They said, “Honestly, we have a lot of patients.”

Riham: Oh, you hadn't gone in yet.

Participant: No, we didn't go in yet.

Riham: Oh, you were still waiting—

Participant: The doctor hadn't seen us yet. We were waiting for five hours.

Riham: Oh, yea, that's right. Cincinnati Children's?

Participant: It wasn't until 12:00 AM—I looked around me and saw a few Americans sitting down around me, too. Then they got up and left. It got to 12:00 AM, and there's still a few people ahead of me in line, so I got up and came back [home]. Yea, honestly! The next day, as I was telling you, she spoke—. [Participant's wife] was speaking to a lady—what's it called—that's Syrian, too. She told her, “There's a pediatrician that”—what's it called—“is Syrian.” I got his address, got off of work, and we went.

Riham: What's his name?

Participant: His name is [Hazem Hannawi?].

Riham: [Hazem Hannan?]. He's a pediatrician?

Participant: Yea.

Riham: I don't know a Syrian pediatrician so that's great that you met someone.

Participant: Yea, honestly. What's it called—He has a clinic I think. So we went and saw him.

Riham: At Cincinnati Children's or a private clinic?

Participant: No, private.

Riham: It seems that as you got to meet—. The organization didn't give you his name because—

Participant: Which organization?

Riham: The organization, Catholic Charities.

Participant: Catholic Charities didn't give us any—

Riham: They didn't give you his name. Because someone like that is really appropriate for your family. He's someone Syrian, knows Arabic, and is a pediatrician. That's what should have happened from the beginning, right?

Participant: Honestly, they didn't give us. We met him through a lady, who is also a doctor—not a doctor. Her husband is a doctor of a—what's it called—

Participant's Wife: My friend—

Participant: Laboratory, tests and whatnot.

Ahmed: He would be a pathologist.

Riham: Oh.

Participant's Wife: The same one.

Participant: I told her “Such and such is the situation.” She said “There's Dr. so and so.” Take him to him—

Riham: So you were able to see him quickly?

Participant: They called him immediately. We spoke with him. He said, “Come on over. I'm waiting for you.” He sent me his address.

Riham: Yea, to the clinic.

Participant: Yea. It took about 15, 20 minutes or more to get to him. I went and told the nurse at his clinic—what's it called—for someone to come and—

Riham: Do you have a car then?

Participant: Yea, I do.

Riham: You learned how to drive and everything?

Ahmed: He's had it for six months now.

Riham: Six months? Wow. That's really good!

Participant: It was important to me to learn to drive and get a driver license.

Riham: No, no, that's good!

Participant: Even the—what's it called—the organization, the one they call the SAF [Syrian American Foundation?] organization of the Syrians that [Indistinct] it's the one that gave us the car. From the Syrians.

Riham: Thank God.

Participant: So every family comes, and they try to help it as much as possible in terms of furnishing their homes. For example, people aren't coming from a country, from Syria. They're coming from countries where they were refugees then left them to come here. So they're trying to help people get to know one another as much as possible. At the same time, if something happens to someone. They're offering, for example, “If anything happens to you, let us know. We're going to try”—. I mean, they've been in this country for a long time. “You guys don't worry. We'll try to help you”—for example—“to show you how to do things.” So they're the ones that helped us with regards to getting a driver license, and they're the ones that bought the car. And you see the furniture—

Riham: So it sounds like the sense of community helps you meet your needs both in terms of health and household.

Participant: Yea, the necessities.

Ahmed: Like, Syrians' solidarity with one another helps—

Participant: Syrians' solidarity with one another in this here state—. I don't know what's going on in other states in terms of how they live. As for here, the people who've been here a long time are trying to help people forget the pain they lived through and suffering they experienced. So, for example, they're taking care of furniture. They brought ¾ of it. For example, like a washer and whatnot are necessary in America like—

Riham: Of course.

Participant: Like furniture. There are a lot of things. Like, let's be frank here, when someone doesn't have, I mean, he doesn't have—

Riham: Also what I'm hearing is—. I'm trying to comment on what you said. The immigrant community is also helping you with your medical needs, too, because—

Participant: How?

Riham: They were the ones that told you that there's a Syrian doctor. They're the ones that are able to take you. Not always, but in the beginning from clinic to clinic.

Participant: No, they don't take me from clinic to clinic.

Riham: Transportation.

Participant: No, the organization used to take us. Catholic [Charities]—

Riham: When [Participant's daughter] had an appointment at the hospital, Catholic Charities used to take you?

Participant: Yea, Catholic Charities used to take us. Then there's an American volunteer—

Riham: An American volunteer. Is that from Catholic Charities or—

Participant: No, she would say, for example, “I want to volunteer my time to help people.”

Riham: Is that Susan?

Participant: Yea, her name is Susan.

Riham: Susan.

Participant: So this lady—. A lot of volunteers used to come. Of course, the organization doesn't take—. Volunteers, for example, used to say “We have today. We want to volunteer by helping someone.” So this lady came and took us a few times. She used to take the kids. So she said, “I'm going to keep following up on things with this family.” Because the organization—. Two or three months passed then we didn't finish the relationship with regards to the appointments. Between two and three months we didn't finish the doctor appointments, and nothing was clear in terms of the test results—

Participant's Wife: But the test results came back.

Participant: Nothing came back.

Participant's Wife: [Indistinct] My son hadn't started yet.

Participant: They have, for example, new people coming, people coming after us, and are busy with a lot of work.

Time (3A.II) – 20:00

Participant: They don't have any translators. So communicating with them is hard. So this lady said, “I'll take them and bring them back.” With regards to the Syrian organization, for the rides there's no one that's—

Riham: In charge of it.

Participant: In charge of these things. They don't have a possibility. How are they going to volunteer? They're busy with their jobs. They're all at their jobs during working hours. So for these things it's hard for them to find solutions. When we came, the time was—. It hadn't yet started.

Riham: That's right. It was early.

Participant: When I came and saw you in the hospital, I had only been here for 10 days or more and didn't know anybody here. I mean any Syrians. I didn't know about the organization. Anyways, the organization didn't even exist. The organization wasn't even in existence. Later, they started organizing with one another and coordinating with the Catholic [Charities]. When a family would arrive at the airport, they'd go and pick them up. [Indistinct] When their food stamps first come out. They started making things easier on them. So, thank God, Master of all creation, in every situation.

Ahmed: I have a question for you. For example, I know in Catholic Charities they were giving you English classes, and there were also—. I heard they were giving you classes about life in general in America.

Participant: Yea, that's right.

Ahmed: Did anybody at any time explain to you how the health system here works? For example, how to make an appointment with the doctor? How the hospital works and whatnot?

Participant: They explained that to us.

Ahmed: They explained that to you? Did you feel you benefited from it? Did you feel that you started to understand how the health system works here? Or did you feel you didn't really benefit from it?

Participant: No, honestly, I didn't really benefit. The health system here—

Ahmed: It's different than what they have back home, right?

Participant: Yea, when I first came to America, for example—. The health system in American is all appointments. I mean its' not the same system as—. It's different from Turkey and Syria. Appointments—. For example, any doctor you go to , for example, [Dr. Esra?]. You go and say, “Such and such is happening with my son.” The problem is that we're not getting the free time to go and consult the family doctor and tell him, for example, “The kid's got the flu/a cold or got sick or whatnot.” You go to the family doctor. For example, he's working and barely making enough for rent or for the—what's it called—expenses. For example, just barely. His rent's $1,000, and you have electricity, car insurance, gas, and I don't know what else.

Ahmed: Expenses.

Participant: When someone first starts working in America they make $10 an hour.

Ahmed: It's for sure difficult.

Participant: I'm mean we're trying, by any means, that if it's not something really necessary not to leave work for that day. When a person first starts a new life, they're going to suffer for sure. So, thank God, things are better. We're finding that, God willing, everything is going to be taken care of.

Ahmed: May God grant you good health. I heard there are classes held on Sunday at the university for English.

Participant: Yea.

Ahmed: Are you guys attending them?

Participant: Honestly, we go sometimes, but if we have something, we don't go.

Ahmed: In your opinion, if there were classes other than the English—for example, classes that teach you more about things like, for example, life here in America, especially medical matters here in America. Do you think they would be benefited from or—

Participant: Of course, now, for example, as for the percentage, it depends on the family. As for me, personally—

Ahmed: Okay, for you personally.

Participant: we came to this country basically to treat these kids that I have. [Indistinct] My intention was to leave for their medical treatment. It's not like I left to live or that I want a better life or anything or that I wanted goals greater than my goals. Every person is content with their life. As for me, I was comfortable in my country, thank God. I used to have a job and was making a living even with the explosions/bombings and whatnot. It didn't occur to me to leave. If someone is going to die or something is going to happen to them, it's going to happen no matter where they are. We left [Indistinct] because the kids got sick. The kids, for example—. For every person, their kids are very special [valuable] and whatnot. So, I made it my responsibility [literally “put them on my neck”]. I mean, if there's a solution, and I didn't go after it. God blessed us with us coming to this country. Thank God, as a country, in terms of how they care of children, it's one of the best countries I've seen in the world. Here a child has rights. In Turkey, for example, a child, just like Syria, a child is like [Indistinct]. I mean they don't have any rights. Here, however, no. For example, when I came and [Participant's daughter] stayed overnight in the hospital, we didn't have insurance yet nor—what's it called. The hospital sent me the bill to my place. The price for the—what's it called—the bill was $22,000. I showed it to the organization. They said, “[Indistinct] Insurance [Indistinct].” So, thank God, the country here, from the people to the government to the medicine, thank God. So I want for someone to understand something about the medicine here so that the kids, so that we can communicate more with them and understand the extra things for their cases. I mean in the future, what's going to happen. For example, if something happens to us, where do we go? I mean in terms of having sick children with me. Do I take them to the hospital or, no, just communicate with the doctor that, for example, the doctor that's following up on their condition. These are the issues. That way someone can be relaxed, know themselves—

Ahmed: Knows what to do—

Participant: He can know which door to knock on. Of course, for example, I go to the ER. Maybe the ER gives him a medicine that isn't suitable for his health. Every patient is different. I wish there would be things like that.

Ahmed: God willing. Is there something we haven't yet spoken about that you'd like to mention? A topic that you feel—

Participant: We spoke about everything. You mean with regards to here in America? Thank God.

Ahmed: Thanks for your sharing your experiences with us. I thank you.

Participant: God bless you. You know, we're talking about the reality of the situation. Thank God, here, in terms of how you're treated and whatnot, nobody bothers anybody else. Nobody—. Now as for the hospitals in Turkey and Syria, there are a lot of problems. If you're going to meet the big shot doctor, the professor, he greets you and meets with you with friendliness and cheerfulness. In Turkey there was a doctor I used to hate. He was a professor responsible for the—what's it called. When he would talk, he would be so rude and brash with the patient and the patient's family. For example, I would ask him, “Doctor, what's the status on my daughter?”

Ahmed: What did he used to say?

Participant: He tells me, “If I told you, would you even understand? What? Are you a doctor or something?”

Ahmed: Are you serious?

Participant: Yea! I told him, “What do you mean?! I'm not a doctor, but I'm this little girl's dad. I need to know what it is.” I told him, “Such and such is happening to my daughter. Did you tell me a year? It's been more than a year [Indistinct]. Come sit in my place. Then you ask me, 'Are you a doctor or something?'” [Indistinct] You say a couple words to him, and he talks back to you so brashly. A lot of them were—a lot of them used to treat us in a really bad way. There was some discrimination and whatnot.

Ahmed: Because you were Syrian?

Participant: Some of them—because we were Syrian—used to, like, they used to swear, too. “You came [Indistinct] the country. We didn't get rid of you yet?” Even our own people at the offices used to say the same thing like “You're Syrian.” I mean, there are some good people, and there are some [Indistinct] people. They used to discriminate. In this here country nobody discriminates with these things. I'm working at the store. Like a store that has customers daily that come in and leave, and my boss is old. He tells them “This guy is new, works with me, and is Syrian” and whatnot. I don't know the language, and I don't know anything. They come and greet me and tell me “Welcome!” “Nice to meet you!” “Where are you from?” For example, a few words. “From Syria.” “Oh, Syria?” I mean, they touch [probably was demonstrating a handshake]. Whether they appear white, black, look Japanese or whatnot they don't care. Nobody, for example, looks at you like “What brought you here?” They don't do that. Thank God, not in any mall—. We went to the malls,

Time (3A.II) – 30:00

Participant: markets to shop. Nobody like—

Ahmed: You feel at ease.

Participant: We're at ease. Before we came, we were a little scared that, for example, here it's clear that we look different than other people. That Aleppo is in a bad state or Aleppo is—what it's called. Thank God, none of that happened.

Ahmed: Thank God. One second, let me see—. Riham?

Riham: Yes?

Ahmed: Are there any other questions you want to ask [Participant]? [Indistinct]

Riham: They're making me work.

Ahmed: They're making you work!

Riham: Just a minute. What was my question? Let me return to—

Participant: So I—

Riham: Oh yea. I was going to ask you—. I wanted to ask you a little bit more about mental health. So, every child has a nature.

Participant: For sure.

Riham: And every person, when they experience incidents in life, you find that they're affected by them. You know what I mean? So you guys have obviously seen a lot of things: sickness, being foreigners, moving, and all those things that your children saw, of course.

Participant: Of course.

Riham: So what you do in your family to try to help their psychology? What are some of the things you do? What are some of the things you try to do for yourself and then for your children to calm yourself down?

Participant: As for me, I'm taking care of things by myself. We, for example, left. We were in a war and whatnot. Every person—. It became like the Day of Rising. Everyone has to take care of their own problems [literally “take the thorns out of their own hands”]. Life became hard. Nobody's looking after anybody else. At the same time, the kids are [Indistinct] to our life. As for us adults, our lives have stopped. Our lives are now just for the kids we have. To give them a life better than the one we had. To live from their childhood—. For example, my daughter's now 12 years old. Once the county I was at in Turkey allowed her to go to school, they were only able to study for one year. She was 10/9 years old when she enrolled and started 1^st^ grade. And [Participant's daughter] was 11 before she studied [finished?] 1^st^ grade. As for education, in the county that we were in they it wasn't allowed for Syrians to go to school. There wasn't any education.

Riham: Are you talking about when you were in Turkey?

Participant: Yea. In the state that we were in, the county that we were in. There were some counties that would allow them to study and some where there was no education. So the kids—. Their future was lost. No studying and whatnot. So here they immediately got them enrolled in school as one the first things they do. The boy—even when he was in school in Turkey. They used to everyday come and say, “There was swearing from the kids. 'You're Arabs! What brought you to us?!'” My kids speak Turkish better than them. But still, their families—. There are some people that hate—what's it called—Syrians. To the Syrian, “What brought you to our land?” So, what do the kids know? They listen to their family. They come to school and fight with the kids. So you go and complain to the teacher, and “You don't have a right [to complain]. You're the one coming here as a refugee, as a foreigner.” You don't have any rights. Even the kids used to hate going to school.

Riham: That's so sad. They used to feel that—

Participant: For example, my kids speak Turkish. So how about those who don't know the language?

Riham: It's much worse.

Participant: It's much worse, of course. So here, honestly, they're studying at school. The boy started—. To be honest, my boy, [Participant's son #2], is a little bit of a troublemaker. What he does—. Like he doesn't—. He hates school a little. So here from the teacher's manners and style, she made him love school. On the days when he wants to go to school—. For example, like on Sunday he's like, “Is there school today?” “There's no school today.” “I want to go to school.”

Riham: That's really nice.

Participant: Students are having a good time. I mean the kids here are having a good time at school. The style of teaching and whatnot. You saw, for example, the kid just now. How many months have they been teaching them for? They started learning how to read and write. There's a concern for education, and that's essentially what's important for us, that the kids are learning. At the same time, their psychology. After I came here, the girl used to put a wet cloth [for fever] every little bit, [Participant's daughter]. So the doctor here said, “No, take it off.” I mean, he changed her psychology. She used to—. Just a few months ago, she totally looked different. You might even remember if you saw her.

Riham: Yea, I was worried about her.

Participant: She changed herself. How she looks totally changed. She got taller, her life, her energy. So one's psychology here—. For example, when you go to the doctor, when the doctor talks to her, he jokes with her, laughs with her—

Riham: That's very good.

Participant: It's not like, for example, he's talking with her and like “Let's go! Hurry up! Quickly!” Here, the way the way they treat you is all nice. I don't want to unfair with anybody. When I stayed overnight at the hospital here—. I've spent the night at plenty of hospitals in Turkey and Syria with my daughter. The problems specific in Syria don't amount to ¼ of ¼ of the problems here. Like when we spent the night in the hospital, the nurse said, “If anything happens with you, just press the button.” Like sometimes [Participant's daughter] would push it by accident, and the nurse comes smiling in the middle of the night and whatnot asking, “What do you want?” Like, for example, I spoke with my family. They asked me, “How is your daughter? How is her treatment going?” I tell them, “Honestly, the hospital—. Honestly, used to say that in our country that the nurses are the angels of mercy. Here in America they really *are* the angels of mercy.” Like in Turkey there were always arguments. Every time I would go to [Participant's wife] in the hospital, I'd see her crying. It would be that the nurse cussed [Participant's wife] out.

Participant's Wife: And I understand them.

Participant: Like with—. What was she saying?

Participant's Wife: She was taking chemotherapy and would throw up, and the nurse “What's that? You started throwing up again? You started going to the bathroom [urinating/defecating]?” And she would cuss her out. It's like, “Why are you cussing her out? She's just a little girl.”

Riham: How terrible!

Participant: This was in the department where they would give her the doses of chemotherapy.

Participant's Wife: She would throw up.

Participant: She would throw up..

Riham: Yea, but it's out of her control.

Participant: She would come cussing and arguing.

Participant's Wife: And I understand her. At that point, I'm not able to say anything. I cry.

Participant: You can't complain to anyone about anybody. Why would you complain? One time we complained to the doctor. It was a department that was specially for chemotherapy, but—

Participant's Wife: She used just go to take the chemotherapy then leave.

Riham: Yea, yea, yea.

Participant: When I would go home, she would take her and go home. Every time she would take her, she'd throw up. She would tell me that—. Of course, when she used to go, I couldn't go because I had to stay with the other kids. She would call me and tell me “Such and such happened.” I mean, what can I do about that? Take her court? Or am I even able to do anything? I can't do anything. [Indistinct] If you go talk to her and complain maybe the next time she's extra mean.

Riham: During that time period, at Children's, based on what you know about the doctors and nurses here you didn't feel the same—

Participant: No, the people here—. I spent about a week at that hospital.

Riham: Yea.

Participant: There's no nicer treatment.

Riham: Thank God.

Participant: I didn't expect to be treated in such a way, frankly. With regards to everything. For example, they told me, “What's your religion?” Even with regards to religion, they asked me, “Do you want to pray? What do you need? Do you want a copy of the Qur'an to read?” Service for everything, and at the same time, the patient—the most important thing, “What do you want to eat?” [Indistinct] You tell the nurse, and she gets it for them. There's nothing like “Eat it, whether you like it or not.” “What do you want?” Even [Participant's daughter] used to, in the hospital, used to get depressed with—

Participant's Wife: With regards to food.

Participant: With regards to everything—

Participant's Wife: Even the [Indistinct]

Participant: Now here she relaxed, psychologically.

Participant's Wife: She didn't [want to] leave.

Participant: And the place [either house or apartment; most probably apartment] we were in was in a bad condition.

Riham: I'm always in the hospital. Come see me.

Participant's Wife: God willing, as a visit.

Participant: The place we were in was in a bad condition. So she would “I don't want to leave. I want to go to a new place.”

Participant's Wife: She didn't enter the place. With great difficulty.

Riham: Okay, didn't you tell me the other one—sorry, should we finish?

Ahmed: Let's start [Indistinct]

Participant: Are the questions over? Is there any question?

Riham: Okay, if we remember anything questions, we'll ask you them later, God willing, but at this time it seems—

Ahmed: Thanks so much for your time, [Participant]!

Riham: Yea, thanks so much! Now—

Time (3A.III) – 0:00

Participant: [Indistinct] not right or wrong.

Participant's Wife: Riham, should I put you some?

Riham: Stand next to him.

Ahmed: 03A

Participant: How many times did I go to the hospital? Not right nor wrong nor did I benefit from all the times I went. Not in terms of medicine and not in terms of—for example, even in Syria and Turkey, if a kid has the flu/a cold, if he has a fever at the very least they give him something, an IV, they give him something to—what's it called—to lower his. Here, they make you keep watching and waiting but not able to come in.

Participant's Wife: They take their time.

Participant: It's not like that. In the end, you get sick of it. You get sick of it. You're waiting and waiting and waiting. She just comes and looks at you, asks you some questions, and then the nurse goes back. She comes back, asks you some questions, then leaves again. Just questions. It's clear that some things are useless like a kid with a fever or a kid that's coughing and has the flu/a cold. It's clear he needs an IV or for them to give him a shot or to give him medicine, a prescription. Even the prescription is not—. Like a kid has a fever for three days? They gave him the weakest type of fever-reducing medicine.

Ahmed: You feel that they just [Indistinct]

Participant: It's only monitoring. There's even one time when I went. I went when there were people that were with us, a few families got up and left. I left like “Am I going to spend the whole day here? We don't have work to do and errands to run and whatnot? We're going to stay sitting?” Even though we weren't even 20 people. There were maybe 20 children when the weather started to get mixed up a little, hot and cold and whatnot. All kids caught the flu/a cold. So everyone had their kids and was sitting and waiting. They got bored, laid down, would fall asleep. The kids—. Like, we gave up all hope. I saw a few people get up and leave, so I got up and left, too. I said, “That's it.”

Ahmed: If, for example, you were in Turkey or—how was your experience different? They would give you medicine immediately? How was [Indistinct]

Participant: In Turkey, when the same thing would happen, we'd go to the ER. There'd be doctors that take you in immediately. There'd be a specialist available and also a few other doctors with him—what's it called—and whatnot. Immediately, “What's going on with him?” “Such and such is happening.” They immediately draw blood. Here they don't take any. Over there they immediately draw blood and take it to the lab. Here, the least they can give you is an IV. Give an IV, for example, put something in it for infection. Put a pain-reliever in it. You find that someone went to the ER and came back. At the same time, by the time the test results came back they would have already given you the IV. “The test results came back. You don't have anything.” The doctor writes you a—what's it called—a [prescription] for medicine for infections or the flu/a cold. You take it and keep walking. In 30 minutes, an hour at most, you keep going about your life. Here I went once, and my wife went once. I started getting embarrassed, quite frankly, to take anybody to the ER. If it's in the evening, I go for 4/5/6/7 hours, and in the end, I don't benefit, so I get up and come back [home]. How many times has my wife been in pain I tell her “If you go, you're not going to benefit at all. I took you two, three times. You didn't gain anything at all. You just spent the whole time sitting down, sitting down, sitting down, okay. Then they tell you to keep taking your medicine.” “What did you benefit from coming all this way? And from all your trouble?” Now, it's different in the clinics. Clinics have care. I feel like there's not a specialist available in the ER. I'm not able to see that anyone gives a decision from them or not to take responsibility if he gave any medicine to a patient. They try to, without giving any medicine, to tell them, “Okay, God be with you!” Or they give him something very simple to “Take care of yourself with it. Manage.” This is something I saw, even in Syria that—

Interview 3A

Note: The participant's husband, Bassam, was far from the recorder, and his voice was quite low throughout the recording, hence much of what he said was indistinct.

Time (3B) – 0:00

Riham: Okay, this is Riham, and Ahmed's with me here. We're going to start an interview right now with a mom that has four kids and is currently pregnant.

Ahmed: The participant number is 03B.

Riham: Okay.

Ahmed: Okay.

Bassam: Come [Indistinct].

Riham: Yea, that's right. Sit here if you can.

Bassam: [Indistinct]

Participant: Yea, you sit here, too.

Riham: Okay.

Bassam: You'll be next to each other. [Indistinct]

Riham: Okay, first of all, thank you very much for—. You had us over, made us dinner, gave us tea—.

Participant: You're more than welcome.

Riham: God bless your hands. You know what we're asking these questions about. So, there are a few questions we talked about earlier.

Participant: [Indistinct]

Riham: We're going to repeat them again for the recording.

Participant: You're more than welcome.

Riham: So you have two kids that have diseases, right?

Participant: Yea.

Riham: And two kids, thank God, that are healthy.

Participant: Thank God.

Riham: Okay, so when we're asking these questions, please speak about all of them. Do you know what I mean? For example, if there's something appropriate for one child but not for another, so we can know how you feel. Okay? I'm going to ask you a question I thought about when we spoke that other time that your son—. What was his name? Ahmad is the one you told me went to the ER, right?

Participant: Yea.

Riham: Yea, so when he went to the ER, I remember that you told me that you didn't feel they did anything to him. They didn't send him off with any medication.

Participant: Yea, they didn't give him any medication.

Riham: Okay, so tell me. What does it mean when—. How did you feel when you left the hospital without any medicine?

Participant: When I went, they gave him a pain-reliever—a fever-reducer. His temperature was really high. And they gave him oxygen. They put it on his mouth. His chest got a little better. It got better than it was before. Yea, but they didn't write him a prescription for medicine to take at home. They said, “You can find it [yourselves].” Yea, then we found it and took him home. At night, his fever came back. So I got up and gave him a fever-reducer [a liquid]. Every time I wake up, I check his temperature and see it's getting higher. I mean, it's not going down. And they told us the medicine is every six hours. I started giving it to him every three hours. I mean, just to lower his temperature. His dad came back. I told him what happened at work. He immediately came and took him to the ER. His dad took him, too, waited. But his dad took him. We didn't call the ambulance. When his dad took him, he waited and waited and waited and waited, but it never got to his turn that time. He came back [home]. Honestly, my friend on WhatsApp was talking to me and said “You sound bothered. Is there something wrong?” Honestly, I told her “My son is sick.” She told me “What's wrong?” I told her “I gave him a cold shower, heated him up, made herbal tea for him, am giving him a fever-reducer, but no matter what it's [his temperature] going up.” She said “Mayyadah, that should never happen. It's not good if a kid has a fever for more than two days. He shouldn't take that long. Within one day a solution has to be found.” She told me about a doctor. I told Bassam immediately. They sent us the address. We got up and went to the doctor.

Riham: You mean a clinic?

Participant: Yea, a clinic. We went. We immediately spoke to the doctor, and the doctor examined Ahmad. Thank God, Master of all creation, the medicine worked with my son. Thank God, he no longer had a fever, no longer—. He stayed—. He was able to breathe like usual.

Riham: Thank God.

Participant: Yea, thank God. Now he's managing. Thank God, Master of all creation.

Riham: Thank God. Is there something that helped your experience? When you were in the hospital and went that same day. Like, you went to the ER twice on the same day. Were they able to do anything the first time so you wouldn't [need to] come back the same day? Do you got me? What I'm asking?

Participant: Mm, no. When we went to the ER, you mean—?

Riham: Yea—. No, not because you went to the ER. You went—. When I spoke to you, you said you went to the ER because you were afraid he wasn't breathing. So, of course, you called the ambulance.

Participant: Mm.

Riham: But, you went back on the [very] same day. Like, not you, but your husband brought the same boy back on the same day.

Participant: Yea, he went back.

Riham: Were they able to do anything the first time so you wouldn't [need to] come back the same day?

Participant: There wasn't a line. It was very crowded. There wasn't—. Like, “Wait, wait.” Then he saw that there was no solution, so he brought him home.

Riham: Okay, if they had given you an appointment with an Arab doctor that speaks the language and told you “The doctor will see you tomorrow morning,” would you have gone back on the same day?

[The participant does not seem to understand Riham's question, which was repeated at least three times.]

Participant: We went back, and we took him and went back. But we saw that there was no solution. So he got him and took him back [home], and the next morning—as I told you—I spoke with my friend—.

Riham: Yea, and she suggested—.

Participant: When she told me, I heard—. We said we're going to immediately take Ahmad to the doctor.

Riham: Good.

Participant: I mean, we got scared.

Riham: Yea, of course.

Participant: Like, we have two sick kids, [Unintelligible: literally “our eyes are broken”], and God forbid he be afflicted with that.

Riham: Amen.

Participant: Yea, and we're terrified. You know what I mean? If only something happened—. I mean, we're working hard and doing everything we can [literally “running with our hands and feet”].

Riham: Okay.

Participant: Thank God.

Riham: Thank God.

Participant: May God not test anybody [with something like this].

Riham: Amen.

Ahmed: Amen.

Riham: We spoke with your husband a lot about how he felt when you immediately arrived, and the place [apartment or house] was—.

Participant: To Cincinnati?

Riham: Yea, when you arrived to Cincinnati, and the place was—.

Participant: The place was really bad.

Riham: Okay—.

Participant: And we weren't able to sleep because of the heat, the disgusting smell, the bugs, disturbances from the neighbors. I used to get afraid. I didn't dare go upstairs alone. I mean, we used to get afraid. I'm talking fear. Like, we used to sleep for an hour then wake up again. One time, by chance, we were sitting down when suddenly the door opened, and a man entered upon us. Yea, apparently to fix the toilet. When we moved into the place the toilet was clogged. It was broken. I mean, it wasn't working, and the water was leaking to the bathroom—to the kitchen. Water on the bathroom floor leaks downward. Right when someone would open [flush?] the toilet, the water used to drip, and this water was dirty, flies, a disgusting smell. I mean, it was really something disgusting. We all got rashes. Even the doctors were surprised by us, that nobody remained that wasn't scratch—. I mean, no one dared come over to visit me at home.

Riham: And you were also scratching. You also all got an itch.

Participant: Yea, an itch. They wrote a prescription for us and said “You have to leave.” When the man from the environment [probably someone from the Housing Authority or the Department of Housing and Urban Development, comparable to the “Ministry of Local Administration and Environment” in Syria] came and saw the house, he said, “This house is uninhabitable.” He even went down to the basement downstairs, and it was all bugs. I'm talking bugs walking around, and he was flashing the light on them. If you only you saw the cockroaches. It was something—. I don't know.

Ahmed: How did these circumstances affect your psychology? How did you feel it affected your health?

Participant: Because there was no cleanliness, and we had sick kids—. I mean, we didn't expect such a place would welcome us. Not a place like that. Like, I left because of my kids, because of their medical treatment and to treat them. Yea, when my daughter was like that—. She was in a really bad mood. She was no longer able to—. She would say, “I'm choking. Please just take me outside. Take me outside!” But we wouldn't dare go outside.

Riham: Why?

Participant: Once the neighbors see my husband “Give us a smoke. Give me food.” They come to you, and knock on your door. He didn't used to sleep at all, my husband, but we used to sleep. He used to stay up all night. The windows are broken, and the screen was only half there. The boy—. Where—? [“What if the window fell?” or “What if he fell from the window?”]

Riham: Like, he was worried.

Participant: He was worried. He went back later, after we had been there for 25 days. We moved to this place.

Riham: Thank God.

Participant: Thank God, Master of all creation.

Riham: So you felt that your psychology improved when you changed your environment.

Participant: Yea.

Riham: Was there anything else you tried to do—? Of course, there are a million things going through your minds when you first arrive in a new country, but are there other things that helped your psychology and your kids' psychology?

Participant: Yea, for sure. When I found out that my kids' treatment, that my kids are going to be safe, that my kids are going to be well, for sure I felt safe and relaxed, and the hospital is really good. Like, they took care of us, and they're treating my kids [or “they're giving my kids medicine”]. That's the most important thing.

Riham: So when you felt that your kids are in a good place

Time (3B) – 10:00

Riham: you also felt relaxed, psychologically speaking.

Participant: Yea, and I felt relaxed.

Riham: Okay. And, of course, you're from the families that have been here longer than other families so you experienced circumstances others didn't. So your point of view is very good to see how the situation was and how it's getting better.

Participant: That's right.

Riham: I want to ask you. Were there things, in your opinion, that improved things for you? If a family is coming in the beginning/for the first time, what are the things, in your view, that they need to succeed?

Participant: They have to struggle.

Riham: What do you mean struggle?

Participant: I mean, they should be strong, never give up, and depend on themselves [literally “stay standing on their feet”]. Patience, patience—.

Riham: Patience, okay.

Participant: Patience. A person has to be patient.

Ahmed: What are some things, for example, organizations can get for them or someone else can get for them to help them? Are there things you wish were here before you came or, for example, could be—

Bassam: The most important thing is the housing.

Participant: The most important thing is—. The most important thing is the housing. The most important thing is the housing.

Bassam: In areas that are safe, that are—.

Participant: That don't have fear. That have good neighbors where you're safe. Where you're able to put your head down on your pillow and relax. Not where you go to sleep and are afraid inside “Someone's going to knock on the door right now. Someone's coming right now. Someone's going to do something to me right now.”

Ahmed: Okay.

Riham: With regards to food and drink, did you find yourselves—. I mean, were they helping you, in your view, to—. Go ahead and speak. It's okay.

Participant: No, it's okay. The first thing was that they stopped the food stamps. They decreased them for us, the food stamps.

Riham: They decreased them?

Participant: Yea.

Bassam: No, like, they gave us a paper that said they're stopping them because of—what's it called—.

Participant: I wasn't attending the class. I was sick. I got pregnant and wasn't able. I went to the doctor, and he wrote me a report. He said, “You're not able to go [to the class], until you get better.”

Riham: Because you were throwing up a lot.

Participant: Yea, I was throwing up a lot. Yea, so when the doctor gave it to me, we gave it to them—. We showed it to them. They weren't convinced.

Riham: This is Catholic Charities?

Participant: Catholic Charities.

Riham: Okay, so if you don't go to class, they cut you off?

Participant: Yea.

Riham: Okay.

Bassam: It's like they punished her for—.

Participant: Because of me—

Riham: A punishment.

Participant: my kids and husband were punished, the four kids and me. I told them “Punish me. What did my kids do?”

Riham: That's right.

Bassam: When a refugee comes, they have financial assistance, cash, and food stamp assistance. The financial assistance is to pay rent until they find work. So they stopped the financial assistance. I went back to the organization. They said, “Because your wife isn't attending the class.” I told them, “I'm attending the class.” And my wife, you know, you used to help her [take her to the ER] and take her to the hospital. You know her condition. You say you're not convinced with her condition, and the doctor is [Indistinct]? Her condition doesn't allow her to leave the house. I mean, she's getting dizzy, and—. Her condition that doesn't allow it.

Participant: The doctor didn't allow me to walk alone. She said, “You can't walk alone in the street. You can't.” It was [Indistinct] for me to fall down on the floor.

Riham: Because you started to faint, right?

Participant: I used to throw up so much I would faint, and the doctor prevented me. She said, “You have to lay down and relax.”

Riham: Like, you used to lose consciousness, right?

Participant: Yea, I used to lose consciousness.

Riham: Yea, yea. It happened to you however many times.

Participant: It happened about four, five times. It happened in front of them. Right in front of them it happened. [Indistinct] I mean, they saw me. How many times did they witness it?! But they weren't convinced.

Riham: So, what ended up being the solution?

Bassam: I started working, but in the beginning they used to give me four-hour shift. Four hours on [Indistinct] nine, on cars, and the cars—. [Indistinct] The place [apartment or house] is 977. That's only the place's rent. I used to make about half that amount in the whole month.

Riham: Yea, of course.

Bassam: I started to have psychological stresses. I came to this here country to [Indistinct] live. I mean, they're giving food stamps, but I have electricity [bills]. I have rent. There are other expenses, like [Indistinct], cleaning supplies, a lot of things I have to—. Like the phone [bills]. So I went back and forth to the organization for three months. We were applying, and I don't know what else. Up until the last month, that lady who was coming and helping us—.

Riham: That's Susan.

Bassam: Susan. So she knew what was going on, that I was going and coming back. In the end, she started communicating with the organization to no avail.

Participant: They didn't [help/respond to] her.

Bassam: They don't care about the issue. They're not living under the conditions I'm living under. How am I supposed to keep up with everything—keep up with the kids and my wife because she's sick? What did me and my children do? She didn't attend. She's in a sick condition. Then [Indistinct] her assistance. I mean, why is it a collective punishment? We [Indistinct] if she got sick or got pregnant, for example, I will take full responsibility. So I spoke with my boss. He increased my hours.

Riham: Where do you work now? Sorry I forgot.

Bassam: I work at a shoe-repair shop.

Riham: Oh, shoes? Like your thing [profession], that you learned—.

Bassam: My thing is manufacturing, but here it's repair.

Riham: Oh, your thing is manufacturing—,

Participant: Yea, manufacturing.

Bassam: I didn't find a shoe-manufacturing job here in Cincinnati.

Riham: and you didn't find manufacturing?

Bassam: So—what's it called—I'm working in repair.

Riham: At least it's a little close to what you—.

Bassam: Yea, it's close to my work and what not.

Riham: learned.

Bassam: I mean, considering I don't know the language. So the boss assessed my situation, being that I have responsibilities, rent, and whatnot. He then made it full-time for me. Do you know what I mean? At the same time, I'm always thinking about how I'm going to get the money to pay off all my bills at the end of the month. [Indistinct] also the plane ticket yet. Refugees arrive, and after three months—.

Participant: You have to pay for the plane [ticket].

Bassam: The plane we came in—.

Participant: We have to pay.

Bassam: For example, we have a debt of 5,000 to the government. They break it up for us into equal payments.

Riham: That's something new to me.

Ahmed: I've never heard of that before.

Riham: That's a misfortune.

Participant: That plane—.

Riham: Five thousand?!

Bassam: Yea.

Participant: Yea, and my husband is the only one working.

Bassam: Every month—.

Participant: Rent, heat, and electricity is all on him.

Bassam: In the winter, the rent—the electric bill used to be around 200, 190. The place [apartment or home]—.

Riham: Lower it. Lower it!

Bassam: It's cold! The rent would come out to 977. The internet would come out to 35. The phone—. Both phones come out to—. These are necessities.

Riham: Yea.

Participant: Elecricity.

Bassam: We also have the car's gas, car insurance—. You calculate the month—. Even on some days when I catch the flu/a cold and was tired, I go to work—.

Participant: He's forced to go to—.

Bassam: If I don't go one day, anyways I'm in debt. I mean, I'm in real debt. I'm calculating everything by the penny just to—. From a few [Indistinct] the money was coming in. Do you know what I mean? I mean the financial assistance. At that time we had a little bit of money with us. I began to pay—. My job wasn't enough. So that lady went to the—what's it called—to engage an attorney. There's a group of lawyers that work pro bono.

Riham: That's really good!

Bassam: [Indistinct]

Participant: Honestly, that lady is really great.

Bassam: I mean, she's seeing—. She comes over every few days, takes Mayyadah to the hospital, takes the kids to the hospital. She sees that we [Indistinct]—.

Participant: Because she—.

Bassam: It's clear that [Indistinct

Participant: My doctor's appointments are every week.

Riham: Yea.

Participant: They made them closer together and made them every week.

Bassam: What I am doing—. Every month—.

Riham: Because you're pregnant.

Participant: Yea, because I'm pregnant.

Bassam: Anyways, how much I'm making each month is clear.

Riham: Yea.

Bassam: It's not like I paid for anything outside of the [Indistinct]. Not even a penny. I'm still coming out in debt. Like, I smoke. Cigarettes alone, car expenses, the kids—. I mean, if they need anything like shampoo, cleaners, and whatnot—. Food stamps don't cover them.

Riham: Of course.

Bassam: So, I mean, I started to have a lot of psychological stress—.

Time (3B) – 20:00

Participant: Even diapers.

Bassam: I started to hate—.

Participant: My son uses diapers. He's six years old.

Riham: He still uses—?

Bassam: From when he got sick with the disease, the medicine—.

Participant: He uses diapers. From when he got sick with the disease, we've been buying him diapers. He's not able to without diapers.

Riham: And diapers are expensive. Very expensive.

Bassam: They're expensive—.

Riham: I mean—.

Participant: It needs cash, too. It also needs cash.

Riham: Food stamps don't—.

Participant: No, they don't cover them.

Riham: cover the diapers.

Bassam: Food stamps are only for food.

Participant: Only produce and food.

Bassam: So, she engaged an attorney. She went to the—what's it called—[Indistinct]. She explained that my situation is such and such.

Participant: We're going to have two soon instead of one diaper.

Bassam: [Indistinct] so we can relax. What am I going to be alone? Put yourself in my place. Where are you going to get from? Expenses or whatnot or this or that?

Riham: Sorry, do you have a case manager or case worker? Like, someone responsible for you?

Bassam: Of course, the organization is responsible.

Riham: But the organization—. What I'm hearing from you is that—.

Bassam: For three months.

Riham: Only for three months.

Bassam: For three months [Indistinct]—.

Participant: [Indistinct]

Riham: And after the three months, do they have someone for you, for your family? I mean, for example, your wife is pregnant. There's something called WIC. Have you heard of it?

Bassam: What's that?

Riham: WIC is when a lady has a baby. They give milk [formula], money for diapers, and things for the baby, too.

Bassam: No, honestly—.

Riham: So they have to transfer you—. They have to sign you up for it. It doesn't come for free. Sorry, it doesn't come automatically. Someone has to help you to do it. Do you know what I mean?

Participant: WIC right now—.

Riham: I don't mean right now. I mean, after you have the baby.

Bassam: [We have places?]

Riham: Okay. Just so I can understand the situation more,—.

Participant: [Indistinct]

Riham: there's no one that comes to you—a case manger or case worker—that tries to help you like “This kid needs this. That child needs that—.”

Participant: [Indistinct]

Riham: “You need this.”

Bassam: No, there's nothing like that.

Riham: Okay.

Bassam: The organization is supposed to keep following up—.

Riham: At least a little bit more—.

Participant: To know our situation—.

Riham: Three months is nothing.

Bassam: We spent the three months full of problems with them because of the housing issue—.

Riham: Yea, and your daughter was sick, too, at the same time.

Bassam: [Indistinct] I told them, “Take me back to where you brought me from. Take me back. Did I come here to—?

Participant: We suffered psychologically—.

Bassam: suffer psychologically over—?

Participant: when we first came.

Bassam: I mean, I'm supposed to take care of all of this myself [literally “It's all on my head?”]? No. Take me back there. It's an easier country.” Here, rent alone is a disaster. [Indistinct] something [Indistinct] like rent.

Riham: Of course. That's right.

Bassam: So we—. As I told you, a week ago—. Last month, we went to the university/mosque and applied. In three months of coming back and forth I didn't gain anything. So we went to the—what's it called—the university student [Indistinct]. We explained to him “The situation is such such. This is what happened.” There's apparently supposed to put back into action the—what's it called. I mean, it's about the level of my pay. [Indistinct] And which expenses I have that are clear for them. I'm talking about the essentials. Like the air that I breathe I have to pay for it. So based on that, they are going to continue to give me—.

Riham: Food stamps.

Bassam: The cash, cash.

Riham: Oh, the cash? Okay.

Bassam: They went back and started giving me cash again. For example, I'm making about 1,400. It's not enough. So, for example, they need to put 200, 300 a month. On the basis that this month is going to start. So, we—. [Indistinct] Like they would say, “You're the one responsible for the family. You have four kids and a wife. You're the one responsible.” I mean, “I'm responsible for them. Okay, then what am I supposed to be?” I don't know the language or have finances to be—. Even the people before me and that came after me are [Indistinct] with the same situation. The work they're doing doesn't have overtime. I mean, it doesn't have extra pay. It's just from 9:30 to 6:00. I mean eight hours of work. The store closes, so there's no opportunity to stay working.

Riham: Yea, if you were able to, you would've worked more.

Bassam: Of course, I would've worked more because I have to work more. The country here—. For example, give him a salary to [Indistinct]. I'm in debt/behind because of the rent. So now I have to go back and pay the expenses. [Indistinct]

Participant: Who's going to work? There's nobody to work.

Bassam: Even, when I first started, it wasn't enough. The mosque paid for my—what's it called. It helped us with about $400 to cover our place's rent.

Riham: Thank God.

Bassam: [Indistinct] helped all the people because [Indistinct] can't find any work—.

Riham: Okay, is there someone helping you with—? It's really good the mosque is helping you with the rent—.

Bassam: No

Participant: No, not always.

Bassam: It was only one time.

Riham: Only one time.

Bassam: They gave to everyone there. [Indistinct] $400 [Indistinct]

Participant: They didn't give any more after that.

Bassam: There were some people who came a couple months or three months before me and still weren't working. So they gave—.

Participant: To all of them.

Bassam: [Indistinct] to all the [Indistinct]. $400, $400 [each].

Riham: But at least they help a little bit with the furniture, help a little bit with whatnot—.

Bassam: As for the furniture, [Indistinct] was in something like a store-house. Some people bring it—.

Riham: Is that Rahma?

Participant: The mosque, Rahma.

Bassam: Yea.

Riham: Jackie's Rahma.

Participant: Yea, Jackie's Rahma.

Riham: Yea, Jackie.

Bassam: Because, for example, people buy new furniture for their places [Indistinct]—.

Participant: I mean, we threw away the furniture that they put for us.

Bassam: The organization [Indistinct]—.

Riham: The organization—.

Participant: We threw it out.

Riham: It had bugs in it, right?

Participant: It had bedbugs in it.

Riham: Yea.

Bassam: They gave us used things that were in a bad condition. [Indistinct]—

Participant: They force you to take it then you get bitten.

Bassam: [Indistinct]

Participant: The ones that came over to my place.

Riham: Yea.

Participant: I mean, honestly, they would tell me, “We came over to your place. Look what happened to our bodies.” We used to want to sit down. We used to want to sit down on the floor, but the carpet was all dirty. It was full of hair, dog hair. It was full of a smell—.

Bassam: [Indistinct]

Participant: It was like you were entering a sheep pen. You don't feel like you're entering an apartment. You need to sleep. You need to eat. I mean, it smelled like ass, like ass. When the door first opens—. Even when they used to pick us up from there, they would enter the place with us—.

Participant: Right when they enter, especially the ones from the organization, their appearance would [Indistinct].

Bassam: Even the manager/director—

Participant: The manager/director wasn't able to handle it.

Riham: And she's the one that made you move there!

Bassam: [Indistinct]

Participant: She's a manager/director and wasn't able to handle it.

Bassam: Weren't we coming back after complaining in the hospital when we spent the night?

Participant: So she came to check it out.

Bassam: She came to see the house.

Riham: With her own two eyes.

Bassam: [Indistinct] Zalikhah was discharged from the hospital. We got her and left.

Participant: I mean, she wasn't able to handle the smell or the heat.

Bassam: She [Indistinct]

Riham: Is her name Megan?

Bassam: Yea, Megan.

Participant: She wasn't able to handle it for an hour, but we stayed there for 25 days.

Bassam: [Indistinct]

Riham: Hopefully you told her.

Bassam: I stood talking with her at the door. I said, “Put yourself in our place. Would you live here?”

Participant: She didn't want to enter because of the smell. Honestly, it smelled like ass.

Bassam: [Indistinct] the contract—.

Participant: If you guys would only see this place—,

Ahmed: When did you sign the contract?

Bassam: I didn't sign anything.

Ahmed: Oh, really? [Indistinct]

Bassam: From when we were in Turkey we explained the situation to them, the UN.

Riham: Yea.

Bassam: That if the house's furniture is used and in a bad condition, don't [Indistinct] it in the place. Don't [Indistinct] it in it. And the assistance was supposed to be for at least eight months. [Indistinct]—

Participant: Not three months.

Ahmed: They promised you eight months?

Bassam: It was supposed to be for eight months.

Participant: They told us eight months.

Bassam: They gave us a—.

Participant: Before we left, all the decisions changed.

Bassam: They gave us a—what's it called—.

Participant: I mean, I don't know.

Bassam: a cultural course about life in America, what your rights and responsibilities are. Because you're going to a country with conditions to live that are different than our life. They gave us a for three months [maybe he misspoke about the length here and meant three days instead]. They requested me—. I was in Malatya—. To Istanbul, and they put us in a hotel. Because we went for 17 hours with the “kamyon” [Turkish for “truck,” but perhaps he meant “bus”] for three days—.

Riham: The course—.

Bassam: For the cultural course and how life is here in America.

Participant: We went back. We were in the bus.

Bassam: So I told them, “The whole time what the UN was telling us in Turkey is different than—

Participant: None of it happened.

Bassam: the life we're living here. [Indistinct] “They made fools of you.” [Indistinct] And from there they made fools of us. What they told me was official. It's different [Indistinct]. Honestly, they told me—. I'm telling you, I don't know the language, and I'm not a [Indistinct]—.

Riham: You're not coming to your brother's or anything.

Bassam: I don't have a guarantor/protector. You're my guarantor/protector, and I don't have anybody. I don't know the language. [Indistinct] I have to learn and find work. We used to look for work. Companies wouldn't hire. Like, “You don't know the language—.” I mean, you couldn't understand one another. They would tell you, “Why should I go through the trouble for them? [literally “Why would I give myself a headache with them?”] Take care of things yourselves!” We went to a cleaning company. They didn't hire us. Now I'm working, and I'm suffering.

Riham: And you, Mayyadah, did you try working in the beginning or—?

Participant: No, honestly. I am with my kids and barely keeping up. Four kids, and two kids are sick, as you know. I mean, they need care. Their mom has to check up on them, feed them, and take care of them. The place [apartment or house]—. I didn't work.

Riham: I mean, soon after you starting getting sick.

Participant: Yea, later I got pregnant. From the time I got pregnant, the throwing up started with me. And I didn't used to be able to—. Sometimes my husband would come from work and cook. Like, he would come and not find any cooked food and nothing. I was sick

Time (3B) – 30:00

Participant: Either he takes me to the ER when I'm really sick—. Then I would think he was going to say “Where should I leave the kids and where should I take you?” You can't leave kids home alone—.

Riham: No, not at all.

Participant: and get up and leave.

Riham: No, not at all.

Participant: Yea, then I used to—. I mean, all night there was endless throwing up.

Riham: Okay, that's it then. Don't get pregnant anymore. Did you hear? He said his mom had 20 kids or however many.

Participant: Sixteen!

Ahmed: Sixteen!

Riham: Now they became 20. If you think about it, she was throwing up for nine months. How kind of life is that?

Participant: Yea, my appetite is really bad. I had four that way.

Riham: Oh, they were all that way?

Participant: Four that way. Yea, four.

Riham: Glory to be God, every woman is different.

Participant: Back home, my mom used to be [sweet?] before she passed away—.

Riham: What did they used to do there for the vomiting? I mean, were you able to use anything for the—.

Bassam: It used to be “fawwar.”

Riham: Say again?

Bassam: “Fawwar.”

Participant: “Fawwar.”

Riham: What does “fawwar” mean?

Participant: He used to make me eat and let the baby—.

Riham: What is “fawwar?”

Participant: It's a vitamin. [She probably means “Fawar Fruit Powder,” which contains sodium bicarbonate, tartaric acid, and citric acid.]

Riham: A vitamin called fawwar?

Participant: A vitamin like vitamin c.

Ahmed: Fawwar is like something you put into water—.

Participant: That's it! It's like the one you have for the flu/a cold.

Riham: Alka-Seltzer?

Ahmed: Yea, [Indistinct].

Participant: They don't prescribe it. No matter how much I asked them for it, they don't prescribe it.

Riham: You can't find it in the pharmacy?

Bassam: We don't know—.

Participant: I don't know—.

Riham: That's Alka-Seltzer.

Ahmed: I'm not sure if it's necessarily that, but it's the same—.

Riham: Tell me about it.

Bassam: [Indistinct]

Participant: It's “fawwar.” If you want it, you put it—.

Riham: It's circular—.

Participant: You put it in water—.

Riham: You put it in a glass of water—.

Participant: A glass of water—.

Bassam: [Indistinct]

Participant: Then it bubbles up—.

Riham: Yea!

Participant: Then I drink it. After drinking it, I have an appetite. I stop throwing up.

Riham: And after all this time you didn't know what it was?

Participant: No, I told them what it is. I told them “fawwar.” I just showed you the vitamin medicine.

Riham: Yea.

Participant: The two big pills/tablets.

Riham: Yea.

Participant: You said it's good.

Riham: Yea.

Participant: Over there they call this “fawwar.” The interpreter is not explaining to them what I mean.

Bassam: [Indistinct]

Riham: Because they're bringing you someone Moroccan, who isn't going to know—.

Bassam: For example, as much as he used to live in—what's it called—our region he knows—.

Participant: Right now, when I said it you immediately know what I was talking about.

Riham: Yea.

Participant: I—. The doctor said—.

Riham: Yea, if you had told me what it does, I would've understood what you meant, too because this—. My grandma used to do the same thing. She used to put the Alka-Seltzer—. She used to really like it.

Participant: And this is—.

Riham: Yea, and it's really cheap here.

Participant: Really?

Riham: Yea.

Participant: Honestly, I had no clue—.

Riham: I'll write it down for you right now, God willing. God willing you won't throw up, but it's just to keep it with you at your place just so you know. Were there other things you used to do like take herbs or anything else to help with your nausea or to help with sickness, either for you or your children?

Participant: No. For me and sometimes for my sister, there's no energy [literally “ability” or “power”] at all. I don't eat. Every time I eat—. Every time I eat, I throw up. I drink water then throw it up. It didn't used to stay down. And I didn't have any energy to run after her kids. I walk, and I don't have any energy. I didn't used to be able to at all. I was tired.

Ahmed: Were there things others than the “fawwar,” like things we have in our societies [Syria] that the translators don't know about? Did you face anything more like that? I mean other than the “fawwar?”

Riham: For example, cumin. Some people use cumin. They boil cumin. Some people use—.

Participant: I don't take—.

Riham: You don't take such things?

Participant: Not cumin or anything like that. The doctor told me to drink ginger soda.

Riham: Yea, yea.

Participant: Yea, well it didn't have any affect at all. They even changed the water. It was a carbonated water they brought me—they told me about. My husband also went out and got some for me. Yea, well, I just felt it went down a tiny bit. A week didn't go by before it went back. They took me back to the doctor and were talking to my doctor “Such and such is her situation.” “Give her water. Let her drink water so the baby's water [amniotic fluid] doesn't dry up.” She was saying “The more she throws up, the more the baby's water goes down.”

Riham: Of course.

Participant: “The mom ends up taking the baby's water. Then the baby gets up and wants water but there's no water around him.”

Riham: Yea, yea. Okay, as Ahmed asked you, in your family, you don't use honey or anything like that?

Participant: No, just like—. It was “fawwar.” I mean “fawwar” was really great. My husband used to always rinse out his mouth with it really well. He used to bring me two boxes instead of one. I used to drink it—. I mean, it's a vitamin for the baby and also a vitamin for me, and it used to give me an appetite. I mean, it's really great. I tried telling the doctor. I said “fawwar,” and it looks like this—. It didn't—. I felt—. I don't know, the interpreter—. So, I go to get the medicine from a pharmacist, and I say, “I don't want this. I want 'fawwar.' Its box looks like this.” I do—. I mean, I don't know English.

Ahmed: [Indistinct] Sorry. The problem is also that the interpreter also doesn't know—.

Bassam: [Indistinct]

Participant: The interpreter is always—.

Bassam: [Indistinct] Even with [Participant] trying to explain herself, it's clear that her language [Arabic] is a little weak—.

Riham: If her language [Arabic] is weak then what about mine?

Ahmed: It's best we don't say.

Riham: God forgive you!

Bassam: [Indistinct]

Participant: I wish they would put a translator like Ahmed. Ahmed immediately [Indistinct] from his dialect—.

Riham: Thank God.

Participant: He's great.

Riham: There's nobody like him.

Bassam: Like, for example, if she's speaking with—. For example, you're speaking Syrian or you're speaking in the Damascene dialect, and she's able to understand and communicate with you.

Riham: Yea.

Bassam: But as for a—.

Participant: A Moroccan—.

Riham: Yea, Moroccans are unintelligible anyways.

Bassam: Or an Algerian or an—.

Riham: Algerians are unintelligible.

Bassam: There are some Palestinians that also say some words—. Or an Iraqi—.

Riham: An Iraqi.

Bassam: who tells you “shaku maku” [“What's up?” or “What's going on?”].

Riham: “Shaku maku!”

Ahmed: Honestly, they say that!

Bassam: [Indistinct]

Participant: There are a lot of words.

Bassam: She doesn't have—. It's not like her Arabic is [Indistinct] with the other languages. Syrian people understand other languages, but other languages don't have any clue about the Syrian language.

Participant: Because I also lived in Turkey for five years. [I flipped?] to speaking Turkish all year.

Riham: Yea, because you used to speak Turkish from the beginning.

Participant: Yea, I speak Turkish, but I used to teach myself to speak with my husband. I wouldn't speak Turkish. I'd try to speak mostly Arabic. Yea, but when we came to Turkey, I was forced. I had to say everything in Turkish. The pharmacist and the hospital. I used to be able to communicate with them.

Riham: Yea, yea.

Ahmed: Did you try using any Turkish interpreters here? Or is the interpreter always an Arab?

Riham: Yea, honestly, that's right. That might be a good idea.

Participant: Yea, well, they now have written on my report that I need an Arabic interpreter.

Riham: Yea, but you can request—. Because there are a lot of Turks here.

Participant: Really?

Riham: Yea, in this area there are a lot of Turks, Turkish restaurants, and whatnot. So maybe they have Turkish interpreters. I mean, you might be able to communicate better with them.

Participant: No, but when we went to the hospital—we took Zalikhah and Muhammad—when we spoke with the doctor, a doctor saw that the kids and I were speaking Turkish. They asked us “What language are you speaking?” The interpreter was asking us. Not the—. The doctor asked her to ask us. So we told her “Turkish.” So the doctor said “Okay, then that's it. This is great. You know Turkish and Arabic. Tomorrow you'll study and become an interpreter for us.”

Riham: Honestly, that's true.

Participant: “You'll become an interpreter.” At that time the doctor said “There aren't a lot of interpreters for Turkish. You'll be able to help the Turks.”

Riham: Yea, that's true.

Participant: At that time, it was just talk—.

Riham: It's a good idea.

Riham: Just talk like that—. How do I explain—. He said, “There aren't any Turkish interpreters.” You know what I mean?

Bassam: [Indistinct] hospital [Indistinct]

Participant: The most is Arabic.

Riham: Excuse me?

Bassam: It doesn't seem it's prepared for there to be a lot of Turks in it because it's hard for Turks to come here.

Ahmed: [Indistinct] Children's is of course going to have more Arabic interpreters in it—.

Bassam: It's all Arabs.

Participant: It's all Arabs.

Ahmed: because they're always coming from—.

Riham: Yea, but in it—.

Ahmed: the Gulf to Children's.

Riham: Yea, but there are Turks that work—. Like, I know Turks that are working in the hospital because there's a Turkish community here.

Bassam: There's a community. For sure there's a community.

Riham: Yea.

Bassam: It's not like they come because of this—they come so they can seek medical treatment. They're coming to work. For example—.

Riham: No, but for example, by phone. Weren't you saying that you're finding a lot of trouble on the phone interpreters? Maybe you'll get a Turk whom you will understand immediately instead of a Moroccan because—.

Participant: No, there are always other languages, but Turkish doesn't come up.

Bassam: No, immediately, they request Arabic. What is there? Arabic, Arabic, a million countries.

Riham: Do you get it? Arabic is—.

Ahmed: More than one language.

Bassam: [Indistinct]

Riham: Yea, Kuwaiti is unintelligible.

Bassam: Bahraini is—.

Participant: Yea, and when they [talk?], we say “muraja'ah” [vomiting]. They say “istifragh” [vomiting].

Riham: No, as Damascenes we say “istifragh.”

Ahmed: We say both.

Riham: Yea.

Ahmed: I say both.

Participant: No, they also say—

Riham: Some people say “'ammi tarrish” [It means “He is spraying” or “He is vomiting,” depending on the Arabic dialect.].

Participant: “'ammi tarrish.”

Riham: Yea.

Participant: What is that, “'ammi tarrish?”

Riham: Do you know what I used to think?

Bassam: He is spraying himself.

Riham: Yea! I used to think—. Because I used to work with a lot of Yemenis—.

Participant: I used to ask her again “What is 'tarrish?'” She would say “[Indistinct]”

Riham: I used to think it was water, meaning they spray themselves with water.

Participant: I would say, “Me? Spray?!”

Riham: I used to say, “Why are you coming here to tell me you're spraying yourselves with water?” There isn't any—? Then later I learned what it meant. For “skhuneh” [a fever], they say—.

Participant: After I came here,—.

Riham: Do you say “skhuneh?”

Participant: Yea.

Riham: They say—. What do they say? Yemenis say a word I've never heard of before. What is it? I forgot. They don't say “skhuneh.” They don't say “hararah” [a high temperature].

Ahmed: [Indistinct]

Riham: Huh?

Ahmed: I don't know what they say.

Riham: Honestly, I forgot.

Ahmed: It's something else for sure.

Riham: Every country has a—.

Ahmed: [Indistinct]

Bassam: [Indistinct]

Time (3B) – 40:00

Participant: I mean, a lot! I didn't happen to—. I didn't go to Kuwait or “Suwait” or here or there. I didn't go there. I mean, I don't know.

Bassam: A person is supposed to, when they know the basic language, Modern Standard Arabic [MSA]—. It's a language that unites all Arabs—.

Riham: That's true.

Bassam: all of us.

Participant: Yea, that's the best.

Riham: So, if he tells you something in MSA, you understand him?

Bassam: Nay.

Riham: Nay!

Participant: It's just—. I don't know.

Riham: Did you learn how to read and write in Arabic?

Participant: I know, but—. It's not like—. I sound it out and figure it out. But with speaking I find the most difficulty.

Riham: That means it's hard for you to fill out forms. If they write them out for you in Arabic, there's still difficulty.

Participant: There's a lot. Yea, there is. But as for mail—. If someone helped me—.

Ahmed: Do the interpreters help you fill out forms in the hospital?

Participant: Yea.

Bassam: Of course, they're all in English [Indistinct]

Participant: They help me.

Riham: But do they read or do they transl—. Like when I—.

Ahmed: [Indistinct]

Bassam: [Indistinct]

Participant: They read it for us. They say, for example, “Such and such.” For example, just like with us when we were filling out some forms.

Riham: Like what we did.

Participant: Exactly!

Riham: Okay, so they help.

Participant: Yea, they do help. No, they do help. It's not that they don't help.

Bassam: [Indistinct] the interview, they give [Indistinct]

Ahmed: If you want.

Bassam: either in English or either in—what's it called. Sometimes, there's an interpreter. He gives it to you in English then puts in your file that you're [Indistinct]

Ahmed: Okay

Bassam: As for the kids, they give them–what's it called— [Indistinct] visiting the doctor.

Riham: Okay.

Bassam: [Indistinct]

Riham: Okay, what else are we doing with questions? I don't remember. Barriers to healthcare—

Ahmed: Yea.

Riham: like healthy behaviors, I'm really getting nothing. Before, when I was talking to your husband, he was telling me something that also bothered him with regards to housing is that the kids aren't able to go outside and play—.

Participant: Yea, they can't alone.

Riham: These are health-related, right? A kid has to run for their body—

Participant: They have to see the sun.

Riham: They have to see the sun, have to breathe air.

Participant: No, they don't go outside without us.

Riham: Yea, they don't go outside without you.

Participant: I don't dare go outside alone. The doctor told me “Go outside a little, take a walk for change of scenery.” I don't dare—. “You have to go outside for a change of scenery, like sit down in the park a little. You have to—“ But I don't dare. When my husband comes, he gets home tired from work in the evening around 7 PM. Yea, so by the time he has dinner and whatnot—.

Ahmed: It gets dark.

Participant: Yea, it gets dark, and we don't let the kids go out alone.

Ahmed: How is your relationship with the other Syrian refugees here? Because I know there is more than one family in this neighborhood. How's your relationship with them?

Bassam: [Indistinct]

Ahmed: I mean, for example, do you help each other? Do you go out together or not that much?

Bassam: Honestly, during the week we see each other at the mosque.

Participant: We gather at the mosque.

Riham: What do you do at the mosque?

Participant: So they can teach us the language [English]. So we can learn English, too.

Bassam: [Indistinct] for the kids.

Riham: Is there also food or—

Bassam: No [Indistinct]

Participant: No, there's no food.

Riham: Or socializing?

Participant: Yup, there's socializing.

Riham: Okay.

Bassam: [Indistinct] they [Indistinct]

Participant: There's no food and whatnot.

Riham: There isn't any.

Bassam: [Indistinct]

Riham: Only a class.

Bassam: Yea, I mean lessons.

Riham: Yea?

Bassam: They see, for example, how someone from first grade to second grade [Indistinct], someone whose language [English] is still [Indistinct]. They try to train in the language to be able to explain how to in English and how to in Arabic. I mean, it's language teaching. The kids are the same. The kids also—.

Riham: So the kids go to the mosque, too?

Participant: Yea, them too. Everyone by themselves.

Riham: Oh, okay.

Participant: [Indistinct] a class, help.

Ahmed: Are your kids, for example, friends with their kids, the kids of the other Syrians here?

Bassam: [“They're friends but—“ or “It's hard.”]—.

Riham: Are there kids their age?

Participant: No, there aren't.

Bassam: In this area there aren't any.

Ahmed: Oh, okay, okay.

Riham: Because sometimes when kids are together, you find that their psychology improves, too.

Participant: It becomes better.

Riham: Because they then have friends, speak their language, and all that.

Participant: No, but in the mosque, when they go, yea, they gather with each other and have a good time more than the class.

Riham: Yea.

Participant: When they see each other, they say “If only we were neighbors.”

Riham: Yea, yea. So they're in different areas far away from you?

Bassam: No.

Participant: No, not that far.

Bassam: They're only 10 minutes away, some of them 15.

Participant: It needs a car.

Riham: It only needs a car is all.

Riham: Did you try to use buses when you first came and didn't have a car?

Bassam: I took the bus once.

Participant: I never did.

Riham: Why?

Participant: Just like I told you, when I came, 25 days were full of housing problems. Then I got pregnant again. And there wasn't—. I didn't, I mean—.

Bassam: And we came [Indistinct]

Participant: And we came to this place.

Bassam: And we didn't take the bus because the organization [Indistinct]

Participant: Yea, I used to walk back and forth from my place to the organization.

Riham: Then, it was really close.

Participant: Yea, about—.

Bassam: [Indistinct] on this street [Indistinct] on the corner, a building [Indistinct]

Participant: I'm talking less than 10 minutes. You walk, and you're there.

Ahmed: [Indistinct]

Riham: Oh, so that's why they put a lot of people there.

Bassam: No, that's not why. I was the first person to come here.

Riham: You were the first?

Participant: Yea.

Riham: So before you all these—. Didn't you say there were refugees from Nepal and—.

Bassam: [Indistinct]

Participant: We don't know when they came.

Bassam: [Indistinct] they were there but [Indistinct]

Participant: We don't mix with them. Only the Syrians.

Bassam: [Indistinct]

Riham: Why don't you mix with them?

Participant: We don't understand them, and they don't understand us.

Riham: How sad

Bassam: [Indistinct]

Riham: With hand gestures. You haven't learned Nepali yet? You already know Turkish and Arabic. Come on, learn Nepali.

Bassam: [Indistinct]

Ahmed: [Indistinct]

Riham: Honestly, that's right.

Bassam: [Indistinct]

Participant: No, I want to learn English. That's the best thing.

Riham: You're going to end up being an interpreter, right?

Participant: No, that's very far off. I have to take, study, and work.

Bassam: A language doesn't come overnight.

Ahmed: It takes time.

Bassam: [Indistinct]

Participant: It takes time.

Riham: Yea, but some people have—

Participant: a desire to learn.

Riham: a desire to learn, and they learn quickly.

Ahmed: Especially kids. They learn the fastest.

Riham: Yea, you see kids—

Ahmed: [Indistinct]

Bassam: [Indistinct]

Riham: speaking fluently after a year [literally “like a nightingale”].

Bassam: [Indistinct] As for me, I hear a word from here and—.

Participant: Then it comes out of there.

Bassam: then I forget it [literally “It flies away.”].

Riham: Do you try to study in—.

Participant: Like, I try to say, “If I learn, I won't need the interpreter between me and the doctor.”

Riham: Good.

Participant: “So he can understand me.” On the days I have to go to the doctor, I have to learn Arabic, not English. I have to learn Arabic, how to speak Arabic, and leave my place.

Riham: Wow.

Participant: So I can explain to her—. So I can explain my situation.

Bassam: Language doesn't come quick. With regards to [her?], it's maybe hard.

Participant: But I want what Bassam talked about.

Bassam: [Indistinct] doesn't learn the language [Indistinct]

Participant: Sometimes I have a desire to learn.

Ahmed: [Indistinct] in the beginning [Indistinct]

Participant: And sometimes I have no desire at all.

Bassam: [Indistinct]

Ahmed: [Indistinct] okay, someone [Indistinct]

Bassam: Okay, I downloaded a lot of programs/apps to help me learn the language. I watch them for five minutes then my head hurts. [Indistinct] to learn a language.

Participant: I don't want to learn what they tell me. [Indistinct] I asked the doctor that, and he told me—.

Riham: Just a minute. [Indistinct] Sorry, do you try to learn—sorry, study—at home with your kids? With their homework or—.

Participant: Teachers used to come over for me. Susan, when she saw the situation—that the organization was punishing me—she said “That's it. I will—. I have three or four friends.” She said “I'll send them over to her.” Everyone would stay for a half hour or an hour more or less if they see me relaxed. When they see me tired, I mean, they go easy on me. They go easy on me. It was once or twice a week.

Riham: Yea, but, for example, Ahmad or Muhammad, because—. When they come home with homework assignments, do you try to sit down with them and learn with them?

Bassam: They don't have any. They don't give them homework.

Riham: They don't have homework?

Participant: They don't give any. They don't seny any work home.

Riham: They're seven years old and don't have homework?

Participant: No.

Bassam: They base that off of knowing that their family doesn't know English. If they [Indistinct] the kid the lesson, neither he nor his parents are going to know—.

Riham: It could be from the school—.

Bassam: They all try to [Indistinct] in school so [Indistinct]

Riham: To maybe help them finish their homework there at school? Oh, okay, that's why. Because that also helps the family. When they work with their kids—.

Participant: And if you work, for example—.

Riham: they help each other.

Participant: and don't know what you're writing.

Riham: No, but you learn together. I mean little by little, not within a day.

Participant: Now, for example, the letters. We sit down, Ahmad, Muhammad, and I, for example.

Riham: Yea, yea, yea, yea.

Participant: My son made the letters into a song.

Riham: Yea, yea.

Participant: My youngest son, Muhammad.

Riham: Yea, yea. Come sing them for us! Muhammad!

Participant: Muhammad, come here and say the alphabet!

Riham: Come and say them. What is it? A worm.

Participant: No, an ant.

One of the Participant's Children [Muhammad?]: No, an ant.

Participant: Okay, cut it out.

Riham: I thought he was going to put it on me. My brother used to always do that.

Time (3B) – 50:00

Participant: Come on, from the beginning.

Riham: Tell it to me.

Muhammad: A, B, C, D—

Participant: No, sit here.

Muhammad: A, B, C, D, E, F, G—.

Riham: Did you memorize them?

Muhammad: H, I—. [Indistinct]

Participant: No, it's okay, honey. Repeat it, let's go. A, B, C, D.

Riham: A, B, C, D.

Muhammad: A, B, C, D, E, F, G, H, I, J, K, L, M, N, O, P—.

Riham: Wow!

Group: Q, R, S, T, U, V, W, X, Y, and Z. Now I know my ABCs. Next time wanchaw—.

Riham: Wow, you're smart!

Participant: From the beginning.

Riham: Okay, now we have to say it in Arabic. Alif, Ba, Ta, Tha, Jim, Ha, Kha.

Participant: This one here really likes to—. Sometime a while ago [Indistinct] he made it into a song.

Participant's Child: [Indistinct]

Sarah: Should Sarah do it?

Riham: Sarah, do you know it, too? Okay, now it's your turn.

Sarah: A, B, C, D, E, F, G, H, I, J, K, L, M, N, O, P, Q, R, S, T, U, V, W, X, Y, and Z.

Group: Wow!

Ahmed: Bravo!

Ahmad: Ahmad!

Riham: Ahmad? Okay, let's go.

One of the Participant's Children: [Indistinct]

Riham: That's in Turkish. Do you know the Turkish alphabet?

One of the Participant's Children: I know it.

Riham: Tell it to me. Honestly, I don't know it, but tell me the song.

One of the Participant's Children: A, B—.

Riham: Do they have a song, too?

One of the Participant's Children: No.

Participant: No, there isn't.

Riham: There isn't a song? There's a song in every language.

Participant: Yea, they—.

Riham: There's not a song.

One of the Participant's Children: A, B—.

Group: S, C, D, K—. [Not in unison]

Participant: E, O—.

One of the Participant's Children: Honestly, I'm getting mixed up. [said in Turkish]

One of the Participant's Children: A, B, S, D, N—.

Bassam: Great! [said in Turkish]

Participant: D, O. It's like—.

Bassam: The kids forgot. They used to have them memorized then forgot.

Ahmed: [Indistinct]

Participant: They're still going to permanently forget them.

Bassam: The same letters as—.

Participant: The same letters as English.

Riham: Yea, it seems that they're the same ones.

Participant's Child: They call the letter “E” “A.”

Participant: I mix up the letters more than anything else.

Bassam: As for you [Indistinct] English—.

Riham: Which one? “A?”

Participant: You have “A.” I go and tell them “aa.”

Riham: Oh.

Participant: She tells me “No.”

Riham: In Turkish.

Participant: Yea, I say it in Turkish.

Bassam: The Turkish language [Indistinct] has some Arabic—.

Ahmed: It has some Arabic?

Riham: Do you have some other questions or should I shut it off?

Ahmed: I don't have any more questions.

Riham: Okay, thank you very much.

Ahmed: Thank you very much.

Riham: We're going to shut it off.

Participant: [Indistinct]

Interview 4A

Riham: Today is May 13, 2017. I am Riham and I am with a father of six children. His number is 04A. Okay, how are you?

Participant: I am doing well, thank God.

Riham: Thank God. We spoke a little before and got to know one another but we're going to start from the beginning and I am going to ask you some questions.

Participant: Okay.

Riham: Okay. Tell me a little about your impression of the medical system here.

Participant: The medical system here, as I told you earlier, is good. The doctors are good and the treatment and care is also good. The doctors are very good, and the treatment is good. The only issue is with our appointments. Since we are refugees and do not know the language, we are missing a lot of our appointments. The person in charge of us at the organization [Catholic Charities] should be helping us with this. The caseworker, who is supposed to inform us and arrange for these appointments, is not letting us know. We're missing a lot of appointments. We do not know Ohio, and we are missing appointments.

Riham: When you ask them about your appointment is, what do they tell you?

Participant: I don't ask – I don’t see this caseworker.

Riham: As in how do you know you are missing these appointments?

Participant: By mail. We are receiving letters that inform us when we miss an appointment. Or after a while, we would receive a phone call from them asking why we missed our appointment.

Riham: Oh, I see. So you wouldn't even know about these appointments to begin with.

Participant: I would have no idea I had an appointment! Imagine you were speaking to me in English. I would answer the phone and hear English and then I would hang up. Then we end up missing the appointment.

Riham: Okay.

Participant: How am I supposed to know it was the hospital or clinic or what not calling? I don't know.

Riham: You have six children, right? May God protect them for you. When you first arrived, they are supposed to take you to get a screening exam. Did that happen to you?

Participant: Yes, right when we arrived they took us to get vaccinated. When we first arrived, they took care of us. They would come to our house to pick us up and bring us back home. Take us and bring us back.

Riham: Okay.

Participant: They would take us and bring us back home. After we got our vaccines and we spent three months with the organization as you know, we were told that we were on our own.

Riham: During those three months you weren’t told which doctor to go to yourself and your children?

Participant: At first they used to make the appointments for us. They did that for us maybe once then they stopped. They also found out we had received a car so they just left us on our own. They said, “You are on your won. You figure out your own appointments and how to get there.” Everything, everything is you.

Riham: You are having trouble making appointments though.

Participant: It's clear. If I wanted to go to Children's alone or even with my wife, we will manage. If I am going for [indistinct] I'll need to call a friend or someone to help me. I will need to ask for help. I cannot speak [indistinct].

Riham: When you call your doctor, do they give you the option to choose Arabic as a language before they connect you?

Participant: No.

Riham: Or maybe provide an interpreter.

Participant: If they call me and I understand that the message is for me regarding my appointment I simply tell them, “no English, speak Arabic”. Sometimes they will have an interpreter call. They rarely have an interpreter call me though. It’s really important they do.

Riham: Do you feel like there is a difference between the system for the children and the one for adults?

Participant: Yeah, it’s clear.

Riham: Do you feel like one uses interpreters more than the other?

Participant: Yeah, it’s clear. We have appointments for the Children’s, for example. My wife and I don’t have any. We keep missing them. If we don’t have someone that carries some of our burdens [Arabic phrase]--. God always sends good people your way. There is this volunteer that comes and takes our names and makes our appointments. If it weren’t for her help we would have missed out on even more appointments! Many many appointments!

Riham: Do you currently have any medical problems?

Participant: Yeah. I have diabetes, hypertension, hypocholesteremia and everything.

Riham: Okay.

Participant: A new issue that just started is the infection in my *kidneys. From the work that I was telling you about earlier with the refrigerator.

Riham: Yeah, tell me a little about your job.

Participant: In terms of work they are giving us jobs that our beyond our capabilities. I worked as a plumber before as I told you. I am a trained plumber but they gave me a job with vegetables. It’s a job for 20 year olds. I am 50 years old and working a job of a 20 year old. It requires heavy lifting and its cold and really really hard. More than you can imagine, it is hard. At first I worked at Club Chef [indistinct]. The first month or two it was fine. The third month the chlorine had an effect on me and I spent it all with doctors. Then I quit. I told them to find me a job elsewhere. They said no, “it’s either this place, or no job at all”.

Riham: Why?

Participant: Just like that. Because they say that I had the abilities to work in this store. But I started getting harmed by it.

Riham: Yeah.

Participant: The smell of onions and the chlorine started negatively affecting me. Ten minutes would not pass without me coughing. Then I got bronchitis.

Riham: What do you mean?

Participant: It’s an infection in the chest.

Riham: Oh so an infection in the lungs.

Participant: Exactly. I am still working even with all this. It really doesn’t suit me. They tell me I need to know English for any other job I want. You need language. Language. language. And you know how my English is…

Riham: Yeah.

Participant: Yeah and something happened with us at the school with the organization I mentioned to you. Back home we were illiterate. We did not go to school. For them to teach us English in a night or day is not going to happen. They keep teaching us sentences and words from here and there. We told them we did not even receive any education back home so you should start with the letters. You have to start with the basics. It goes in one ear and out the other. Nothing we learned would stick. From interacting with people, we learned a word or two. We can get around a bit. We socialize. We figure out things.

Riham: Thank God. Okay so in your opinion, one of the biggest problems is the interpreters.

Participant: The interpreters and missing my appointments. I am missing a lot of them. Yesterday I had an appointment. My wife told me that I missed it. No one called or reminded me. They did gave me a paper. Yes. But a person has a lot on his mind. They aren’t going to remember this piece of paper.

Riham: In Syria, how would they remind you about your appointments?

Participant: We don’t have this. If we have an emergency we go immediately to the emergency room and they take care of you right away. They give you the medicine suitable for what you are suffering from. For example, if you have bronchitis, they will give you medicine specifically for bronchitis. They won’t give you a pain relievers just to ease the symptoms. Here they just give you pain relievers all the time. You just suppress the illness over and over again and then it becomes permanent. You get full of disease eventually. Like what happened with my daughter whom I told you about yesterday.

Riham: So in Syria, you don't have any appointments?

Participant: No. If you want to go see the doctor you would just call and ask if they are open and available. If so you just go on that same day.

Riham: The same day.

Participant: Yeah. If you’re going to get treated, with a specific problem.

Riham: Okay.

Participant: It’s not like here.

Riham: Yeah, it’s not like here at all.

Participant: No, no, no.

Participant's Wife: There if you want to see a doctor you go on that same day. If you have an emergency you go to the emergency room right away and are seen.

Participant: Here it’s trial and error.

Riham: I am sorry, could you explain further?

Participant: It’s trial and error. [indistinct] They don’t give you the final treatment.

Riham: Here they really care about not diagnosing you with anything until they are 100% sure.

Participant: But they don’t give you the medicine and treatment needed for your situation.

Riham: Yeah.

Participant's Wife: He is speaking in general.

Riham: Explain what you mean by the right medicine is not given. I know we spoke a bit yesterday but please repeat your thoughts now.

Participant: Let’s say I have diabetes or even high cholesterol. If I go see a doctor he will give me something to ease the symptoms or the pain. He won’t give me something to cure the illness completely. Do you know what I mean?

Riham: But diabetes cannot be cured, for example.

Participant: It’s just an example I am telling you. It doesn’t have to be diabetes. Any illness. For example, my daughter and her [indistinct]. If they had only given her the exact treatment right away…

Riham: I am sorry, her what?

Participant: Migraine.

Riham: Oh, a migraine. Tell me the story from the beginning because I am not familiar with it.

Participant: My daughter got this illness. It started off with pain in her stomach. We took her to the doctor, and he could not diagnose the illness 100% and give us the medicine that's made to treat this illness. He gave her a pain reliever. It made no difference. The pain kept coming back over and over after the medicine would wear off. Now the disease has set in her body more and more. It's 60% now in her intestines [or gut]. It increased and grew stronger because they did not give her the proper medicine from the very beginning.

Riham: Perhaps they didn’t know what the illness was.

Participant's Wife: She ended up having an infection in her intestines [or gut]. Instead of giving her medicine to treat the infection they gave her pain relievers. The infection grew and grew in her body.

Participant: The disease spread! It is more than 60% now. Isn’t that a disservice to the girl. Yesterday I had to go pick her up from school because they called and said she wasn’t feeling well.

Riham: How old is your daughter?

Participant: She is 20 years old.

Riham: Is this Crohn's?

Participant: Yes, Crohn's.

Riham: She didn’t have this medical problem in Syria?

Participant's Wife: No.

Participant: No, it's new. She did not use to complain of this before.

Participant's Wife: I never had to take her to the doctor before.

Participant: In our country, we can even use herbs as medicine. We use this or that. We treat them with herbs. Here they don't. They don’t know.

Riham: Did you try any of those therapies that you were using in Syria, here?

Participant: Yeah we bring it and use it for like a cold or what not. It’s better than sixty medicines.

Riham: Are there any other remedies you use at home before going to the hospital or --.

Participant: Yeah. For example, if someone has a fever. You measure how high it is. If it’s high you can take care of it at home by putting something on the head. There is no need for the emergency room or the hospital. This thing, it’s a light matter. If it’s something serious then you need to take them in.

Riham: What is --.

Participant: My son Mohamad-nur had a fever of 102F. His mother had to take him to the hospital. Do you know what I mean?

Riham: Yes.

Participant: It depends on what the disease is bad, if it's serious or dangerous, then you need to take them in to the emergency room.

Riham: Okay so then tell me what you consider to be a serious illness. From your viewpoint.

Participant: When it comes to a child it’s hard to tell what is serious and what is not. It’s all serious when it comes to a child. You don’t know what he’s dealing with. My son had a fever. From what? It turns out my son had an infection. Of his tonsils and his throat. It caused the fever.

Riham: Okay so a fever. Is there any other illness or situation that causes you to go to the ER?

Participant: Yes. My daughter Asma, sometimes loses consciousness. I am not able to pick her up. I need to call the ambulance to come.

Riham: Does this still happen to her?

Participant: Sometimes. It’s been a while now, thank God, since the last time it happened. It’s been over two months, thank God.

Riham: Thank God.

Participant: Yeah, something like that.

Riham: We have some pains that physically affect the body and some pains are mental or emotional. How do you teach your kids to cope with pains that may affect them mentally or emotionally?

Participant: From the day I had my children till now, I have not experienced anything like what I am experiencing now in this foreign country. My wife has been with them all the time. I just go to visit them every now and then. I would come and go. My whole life has been in a foreign land.

Riham: Why?

Participant: From the 90s…I come from a very poor family. They are only supported by God and myself. I had to do it to support my family. My wife would be in Jisr [Jisr al Shughur, city in Syria], and I was in the West. Back home there wasn’t a job around that we wanted enough to support my family. I had to work in Lebanon and come back to Jisr.

Riham: Okay let me see if I understood you correctly. You are saying that you go and work in a different country and then come back to your family.

Participant: Yeah, just to visit. I would work in Lebanon and then come back to Syria to visit.

Riham: Oh okay. So you have been doing that from before.

Participant: Yeah from the 90’s. I have been in Lebanon since then.

Riham: Okay I understand you now.

Participant: My family isn’t in a good financial state. I had to support them. I have to prepare for their futures, whoever needs to get married and start their own families. Whether it be my sick father, my family, or my sick mother, I need to support them. All of this was demanded of me. Thank God. But God helps us.

Riham: Thank God. In order to care for your health and the health of your children, what are some things you do here in America?

Participant: I am trying. I am trying to land a comfortable job for myself. One that is easy and suitable for me and my age. In order to support my kids and not have them dependent on anyone else. I also don’t want to be dependent on anyone. My only wish from this world is for God to send me a good job to support my family because we migrated here for the sake of our children. We wanted to give them a good life and education. We broke our backs just for them. We used to live in Lebanon and everything was great. Their mom was working and I was working and we weren't in need of anything, thank God. What broke our backs was their education. We don’t only want one or two to get an education. As much as one worked back home, education is expensive. We wanted all six of them to get an education. This is what caused us to come here.

Riham: May God send you what’s good.

Participant: Amen.

Riham: It seems like you really care about your children and your whole life has been dedicated to enhancing theirs.

Participant: It's not just to enhance the lives of my kids. My parents are my whole world. There is nothing more valuable than a mother and father in this world. Then comes my family. If one loses a mother or a father, no one can ever replace them. A child can be replaced though.

Riham: [laughs] May God forgive you.

Participant: If a brother is gone, no one can replace him. You can have another child, though!

Riham: A child can be replaced?

Participant's Wife: As in you can just have more kids.

Riham: I understood what you are saying. I was just joking with you. In your opinion do you think you can secure the things your kids need in order for them be successful in this country?

Participant: There are many things we wish to secure for them. Let's say that God willing, we will. But. Since we are new here it's not the case yet.

Riham: What are those things you wish for? Tell me.

Participant: My wish. My wish is their education for one. We want them to get educated and become like you. If any one child has any passions or dreams, I want them to realize them. This one right here wants to become a doctor. I have to do my part to contribute to that and the rest I will leave to God. We will do what we possibly can to help them. We leave what we cannot do, to God.

Riham: Thank God. In order for your daughter to become a doctor and your son to become what he wants, what are you in need of?

Participant: I need a job to be able to get money to financially support them. They have to overcome a lot of obstacles to reach their dreams. Money does not come easy. One needs to strive for it. The type of work they throw you in here is –all of their work… It's as if you are worthless and they say “these people are coming from behind the cows.” For us, every person can become a "doctor or a Sultan" [and expert] in their work as they say. If they throw me in a job that is not within my area of expertise it's --. I come home unable to stand on my feet. My feet are tingling like bugs. Especially since I have diabetes and several other illnesses. On top of that everyone thinks they're better than you at work. Those that are and those that are not. This is what tears a person apart. Do you know what I mean?

Riham: Yeah.

Participant: That's what's happening to us here. It's because we are new here. Hopefully, when we spend more time here and adjust, I am sure we will feel more at ease. By that time one can learn the language and when God sends a person a job that is suitable for them. Then we'll feel at ease.

Riham: How long have you been here?

Participant: We have been here for 11 months.

Riham: That’s almost a year. Same as me then. Yeah, we both came in July.

Participant's Wife: June.

Riham: I came in July and you came in June.

Participant: Yeah, we came in June. We have been here for 11 months.

Riham: You were telling me about a lot of things that have hurt your head [troubled you]. From what I know about Syria, the war and the difficulties that came from it, you may have experienced a lot of stressors from the past and now the present. Can you tell me what you do to cope and take care of your mental health?

Participant: My mental health? My mental health is constantly tired. I am not doing well psychologically. My mental health? It betrayed me to want to go on top of this building and jump off. From all the pressure I was feeling.

Riham: When did you feel this way?

Participant: About 4 or 5 months ago.

Participant's Wife: In September.

Participant: Yes, in September.

Riham: What happened during that time?

Participant: We felt a lot of pressure, stress. The charity organization left us on our own. They cut off all our benefits. They were supposed to return $800 from the house to us but they didn't. They ate it [stole it].We called someone for help, and he wouldn't answer. We have no one to turn to other than God. We are new here. If they don't help us, then who will? For example, if you are responsible for us we are going to complain to you. Who will meet our needs other than you? The ones that are responsible for us do not answer our phone calls even if we call 20 times. If we go to them they refer to others and say maybe they can help you. Why if you are receiving a salary and are assigned to us, why are you sending us away? Why are we any different than others who are asking them for help? If this person is a human and that one is a human. One is educated and one is not. Why would that one that is educated suddenly become better? It shouldn't be like that. Everyone should be treated equally, like two teeth of a comb. They should treat refugees the way they would treat their own people. We just want them to give these kids what they deserve. To give them what they need and not put them in a situation where they are dependent on anyone. A refugee in every other place is treated better than we are treated here. We left our homes, our families, and our world. Nothing is left for us….A refugee here should be taken care, treated well, and valued. Everyone should take all this into consideration when dealing with a refugee.

Riham: What are some things that made you feel like you were not honored here.

Participant: Being abandoned. When a person feels like they are abandoned, it makes him lose it. When you make me pay a security deposit and I leave the house, shouldn’t I get my deposit of $800 back? What would you do with yourself? Wouldn’t this stress you out?

Riham: Of course.

Participant: Stress topped with more stress. That's what causes one to explode. I told the charity organization all this. They cut off all my cash benefits. If my father asks me for a $1000, I don't have anything to give him. What will I do? If my child even asks me for a dollar, I don't even have that to give him. How do you think that makes me feel in front of my son? When I was in Lebanon, if my father were to ask me for 1000, I would give him 5,000. We didn't come here to be treated this way. We are being treated unjustly here. In our whole lives, no one treated us this way. I have never wronged my kids when I was far away from them, nor was I ever mistreated. In Syria. In Lebanon. No matter where we went our situation was good. As soon as we came here we started to feel this stress. I told them I am going to jump off a building. That is when they sent me some psychological help. Sign a paper about not hurting yourself or your wife or your kids. They raised our hopes that they were going to help us again then they deceived us.

Riham: What do you mean?

Participant: They promised to help with the rent. They would pay half the rent. They did not give us the $800 nor help us with the rent. Not even for one month. These are the types of stressors we are dealing with.

Riham: When they sent you the --. I am sorry what was it that you used to describe the psychological help you received?

Participant: A social worker

Riham: Oh you meant social worker.

Participant: Or a psychologist.

Riham: Okay, did you feel secure and at ease when you were able to talk to the psychologist? Did you speak to them like every week?

Participant: Every week? Who is there to answer your phone calls every week?

Riham: So they did not refer you to a psychologist? Appointments?

Participant: They just put me in a room and did not let me leave till we were in agreement with me not harming myself or my family. All they did was raise our hopes. We felt like we trusted them. We thought they would help us after that. They didn't of course.

Riham: I work in the emergency room so let me tell you what happens when someone comes in who is in the same situation as you. They would sign this contract and receive a referral to speak to a therapist every single week or every other week.

Participant: No no no no no. A person as much as they may speak they might not necessarily harm themselves. They might say they would but they won’t actually do it.

Riham: Some people do.

Participant: Maybe if they lost their mind. Sometimes a person just talks and talks…and they won’t actually harm themselves.

Riham: Let me just tell you that I see all kinds of things. Yes, it is possible for one to harm themselves. That's why we take it seriously.

Participant: I also see all kinds of things. I saw all kinds of things happen right in front of my eyes. I understand. I will not harm myself though. I have children. If I harmed myself or even killed myself the children will be in a worse state.

Riham: They didn’t give you a referral to a program?

Participant: No

Riham: Nothing at all? Just that time that they spent with you?

Participant: That’s all. Then they signed a paper and did not even give it to me.

Riham: So --.

Participant: All they do is deceive the refugees here just so they can gain money. That sums it all up.

Riham: I am stressing on this point because --. It’s okay leave the window open. I was actually going to ask you if we could sit outside since I don’t get to see the sun as much.

Participant: Sure we can sit in the back, it’s not a problem.

Riham: No no, it’s okay. So, I am focusing on this point because I want to know how we as doctors can help a refugee. A refugee might experience psychological problems different than that of a person who has been raised here.

Participant: Of course. If someone leaves a war zone, are they going to be comfortable mentally? No, they are not going to be in a good mental state. Our families are all scattered. You won’t even find two family members in one place. Every single one of us ended up in a different country or city. Our mental health is going to be ok? No. This will certainly impact a person’s mental state.

Riham: Yeah.

Participant: It's been destroyed in ways you can't even imagine. When we were in Lebanon, at least we had our family members around us. Then we all got separated. How is our mental state going to be? On top of that, we have all this stress. It's good enough that we are standing on our feet. Also, the Syrian community here, thank God, keeps telling you that it's okay. They calm you down.

Riham: What do you mean by that?

Participant: As in they remind you to cool down and not get so worked up. They tell you just hold on to your faith, be with God. The Syrian community here? There is nothing better than the Syrian community here in the world.

Riham: That means you are finding that the community strengthens you.

Participant: May God reward them. They are trying to get us everything we need. Of course, they can't possibly provide everything. Some things are out of their hands. Some are also doctors, but they still may have their hands tied. Whoever is able to, they are helping. Making things easy for us. If it goes beyond their capabilities then, of course, they cannot help.

Riham: Of course. You have been here for almost a year, right?

Participant: Yeah.

Riham: Is there a solution you can think of that may have made it easier on you when you first arrived? A system that would have helped refugees access healthcare or meet their needs? I know that the hardest time for a refugee is when they first arrive.

Participant: A refugee. A refugee needs to feel from the moment they arrive. A refugee needs to feel like they are not left on their own. That they are taken care of so that they do not feel like refugees. So that they do not go into depression. They cannot just throw a person in --. We did not see anyone at first. They threw us in this house, just like one would put an animal in a cage. They locked us in this house and it wasn’t till after about three weeks that they started taking us around. To the community.

Riham: Three weeks?

Participant: Yes, three weeks.

Riham: How did you buy food?

Participant: They gave us some money. After they vaccinated us they gave us some money in our pockets. We learned about a “mall.” We learned how to get groceries. Did anyone stop by to check on us? No. It wasn’t until after three weeks until they took us to the organization. When a refugee first arrives you should not let them feel like they are refugees. They already endured a lot of stress. They don’t need any more. They should really take care of them. We were so happy after meeting the Syrian community here. They did not leave us alone. They take us out every week or two. They invited out to parks, to restaurants, take us out. When we first arrived the organization did not even tell us there was a Syrian community here! They don’t even inform them about us.

Riham: Okay. That means if they had informed both sides it would have improved your experience. So if a new family comes and they inform them that other Syrians are here, then it will ease their transition.

Participant: Yeah, of course!

Riham: Us as Arabs --.

Participant: We need one another. We need each other! Now the new refugee families that are coming do not feel like they are refugees. Why? Because now every time a family comes we get a message on the group from the leader of it, informing us and asking if anyone wants to go and greet them or check up on them. When we first arrived, we honestly did not see anyone for 15 days. No 3 weeks! On top of that, the house is filled with insects and animals. Around the house not in the house. There was a balcony. We had a raccoon living in front of the house under the porch. My son woke up at night to use the bathroom, he went in and saw a raccoon outside the window. He was so scared! This one [his daughter] was also hanging up the clothes to dry and she saw a "hanash."

Riham: What's a "hanash?"

Participant: A black snake.

Riham: Oh no!

Participant: We call it a "hanash." We were sitting to eat one day and looked out this window. There was a plant and inside we saw three snakes coming out.

Riham: Oh my!

Participant: Next to the house, next to the house, there was ghreer. It’s like a rabbit but with a long tail. Making a nest. “Abu Basil”…like a rat, would be climbing all around. We couldn’t walk in the street. My kids did not like leaving the house without me. If I wasn’t there, they would not walk outside. They were so so scared.

Riham: Why not?

Participant: Because of the snakes and the raccoons.

Riham: So out of fear.

Participant: Yeah there is fear from both sides. Fear of the neighborhood they placed us in. Filled with drug dealers and God knows what. That is the biggest fear. They used to deal with drugs there. The man had a lawnmower. The neighbor. So my friend came to visit and asked him to mow the lawn and he would pay him. But my friend came and told us they are drug dealers. We had no idea till our friend came and told us. The neighborhood was a dirty area. From both sides. The social environment and the animals to worry about. We had pressure from both sides.

Riham: In your opinion, you think that all this stress is what caused your daughter to faint?

Participant: Yeah.

Riham: Tell me a little about that.

Participant: Imagine you have an animal that is used to going out and coming back in as it pleases. If after that you place it in a cage and do not allow it to go, what will it do with itself? It will do what it can to get out of this cage. We are keeping our kids in the house. Restrained. In Lebanon, they used to go out in front of the house to sit or play. They would go out to visit their grandpa. We would never sit at home on the weekends! We would always go somewhere. To the park. Here they came felt restrained at home. All the pressure, stress and depression caused that to happen to her.

Riham: After all this happened to her, were you able to find out from the doctor why this happened?

Participant: We did not get anything good or bad from them. After the third time of going, they finally said it is a migraine. It took three visits or four visits for them to diagnose her with a migraine. They did not say it was psychological. They just said it was a migraine. Where would this migraine come from? She didn't have it before. Where would it suddenly come from? Isn't from psychological stress?

Riham: Okay.

Participant: She used to come and go all the time in Lebanon. She would go walking… for long distances with her siblings. They would go to learn the language. She was perfectly fine. She didn't get dizzy or anything. When we came here, this illness started.

Riham: What are some things you and your wife did to help her cope with her emotional stress and prevent this incident from happening again?

Participant: We first tried to move. From the very first month in it, we told them we do not want to stay there. From the first month! Especially when we saw what it did to our kids and the animal situation. From the first month. They forced us to stay because we signed a contract before coming to America that we would stay there for three months. What did I do wrong? Why do we deserve this? …My kids were suffering emotionally. They sent us animal control to come and take care of the raccoons and Abu Basil. Every day they would catch two or three of them. The traps kept going off all day. A trap here. A trap there.

Riham: You could have opened a zoo. [laughter]

Participant: Yes, honestly. They ended up catching six raccoons! Six of them!

Riham: You should really open a zoo and charged an entrance fee.

Participant: The last thing I need is more animals. It’s enough we had those animals over there back home — [laughter]

.

Riham: Okay, so you moved to a new house. You did that for the sake of your daughter’s well being.

Participant: Not just for my daughter. For all of them. They started all falling sick one by one.

Riham: What illnesses did your other children have?

Participant: First it was Asma then it was Amal. [indistinct]

Riham: Did you consider going to a gym or to a park? Did you have one near that house?

Participant: No. At the verrrrry end of our stay there my son walked around and said he found a park. Everyone one of my kids became happy. He started taking them to the park. This relaxed them a bit. It improved their moods. This happened a short while before coming here. Perhaps it was a month before we moved here that he found this park. It helped their psychological state a bit. A little bit. Maybe 1% better. By that time my daughter Asma had already developed this illness.

Riham: If you were told about the local parks and places that you could go to with your family when you first arrived, would that have helped?

Participant: It doesn’t matter how many places they tell us about. The house had animals. What benefit would we have gotten from knowing about these places? If we went out and they had a good time but then returned to fear in their home, what’s the point?

Riham: It’s clear that the living situation wasn’t healthy.

Participant: It’s depressing in and of itself.

Riham: I am trying to help us focus on the things that we can change. What tools can I provide for you as a father in order for you to be able to help improve your children’s mental health? Do you understand what I mean?

Participant: Yeah.

Riham: The schools, for example. Tell me some ideas.

Participant: The first thing I would need is to have an ease of mind. I need that to be able to help my kids. After that, it is going to good schools.

Riham: Is anyone bothering your kids at school?

Participant: No, they are not. At Withrow, they are saying that there are issues between the students. Emotional and verbal abuse. There were some egging that happened in the homes. It was on the news.

Riham: Oh no.

Participant: We want our kids to feel at ease. If the kids are at ease, then the parents will be at ease both physically and mentally. If, however, we see that our kids are unhappy and that they go to school as if they are going to a funeral, we will not feel at ease! The mother won’t feel ease. The father won’t feel ease… At the end of the day whoever has money makes it in this country. If you have money then you can get by. If not, then you cannot.

Riham: That’s in any country, right?

Participant: Yeah, but it is different in this country. The expenses are a lot here. And it is different. The landlord here will not wait for you to get the rent. If you don’t pay by the 10th, you get evicted.

Riham: It’s a different system then.

Participant: It’s a different system.

Riham: It’s different than our countries.

Participant: In our country, if a month comes and you are able to pay then you do. If another month comes and you are not able to pay then the landlord will be patient with you unlike here.

Riham: There is no trust here.

Participant: No there is no trust. As much as they say there is safety, safety, safety, it's bound to not be safe. To have problems. Robbery. Thievery. I still take the precaution of everything. From the number of people that have told me about houses being robbed, I decided to carry a weapon. I do it to protect myself and my family. I am looking for a weapon.

Riham: A gun?

Participant: Yeah. If a burglar were to come in, he will not come in unarmed.

Riham: Of course.

Participant: Let me tell you. My friend has “three on his neck”.

Riham: What does that mean?

Participant: It means he was attacked by three people and he killed all three in self-defense. If he wasn't carrying, it would have ended differently!

Riham: Okay.

Participant: Do you see what I am saying? A person needs to protect himself and his family. In this country specifically.

Riham: So you feel that in this country, even more than in Lebanon, you need to protect yourself?

Participant: In Lebanon, it is an Arabic speaking country. You also know your friend from your enemy. You can’t talk to anyone here. Here you cannot because of the language. You will not know who your enemy is and who your friend is. Here you don’t know anyone.

Riham: That’s really hard.

Participant: By the time you acclimate to the society, learn more, the person who would have hit would have run and the person who ran hit [Arabic saying implying chaos]….[long pause] You experience a lot of trial and error. That’s how it is. A person needs to turn to God during this time.

Riham: I hear you often saying this like you will turn to God and your relationship with God. Tell me how your faith has helped you during these times.

Participant: Of course. It definitely puts one at ease. When times are tough we have prayer and we have the Quran. It eases the mind and soul. A person gets up, prays two Rukkah’s. His mind is at ease. Our religion helps us with a lot of things!

Riham: In your opinion, has your faith helped you in the past or is it playing a bigger role now?

Participant: Our faith us been helping us from the time we were born.

Riham: Thank God.

Participant: Look, there is nothing better than faith.

Riham: No there is isn’t.

Participant's Wife: The first and foremost thing it helps with is patience.

Participant: “Be patient and remind others to be patient” [Verse from the Quran]. Patience opens doors for ease. We experienced what no one has ever experienced but we are being patient. God will surely compensate us. He has to make things easy. Our hopes in God are high. “Whoever fears God, He will make a way of out for him” [verse from the Quran]. We are relying on God. No matter how much a servant does for God, he will not even come close to doing what God does for him.

Riham: So you are finding that relying on God is helping your mental health?

Participant: Of course. We rely on God every hour of every day. Whenever you leave your house you rely on God. Pleasing God and you also please your parents. If God is pleased with you then --. Every time you leave the house your focus should be on either pleasing your parents or pleasing God. Your parents being pleased with you is a sign that Allah is pleased with you.

Riham: May God be pleased with us.

Participant: We talk and talk and talk, but I wish we could give him a bit of what He has given us.

Riham: It’s not in our hands.

Participant: Do not say it is not in our hands. Everything is in our hands. We saw the worlds that’s what happened. We have ears and eyes --. We have many shortcomings though. No one is perfect. We have many shortcomings with God maybe that is why this is happening to us. God is everything good. There is no imperfection that comes from Him.

Riham: Mashallah. This is something you should be proud of -- your faith. Your religion and your faith are helping you a lot.

Participant: Thank God. How can one not be proud of their religion? No matter how wrong a person is and how much he sins, their religion comes above everything else. Am I a prophet? No, I am not a prophet. I do a lot wrong, but I know my religion. I know that my religion is the truth. I know that there is a God.

Riham: Do you feel like a foreigner here? I was telling you how I am from a city that has many Arabs so sometimes I feel like a foreigner here. I am American and I feel this way. How about you? 53:10

Participant: From the minute the plane departed from Lebanon I started to feel like a foreigner. The whole time the plane was in the air, my tears did not stop flowing. It was because I knew where I was going. I was going to a land of a different faith. When the plane was flying…Where am I going. Where I am going..We know that God only has good in store for us. If He did not wish us good, He would not have brought us here. We have to leave it all to God.

Riham: Did it take you a while to find the Arab community? After three weeks of being here, we ran into a woman from Homs. In the school. We saw her like we were seeing a ghost. My wife and her looked at each other. She asked my wife if she was from Syria and my wife asked her if she was from Syria and they both said yes. They hugged and kissed one another. We have not seen her before.

Participant's Wife: I cried so much. It was as if I saw my mother.

Participant: We invited them over. They asked for our address. We didn't hear from them. Then on Sunday, they came over. When we first met her it was a Wednesday.

Riham: Oh they weren’t refugees, they were American.

Participant: No, they were refugees! They were here before we came.

Riham: Excuse me. Just so I can better understand, did the charity organization not introduce the refugees to one another?

Participant: No, no, no. Not at all. It was by coincidence that we ran into her. We were not introduced to anyone before that.

Participant's Wife: She was attending the English lesson. I looked up and saw someone with a scarf on. She asked if I was from Syria and I said yes. She then asked when we came. I told her we just arrived recently. She embraced me and kissed me.

Participant: So we just met each other there by chance. In the organization. It was the work of God. He sent her our way. On Sunday they finally came over. They came bringing goods with them. Then a friendship formed between us and we started visiting one another frequently. Ramadan then started and we invited them over. Then they invited us. Slowly we started meeting the rest of the Syrian community. They introduced us to them. After a while --.

Riham: That’s great. You should give me their names for my study if you are comfortable.

Participant: Yes. Yes. After a while, they introduced us to the community that had been here for a long time. The community then started to introduce us to the doctors in Cincinnati. They started inviting us to their homes. We stopped staying at home. Every few days a doctor would invite us to a restaurant. We started to get some fresh air. When we start to see other people that share the same country, the same faith, we get united. I started to become more comfortable. Then they brought us a car, may God reward them.

Riham: Did you know how to drive from before?

Participant: No.

Riham: You learned here?

Participant: Yes, here. I got my license here and thank God the Syrian community brought us a --. Are they called Syrian American?

Riham: Yes, Syrian American Foundation and Rahma.

Participant: They are the ones that brought us a car and helped us get our driver license. They helped us a lot, thank God.

Riham: Thank God.

Participant's Wife: They came and saw the state we were in. They said --.

Participant: It was like when we first saw the Syrians. It was like you were fishing, and all of a sudden you caught a great fish….Now things are different. A refugee does not feel isolated when he comes anymore. They are meeting them soon after they arrive. We do not want to let the other refugees to feel like they are in a foreign place. There was a guy and his wife and he said he did not feel like he was in a foreign place when he came here unlike his experience in Jordan. This was we feel a sense of belonging more. Before you came…we were gathering together, planning out, we want to all go to a park. Of course, we lost our families but they are kind of becoming our family.

Riham: People appreciate this sense of brotherhood. In my own family, at first it was just my dad and his brother that came here to study. They've been here for around 40 years. They have been here for a while but at the same time, we also have a huge community. But it did not start like that. People were coming from all over without their parents or siblings.

Participant: They came as students.

Riham: Some came as students. You are like a second generation of migrants so you will hold on to one another and create a community like that. I grew up without my cousins or other relatives. I did not meet them till I was around 25 years old. I only visited Syria one time. Here our friends, family friends, are like our siblings.

Participant’s wife: There’s another family. Their story is very sad. I will give you their information to reach them too.

Participant: They stopped bringing as many refugees now. Thank God we are the only refugee family that came here and does not have a disabled child. A lot of them that came here it was because of a disabled child. One had kidney failure and one a stroke. Some had heart problems. Almost everyone else that came here it was due to a medical reason. There isn’t one now…that isn’t coming without medical problems.

Riham: For a medical reason.

Participant: Yeah, as you said. Thank God.

Riham: Yeah this is likely purposeful. People with medical problems get expedited.

Participant's Wife: For a specific condition.

Riham: Yes, a specific condition. It would be a condition that the doctors here might specialize in. When I speak to the charity organization, they are not quite prepared for this population. They did not take this into account. Because from before it wasn’t the case. Things are different now so the needs have changed. The medical problems were not as common as they are now. The reason you are feeling this difficulty, I think, maybe because the charity organization is not prepared to deal with the issues you have come with. We can work together to come up with a solution for the problems refugee families are facing.

Participant's Wife: They do not have the tools needed to meet all the needs of the refugees.

Riham: Yes, but not all the needs. They may not able to meet all the needs but at least the transportation, the doctors --.

Participant: What do you want better than the cars they provided us? Instead of helping with the transportation they are giving out cars.

Riham: That is Rahma.

Participant: No, this woman wasn’t from Rahma.

Riham: I am talking about the refugee resettlement agency, Catholic Charities.

Participant: Oh, Catholic Charities. Just as Participant 2A told you yesterday, Catholic Charities just wants to put you in the mouth of a cannon and throw you in the work field. To the work. To the work. To the work. They are trying to push you out of the organization so they can welcome someone else after you….They don't teach you well. I am telling you they did not even teach me the ABC's! I have been here for 11 months and I do not know the English alphabet. They taught us them in the first two months and they weren't good lessons. We are not typewriters! We told them we were illiterate in our own country. We don't know how to read and write, and we are refugees that are dealing with a great amount of stress. In our own country, they were telling us that we would receive help for a whole year. They were supposed to take care of us for an entire year. That is why we were shocked that they left us on our own after three months. Put a boot in our behinds. If we knew? No one would come here if they knew this. Nobody would come. You put us in a situation…and You raise our hopes then we come here and see that the reality is much different than we expected.

Riham: So you feel like they were withholding information to you before they brought you here?

Participant: Yes!

Riham: Do you feel like you are being taken advantage of?

Participant: Yes, we are being taken advantage of. The people are taking advantage of us.

Riham: They are taking advantage of you?

Participant: They wanted to kick us out of Lebanon because they have a lot of Syrians there. This Shiite there do not like the Sunni. They want them out of there as much as possible. We came here and also --.

Riham: Did you stay in a refugee camp there?

Participant: No, thank God.

Riham: Oh, because as you told me before, you were in Lebanon already?

Participant: Yes, I was already there. I was there from the 90’s. I left the army in the 90’s and then went to Lebanon.

Riham: That is why your accent is somewhat Lebanese.

Participant: It's a mix of Lebanese and Aleppo and Jisri!

Riham: I have Lebanese friends. I feel like a Lebanese person is speaking to me right now.

Participant: Yeah it’s a mix.

Riham: A mix of Lebanese and Aleppo.

Participant: No matter what…as time passes..everyone always goes back to what they grew up on.

Riham: Of course.

Participant: I lived in Lebanon for about 15 or 16 years.

Riham: Yeah.

Participant: I should be speaking Lebanese perfectly. But I don’t.

Riham: You did not learn it?

Participant: I would when I am there, but when I go back home I speak my own dialect. So they could tell I was different in Lebanon.

Riham: I like how… in Michigan there were a lot of Lebanese people. Both Shiite and Sunni. When I was a medical student I did not know the word "hakeem". I thought hakeem meant someone wise [literal translation]. They would ask me where the "hakeem" is and I would say I don't know where you would find a wise person. I spent two weeks not knowing and then my friend finally told me.

Participant: Yes! Because a doctor is supposed to be wise, all knowing. Do you know what "jaajaa" is?

Riham: No, what is it?

Participant: This man. Sameer Jaajaa. He is the “hakeem”.

Riham: It doesn’t sound nice. “Hakeem” sounds nicer.

Participant: Sameer Jaajaa is a person who is wise.

Riham: Oh, I don’t know him.

Participant: They call him “The Wise”.

Riham: I liked that word.

Participant's Wife: Tabeeb is another one.

Riham: Yes I know “tabeeb” and I know “diktuor” but I did not know the meaning of “hakeem”. Yeah, they would say to call “hakeem” and that the “hakeem” came. They have some nice words and phrases.

Participant: The Lebanese people have a nice accent. They are talented.

Riham: Yeah, it's nice. They are known for that. The Egyptians and the Lebanese.

Participant: They speak nicely.

Riham: Is there anything else you would like to add regarding these programs?

Participant: I feel like they need to improve the work situation. As a calling. A calling for us. Each person should be placed in a suitable job until they acclimate to the new environment. Until the mental stress has eased a little. Everyone is an expert in their own field. Everyone in their field is a “Hakeem.” You and me. My wife is an expert in cooking. She is a chef. In the kitchen.

Participant's Wife: You did not know?

Riham: No, I didn’t know. Now I am going to have you cook for me. You’re a chef. Good for you.

Participant's Wife: Of course. Anytime.

Participant: Everyone feels comfortable in their own area of expertise. I spent 12 years under one teacher [apprenticeship].

Riham: To learn plumbing.

Participant: It’s not a matter of learning. I knew my area well. I became a teacher. But I liked him and he liked me. He calls me now and says “Where have you gone, my brother?” If you are good at what you do, have ambition in it, people like you.

Riham: May God send you a job that is suitable for you.

Participant: I wish I can benefit from my training here. Over there I know the tools I worked with, the system, and a little bit of the language. You won't feel stressed if you do what you are good at. Our field is like gold to us. I could get $30-40 per hour here. That is…more than a doctor makes. But where is this job? When we finally find the job of our dreams here, they tell us we need to know the language. I know Arabic, and I am really good at it!

Riham: Is there anything else regarding the medical system here or providers --.

Participant: No, there is nothing else. We just need them to ease our situation with the transportation and to provide interpreters like you mentioned. They should have people to assist you with things that require the English language. They can make appointments for us, and if you don’t know how to make an appointment, they teach you how to make one. They help with understanding the bills. Things like that. This will be helpful and may God reward them. This is what we need.

Riham: Thank you so much for your time.

Participant: May God bless and protect you and make you successful. If you’re single, then may God send you a husband! And if you’re married, then …

Riham: Amen. God willing!

Participant: If you are married, then may God send you a child!

Riham: Thank you. We will end here.

Interview 4B

Riham: Today is May 13, 2017. I am with a mother of six children. Her participant number is 04B. We will now begin. How are you?

Participant: Thank God. How are you?

Riham: Thank God. We will start with a few questions and then I'll give you a chance to share your thoughts on the medical system here and how your experiences have been so far here with doctors in Cincinnati. Do you want to start off by telling me because we actually met in the Emergency Room?

Participant: When we first arrived it was really difficult for us. Especially since we didn't know anyone here. We suffered a lot. The home they placed us in was not good at all. It affected my children's emotional state. There was fear. There was no ease of heart. This is how it affected the children. We took them out of a frightening place and brought them to another place that gave them no sense of security. We left Syria due to the fear, and killings, and oppression. We went to Lebanon and still did not feel safe. We then came here for the sense of security but still did not find it. Our house was horrendous. The neighborhood was scary. We felt we couldn't go out much for that reason. We felt like we were locked up and restrained at home. We regretted the day that we agreed to come here. This stress, the pressure, was hard on the children. The first one to be affected by it was Asma. They forced us to go to the charity organization. Her father, her sister, and I all had to go so she ended up staying with her siblings all day long. We would leave in the morning and not return until around 4 pm in the evening.

Riham: Was it in the Summer? Wasn't she in school?

Participant: No, they didn’t have school at the time. We arrived in June so school was already out. When we went to register them, they told us we need to come back when school starts. There were no activities or programs for the kids? They were stuck at home. It was terrible. They told us no there wasn’t anything for them. The first time I went to the hospital was when my daughter lost consciousness. They ran some tests and said they didn’t find anything so we left. They gave me some medicine, an enema, and told me to give it to her if this situation occurs again.

Riham: Like a suppository. Yeah, go on.

Participant: They guessed that it was a seizure. They thought that cause when the incident occurred she first fell down, then was shaking, and then she lost consciousness. Because of this, they thought it was a seizure.

Riham: Did you understand what a seizure is when they told you?

Participant: Yes, we call it "sara3''. I knew because my sister-in-law has seizures. I compared their situations, but my daughter had not been like her aunt. That's why I didn't believe that she had seizures and told them this. I was not convinced with the diagnosis. When it happened again --. On Saturday, she was fine. On Sunday morning she woke up --. She started to get a headache on Saturday so she took an ibuprofen and went to sleep. We closed the door so it can be dark. Everything was fine. When she woke up she was still fine. She had breakfast with us. When she took the medicine it got stuck in her throat so she vomited. I was removing her clothes so I can wash them and I came back and found her doing this [shows with her hands]. I thought she was sleeping. I started to call her name and lightly slap her face but she wouldn't say anything or wake up. When I did this [shows with her hand], she flipped over on the sofa. That is when I got scared. I went and gave her the suppository like they told me so that she would wake up but she didn't. They told me to give it 10-15 minutes. In 20 minutes max she should've woken up. My daughter did not wake up. When she didn't wake up I called the ambulance. They came and took us. Since it was the second time they take us, I insisted that they find out what is wrong with my daughter. I told them I will not leave the hospital till they find out because I cannot keep coming back to the hospital every day. They admitted us and gave us a room in the neurology department. We met with Dr. Iyad, and they started to run some more tests and the specialists came to see her. They started to work on her case. There is one thing I want to mention though… I didn't know the “routine” [used English word] was like this here. That from the first time something happens and a person goes to the emergency room they shouldn't just give them a pain reliever and discharge them. Tell them “Go.” They should really look into the matter. It's not just superficial. When I took my daughter the first time, she wasn't gaining consciousness. You were there and you saw her. We kept hitting her face but she didn't wake up for a while. I didn't understand at the time why she wasn't gaining consciousness. I didn't know why she wasn't waking up. Why'd she get dizzy to begin with and what was going on with her? It wasn't till much later that the neurologist told me. Two months later, we were having a birthday party and had guests over. I saw that her face was getting pale and she complained of a headache. I told her to go upstairs and sleep. You know where my kitchen is? I didn't see her go up the stairs. She walked from the dining room into the kitchen. I was sitting with my guests. It was the birthday of [indistinct] and Mohamad. We had invited some of the other Syrian families. It was a nice gathering. I got up to look because a few minutes went by and she still didn't' go upstairs. I was wondering what she was doing. I couldn't hear anything either. I went into the kitchen and if you saw the way she looked you would have thought she was dead. Um Hasan's young daughter was standing over her talking to her but my daughter wasn't responding. Even that time they just brought us to the emergency room and discharged us. I felt something was not right. Everything has a cure. I know that God sent down the cure before the illness as we say. In order for a person to get treated. This point is really important. That if someone is in pain and they find the source they shouldn't --. I am not saying it's neglect on their part, but perhaps this is what the protocol, the “routine” is. I don't know the system here well.

Riham: Yeah, it's different than the system in Syria.

Participant: Yeah. For example, we know that if something happens to a child they take them in and immediately give them the medicine. Yes, they run some tests but they give them the medicine right away. Here, all the hospitals do not give the right medicine right away. They wait. I don't know what the rationale behind it is, the waiting, but, in my opinion, it's wrong. If a person has a simple problem, it's going to increase and get worse with time. In the end, it's going to be hard to treat it. Thank God that at the end of all this, I now know what is wrong with my daughter. It's not seizures. Dr. Iyad told me it was not seizures. He said if it happens again and if that when I squeeze her finger or pinch her, she does not gain consciousness, then I should call the ambulance immediately. He said to not even give her the suppository that they gave me. He said because my daughter is not having seizures. If he didn't tell me this, I would have still been giving her the suppository! It doesn't help her and might even be harming her. Let me show you how many boxes I have of this medicine. I don't use it. I don't know whether to throw it out or what. I leave it in the cabinet. I gave it to her once but never again after that. The doctor told me if the incident happens again, to just call an ambulance immediately. He also taught me a few things I can try with her. He said if they don't work and she doesn't gain consciousness, then to call an ambulance. One time she was coming back from school--. I feel like this thing happens to her in a matter of seconds. She came home from school and she was fine. She was coming home by bus. She turned pale. Look how it instantly shows on her face. Her face turns pale and she's not able to open her eyes. She tries to open them but is unable to. Then what happens to her is that she is unable to speak and then she loses consciousness. She no longer is aware of anything around her. When it happened to her for the third time and I took her to the emergency room, I told them I need a specialist. I respect all of them and they did not have any shortcomings with us but I needed to see a specialist. Children's Hospital has the best care and excellent doctors so they should also have specialists available. The children should be referred to specialists immediately. That is their area of expertise. There is a difference between an emergency room doctor and a neurologist.

Riham: Of course.

Participant: A Gastroenterologist is different than an Oncologist. Everyone has their own area of expertise. We got used to the system in our country. If we go to a children's hospital, every doctor has their own specialty. If one doctor doesn't understand the illness, he will send us to a specialist. He will say, "This is not my thing so you should take the child to so and so." It wasn't until after two months that they sent me to a specialist. After I had already put up with so much.

Riham: Did you have an appointment, or did they not make one at all?

Participant: They made me an appointment but it was going to be at a later time. I ended up going back to the emergency room again before the appointment. We went and called them again.

Riham: So it's not really an issue of referring you to a specialist but rather an issue of time.

Participant: Yeah.

Riham: Meaning, they referred her to a neurologist, but they took so long that another incident occurred with her.

Participant: Yeah. Like for example, my daughter is having the same problem recur over and over. They even told her she might need to see a therapist. It's really important for them to see a therapist. Especially for children of war. All my children --. This is a very important matter that the Children's Hospital should attend to. They should have programs every now and then. My children really like Children's Hospital by the way. Whenever we have an appointment there for them they would get really excited about going. They really care for them. A person loves those that care for them. In terms of my children's psychological health, they cannot stand each other. They reached a point where they became mentally exhausted. Children's Hospital is excellent but they need to have programs for children of war. Programs that help them express their feelings and allows them to make sense of everything. All the stress they have inside will only cause them to become ill. Pressure. Pressure. Pressure inside. Emotional stress is much worse than physical stress. Emotional stress on the inside can really destroy a person. We moved our kids from one place to another for this reason. In Lebanon, for example, they didn't have a good medical system. Do you know what they did have, though? Caritas…They had programs for children of war. They were both educational and psychological. They would do everything. They provided all transportation and they would take them on field trips. They would have programs that preached to them. They would have skits and plays. They would be beneficial. This aspect was excellent in Lebanon. I told Deema here, the one that is in charge of --.

Riham: Syrian Foundation.

Participant: I told her that if they do this thing, they will save our kids. You would save them from the emotional buildup they have inside! If it is not released, it will have a negative outcome on society. They are forced to interact and be part of this society. They have a beautiful method here of dealing with children. It's appealing to them and better than in Lebanon. It's better by a lot. The fact that at Children's they truly care about the children, it is also considered nurturing. I wish that my children can be exposed to that and all the children of refugees. Because the things they saw--. If you would ask my daughter, she would tell you how she saw dead bodies laying around. She will tell you about the war. Inside her, this will create a darkness. My older daughter Asma, this emotional pain… is what caused her migraines. At the end, they told me that she was having migraines. From the inside. When the doctor would see her, he would recommend she sits with a psychologist, increase sessions with her. They are trained to take children out of one state and into another. They would give a child hope and a sense of purpose. The way Dr. Iyad…with all her pain…Dr. Iyad changed all of our perspectives. He is Syrian and we are proud of what he became. We saw him standing there, speaking in front of everyone. Like, I always try to motivate my children with this.

Riham: What do you mean?

Participant: I tell them that here we have so many resources. I focus on their potential.

Riham: Yeah, I understand what you mean. As in opportunities.

Participant: Yes. They have so much potential. Every child has a talent and a certain type of intelligence they are born with. However, when they have internal stress and burdens, it can be destructive. We moved our kids from one place to another for the sake of their future. Maybe we lost our chance but they still have theirs. This is really important and it really helps. Us Syrian refugees sometimes see our children, bad…crying…devastated. Even if they are sitting next to other children, you will find them crying. He won't be able to sit still. Because there is something going on inside of this child. It's fear. Fear of everything. The children need to learn how to integrate into the whole society so that they don't feel alienated. Alienation is really hard. To go from one environment to another one that is completely different is difficult. We left Syria to go to Lebanon but they had similar environments. Here, the environment is really different. In order for the kids to learn the customs and traditions --. There aren't traditions to learn here but there are rules.

Riham: There are customs and traditions.

Participant: I don't know.

Riham: You just didn't learn them yet.

Participant: Yes. They need to so that they don't feel any difficulty. This would make it easier on them. Their illnesses will decrease. They would have a sense of motivation. They would find their talents and develop them. I saw something at Children's once. They were doing something with the children. I don't know if it was only for the patients or not.

Riham: Tell me a little more about it so that I can explain.

Participant: I saw a place in which they had toys and musical instruments --.

Riham: Yeah, yeah, yeah.

Participant: I feel like my kids will really enjoy a place like that.

Riham: Yeah. It's like a television show for the children to watch so that they do not get bored. The whole hospital can watch because it is their own channel. They have music and plays.

Participant: It's really nice. This is protective. If a child is in a lot of pain and in a bad state when they go down and see something like that, it gives them hope.

Riham: Yeah.

Participant: They have hope in life. They start to get hope that there is still some good left in the world. That there are people there to help them get out of this state they are in. Self-motivation is the basis of everything in life. If a person does not have the inner strength then they get destroyed.

Riham: As a mother, what do you do at home to help strengthen your children's mental state?

Participant: I start to do for them the things they want to get them out of this state. Take them out. They need to get it from their own peers though. They don't have any friends here. Look how you were talking about your friends? Your friends here help you cope with being away from family. The bond between friends is important. If one is going through something, her friend will uplift her spirits. My daughters do not have that here. How did they get friends? My daughters do not know anyone in Lebanon. However, Caritas [program in Lebanon] brought together all the girls and introduced them to one another. All the girls came together to share their stories. If they had anything they wanted to share they could do that there. They had motivations and goals. They spoke about the things they wanted to do but couldn't. A person sometimes has a feeling of helplessness. My daughter, Asma, has this. I tell her to please get out of this state she's in. I tell her that she is destroying herself. She is always upset, stressed. I don't know what the reason is. There is something going on inside of her but she doesn't share. If she had a friend or a peer --. She might think that if she tells her mother that she will burden me with her problems. She sees that we are going through our own problems with work and our other matters. Our life matters are hard. The children notice this. The children don't want to add on to our burdens. Honestly, the things they saw and heard are way above their age. They are children but did not have a childhood. Their childhood that they are meant to enjoy was replaced with war. The three older ones grew up in the country, and they might have experienced its beauty. The three younger ones did not get to see that. This child, for example, does not know anything about our country.

Riham: The six-year-old.

Participant: Yeah. He was two years old when we left Syria. He was born in 2011, and we left in 2013. We had to migrate at the end because we could no longer stay there. We left in 2011 and went to Lebanon 2 mo but then we ended up going back for the sake of their education. It was hard to put your kids in school in Lebanon. They were expensive. My two older daughters went to [indistint] school but this one [her eldest] could not go.

Riham: She didn't go with them?

Participant: No.

Riham: They did not allow her? She is not that much older than them. She's only older by a year or two, right?

Participant: Yeah, but she is not in the same school. The program is only for the students.

Riham: I see. So there needs to be a program specifically for Syrian refugees. Not just for a certain age group but rather for all the children.

Participant: Yes, all of them. Not just for the children. For the adolescents as well. This age group is so hard.

Riham: Adolescences is hard in any country, any place, and any environment.

Participant: Yeah. In order to prevent our children from leaning towards the wrong things. They need to go out. We cannot lock them inside. If we lock them up it will destroy them. They need to integrate into society and know the good from the bad. In Lebanon, we were in a neighborhood that was filled with drugs and weed. They used to give them lessons regarding this topic. What they should do, and what is right from what is wrong. The kids start to learn about the consequences of these things. They start to learn that they shouldn't use drugs. It happens a lot in schools now.

Riham: Yes, a lot.

Participant: In the schools that my children go to, they have many problems there.

Riham: As in drugs?

Participant: Everything. They are throwing eggs at the teacher. It was in the news and the police were involved. If you look it up you will find it. This is an issue of health….emotional health. They should be given lessons in health class. They should do it even if it's once a month. My kids used to do it once a month but every two weeks they would go down and learn English or hear stories. They would have drawing classes.

Riham: This is in Lebanon?

Participant: Yes. They would give them lessons. To teach them how to defend themselves if someone were to attack them.

Riham: That's great. So that they can become stronger.

Participant: Yes, so that they can strengthen them. They knew which environment they came from and the things that they saw. Kids in this society are sitting like this. This is not healthy. It's not just a matter of health. It means that this child's mental state is suffering. There is something inside that is not good. They are struggling with something on the inside. However, they are not able to express it. If they do programs like these, which are important for the refugees, whether it be in Children's, in a hospital, or in other organizations --. Maybe there are other organizations, but they need to work together, to combine efforts, to do this. It's important to do them for all ages. These children are coming out of a war zone. The children of wars need a lot of work. It's not a word or two. When a child sees someone killed in front them it will take more than one word to heal them. If a child is being threatened with a gun, it will take more than a word. If a child sees their parents being threatened, it will take more than a word. It will kill them on the inside. My kids saw everything. Asma and Amal…the eldest, they were aware of what was going on. Asma had an interest in seeing and knowing but at the same time she could not handle the trauma. One night we were in our home. Our home was on the fourth floor. Air strikes started to happen. We went down to the first floor. It was my brother’s house. I picked up the little ones. They were sleeping so we picked them up. The older ones went down on their own. Asma went down the stairs and entered through the doorway of her uncle’s home. At that moment there were lots of shots fired on the front entrance of the building. The girl held the door like this. She turned pale and froze. She couldn't move. We yelled at her to come in and she couldn't move. She froze. We suddenly see blood coming down her [indistinct]. They brought her in and there were 14 people in the corner. Can you imagine 14 people hiding in one corner? We sat in one corner that was the only area that the gunfire couldn't reach. It was safe from the crossfire unless a bomb came down on us. All these things destroy a child on the inside. When she was seeing her doctor--. The doctor they referred Asma to is really kind.

Riham: The psychologist?

Participant: Yeah, her name is Heather.

Riham: Heather, okay.

Participant: Do you know who I am talking about?

Riham: No, I don't know her.

Participant: She is so kind. She is excellent. She has great methods. She sits with me and asks me to tell her what kinds of things Asma saw. She asks me which way we should go about things. She works with us. There is cooperation between us as parents and her to help my daughter. My daughter's state improved a little. She started to become stronger internally.

Riham: That's great.

Participant: She started to have self-motivation and goals. She was no longer living in fear. She used to be afraid of anything. Everything. In the other house, from how many animals it had around it --. They would scratch on the walls and the raccoon that would pop out from here and another one from there. The children were not used to anything like that. They said that it's normal here. We weren't used to it though. It was too much. We can’t live like this. We used to repeat ourselves a million times before the charity organization did something about it. We spent two and a half months struggling with this issue before the health inspectors came. The bathroom upstairs would leak into the bottom floor. My son and I developed asthma. We had it before but it wasn't this bad. From all these smells. Can you imagine that the garbage truck would not come to us? We spent a month and a half throwing the garbage out in the basement!

Riham: Oh no! A month and a half without waste services.

Participant: The garbage truck would not come. It was unreal. We didn't expect this at all.

Riham: It's wrong.

Participant: They didn't tell us this! In Lebanon, they told us that we will see our house and it will be in the best condition and furnished. Not completely furnished with the best, but in an acceptable state. They told us that everything will be provided for you until you learn how to speak English and are able to work. Only after then will the charity organization leave us on her own, they said. When we came here they stopped after 3 or 4 months. They told us to manage on our own. My husband would get really upset but I calmed him down and said not to go about it this way. My husband is the short-tempered type. I told him that things are slow here. Slow. At the end though, I could not take it anymore either. I lost my patience. They gave us a house against our will. It was not good at all and unhealthy to live in. The health inspectors came and said this house does not meet the health requirements and that we should be moved out of it. By that time, my daughter had already gone to the hospital. If it wasn't for this social worker at the hospital, no one would have listened to our complaints. No one would have listened! Dr. Iyad told me to speak up and not be afraid. He said here there is accountability and you will get your rights.

Riham: That's true.

Participant: If it weren't for the social worker we would have never left that house.

Riham: So if your daughter did not get sick, no one would have removed you from that home.

Participant: Exactly. They said “This, you must accept it.” We needed to live with it. We left our own country so that no one will force us to do something against our will. How dare they try to force us to do something we don't want to do here. I told them that I came to a country that values human rights. "This is not a humane treatment", I told them. I confronted them on their promise to take care of us until we were able to speak the language. We are not robots or computers to be able to learn the language in three months. How could we be capable of paying rent and our debts on our own after only three months? They cut off everything. Even the food stamps were cut off. All because of the organization’s slowness. I told them if they are not able to keep up they should seek assistance from others! I know that there are many charity organizations here. They can work together!

Riham: There are many charity organizations.

Participant: And all they need is for someone to inform them so that they can respond. I don't understand why they were hiding this thing. Did they bring people here so that they can crush them? They are giving the wrong impression of this nation. If it weren't for the fact that we met many Americans, we would have thought they were all like them. Thank God, we were able to meet good people. As for the Catholic Charities, I have no idea why they treated us that way. Um Al-Bara when she came, they also placed them in a home. She has two kids that have cancer. They went and placed them in a home that had no air conditioning. They brought them here during the hot summer. In their neighborhood, a woman kept knocking on their door begging for money and cigarettes and I don't know what else. She wouldn't leave them alone. I brought them in my house for two weeks until their daughter left the hospital. Our house was more suitable. They stayed until they found them a house. Deema even called. We knew Abu Al-Baraa and called to tell them to come see the state of this family. They are not able to stay in their home. Deema and her husband then came and when they saw the situation, she nearly lost her mind. She called the Catholic Charities director and told him that it was unacceptable. She told him that they just wanted to help and did not want anything in return. They don’t want a salary. They were an organization that wanted to help people. She said, “They are like family and we need to be the first to take care of them.” She asked him why they don't inform them of their situation so that they could help. She said that she did not want anything from them. They just want to help. She went on to say that since you cannot keep up we are here to help. She did not understand why they weren't informing them. I told you about Um Hasan as well. I told you to go visit her. Can you believe that they spent three months here and no one knew a thing about them. Her husband almost had it with the charity organization. He wanted to commit suicide. It wasn't until some young man saw them and informed the West Chester mosque, that Arabs started to go visit them. One time in Ramadan, a woman came to visit me with a translator. I told her that there was no one to take us to the mosque and that this was a month that we were anticipating the whole year. There is a prayer that we love to perform during this month. I asked her if it was really true that there wasn't a mosque around. She told me yes there is a mosque. She said that they informed them but that they didn't come to us.

Riham: Did you believe that?

Participant: No. I said how is that possible!? I told my husband and we talked to our family and they all said to not believe that. He said that he lived abroad and he knows how the Arabs are with one another. Arabs stick together no matter where they are. He said to not believe them at all when they say that. Um Hasan even told us how the people helped her when she first arrived. I started to wonder why no one would come to us. Is it possible that no one heard about us? We wondered why. We kept praying that God sends us someone good that will show us around and help us, direct. Once they heard about us, they came immediately. They saw our home, they saw our condition, and they helped us find a new house. They helped us--. The first one I met was a Syrian woman. A translator secretly told me about her once. She said it wasn't in her place to say but she did not like the condition that we lived in. I told her that we spent 10 days all sleeping in the living room. If my son wanted to go sleep in his room he would go in a find the raccoon on the window. If the screen isn't closed, the raccoon would enter and go sleep in his bed. How can a child make sense of this? In his whole life, he has not seen anything like that. Once my daughter was admitted to the hospital, Dr. Iyaad told me to speak up and not to be shy. He said you have rights, and you should ask for them. He told us to speak up. After that, the social worker came to see us. The psychologist also came to speak with me. They all kept coming and making sure that I shared everything with them. The social worker even started calling and getting more information. She wanted to kick herself when she heard what was happening. I was telling Dr. Iyaad that the food stamps had been cut off for a whole week. He was shocked. He asked how were we eating and managing on our own. Had it not been that we brought our own money we would have only relied on the small amount they gave us. We're a whole family.

Riham: You have six kids.

Participant: We have six kids. Our kids are also not used to this. Thank God, in the past, they did not feel deprived. It's not important how much money you have but you should not let your kids feel like there are things they cannot have.

Riham: You mean they don't know what poverty feels like.

Participant: No. Even during the war, they did not feel that. You should see how much food and drink we left in our home. We left it all and came. We had lost one child and did not want to lose another. So we left it all there. We came here. Dr. Iyad said this is unacceptable. He told me to tell the social worker everything. I wrote all the issues down so that I don't forget. When I told the social worker everything, she could not believe it. She said it doesn't work like that here. Then how come the Catholic charities is doing this? She called them. He is from Africa. His English is not the same as an American native speaker. She told him that she did not understand him well. She tried to reach him using many different ways. In the end, she said that she was going to send a letter stating that this amount is too little for us. If my kids do not eat properly, then that is not healthy.

Riham: It will really affect their health.

Participant: Of course. So we can get our food stamps. If you limit the food a child eats then they will not get the nutrients they need. Every day they should have eggs and milk and protein. Their bodies need fruits and vegetables. We need to live. They gave us an amount that was barely enough. We have never deprived our children this way. We came here and could not even give them a dollar to spend as they please. They told us that was the only thing provided. They also forced us to live in that home for about 4 months and a half. It was difficult to move out of there. My husband had to protest till they finally heard us. They said that we did not tell them. I told them that my daughter got sick from the first month and that we told them we didn't to stay in that house. It wasn't in a good condition at all. The location was also terrible. Every single person that would see it would get shocked. They said that it was scary.

Riham: You mean the area.

Participant: Yeah.

Riham: It had violence?

Participant: Yeah. We would only hear gunshots and police sirens. The ambulance and fire trucks as well. We would not even dare open the windows or let alone the curtains. Can you believe that when we got here there were no curtains in the house? It was completely open. I told them that we were Muslims and that we cannot keep covering our heads all day long. We need curtains, I told them. They said they would bring us some tomorrow. They kept saying that tomorrow they would install them. It wasn't until after two months and half that they finally put us some curtains. We sat covered. On top of that, the house is colorful. It's important for us to have our privacy.

Riham: It also affects one's mental state.

Participant: Exactly. I am telling you this is something the hospital should take care of. It is not your responsibility to do the programs themselves, but rather you should team up with other organizations that do them. It will help these types of people. These are children. It is their right to have a life. They need to know what childhood feels like. They do not need to take on the responsibilities of adults from the day they are born. What did they do to deserve this? This is what happened to their country. They also have rights. They told us that we have rights here. We see it here. When my kids go to school, the principal welcomes them at the door. There is nothing more beautiful than that. The way they treat the students, there is nothing more beautiful than it. The educators. On the other hand, there are some things that can be improved. Emotional and psychological success is also important. Helping children reach their maximum potential as well. If they have any talents, they should help them develop them. These things will really help. If you hear the stories I tell you. For example, the last one that came.

Riham: The last one? How long has she been here?

Participant: It's only been a month.

Riham: Oh, she's really new here then.

Participant: Yeah, a month or a month and a half I think. If you see the mother and the children, it is really sad. I remember myself when I see them.

Riham: In your opinion, what can we do for those that just arrived?

Participant: Compassion. Emotional support. A person might not complain but they sometimes just need a hug. They need someone to hug them and help them forget the war and the disturbing things they saw. They need someone to show them the beautiful side of this world. They need to feel happy and free in this world. To feel like no one is interfering in their lives. Do you know what I mean?

Riham: Yes.

Participant: This thing will really help. My kids met some people that have not been to school yet.

Riham: That's unfortunate. Are they older kids?

Participant: They are the same ages of my kids.

Riham: So they are school-aged kids, they aren't young.

Participant: Yeah, all my kids go to school. Their story is really sad.

Riham: Why do you think they haven't started school yet? Is it because of the vaccines?

Participant: I don't know. Every day we hear different stories. They said that when they arrived school was out for the year. When schools started, they still did not let them attend.

Riham: Why not?

Participant: I don't know. It's because of the pace the caseworker is moving at. If a caseworker is not working from their heart, the families that are relying on them will get lost. Especially if they were not strong enough. A caseworker is responsible for the families that come here. They would be the ones that welcome us in the airport. I appreciate that they found us a home and for what they did to us, but how can they ignore our needs and struggles? Here there is nothing out of their hands. If they recognize they cannot meet my need, they can refer me to someone else who can help. If a person cannot do something, then there are others who can.

Riham: The communication and collaboration between people, then?

Participant: The communication between different organizations. They should be proud of the work they are doing with the people they are helping. It will benefit them to help us. Both sides will benefit. It will help their reputation. For example, someone that is doing research on a social issue, if they take the data and helped the people involved in the study, isn't that a benefit to the researcher?

Riham: Of course.

Participant: How many people have you helped with this research? You get twice the reward in this case. You get rewarded by God and rewarded from the people. You also get a good reputation and move up in your job. Is it correct?

Riham: Of course.

Participant: This thing should be a goal. The services that Children's offers are excellent. They really care about the child. Sometimes they call my husband, but of course, he does not understand what they are saying. They should have a translator.

Riham: Yeah they should. Are they speaking to him in English?

Participant: Yeah.

Riham: How is he supposed to understand what they are saying?

Participant: He doesn't. They have both of our numbers so they call him and they call me. Sometimes I am not available though.

Riham: When they call you, can you understand what they are saying or do you immediately ask for an interpreter?

Participant: If I feel like I did not understand what they are saying, then I immediately ask for an interpreter. They then tell me to hang up and that they will call me back. They take a while to call back as they find an interpreter.

Riham: Others have told me that they had some issues with the interpreters over the phone. Are you having any problems? For example, you get a Moroccan interpreter or an Algerian.

Participant: You just reminded me of something. There was this one time when we were in the hospital. We had an appointment. My daughter was in the hospital at that time. They got us an interpreter that was in the emergency room. It was around 2 am at night when I took her. This happened at the beginning of the year.

Riham: Okay.

Participant: Yeah, so we entered the hospital because my daughter had lost consciousness. They gave her medicine. I was talking to the interpreter, and he fell asleep on the phone! I could hear him snoring. The nurse even heard him snoring and she did this [shows with her hand]. She said "oh my gosh", and hung up the phone. She tried calling him back three times. He kept snoring. He was sleeping.

Riham: To tell you the truth that wasn't the first time it happened. It won't be the last, either.

Participant: I don't know if the interpreters have night shifts.

Riham: I don't know. Honestly, since I work both during the night and day --.

Participant: The two times that I took my daughter to the emergency room at night, there wasn't an interpreter on site.

Riham: Available on site.

Participant: Yeah available on the grounds. It was only by phone.

Riham: Yes, by phone.

Participant: Twice when she was talking to me, we just heard the interpreter snoring. I told her that he was sleeping. She is giving med direction, and he is sleeping! She hung up and called someone else. It was hard to get in touch with an interpreter.

Riham: The ones on the phone you mean. How about the translators on site?

Participant: They are great.

Riham: You don't have any problems with communication?

Participant: No. For example, I am the one that is mostly with my children when they bring the interpreters. Even if the interpreter was Palestinian or Moroccan, I can understand what they are saying. If there is anything that I did not understand, then she tries to explain it to me till I understand.

Riham: You are comfortable with the interpreters on site.

Participant: Yes. Only the phone interpreters are not great. Just imagine that something terrible was happening and the doctors want to explain something urgent to me and the interpreter is just sleeping.

Riham: Yeah. It's wrong.

Participant: I am supposed to sign a paper when there is an urgent matter. I refused to sign if I did not understand. That's my right. The interpreter is sleeping. They still bring me a stack of papers to sign. How am I supposed to sign what I do not understand? I even ended up calling my friend and she translated everything for me. If I did not know this friend and if she did not translate for me, then what would I have done? There is a huge problem at night with the interpreters.

Riham: When you would go to the doctors during the day, to your appointments, do you have any issues?

Participant: You mean at the clinic? No, I have never been to them. Since I am working, Carol is the one that takes my children to their appointments.

Riham: Oh, she takes them without you being present?

Participant: Yes. We have a form that we gave her. It's because I am working and their father is also working. It's not possible for us to leave work every day or so and take them to their appointments.

Riham: And you have six kids.

Participant: Yeah. All the appointments. She helps me a lot with this matter. May God bless her.

Riham: Is she from a different organization?

Participant: She's a volunteer. The organization called her once to help. She liked us a lot and we liked her as well. We got attached to her. She helps us so much. Without her, I would not have been able to do anything. She helps us a lot with filling out forms and with the appointments. She is the one that makes our appointments for us. We don't know how to make appointments. She does everything. She has a notebook just for us. She writes all the specifics in there. She has all the birth dates because of it is normal to forget. She has all our names written along with our doctor appointments and everything. She calls and says, "You have an appointment tomorrow." Or she says, "The children have an appointment and I will pick them up from school". She is the one who takes them to their doctor's appointments.

Riham: How long is she going to assist you?

Participant: All the time… As soon as I say, "hello" --. The other day, my husband got really sick. I don't have a driver’s license so I could not drive the car and take him. I ended up calling her. She had just finished taking my daughter and me to the dentist that morning. It was a Saturday. She dropped us back off at home and left. I ended up calling her and telling her what happened with my husband. She dropped everything and rushed back to help.

Riham: Why didn't you call an ambulance?

Participant: I don't know why.

Riham: I am not telling you that you should have called. I am asking why --.

Participant: Do you know why I didn't call? If I were to call the ambulance they would have taken him to the UC.

Riham: You didn't like the UC?

Participant: The UC emergency room is not good. They do not have good services when it comes to the emergency room. Can you believe that once when I called the ambulance and they took her to the UC --. I thought they would take me the Children's as soon as they saw our paperwork. They didn't and took us the UC once. The girl had been unconscious for three hours, and they did not bring her back from it. Their reason was that they still need to open her file. I kept saying come see the girl. They said they needed her files from Children's before being able to treat her. This is not good at all. The girl could have died.

Riham: God forbid.

Participant: It's unbelievable. I would never go back to the UC for an emergency. I told my husband not to go to the UC when he had an emergency. There is a hospital nearby. It's about 8 minutes away and we were told about it by Jenny. She said to go there instead of the UC.

Riham: Did you like that one more?

Participant: Right when you enter, they call you in. When I took my daughter Amal to the UC for an emergency, I swear she was sitting in the waiting room for at least five hours.

Riham: Just waiting.

Participant: From 10 am till 5 pm. Count how many hours that is. Waiting.

Riham: So they had a long waiting period.

Participant: Yeah. They took her name and ran a test and then had her wait outside. They did not give her a pain reliever or anything. She was screaming and crying in the waiting room. From her abdomen.

Riham: This is before they found out about her digestive problems?

Participant: This was when she went for the first time. It was before they found out that she had Crohn's. They actually weren't the ones that diagnosed her.

Riham: This whole time that you were waiting, was it in the waiting room or --.

Participant: Yes, it was outside.

Riham: It wasn't in a room, then.

Participant: No, it was outside with everyone else. It's a really big place.

Riham: They have a lot of people that go there and are very busy.

Participant: A lot. You would see a lot of people there. One person would be screaming and another would have an injured head. They still wouldn't call them in. They would say that they don't have any available beds.

Riham: Yeah, they won't have available beds.

Participant: I went to the cafeteria after a few hours to get her something to eat. I went up just to ask where the cafeteria is and then I see Dr. Mulham. He asked what was wrong. I told him what happened with my daughter and that we had been waiting there from the morning. He asked why they did not call us in yet and I said I didn't know. He reassured me then that her problem is most likely not urgent so not to worry. He went and talked to the receptionist for us to speed it up.

Riham: Yeah, that is the problem that we have with big hospitals for adults.

Participant: The public ones.

Riham: No, not the public ones. The bigger hospitals, they open their doors to everyone. That is why the waiting room time is longer than usual.

Participant: Yeah, they don't even provide an interpreter.

Riham: Yeah, they do not have the resources.

Participant: Yeah, they do not have the resources needed to help the people.

Riham: Yes. That is why it is difficult to work in these places. The doctors are not enough, and the patients are many.. and there aren't a lot of beds. I mean not enough for everyone. Do you know what I mean?

Participant: Yeah. Can you imagine that when my daughter finally was called in, they did not even have an interpreter? How was I supposed to understand? They didn't call an interpreter. There happened to be a woman from Sudan there. She spoke Arabic.

Riham: Thank God.

Participant: Yeah, she was cleaning and saw the way I looked. She came and asked if she could help me with anything. I told her to come help us understand why my daughter was feeling the way she was. They just put us there and left. All morning they had us waiting outside. When they finally called us in they still left us waiting in a room. Did they not want to give my daughter anything? Did they not want to do any imaging? an ultrasound? She said they had a machine for females that could help them examine everything [vaginal ultrasound] but I told her she could not use it. She got upset and left.

Riham: Who, the doctor?

Participant: Yes.

Riham: Maybe she did not understand the cultural context?

Participant: I don't know. Maybe it was because I did not let her do her job. She was a specialist and came to do her job but I did not let her.

Riham: Were you able to explain to her why you were upset?

Participant: No, she just left. I told her I will not allow you to examine her but you can just do an ultrasound.

Riham: There wasn't an interpreter, right?

Participant: No.

Riham: So maybe there was a misunderstanding. She didn't understand you and you did not understand her.

Participant: I don't know. There was no interpreter. There is a huge difference between UC and Children's Hospital. I swear, even adults would wish they were children just to go there. When my daughter would go to Children's, she would come out and tell her siblings about her experience. When the older one would go as an adult to another hospital, she would have the worst experience. She would then say that she wished she wasn't that old. Her psychological well-being is not great. I don't know what to do for her. I almost gave up. The closest I have ever come to giving up. The oldest one is not coming out of this state that she's in.

Riham: Can you send her to see the doctor that Asma is seeing?

Participant: I wish. I don't know if they would accept her.

Riham: Ask them.

Participant: Who am I supposed to ask?

Riham: Ask the same person that you make Asma's appointments with and tell her that her sister--.

Participant: The inpatient division was the one that requested she see a doctor and they are the ones that sent her.

Riham: Yeah but if you are able to speak to the therapist herself or to the social worker --. I forgot what they call her.

Participant: The caseworker?

Riham: No, the one your husband told me about. The one that wrote the list. It's not a therapist but someone else that helps you with these matters. A social worker

Participant: Yeah, a social worker. I know what you mean now.

Riham: Can you talk to them and ask?

Participant: I don't know how to talk to them because I don't speak English.

Riham: Okay, then use an interpreter.

Participant: Should I call them and say I need an interpreter?

Riham: Yes. Tell them you need an interpreter.

Participant: Yeah she actually gave me her number at the hospital and told me to call her anytime I needed anything.

Riham: Yeah. Heather, right?

Participant: Yeah.

Riham: Tell her that you have another daughter so that she can either refer you to someone else or that she can also see her.

Participant: She is fed up with her life. She's my daughter and I feel like she is fed up with her life.

Riham: Do you feel like she is--.

Participant: She is no longer motivated to do anything, I feel. She just lays down in bed and sleeps. Sometimes she covers herself but she is not even sleeping. She would either be crying or upset from something. The pressure we get from work is a lot.

Riham: Does she work with you?

Participant: No, but she helps me with cooking. If she's tired then I don't allow her to help. I send her upstairs to sleep. Her pain is bad. She has no energy to help.

Riham: Let me see what time it is. My phone is no longer working from the heat.

Participant: It's 3:30 now.

Riham: Okay, we will end in five minutes, God willing. Is there anything else before we end? What I heard from you is that we need a program for mental health, a program for skills and growth for children.

Participant: Yes. In any field. Anything can make a child happy. It also should not be age restricted. Every child of war should be able to attend. Young. Old. Adolescents. Let me tell you what they were doing in Caritas.

Riham: What is Caritas?

Participant: Caritas is a foreign name for the program.

Riham: Caritas. Is it a name of an organization?

Participant: Yeah. It's called Caritas the foreign company. It deals with mental health and it's for refugees. Do you know what I mean?

Riham: Yes.

Participant: It would meet the needs of the things that were missing. In Lebanon…they recognized the shortages. It would have skits for them and they would bring doctors to educate the older kids. They would have workshops.

Riham: Here, they didn't have lectures on health?

Participant: No.

Riham: Not at all?

Participant: No, no one told us if there were.

Riham: So if we would have programs about health that talked about things like home remedies and how to take care of one's body – health literacy…

Participant: Yes, and how one would acclimate to a new disease. Like my daughter, how she got Crohn's. She should learn how to cope with it and not keep thinking about how she is ill. If a person keeps focusing on their illness, it makes them worse. For example, if they would do it once a month or even every week. Actually every week someone needs to meet with the refugee kids. They should split them up into groups depending on their ages. The adolescent age is really hard.

Riham: Yeah, it's really hard.

Participant: It's really hard. They need guidance on how to manage their emotional state, their social skills, and their health. They are able to do all this. This would be a great mark that they left in history. They would be helping a child come out of a terrible state and into a better one. When you would have like an interview with a child--. In Lebanon, they started interviewing the kids at the end of our time there. To speak about how they were and what they became. My daughters would never dare walk the streets before. After that, they started going out and coming back on their own. They gained confidence and strength.

Riham: Yeah.

Participant: They had trust in the people around them. They would have increased self-confidence as well. They felt like no one can mistreat them. Can you imagine that they brought them martial arts instructors that taught them how to defend themselves? Like judo. It was specifically for the females.

Riham: That's really nice.

Participant: They didn't tell them that they were girls and should not be doing things like that. They told them the opposite. That because they were girls, they needed to learn how to defend themselves so that no one can harm them. It was their right. That's what needs to be done for these children.

Riham: You also told me that unfortunately there were times when girls would get kidnapped from school.

Participant: Yeah, in Syria.

Riham: In Syria, right. Your daughters saw this thing happen.

Participant: My children did not see it happen, I did.

Riham: I mean they heard about it happening. They did not see it with their own eyes but they heard about it happening.

Participant: Yeah, they started to get scared.

Riham: Yes, they heard about it and it scared them.

Participant: I started going to pick them up and drop them off. My children's schools were near my home. The high school was on this side and the elementary school was on the other side. When anything would happen I would have to run to that side and get the older kids and then run to the other side and get the younger ones. I would feel like my heart was torn to pieces. I would just pray for God's protection. “Please God have mercy on us.” Thank God that nothing happened to them. However, emotionally they are really suffering. A lot. All the kids are. My children are like any other Syrian refugee. They used to bring them people that were specialized in working with children. They would have programs for them. You would find the children either drawing or creating crafts… doing something else. They would have skits and would celebrate the holidays. They would put joy in their hearts. The kids would leave the house a certain way and come back feeling different. They would be like chirping birds when they came home. When they came here, we expected to experience similar things. They got depressed here. Can you imagine that when they tell them that there is a holiday or a break from school, it would be as if they were at a funeral? They would be really sad, honestly.

Riham: Because there is nothing to do?

Participant: Yes, there is nothing to do. There is nothing for them to busy themselves with. For example, during the weekends, they look forward to going to the mosque. At the mosque, they would learn English and each child would have a different teacher based on their age. They teach them. They bring them toys and snacks. They place the love of learning in their hearts. Do you know what I mean? The children would improve a lot from all this. They have a daycare now. They brought some girls that like this type of thing, and they help in the daycare. The Syrian kids are plenty. If we sit in the lesson with all our kids we will not learn anything. The kids might cry or want to eat or drink and I don't know what else. Do you know what I mean? So they started this new set up the other week. The kids did not acclimate to it yet, though. The really young ones still did not adjust to this new setup. The three or four-year-olds.

Riham: Yeah, it's early.

Participant: They are still not used to the people taking care of them. I even told Haneen the other day. Haneen is the woman in charge of this. I said, "Haneen when it comes to children, if you want to appeal to them, you need to bring them something strange. Not something familiar to them." They have toys for them there and stories but they have these things at home and they are familiar with them. However, if for example, they bring a toy for the kids to build or something that requires them to use their minds, this is really good. A child will also develop their mental skills. They will try to take on the challenge. Even if they are young kids. My niece is two and a half years old and she goes to the daycare in Canada. Yesterday, her teacher gave her a board and she drew a flower on it for her. She told her to color it because it is a gift for her mother. She sat and told her mom to wait and not go as she was coloring. The teacher had all the colors in front of her and she would choose the ones she wanted to use. This is a really nice thing and it's new. It's simple but it's interactive. It allows a child to think about how they will go complete the activity. They will think of how they will make it look nice. This is just a drawing but she gets to use the colors she wants. In Lebanon, this was the best service they offered. All the other services for refugees were lacking. This was the most beautiful thing they did though. The kids used to love Caritas. You could even quiz them on why they chose the colors they did and why the like it. This will allow the child to express their feelings as well. This child will then be able to play a role in society. He will become a strong individual. He will not allow any little thing to break him. He will no longer be afraid. He will know that there are people --.

Riham: The pain will decrease.

Participant: Yes, it will decrease. The pain with everyone decreases. Honestly, my young children have left that terrible emotional state they were in. Asma did as well. The doctor helped her so much. Amal, however, is still not getting out of that state. We couldn't tell on the outside that something was wrong. I guess she was suffering from the inside and hiding it. Asma used to yell and cry and get upset at her siblings but Amal never did. She never used to say anything. That is what really affected her. I don't know if you noticed but you could tell from her face. It has no life in it or energy. A girl her age should be at a high peak of life and energy. She should have many goals. My daughter says, "If God takes my soul, then I will be at ease."

Riham: Does she say things like that, now?

Participant: Yeah. She says when God takes her soul she will be at ease. Especially after she learned that she had Crohn's.

Riham: You really need to get her help.

Participant: How should I know where to go?

Riham: Once we turn off the recorder I will give you some numbers, God willing. I will send them to you either tonight or tomorrow, God willing. I will try to help you find someone because this is wrong. You should watch her closely though.

Participant: I get scared, honestly. Yesterday, I was at work and the whole time I am sitting at the machine, my mind is on my children. At one point it's Asma and another point it's my other daughter. When it comes to the younger ones, I am not that worried, thank God.

Riham: Yes, the younger gets, praise be to God, are like a sponges.

Participant: Yes! They can recover quickly but the older ones are depressed. Due to the things that they missed out on and on top of that they are in their adolescent years. That is why I am telling you about these ideas. Maybe you are not able to do them as a hospital but there are many organizations that do.

Riham: Yeah, there are. God willing, I will take the things you have said and at the very least we can connect a Syrian refugee child to the right organization that can help. There are many organizations. To be honest with you, I don't really know them that well because I am new here.

Participant: Yeah. In Caritas, they had programs separately for boys and one for girls. Certain things would appeal to the boys more and they would provide it for them. Same with the girls. May God reward them. There were two directors of Caritas and they would constantly tell me that I had many ideas and that they would like to hear them. They asked me what I wanted for my kids. They also asked what problems did I feel my kids had that needed to be addressed. I would tell them that my girls would be scared of everything. I wanted someone to help them become stronger. They then set up that self-defense program and gave them lessons on the topic. They would empower them. The teachers would tell me what they did. The girls wouldn't tell me a thing but I would see them change for the better. When I would tell the director this she would become happy knowing that they are having an impact on the kids. They are taking them out of a bad state and into a better one. It makes them happy to know that. When I told them we were leaving they said they were really happy for us but at the same time they were sad to lose us. They reassured me that the future of my children will be great here. They would talk to them a lot about the potential their future has here. When they came here, they did not see it though. They were shocked. Especially the older ones. Amal wasn't able to attend school. Even if she were to lose her voice asking… she would not be able to attend school. If it wasn’t for Jackie, the social worker? We could not place her in a school. I told her please, my daughter is dying to go to school. She is seeing her siblings go to school and she has to go to work. It's her right to get an education. How can she not be able to attend school? Some Americans told me that there are schools for her age. She still has a chance to attend.

Riham: Please excuse me, but I am going to interrupt you because I have to get to work. I apologize. Is there anything else before we end?

Participant: No, just these things.

Riham: Okay.

Participant: If you were to address these things you would have done something huge.

Riham: God willing. Thank you so much.

Participant: Thank you as well.

Interview 5A

Riham: Today is May 17, 2017. We are with participant number 05A. He is a father of seven children. Their ages range from 10 months to 18 years old. I am Riham.

Ahmed: I am Ahmad and I am here as well. Let us begin. How long have you been here in Cincinnati?

Participant: I have been here for three months.

Ahmed: Three months.

Participant: Yesterday, I completed three months.

Ahmed: Okay. How has your experience been here so far?

Participant: Honestly, it's been a depressing and frustrating experience.

Ahmed: What matters were the most frustrating?

Participant: Honestly, it was the first house we lived in and also the incident that happened to me with the shots fired at the house. The neighborhood was filled with crimes and was old. It had snakes and wild animals that we didn't know what they were. A person would not dare open the front door of their home out of fear. We would get frightened of everything. My kids were hiding under their beds. When we first arrived, the situation in which we were living was much different than it is now. The neighborhood was a lot different than this. We were surprised and shocked by these things. We became completely depressed. We did not feel anything. I was in the hospital at Cincinnati Children’s when we first arrived. An ambulance took my son there immediately. My wife and kids went home. There was no way to communicate with my wife. I did not know if they arrived home and what they were up to. Did they find food to eat or not? I had no idea or knowledge of how they were. I spent four days --.

Riham: You spent four days without speaking to your wife?

Participant: Yes.

Riham: Oh no.

Participant: Yeah, honestly. Then God sent a young man from the mosque. He was the one that greeted us at the airport when we first arrived with the charity organization.

Riham: Rahma.

Participant: Exactly. He was from the Rahma organization. He came with me to the hospital and said my family was doing well and he was going to let me speak to them. There was a woman that worked there named Jackie. I spoke to my wife and I asked her how the situation was. She said they wanted to go back because we cannot live here. They then took me to her. I left my son alone in the hospital and I told them I was going to leave for an hour. They said it was fine and that my son was sleeping. I went home for an hour to see how my family was going. I asked them what happened. They said we cannot live here. Come see how the house is. It was filled with snakes and [indistinct]. It was a terrible situation. At night people would come and smoke in front of the house. They had weapons with them as well. The place we used to live in had a small opening and the other side of the road was closed. It was where many people that smoke weed and do these things come to hang out. The whole area had only Black residents. It didn’t have any Arabs living in it. It was as if they found the cheapest place possible. On top of that, the rent was high. I later discovered that the rent was 500 and they gave it to us for 850. There is greed in this organization. When they would rent out to the refugees, the owners would get greedy and raise the rent. After the four or five months are over, if a person wants to stay in their home, they would need to make a new contract with the landlord. He might then subtract 200 to 300 dollars if he sees that you are interested. However, the first few months they raise the rent so they can use up all the money that is assigned to you.

(3:58)

Riham: That means the amount they give each person --.

Participant: Yes, 1,075 a person. Out of the $9,675, I only got $500 of it.

Ahmed: What happened to the rest of it?

Participant: The rest of it they charged me $500 for the beds and the mattresses were I don’t know how much and a lot of other things.

Riham: Oh no! Explain it to me once more. So they are charging you for everything they provided for you?

Ahmed: Without consenting to those things.

Participant: Yes. They said the $850 was rent, another $850 was for the security deposit, and they made me sign some papers. Then he said they brought us beds and told me the amount. I don't know the prices of things here. If he were to tell me they cost a $1000 or $2,000, how am I to know? Even if he told me a $100, I wouldn't know. He just made me sign here and there. They gave me an amount of $740 by check. My caseworker came with me. We cashed it out at the bank and left. He went and bought me two phones and some breakfast. It was about $100 or $200. He said the rest was for me. I did not know anything about the currency here to be able to go to the supermarkets here. I wasn't even able to leave my home. After ten days of being in the hospital, I went back to find my kids frustrated at home. When I would ask them what is wrong, they said come see what we see. At night, we heard gunshots fired on the windows. People that come here do drugs. The neighbors told us that drug dealers were living in our home before we came and they were kicked out by having their things thrown out or what not. We went to the charity organization and told them to change our home. They said we cannot change it till after three months. I said I would not wait three months because it would kill me. I wasn't even able to sleep at night. I had a son in the hospital. I had to go back home because I cannot let my wife and kids sleep alone at night in that house. No one knows them to check up on them. Somehow, word got around that I came to the organization with my family. I said I am going to jump off the building and commit suicide because I did not have the power to change anything. I wanted to go back to Jordan. Take me to any other country, but I will not stay here I said. They said to calm down and that they will resolve our issues soon. They took us to a different area to show me a house next to a church. The man in charge of the church said that we could live in that house and I could work at the church. They would give me the house for free. I said just let me live here now. They said no, after three months. All that talk was just a show. To get my hopes up by telling me they will give me a house for free and provide me with a job. I told them I don't care about the money. I will pay the rent for three months just to leave that house and I will get another one. They said my benefits would end. I said I was willing to pay anything. I just needed to get out of that home. They said that they will find me another house tomorrow. I went and slept at some people's houses during the first night then I went back to my family. We locked the doors and kept hearing things from outside every little bit. We stood there holding a stick and feeling afraid. Inside the house, we would hear things on the wood as if something was inside. It woke up the whole family. We would just sit there and cry.

(7:36)

Riham: I am hearing you say that you felt fear.

Participant: Yes, it was fear.

Riham: You came from a frightful place to another frightful place.

Participant: Exactly. We would just sit and cry. When we got the phones we would call Jordan and talk to our families whether they were in Jordan, Lebanon, or Turkey. We would tell them to help us with our situation. Some people even wanted to come and we warned them not to come. We said we were in a terrible state. We said no one should come here. We told them they would rather die there than come here. We explained the situation to them. We tried talking to the charity organization, and they would not answer. The mosque started looking for a house for us. They found us a place and said once the woman moves out, we can live there. We spent the first month there. The second month, he told me they are waiting for the house to be available. We waited that whole month and again it was still unavailable. We started talking to the people that we knew here. We said to please find us a home. A man heard about us. A woman was volunteering in the mosque and her name was Haneen. She is Palestinian. May God reward her and her parents. They came to us and asked us what we needed. They started to visit and take us out. They brought us food and things we needed. We spent a while not knowing how to eat our own food.

Riham: I was under the impression that you get an amount --.

Participant: Yes, $750.

Riham: No, the food stamps.

Participant: It hadn’t come yet.

Riham: How long did it take?

Participant: The food stamps came after 15 days. We didn’t know how to go and use it. We didn’t even have a car to go in.

Riham: So they gave you something, but you were not able to use it or bring food with it.

Participant: Yeah.

Riham: No one --.

Ahmed: Taught you.

Participant: I sent the mosque a picture and asked them, and they said that the next day, they would send someone to us. The man they sent did not come. Then this woman came, the Palestinian one. She told her father, and her father came. He came and helped us use it. It was from Kroger. We ate and managed at that point. Before resolving this issue we did not eat or drink or know how to deal with any illness. My son was sick and the other one got a high fever. He told me to call 9-1-1 and they would come. I did not know how to call and speak English. I always tell them “Arabic.” Their mother brought some medicine from Jordan. We spent that month living in the worst conditions. After two months we said we will not continue living there. They said okay fine. That is when they found us this house (points around). The landlord of this house said it would not be available until the fifth of the month. As soon as it was empty we could bring our stuff here. The woman then changed her mind about moving. It wasn't until the end of the 3rd month that we finally left that house. We spent three months experiencing things you cannot imagine. Even if I kept speaking, I will not finish telling you all the things we went through. I am only giving you fragments of it.

(10:51)

Riham: Yeah, of course. I don’t really know your story well but I do know bits and pieces of it. You came to America because your son was sick, right?

Participant: Yes.

Riham: Tell me a bit about his illness. I know that Children’s Hospital was aware of your case ahead of time. That’s not a long time. There are some refugees that we know things about and some that we only know a little about. When you arrived, did you go directly to the hospital from the airport?

Participant: Yes.

Riham: Okay, tell me a little about your son.

Participant: I used to live in Jordan. I spent four years there. We were in a terrible situation. It was, however, an Arab country, and I felt settled there. In terms of education, my children were not receiving any. They would study some things because life was difficult for us. My kids and I used to work just to keep up. They came to us and said we had an interview. We went to an interview regarding coming here to America. They told us it was better for us for the sake of our children's future, and that America was beautiful and what not. We signed up so that we can learn more about it. We started asking around as well. We started hearing that it was better for us to go to Britain or Canada or Sweden. That America was not offering good services for refugees. They said that they would leave them on their own after a month. After three months I was told that they had no longer had anything to do with us. We were completely on our own. Today, they told me this. Some people can tolerate these things and others cannot. We told them okay we will rely on God and accepted. We had some interviews. When we reached out to people that came here they warned us against coming. They said they lost their children. Things like that. The process was long. It kept getting delayed every month. We were also really hesitant about coming. In Jordan, we felt some pressure as well. They said we were not allowed to work and things like that. That the work permits were not for Syrians. Jordan also started to do this thing where they were finding reasons to kick people out and send them back to the Syrian border. People started to die. Now they have approximately 40,000 people between the border of Jordan and Syria. Some of them were ones that came and were not allowed to go in and others were ones Jordan kicked out.

(13:47)

Riham: Were you living in a refugee camp?

Participant: We were living in Amman itself. Those living in the camps had an okay life. We went out so we could get our children some education. My sons were also able to work. Some of them worked in restaurants and some in coffee shops. We lived there and had [indistinct]. My wife had 5 [indistinct] children. She would cook and clean and do all the housework. It is like that till now. She had to do it on her own. She did not have a washing machine so she had to hand wash everything. She started to feel pain in her hands and feet. I started hearing that America was a land of prosperity and luxury. I thought I could make something of myself. I thought that perhaps we could tolerate the situation here. Over there, I got exhausted. There they just need people to work for them. I agreed to give it a try and trust in God. We went to the organization and they sent us to do a medical exam. We went to do this medical exam. When we were there, they gave us shots. When they gave us these shots, his feet started hurting him on the first day. His fever was high as well. They said that was a normal symptom of the shot. The next day, my son said that he wasn’t feeling well. He said his feet were hurting. I told him that perhaps it was from work or walking or something like that. On the second day, he said that he noticed that he had not urinated since getting the shot. On the third day, this was swollen [shows with his hands]. The date was the 23rd of November. The vaccine was taken on the 21st of November. On the 23rd, he said that we were all getting better, but he felt something was wrong with him. On the 24th, he developed urine restriction. On the 25th, at 11 pm, his stomach got swollen from here [shows with his hands]. We took him to the Emergency room. They said they needed to empty his bladder. They used about 4 bags to empty his bladder.

Riham: You mean it was really full.

(16:43)

Participant: Yes. The doctor came and said to do a certain procedure. They did not listen to him. They said he would be fine now that they emptied it. We said he wasn’t able to walk on his feet, though. He started to crawl. It was hurting him up to here.

Riham: Before that, he was completely fine?

Participant: He was completely fine and working at the supermarket. When we got home, it was around 1 am and he said that his feet were not able to carry him. I told him to go to sleep. He said how was he going to sleep. His feet have been hurting him for two days. He thought he was just exhausted. We brought him cream from the pharmacy. I told him to crawl and stay off his feet. He started to crawl to get around. I thought maybe he did not want to work or something. We did not really look further into the matter. The next morning we found our son on his bed saying that he wanted to die. We said, "what is wrong with you?" He replied that he could not feel anything from this part of his body to that part. He said he is still unable to urinate. On top of that, he is not able to get himself to the bathroom. He ended up injuring himself while trying. We found his back injured. This was now the 26th of November.

Riham: Meaning it was after it by one week.

(18:21)

Participant: It was five days after the shot. I decided to take him to a specialist. They were really expensive. We went to a urologist. As soon as he examined him, he said that my son needed to see a neurologist. They referred us to a neurologist. Right when he saw him, he said you need to take him to the hospital immediately. He said it has to be a good hospital. He said my son could have Guillain-Barré. He said it could be several other things but that it is spreading in his body. He said it was a virus that is spreading through his body. He said if your son gets to the point of needing a respiratory device then it is over for him. We called an ambulance and took him to a hospital called Al-Basheer Hospital. It was a public hospital and the biggest one in Amman. When we went there, they examined him in the emergency room and said that he needed intensive care and that they did not have one. They said we needed to find a hospital that had an intensive care unit. We asked the doctor and he started to question us about what my son ate. We said he did not eat anything that could cause this. He asked if he took any medication. We said no. We completely forgot about the vaccine that he took.

Riham: You forgot.

Participant: I forgot. The woman started to call us from home and say that most of my kids were sick. They saw their brother and felt something was wrong with them as well. I went to find him an intensive care unit with an available bed. LC or I don’t know what it is.

Ahmed: The ICU?

Participant: Yes, the ICU. That way they can put him the respiratory devices and other machines.

Riham: The ICU?

Participant: Yes, the ICU. They did not have a place. I went around to the different hospitals looking and had the doctor's report with me. A doctor I met told me I would need a lot of money. He said that my son would have to be admitted and need to take lots of shots. These shots might cost $8,000 or $6,000 or even $5,000. He said my son was now in critical condition. He spent two days in the hospital and no one cared for him. Until one day a man came and said he heard about my son was 18 years old and he wanted to save him. He said that perhaps if he were to save my son, then he will also be saved. He said he had been there for 11 days and he wanted to transfer to the internal medicine unit. He went to the doctor and said that he wanted to give my son his bed. The doctor assured him that he was in a stable state and was just in need of further observation. Since this man was no longer in critical condition, he moved and gave my son his bed. We started to cry. His mother also started to cry. We felt like people were empathizing with us. Some were offering to help pay thousands of dollars for my son's treatment. The doctor told them it wasn't a matter of money. It could add up to $80,000. Then my son started to tell me that all this happened to him from the shots he took. I asked him which shots. He said the vaccines. We had a paper stating that if we had any symptoms from the shots then to call the organization and inform them. I called them and no one answered. The next morning, someone called me saying that they received my call. I told them my name and that they gave me this paper telling us to call if anything were to happen after taking the vaccines and I explained to them what happened to my son. He told me it wasn't from the vaccine and hung up the phone. I didn't know what to say, honestly. The doctors came and started to bring in other doctors to examine my son and run some tests. They sent the results and told me that there were shots they needed to import. They said they did not have them there. He said each shot was $6,000. I needed three shots so that would be $18,000. Someone I knew said that if I were to give him the passports he could find a donor. He said there was this wealthy donor who would like to donate but wants to make sure he does not get cheated. He said to give him the passports and he will buy the shots for us. They then brought us the shots from a clinic or doctor. They gave him the first shot and my son started to lose consciousness. He was hooked up to the respiratory device at that moment. They took him to do an MRI and said that the white or gray part here was being cut off by the virus. They said that they did not know what my son had. The symptoms were not matching up to any disease. There was something strange about this case, they said. They kept going on like this for three days. Then they asked for the papers we received after taking the vaccines. They took it and a doctor named Amjad Al-Asaq who was a neurologist. There was also a doctor named Jamal Al-Akhras. I also have his reports. They took the paper of the vaccines and left. At that point, they had given my son all three shots, but he did not respond to them. They said that the virus has reached my son's brain. There was nothing else that they could do, they said. Just put your faith in God. I started to ask God to save my son. Thank God, He did.

(24:23)

Riham: Thank God.

Participant: The next day they came and said that the vaccines were the cause of my son’s illness. They said that my son had a disease called ADEM [Acute Disseminated Encephalomyelitis].

Riham: What is it?

Participant: ADEM [Acute Disseminated Encephalomyelitis]. They said the vaccines caused this illness. They said that out of 3 million people, it affects 2 or 3. They said this was my son's destiny. I told them that the organization said it wasn't from the vaccine, though. He said that this was his diagnosis. He came to this conclusion after doing an MRI and all the other exams all over again and confirmed that this was the illness. He said if anyone says that this was not the case, then to show them the report. He told me how he was going to treat it and that he was going to prove it to me by giving my son two shots. He gave him the first shot and the next day he gave him the second shot. Prior to giving him the shots my son was in and out of consciousness, he would laugh at times, and do certain things at times. I have it all on video. After the first shot, he started to converse more with me. After the second shot, he started to become more stable. After the third shot, my son improved so much. He spent two days in that state. He started to be able to breathe on his own. However, he was still having issues urinating. He spent eight days--. Was it 8 days?

Participant's Child: 9 days.

(26:05)

Participant: 9 days. On the 6th or 7th day, he started to ask for food. There was a doctor there that was managing the machines and he was shocked. The doctors started to have some --.

Riham: Hope?

Participant: Yes. They said by next week your son will be able to go to another unit. He said that he had found his cure and he was online consulting others and what not. He said that this was Adam's Disease. He said that my son was no longer in critical condition. He took him out of the ICU and into the neurology unit. When we transferred to that unit, the immigration organization showed up with a doctor named Dr. Manal Ali. My son started to get mad at her and asked her to leave. He blamed them for what happened to him. She said we want to help you get treatment outside of this country. I said that when we first talked to them they did not help and only after they heard about my son getting out of the ICU and was in a stable state, they came They said they wanted to come and prove that it was not from the shot. After a few days, the doctor came back with a different approach. She said they wanted to study the case because it was a rare thing. They wanted to ask us some questions. We said if that was the case then we will cooperate. Perhaps others will learn from this. She went in and started to question us. She kept asking about his vision every now and then. She would ask him so many different questions and we did not notice that she was not there to help us. It turned out she was making a case against us.

(28:25)

Riham: Excuse me, but I did not understand this last point. She was examining him --.

Participant: She was examining to see whether this was a normal illness or from the shots. She was examining him as if he had Guillain-Barré and not ADEM [Acute Disseminated Encephalomyelitis].

Riham: I’ll be honest with you, I heard of Guillain-Barré but I don't really know much about ADEM. I heard about it once.

Participant: The doctors were coming up to me and asking if I was being serious or not. I said I was being serious. They joked that I was claiming there was a disease called ADEM and asked if there was Eve’s disease as well? They asked where I got such a thing from. They said that in their 40 years of practice, they have not heard about this disease once.

Riham: I am still new.

Participant: When they heard about this disease and they looked it up online, they came back and apologized.

Riham: Sometimes there are diseases that are rare.

Ahmed: You won’t find many cases of it.

Riham: I have seen Guillain-Barré even though it is also rare.

Participant: Yeah, they told me this.

Riham: It seems like this disease was the reason he came to Children’s Hospital. We have specialists here.

Participant: Exactly. We spent a month in the hospital there. I will not get into the details of our experience there with the hospital.

Riham: Yeah, you do not need to give me those details because I would like to hear more about what happened when you came here.

Participant: Yes. The doctor from the organization started to follow up with us. They sent my sons medical reports to them. They wanted to reevaluate my son and redid all the exams themselves. They sent him to doctors they worked with.

Riham: Yeah, so they can diagnose it themselves.

Participant: Yes. They examined him and ran more tests and did an MRI and everything. I told them I did not have any money to pay for all these and so they took care of it all. They would come and take him from the morning. They examined him at the university hospital. We said that we were not getting anything out of it. They were just examining him and not giving him any treatment. They said we are going to send him to get treated in America. We sent his paperwork there. They gave us the names of the doctors that were following his case. They said our affairs were all set from before. My son said that if we were going to send him to America, he was going to commit suicide. He said they keep giving me shots and I can not handle going. His mother did not want to go and the other children were saying that they will only do more of this to his brother there. I was in a dilemma. The doctors would tell me that this was my chance to take my son to get treated. I went back to the doctors that were treating him and they told me they had several treatment plans. They said that now your son has reached a stage where he was out of the critical state and halfway to recovery. They said that one plan they had was to remove this medicine from his body and replace it with another one. They said this plan they had was better.

Riham: That is plasmapheresis.

Participant: Exactly. This thing you mentioned, all the doctors have been asking me about it and I wasn’t able to explain it.

(32:01)

Riham: Plasmapheresis.

Participant: Yes! I used to tell them that it was dialysis. They would say if that was the treatment then its one that needs to be done over and over again.

Riham: No, no, that is dialysis. This is plasmapheresis.

Participant: That's what I thought it was and the doctors told me to be careful not to do dialysis because I would need to do it every six months. She told me to come to America for this reason. She told me all this just to encourage me to come here. I started trying to convince my wife and my son about going and explaining the reasons. Sixteen days after leaving the hospital we kept discussing the matter. The doctor was encouraging me to go and come back after he is treated. They said that the plane ticket would be on me though. I told her no. She also said that I needed to take my whole family. She said if you want to come back after three months, you can come back. I went to another doctor and they told me not to go. They said those that are meant to live are going to live here and those that are meant to die will die so just stay here. He said they have the treatments there. This doctor was a professor at the University of Jordan. He said that he has a treatment plan for my son. He said that when my son completes three months --. At that point, my son had completed two months there. He said that he has one more month and he will be in the recovery phase. He said that the same way the illness spread, it will decrease. If it spread quickly, it will need time to decrease. He said this stage needs time. He said he had a treatment plan to do the plasmapheresis--. Please write the name down for me.

Riham: I will write it for you.

Participant: First, my son got a cortisone shot. He also received some other shots. This resulted in his body needing food. He would eat all day and night.

Riham: I don't mean to interrupt you but can you tell me about when you first arrived here. How was the communication with them in the hospital? Did they understand your situation well or did they not communicate well with the doctors there.

Participant: I have not gained any benefit from them so far. The doctor promised that we would have a team of doctors working with my son and that he would have a special chair on the plane.

Riham: Did he have a doctor traveling with him?

Participant: Yeah that was what we were told. It was a nurse that came and left with my son.

Riham: Yes, a nurse.

Participant: When my son agreed to come and they told us that the hospital knew that we were coming, we booked our flights and came. When we stopped at our connecting flight in new york, the nurse said that he was only going to accompany us to New York.

Riham: You had to continue to Cincinnati on your own?

(35:31)

Participant: Yeah, that was the plan. I talked to him and said that I had some papers I needed to give to the medical team. He took them all from me and said to wait for a little because he will talk to the medical team. He said he will continue with us to Cincinnati airport and that the medical team will be waiting for us there when we land. The medical team was there waiting for us and there was also an ambulance. They carried my son and brought him to the hospital. My family was taken by another car. When we arrived at the hospital, that is when the struggles began.

Riham: Tell me about your experiences in the hospital.

Participant: I am about to tell you. This is when our expectations were not met. When we arrived at the hospital, they admitted us and my son was examined. They said he was doing well and did not need a ventilator. It was nighttime. They said we will come see him in the morning. They said they had all the medical papers that the nurse brought and they will go over it. They continued to examine him. The next day they did the same thing. They did not give him anything at all. They did a test on his back --.

Riham: I understand what you mean. It is an LP.

Participant: They drew blood and ran a lot of tests. We spent 3 or 4 days like this. We asked “What is the solution now?” At that point, he was wearing adult diapers. We asked what the treatment plan was going to be. We also requested to go back home if his situation was stable. There is nothing like being home. Since he will not be undergoing treatment then it means his situation is stable. Let him go home then. They said that every day or two he will have an appointment.

Riham: How will you get to and from your appointment?

(38:13)

Participant: Exactly. The caseworker would either take us or some volunteers. So we would go. Sometimes they would send us a taxi that is paid for. One time a woman named Roseanne came.

Riham: Yeah, I know her. She is the one that told me about you.

Participant: She was the first one to greet us and she was the one helping us with these matters. So we started going. They would examine him and do things like urine tests and whatnot. After twenty days they gave him pills. Every day he would need to take a pill. He spent 6 days or a week doing that. It did not help him at all. We went back for a review. They said let's stop these pills and try a new one. He spent a week or 10 days taking the second pill. When we went back for a follow-up we told them that his situation was still the same. The doctor said he will prescribe a third pill for us to try. I stopped him there. I said I wanted to know what disease my son had and what treatment plan he is on. I wanted to know what other treatment plans they will try and what is it that happened to my son, to begin with.

Ahmed: This whole time, they did not explain any of this to you?

Participant: They had not explained anything to me at all.

Riham: Maybe they did not understand. From the little knowledge I have about the situation, I think they had a hard time understanding the case. Right?

Participant: No. We had interpreters to explain the case.

Riham: No, I don’t mean --. As in they did not understand what happened to begin with.

Participant: They knew what happened. They had reports telling them of the vaccine he took and what happened to him. As in they did not know what the illness is?

Riham: Yes.

Participant: Let me tell you something. Cincinnati Children’s Hospital is one of the most known hospitals in the whole world. How did a doctor in Jordan diagnose him? How is it possible that this illness affected only my son and was rare to that level? They did not even explore the possibility that it was Adam’s.

Riham: The system here is a little bit different than the one you might find in your country. When a new patient arrives, they like to start from the very beginning. They don’t want to say a word until they are a 100% sure.

Participant: Okay, but it has been three months. How long will I wait? Two years?

Riham: Till now they did not tell you anything?

Participant: Till now they did not tell me what the problem is or what the report discussing what he has and what his treatment plan is.

Riham: Is a neurologist seeing him?

Participant: Yes he is.

Riham: Did you think of asking the doctors regarding this report that you wanted?

Participant: The first doctor, the urologist, got irritated with me. Usually, after examining the patient, the doctors ask if we have any questions. The translator told him --. What was the name of the urologist?

Participant's Child: I don’t know.

Riham: It’s okay, we don’t need to say any names.

Participant: After he finished telling me about the pills were going to try, he asked me if I had any questions. I told him that first, you told us we were going to try out the pills. Why is my son being experimented on? Secondly, I told him, that I needed a report describing the treatment plan. As I was still talking, he gave me his back and walked away.

Riham: Oh no.

Participant: It was as if I wasn’t even there. I got really angry. Roseann was there. The interpreter was there, our caseworker was there, and there was also another doctor and some nurses there. I lost it. I just needed to know about the treatment plan for my son. I have seven kids that I needed to register in schools. They have appointments too for their vaccines. Every time they would have a vaccines appointment they wouldn't want to go. They would not want the same thing to happen to them.

Riham: Yeah, of course. They are scared.

Participant: They are scared. This woman here said they better not come near me. I had to convince my family. I had to keep trying to persuade them to get vaccinated.

Riham: That means you were dealing with a lot of mental stress.

Participant: You don’t even know what happened to me. I started to have health problems. I also developed a rash. I went to the hospital and got medicine. [indistinct]. My neck from here to there it is all swollen. [indistinct].

(42:58)

Riham: So with these situations that you were finding yourself in and all the stress that you were dealing with --.

Ahmed: Anxiety

Riham: Yes, anxiety. What methods did you use to help cope with all this?

Participant: I would cry.

Riham: That’s all.

Participant: Yes, that’s all.

Riham: Would you write anything down?

Participant: No.

Riham: Would you message anyone?

Participant: I would try to message people in Jordan so that they can bring me back.

Riham: No, no I mean in terms of helping yourself. Do you talk to your brother, for example? Maybe your sister or a friend.

Ahmed: Do you talk to anyone about your problems?

Participant: No, no.

Riham: Is there anything else you try to do? Like walking for example.

Participant: I just cry. I also started to smoke four packs of cigarettes now.

Riham: Each day?

Participant: Yes, each day. I don’t eat any food at all.

Riham: Four packs of cigarettes?

Participant: Four packs and cigarettes and I don’t eat any food. My wife tells me to have mercy on myself.

Riham: Yeah, have mercy on yourself.

Participant: Water, I barely even drink. My son tells me that I brought him here and he did not benefit at all. They just make these appointments for him and he has this training.

Riham: Physical therapy.

Participant: Yeah. He says he did not benefit anything at all. He said you brought me here when I could have stayed in Jordan. The doctor there would have at least done the plasmapheresis. It's been six months now. He could have been cured by now. When this incident first occurred, we spread the news on Facebook. People from all over the world started responding. They were saying things like not to worry about our son because their kid had something similar happen to them. They started saying that your kid will need a month to six months to go back to normal. They said it was something simple and by six months he should go back to how he was.

Riham: God willing. Everyone is different.

Participant: In the Middle East, it seems like they had experience with this. There was a doctor from Egypt that treated people from Syria. They sent their reports to Egypt and they treated them there.

Riham: Do you feel like the doctors here do not have any knowledge of this type of illness?

Participant: If they did they would at least have given me a report.

Riham: I want to go back to this point. When you would ask them to explain something to you, how do they respond?

Participant: They tell me next month.

Riham: I am sorry, what?

Participant: The next follow-up or appointment.

Riham: You also told me that you had a problem with missing appointments. Tell me a little about that.

Participant: I will tell you now about these appointments. I have a list.

Riham: A list is fine.

Participant: I have four boxes over there now and I do not understand a thing about them.

Riham: Does an interpreter help you understand these papers?

Participant: No. The interpreter doesn’t help me. The interpreter only helps with the conversation between me and the doctors. If I ask him to translate for me the papers, he would say this is not my specialty. I am not able to explain the paper, only what you or the doctor say.

Riham: Okay, is there anyone else at the hospital that can help you understand these papers?

Participant: No.

Riham: Does anyone help you in filling out the papers?

(46:32)

Participant: In terms of filling out the papers, the nurse or the interpreter usually helps me. The nurse would tell the interpreter how I should fill them out and the interpreter then tells me.

Riham: You don’t speak English at all from what I know, right?

Participant: None at all.

Riham: Yet they give you papers that you need to understand, however, no one is helping you.

Participant: Sometimes they even make me sign papers I don’t know a thing about.

Riham: How do they remind you of your appointments?

Participant: They don't. They used to call me. In the end, I started to go out and ask people outside. Yesterday they gave me an appointment for my son's foot. My son broke his foot before and it started hurting him. They gave me these so that I could arrange it with the doctor and make an appointment with him. It's been four days and every time I tried to make an appointment, I couldn't. Today I went to the organization and I told our caseworker. He said that he doesn't have our case anymore but since I am a good person, he will do me this favor. He made this appointment for me and wrote it for me in English. I still don't know now. I have to now find a foreigner that speaks English so that they can tell me what is written.

Riham: Oh okay. I understand what you mean. Even if they gave you an appointment, it’s still written in English so how are you supposed to understand it.

Participant: Yeah. In the hospital, they told me --. Let me tell you what they did. They would give me an option to speak to an interpreter if I had any questions. I would call the number given to me for the interpreter and I would tell them that the hospital called me and I don't know what they said so I need help. They would say to hold on for a bit. They would then call the hospital. Then they would come back and ask me which department called me from the hospital. How am I supposed to know which department? I have seven kids. Even the school, the same thing. The hospital calls and I don't know which department, and what the appointment is even for. The other day the schools called. Maybe four times about the dentist or vaccines. I had to go to our caseworker to get information. When he was responsible for our case he would inform me the day before. I'd find out that we missed an appointment and they would say that they called me. How am I supposed to understand them I asked.

Riham: Okay, Abu Ahmad. I am asking you these questions so that we can improve the situation. In your opinion, what can we do as a hospital to help you get to your appointments? Tell me and please include details. Firstly, you want someone to speak to your in Arabic.

Participant: Let me just tell you this. The thing that we want is --. Let’s say I come to America and I am greeted by a team of doctors at the airport along with an interpreter. If one doctor sits with me and gets to know everything about me, I don’t want another one to come after and have to repeat everything.

Riham: This is the system here.

Participant: Yes, this is your system. The same way I have a specific doctor for me, I want an interpreter that specifically takes care of our appointments. If we have an appointment then he would call us to inform us about it.

Riham: Oh, okay. One that is specifically assigned to your family.

(50:24)

Participant: One time they brought me an interpreter that speaks Arabic, but he was not able to understand me. He was Moroccan.

Riham: Oh, a Moroccan.

Participant: Moroccan or Libyan.

Riham: Libyan is difficult to understand.

Participant: I told them this guy does not understand me. He speaks Arabic but doesn't understand me. How is that possible. There is the modern standard Arabic. He should at least understand that. For example. If my doctor would walk in, the interpreter would come in with him. Once the doctor is leaves, he starts to walk out with him too. If I tell him to stay a little, he tells me he is not able to. I would say okay then keep the doctor here and explain this to me. He would not do it. The other day the doctor told me like I told you --.

Riham: Let me better understand this. When they call you, they should speak to you in Arabic. Secondly, they should not only speak in Arabic but in your dialect.

Participant: No, no. It doesn’t have to be my dialect.

Riham: I mean at the very least, a Syrian dialect.

Participant: Syrian or at the very least they understand Arabic. If he says something and I ask him what was that? He says I already translated and we are moving on now.

Riham: What?

Participant: Sometimes the words get choppy. My phone signal is choppy for example. He would then tell me that he will not repeat himself. Let’s say Dr. Ahmad here speaks to me. He might say the 7th of March. I might ask, what is March? He would say I already told you March and you should go figure out what March is. Tell me which number month it is.

Riham: I am the same way. If you were to tell me the names of the months in Arabic, I would not know them.

Participant: If he were to say the month in Arabic, I would know it. However, if he says the 5th of March, how am I supposed to know what March is. If they would say for example --.

Riham: June for example.

Participant: Yes, June. What month is June? It’s the sixth month. He should just tell me month six.

Riham: Yeah. Let me make sure I understand this. The interpreter also doesn’t know --.

Participant: The interpreter is like a parrot. He just repeats what he hears.

Riham: Yeah, for example, my Arabic language isn't that strong, right? I don't know the name mayo and what not. If you were telling me, I would want to know which month number it is. That means the interpreter needs to be able to explain things to you to make it easy for you and not more difficult.

Participant: Exactly. Today, the doctor was telling the interpreter that for example, this young man got sick on the 26th of November. The interpreter should not be repeating the word November. He tells me, "November "so I asked, "what is November?" He should say, "it means the 11th month." He only tells me, "November".

Riham: Yeah, what is up with that?

Participant: There would be times that I would ask what is this appointment. He would respond in English. I would tell him I am an Arab so tell me in Arabic.

Riham: That means they are not really smart.

Participant: It is not a matter of not being smart.

Riham: Abu Ahmad, a smart interpreter would take this word and they can either read it to you in English or translate the meaning of it into Arabic. The one that is not smart will tell you the word in English because he does not know the translation of it.

Participant: Yeah there might be some words that they are not able to say in Arabic. They would be Arabs but they would have been living here all their lives.

Riham: That means, Abu Ahmad, that even though I am Arab, my language isn’t that strong. I used to observe the interpreters and even I would notice that they are not that good. For example, I was in a room. I have a card here that says that after a month I will be able to speak in Arabic with the patient. Prior to getting this card, they would not permit me to speak in Arabic. What would happen is that I would be with a Syrian patient. We would be able to understand each other but they would bring me a Libyan Interpreter and then neither us would be able to understand anything. They would not let us speak directly. This is a problem that they have. I wrote them a letter saying that I am standing there and watching two people unable to understand one another. An American doctor would not know this. That is why this research is important. I want to hear your thoughts and take them back to them. There is a clear problem with the interpreters.

(55:21)

Participant: I also have a lot of appointments that I missed. I would make a schedule of my appointments. What would happen is that they would call me to tell me the appointments have been either canceled or changed. They would give me a paper and I swear to God I have no idea what this paper says. This one too and that one. Today I went to the organization and they translated this one for me. They told me that I missed a lot of these. My son here had four of his appointments canceled. I started writing this. Do you see how I started writing notes here?

Riham: Can I ask you a question? Please do not take offense. What was the system like in your country? I was born here, so I have no idea. Is it a similar system to what we have here or no?

Participant: In our country, they would tell you that you have a follow up on so and so day. There is something good that they do in our country. For example, they would tell you that you had an appointment on the 16th. You would memorize it and that is it. There would not be any changes or cancellations. If a doctor was not going to be there, they would put another one in his place. The new doctor would have the file with him with all the details. They would not change your appointment. There is another thing that they do. If you do not go to your appointment, you do not miss out on it. They would give you a chance to talk to them and reschedule. Here they call and talk to me in English. Why would they speak to me in English? When I first arrived a computer used to talk to me. I would say I don’t know English, only Arabic. It’s a computer though. I would keep repeating “Arabic, Arabic”.

Riham: An automated message.

Participant: Even with the text messages. Let me show you all the messages. I would reply, “No English, Yes Arabic”, but no one would respond to me.

Ahmed: The suggestion you made earlier was a good one. That an interpreter is assigned to each family.

Riham: A family interpreter. Your family would have a specific interpreter.

Participant: Yes and he would know about our appointments. He would be the one to call us and what not. When I go to my appointment in Millville, I will have an interpreter there. When I go to the hospital, I get a different interpreter. I am not able to tell the interpreter about all my problems from before. If I had one assigned to me, he would know our background. He would have a complete file for me. Every day they bring me someone new.

Riham: Okay, so tell me what you think about this idea. Let's say a Syrian family comes to America. How about they assign to them a caseworker that speaks Arabic and an interpreter.

Participant: We have that already. Some families have Arab caseworkers.

Riham: Yeah, but you do not have one.

Participant: Mine is Nepalese. Let me tell you something. It is wrong but that is it, I am going to say it. The Nepalese caseworker was the reason we missed our appointments, to begin with. When they would make our appointments and contact the Nepalese, he would get some help from the Arab caseworker there. He would tell her about our appointments. When he gets other people, he would help them more than he would help us.

Riham: How do you know this?

Participant: I saw him. That other time he made me miss an appointment because he had to greet people at the airport. He sent the Arab woman to come take us. The Arab woman also has her own clients. He sent her to take me to the hospital for the appointment but she said that he might be the one to bring me back. She took me there and talked to them about my appointment. They told her that they had rescheduled this appointment. She said he has two kids and only one of their appointments was rescheduled. This was a Friday. I missed out on Friday prayer, honestly. We kept waiting there for two hours till he finally came. One time he took my son from the morning and threw him in the hospital. He stayed there until the evening and no one cared for him at all. He went to the interpreters there

Participant's Child: He did this to me three times.

Riham: Three times?! Would he just leave you at the hospital?

Participant's Child: He would leave me and tell me that another car will come pick me up.

Participant: A volunteer.

(61:14)

Participant's Child: He would take me and maybe later pick me up. In the meantime, I would be alone there and I would have to go speak to the interpreters to arrange everything and my ride home.

Participant: One time he came home in a taxi cab. We are here thinking he was in good hands. I am sorry but I am going to have to say this. He wet himself like four times and no one helped him clean up nor cared for him.

Riham: This whole time he is in the hospital waiting.

Participant: He is just waiting.

Riham: That is so wrong! It is unacceptable. We’re talking about a sick child that is being treated this way. The problems I am hearing are with the language and transportation. What else is there?

Ahmed: We did not really finish the conversation regarding the transportation. How were you getting to your appointments?

Riham: He is telling you.

Participant: For the transportation, we used to get volunteers. We also met a few Arabs that wanted to help. They told us about the bus system but it was very hard to use. You wouldn’t know which bus to take. We weren’t able to resolve this issue and use the bus system. We had people that have been living here for a long time. They were Arabs and offered to help with transportation when they were available. One might say that if I was in the hospital and did not find a ride home, I can call him and he will send me a car.

Ahmed: Who would pay for this?

Participant: The hospital would pay. It is covered by the insurance.

Ahmed: Okay.

Participant: We get up to 15 rides. However, the problem with the doctors here is a big problem. The Arab doctors were used to know a lot more about the illnesses and right medications needed. The reports here are terrible. They just give me some papers based on my visit and it would be in English. They know that I am an Arab so why are they giving it to me in English? The reason for the visit and all the medication and treatment that was provided is on these papers. Yesterday I went to the neurology department. I had an MRI done. They drew some blood as well. I did all these things but when I left, I did not understand what happened to me. They just gave me some papers and told me to leave.

Ahmed: They didn’t give you anything in Arabic?

Participant: They never give you anything in Arabic.

Ahmed: Even for Ahmad?

Participant: Not even for Ahmad. They didn’t give me anything in Arabic. The interpreter there should at least explain to me the reason for the visit. Why can’t he write it in Arabic and give it to me? I am an Arab, I don’t know English. Why would I be put in a situation where I had to go and urge someone else to translate it for me. The other day I went to an Iraqi that fixes tires. He tells me that the next day I had an appointment for my son. He told me that the papers were telling me the reason for the visit and the treatments provided. There was also a notice on there that my appointment was for the next day. I don’t understand why these papers weren’t in Arabic. I need it in Arabic so that I can understand. How is that a difficult thing to do? Just print out a copy in Arabic. Look at that whole box I have over there. It is filled with medical documents that I do not understand at all.

Ahmed: Those are all in English?

Participant: Some are ones that I got in the mail. Some are for rescheduled appointments I think. This one right here might be an appointment.

Ahmed: No, this one is just telling you that there are some prescriptions that you should pick up from the pharmacy.

(66:01)

Participant: How am I supposed to know?

Riham: Abu Ahmad, I am going to ask you a few more questions pertaining to the emergency room. Have your kids been to the emergency room?

Participant: Here?

Riham: Yes, here in America.

Participant: No.

Riham: Okay. You also told me before we turned the recorder on that when your son got a fever and the other one --.

Participant: Honestly, I don’t like taking them to the hospital for those reasons.

Riham: Why not?

Participant: It's a lot of trouble. A lot. My son had a fever and my daughter's stomach was hurting her. From the day we got here till now, she has been having some stomach pain. When they were making her appointments for the vaccines and the dentist, we told them about her stomach pain when they asked. The first time, they gave her this heated bag to place on her stomach. The next time they gave her two pills and told her every week to take the vitamin D pills. In our country, they would have investigated what she ate and asked more questions to find out the cause. Here they did not care about her at all. When we went for the vaccines, they were asking questions before giving them to us. I told them of all my issues but they said since I don't have a fever they can just give me the vaccine. If I complain of a headache, why not send me to a doctor to further examine me?

Riham: You feel like it's hard to --.

Participant: When I heard in Jordan, from the doctors and the hospital, that the medical care system here was great, this was not what I expected. I did not imagine it to be like this I imagined that from the moment we arrived my son would be taken to the emergency room immediately. There, I imagined that he would be met with a specialist and have all the necessary tests done. I thought that within an hour or two, we would get all the results. I expected to get the medication needed and be sent on our way.

Ahmed: That wasn’t what you experienced, right?

Participant: It was the exact opposite.

Riham: Yeah. Tell me about your other children. I know that your son Ahmad is being treated at Cincinnati Children's. Are your other children coming --.

Participant: Tamer is. They gave him a shot --.

Riham: Just Tamer?

Participant: Just Tamer. Tamer also has something I would like to talk about. When Tamer got the [indistinct] shot, the same thing happened to him so they started to treat him.

Riham: How old is Tamer?

(68:58)

Participant: He is either 14 or 15. He stopped going to his appointments with them. It’s been four or five now that he’s missed.

Riham: Why? For the same reason then? Since you are not being informed of everything.

Participant: I am not being informed of anything. Another thing I want to share is that the other day I was sick. I went to the hospital because a rash was developing on my hand. I went at around 1 pm. I had an appointment that day for my son at around 4 pm. The appointment was at the place next to the Children's Hospital.

Riham: Oh the University of Cincinnati. The UC.

Participant: Yes, the UC. I was there from 1 pm to about 5 pm.

Riham: At the emergency room?

Participant: I was in the waiting room till about five o clock. My son had an appointment at 4:30 pm. How was I supposed to take him? I asked them if I can go and bring my son to the hospital, and come back. They said if I left [indistinct]. My son ended up missing his appointment because of me. God sent me someone that I was able to tell this to. I asked him “What should I do.” He said to call them and reschedule the appointment. He called and told them he was my friend. He took my name and information and started to give it to them. Then they started to question him. He said he was only trying to help.

Riham: Were you able to reschedule the appointment?

(70:43)

Participant: Yes, I changed the appointment.

Riham: If that man wasn’t there to help you, you would have missed the appointment.

Participant: I would have missed it.

Riham: I have another question for you. Do you have a number you can call if you need to reschedule an appointment?

Participant: I don’t know how to. I do have a number. However, I do not know the details of my appointments.

Riham: I see so the difficulty is in knowing the details.

Participant: If I need to call an Arab to help me, I am going to have to give them all this information. It’s easier to just call the hospital directly. Even if I spent four hours talking to you, I will still have more to say.

Riham: We know. If we can get your story heard --.

Participant: It’s true that I am not educated but I have so many great thoughts and ideas.

Riham: Yes, that is why we want to hear them.

Participant: May God reward you both for taking the initiative to do this research. This could save many families.

Riham: God willing.

Participant: A lot of families. I am about to tell you some more things. If only there was an interpreter that called me everytime I had an appointment. If every call was a dollar, I would pay a dollar. I would pay a dollar or two per call because this would really make it easy for me. It is better than going and taking a cab and begging other people to come and take me. That other day a woman came to me. People, in general, come over from time to time to see what we need and how they can help. We showed her our papers because she knew Arabic and English. She went through them and told us what to discard and what to keep. This woman stayed here from 6 pm until midnight helping us. This is very wrong of this hospital to do this to us. She said they were here to support us and stand by our side. She organized these piles for us. I haven't seen this woman in over a month. I don't know what she has written on some of these. Some of them would have numbers inside to call for help but when we call it is all in English. I try to request to be transferred to the Arabic line. They would ask who are you and when I told them my name they wouldn't know me. Imagine we did this interview once and I never see you again. That is how it is. They have a different interpreter each time. How are they going to know me? This is a problem, especially in the Children's Hospital. My son told me that it has been two months now and he has two sessions a week.

Riham: You mean his physical therapy.

(74:52)

Participant: Yeah, the building behind the hospital. He finished the first week. Of course, there was a huge mix up with the appointments. They would reschedule or cancel them. Sometimes he would go and sometimes he wouldn't go. He started to say that if he was able to stay at home and bring the machine here, it would have been better for him. He is going through too much trouble with them.

Riham: Did you try to tell them that you would like someone to visit him at home and help him?

Participant: Yeah, but how does that work? Someone from the hospital will come here?

Riham: Yeah, in some cases they come.

Participant: Yeah, they come for the patients that are unable to go to the hospital. I have a friend named Abu Muath. He has a son that is really ill and he is bedridden. They would send the medical team to him at his home.

Riham: It is because Ahmad is able to move around, thank God.

Participant: He is finding difficulty in the fact that he has been going to physical therapy for two months now and has not benefited at all.

Riham: Yeah, it can take time.

Participant: Yeah, it takes time. They told him that he needs to set his appointments from now. They said I have one on this day and that day. Yesterday, I made an appointment for the dentist on the 23rd of May at the same time. How am I supposed to deal with this now? They should have it all in their system. They should have my name and show all the appointments that I have so that they do not conflict.

Riham: Yeah, they need to correspond.

Participant: There needs to be more communication between the hospitals. It should show in their system that this man has to take his son to his appointment on the 23rd at 4 pm. How am I supposed to make it to my appointment on the same day at 5 pm?

Riham: Yeah, the system here does not work like that.

Participant: They said no, his mother should take him. Well, his mother has little children.

Ahmed: I would like to quickly talk about a topic before we end. We heard that your old house really affected your mental and emotional state. Can you tell us in a few sentences how it affected you all?

Riham: Not just how it affected you but also how you were able to save yourself from this experience. If others had been in your situation they might have attempted suicide, for example. Tell us a little about how you felt and how you were able to save yourself and your family from this traumatic event.

Participant: Firstly, we all suffered from depression and fear. Even the children amongst themselves --. One of my younger kids, his name is Mulham, he started to say strange things. His mother would say something is wrong with this child. Something unusual was happening to him. There would be times would he would yell so much. Other times he would break and hit things. He was used to going out and felt like things were different. I used to open the door for him and stand with him so he can step outside. This would be during the day. He would run in the street and we wouldn’t be able to catch him. He wouldn’t want to come back in. We would all have to chase him. His mother was saying how is this right?

Riham: They don’t have a park to play in?

Participant: What park?

(78:31)

Participant: We had an open field and a porch made out of wood. We weren’t able to go sit on this. Our neighbors had dogs and there were some kids --.

Riham: You mean it was dangerous. He was not able to play outside.

Participant: No, he wasn’t.

Riham: A child needs a park, they need --.

Participant: When we would catch my son to bring him back in, he would hit himself twice. When we told the organization this story, they said they will bring him a therapist. He said he will refuse to sit with anyone.

Riham: Okay, when we end, God willing, I’ll give you the number of a woman that I heard about. This isn’t my field but I heard that she is really good with refugee children. Just give me a few days and I will bring you her contact information, God willing.

Participant: God Willing.

Riham: He needs to see a therapist. These things that these kids have seen are affecting their psychological state.

Participant: These thing that we have seen are causing us to fight in front of our kids. The kids have gotten used to the fact that if we are all at home, we would be fighting constantly. Fights would happen between the mother and the kids, and the kids with each other, and the kids with me. My son right here started smoking. This other son right here wants to start doing hookah. His friend would invite him to come do hookah with him. There is a lot of pressure that they are dealing with. We don't want to push them to their limits. They are on the verge of losing it. This young one right here is battling depression. He is really depressed. I found his skin all scratched up.

Participant's Wife: He puts pressure on himself. He opens and slams the door. He picks things up and throws them.

Participant: From all the pressure.

Riham: What are somethings you are trying to do to help them cope with all this pressure? Also, what tools do you wish you had to help them cope.

Participant: Honestly, I am not able to even think about this matter or attend to it. The most thing that has been on my mind is how to go back or even go to another country. To Jordan, Egypt, Lebanon or Turkey. A country in which there is freedom. You do not have freedom. I have not seen any freedom here, I have only seen a country that complicates life. I am even struggling with the language. I am not able to comprehend anything.

Riham: Just be patient a little more. You have only been here for a few months.

Participant: I don’t even know the alphabets or the numbers. Till now I can not say the numbers. I only know how to say thirteen and six.

Riham: Just hear me out, my brother. We can help you with these matters.

Ahmed: This is Roseanne's contact information.

(82:11)

Riham: Hold on to this number. Roseann is really good, I know because I work with her. It’s her address and number.

Participant: Okay, this is her number and name.

Riham: In your opinion, what programs are needed for the children. To help them with their emotional state.

Participant: We honestly do need things like that.

Riham: Yeah, so that they are able to play, and open up, and write.

Participant's Wife: To be able to calm down.

Riham: Yes, to be at peace. What do you think about this topic?

Participant: It is a good idea.

Riham: As you said, the problems with their identity have an effect on their mental state and their mental state affects their physical state. A state of peace and healing --.

Participant: There is something else I would like to tell you. My son when he was being affected by his health and it was affecting his brain he would say strange things. Sometimes he would ask for a lollipop and other times he would say he was a man and wanted to get married. He was never like that. Out of respect for his mother, he wouldn't say some things. I asked him yesterday if he wanted to hit me. He said he wanted to hit me in the head. Is there any cure for him here? We did not benefit at all from this hospital. We did not see any progress in terms his bladder problems or his feet or his mind. I came here and what did I get from it? All I want is a medical report. I want to update the doctors in Jordan. My mother and sisters keep asking me what happened to my son. I would tell them nothing happened at all. They would ask what he was given and how he is being treated. I would respond and say he was still the same way. Then why did they deceive you into going they would ask. The Jordanian doctor promised to do the plasmapheresis and a list of other treatments. He would have been in a much better state than this. I didn't find the medical care here we expected.

Riham: God willing, we will pass on all your thoughts. It will need time.

(84:52)

Participant: That is your problem. This is the problem in all of America. You like to take your time. Everything has a long process. The other day, I had smallpox. The other day, he and his brother had [indistinct]. He fell down the stairs and wasn't able to feel anything. We called 9-1-1. They called me from the organization to find out what happened. I told them to take him to the hospital. Why are they contacting me? The caseworker came and the manager. They took him and I don't know what they did to him because I wasn't there. After that incident, I did not receive any information about it.

Ahmed: They did not give him a report or anything?

Participant: Nothing.

Riham: It seems like a misunderstanding. No one is explaining anything to you. Please excuse me, I have work.

Participant: Who am I supposed to tell this to. I don’t have a doctor that understands. I don’t have a caseworker. I don’t have an interpreter. There is something called getting it off one's chest. I want this to get to everyone involved.

Riham: God Willing.

Participant: These matters need to be heard by the people that can provide help with them. This thing you are doing made me really happy. It made me feel like no matter what illness we have, we will manage. I wanted to pull my hair out just to be heard. If every month or two you could visit it would help. Even for my son Ahmad. If an Arab doctor would stop by every now and then to check up on him, it would help. Maybe he is not coping well. Maybe he is being aggressive and not taking his meds. Someone needs to check up on him. My son is telling me that he is taking his medicine. I have to give it to him myself because I fear that he will lie to me. The other day he told me that he will stop taking his medication. I asked him why and he said because they are deceiving us. He said they keep delaying his follow up appointments`. The doctor should be coming here to check up on him or maybe a therapist. Even if doctors like you that are doing research come and visit Syrian refugee families, it would be good. Maybe they are not eating or maybe they are ill. People are not aware of what we are going through. My son sometimes comes back from the hospital with papers and asks me to explain them to him. I ask him what happened to him and he doesn't know. In that case, I need to always go with him. Sometimes I can not go. I need another paper for the organization stating that I need to accompany my son to the hospital. Yesterday he told me that he is going to cut my food stamps because I am not showing up to the organization. I tell him I have kids that I need to take to the hospital. He says I need to bring him a letter from the hospital. How am I supposed to know that I needed to bring a paper from the hospital?

Riham: I pray that your situation gets better.

(5A part 2 2:29)

Participant: You two were sent down as a mercy from God to us. We were able to get so much off of our chest. At first, I was wondering what I was going to say during this hour. Now I realized that even if I were to speak for 10 hours, I would still have more to say. There are so many things. I am sure when you walk out of here I am going to remember some more things that I should have said. This is important because now I am able to share my burdens and worries with someone. The pain and suffering of my wife and children along with our experiences. We shared the struggles we had with the doctors, the cars and whatever else it was. The most important topic is the practice of medicine.

Riham: Yes, the most important thing is health.

Participant: I came to America from Jordan for the sake of an illness. How can I be let down? If anything happens to my son, it will be America’s fault and all the doctors here. Yesterday, I was speaking about a topic that wasn’t right. There are people here with similar stories. They just want someone to talk to. I heard about America and this hospital, and it truly is a magnificent hospital. They should be empathetic. It doesn’t matter which country they are from, they should be more empathetic with the families. The medical aid is the most important. Thank God for you and Dr. Riham you are caring about the suffering of others. Look how God works. I went to check on him and I found her [Dr. Alwan].

Riham: You walked in and found me.

Participant: This is not a coincidence.

Riham: Everything is written.

Participant: I honestly, have a lot of faith in God. I pray at night and during the day. I cry to God. I ask God if it is good for me then take me out of this country. I could not take it anymore. I had no one to vent to. I didn't find anyone to tell all this too. I didn't find anyone that would empathize with what I am going through. There was no one to give me direction. Consider me someone crazy and help me find my way. They come up and say so much to me but I do not understand a word. If I had access to an interpreter --.

Riham: I am going to stop the recorder now.

Interview 5B

Riham: Today is May 19th, 2017. I am Riham and I have Ahmad here with me. We are with a mother of seven children. The participant number is 05B.

Ahmed: Um Ahmad, how long have you been here in Cincinnati?

Participant: We have been here for exactly three months and one day.

Ahmed: Thank God for your safe arrival.

Participant: Thank you.

Ahmed: Can you tell me about the reason for coming here to Cincinnati?

Participant: We came here to get medical treatment for my son Ahmad. We were refugees in Jordan. Immigration contacted us and wanted us to get vaccines. We took the vaccines and two days after taking them, my son Ahmad got sick. He got sick from the vaccine itself. He became paralyzed. We took him to the hospital there in Jordan. After about 16 days, the immigration organization started to contact us. They came to visit us in the hospital after Ahmad was no longer in critical condition. They came to visit him in the hospital to prove that it was not from the vaccine. They wanted to bring us to America for this reason.

Riham: How old is your son?

Participant: 18 years old.

Riham: May God protect him.

Participant: Thank you.

Riham: We heard his story before speaking to you so we are going to ask you questions that deal with more of how you have been feeling rather than his condition.

Participant: God willing.

Riham: God willing. Okay so tell me about when you first arrived here. Your son went immediately to the hospital and you came here with the rest of your children.

Participant: Yes.

Riham: How were you feeling during that time? Did you go to the hospital at all? Participant: No, he went with his father straight from the airport. Since he was unable to walk and he was using crutches, they took him in a wheelchair. I took the rest of the kids to the house. When we got to the house, let me tell you how we felt. It was a strange feeling. We felt loneliness and alienation. Thank God.

Riham: Thank God.

Participant: We were not able to talk to Abu Ahmad [her husband] for four days. I did not know what was happening with him or anything. Thank God.

Riham: During the four days you were there alone, did anyone come and check up on you?

Participant: Some people came and checked up on us. After four days of trying to reach my husband and son, a Syrian young man came. He took me there to the hospital. I was able to see my son after four days.

Riham: During these four days you felt like you did not know anything that's happening.

Participant: Yeah, I did not know what treatment he was getting and what his diagnosis was. Till now we do not even know the diagnosis.

Riham: Tell me what that means.

Participant: That means they have spent three months examining him and have still not diagnosed him. We did not benefit at all [from them]. Till now they did not give him any cure. They take him sometimes to get physical therapy. Thank God. They told us that this disease spread quickly but it will need a longer time to cure it. We have still not gotten any results. His eyes have been hurting him. They gave him medicine and it seems like that is the reason his eyes are hurting him. They turn red and itch him. He would suddenly get angry. He gets tantrums and the other day he hit the floor out of anger.

Riham: Did he used to get angry like this before this illness?

Participant: No. There was nothing wrong with him. If it weren’t for that vaccine shot he would not have been paralyzed like this. He used to be a normal person.

Ahmed: How did your children’s mental wellbeing change after coming here?

Participant: Yes, it changed.

Ahmed: How did it change?

Participant: In many ways. Feelings of hopelessness and loneliness. You would find them locked up in this house. The appointments for the schools are conflicting with the hospital appointments. Their psychological state changed a lot. It is frustration [hopelessness]. The whole family feels this way. Even the younger kids started fighting with one another due to the prison they are in. I don’t know.

(5:56)

Riham: Why do you feel like it is a prison?

Participant: Because we were in a whole different world and we came to a new one. We had freedom and the kids were able to go out as they please. If a child would get a fever, you could go and find a doctor close by. You would be able to speak to him easily and understand one another. Here we are really struggling with the translation. Even with an interpreter, we still find it hard to understand them at times. He is not able to help the doctor understand us nor help us in understanding the doctor.

Riham: Why? Is it because of the dialect?

Participant: No, it's the whole speech itself. If we were able to talk to the doctor directly, it does not compare to the word or two that is used to sum up the conversation.

Riham: So you feel that your speech is getting cut off?

Participant: I don’t know. We don’t know the language but most of what we say --. Like if we were to say that the boy has a fever, the doctor just replies that he will heal on his own. Twice I took my son because he had suddenly gotten a fever. The doctor would tell me that he will heal on his own. He had a fever, and would not eat and kept vomiting. He is clearly ill but the doctor would tell me he will heal on his own. She would say if it gets worse than this, then bring him back. “Call the ambulance and go to the emergency room”, she would say.

Riham: This is your son that is 10 months old.

Participant: Yes, this little one. It’s wrong to do this. I have three children under the age of five. Sometimes it’s just a matter of a cold breeze that makes them sick. We are not able to go and speak to the doctors in the emergency room. I don’t know.

Riham: You have seven children and you have experience with doctors because your kids would get sick from time to time. In your opinion, is the system here different than the one in Syria and Jordan?

Participant: Yeah, it differs a lot.

Riham: How does it differ? Tell me.

Participant: From all aspects. Firstly, in the Arab countries, the doctors are close by. Each area has a hospital or a clinic. Here we only go to the hospital. There they are nearby and the clinics. The dentist could be found right next to it as well. We would find any specialist we needed at their clinic nearby. We don't go to the hospital unless a child has an urgent matter. Here, everything is in the hospital. This is wrong. We feel like there aren't enough doctors. They make us an appointment with the neurologist at the hospital. If we don't understand them, then we miss the appointment. I just missed my appointment with my primary doctor. I have a “disc” in my back. It's preventing me from helping my children at home. Many days would come where I would not be able to handle it and I would have to sit from the pain. I have [indistinct]. If I move my hand like this, I can't feel my neck and my hands would go numb. Apparently, I had an appointment on the 8th of the month that I missed. No one came to take me there nor did they remind us of it. The guy responsible for us did not tell me at all about this scheduled appointment. I missed it. He only told me after I missed it.

Riham: He doesn't tell you beforehand?

Participant: No, he didn't tell me. When I was going to see a gynecologist, I told them that my back was hurting me so they gave me a paper. I gave him this paper and told him it's an appointment. That way he can write it on my schedule and call to tell me. He did not call or tell me or come to take me.

Riham: You are finding trouble with the specialists then. You were able to see your primary doctor but not the specialists.

(10:29)

Participant: Yes. Even the primary doctor. I wasn’t even able to go there and ask for a referral.

Participant's Husband: She would go to any of her appointments. Like she would go to her dentist appointment and complain of her back, for example. That doctor would write down for her where she should go. We would wait until any upcoming appointment to talk about our pains and illnesses.

Participant: That is true. There are many times that we experienced pain or an illness --. My son Ahmad went to the hospital once. He had pain in his molar teeth and gums. So we took him to the doctor to follow up on his illness with his feet and his paralysis. We told them that my son was in a lot of pain. He was not even able to sleep from how bad the pain was in his molar tooth. His gums looked bad as well and were swollen. He was in so much pain and could not bear the pain. They tell him to make an appointment with the dentist. When the guy that was with him made him an appointment at the dentist, the appointment was going to be after 20 days. This is with him being in a lot of pain. How was he going to handle it for 20 more days? We came here and gave him a pain reliever that we brought with us from Jordan! He was still crying all night long even with that pain reliever. He tried putting salt water on them. He spent three days crying from his tooth pain.

Riham: What are some other home remedies that you try to help your kids heal? You mentioned the salt water. What else do you use?

Participant: For example, he used warm water and salt and gargled with them frequently. It made the pain a little bearable. For the younger kid, I might boil some anise seeds or cumin and put it in a bottle for him to drink. I use a cold compress on their heads when they have fevers. My daughter frequently experiences stomach pain so I give her some mint tea. We would help them manage with these remedies because we lost hope in getting them treatment at the doctors, treatment such as a pain reliever or medicine for their illness. The younger kids, I always give them mint, anise seeds, or cumin as well. I get herbs and boil them and give it to them to drink. When they get fevers I use some cold compress and give them herbs so that they can heal from the fever and cold. I give them a bath in warm water when they have a fever.

Riham: Are these the same things you used to use while you were in Syria or did you start relying on them more here?

Participant: No, I started to rely more on them here. In Syria, if we felt a child was a little ill, we would take them to see a doctor immediately.

Riham: How would the doctor treat them?

Participant: He would give them a shot, a pill, or medicine to drink. They would examine them to see what is causing the fever. If it is their tonsils, then they treat them. If they had a fever they would give them a pill or liquid medicine. They would treat them there. Even if I were to come the same day and my child was feeling ill, they would give them an IV and keep them there for an hour or two until they get better. This is at the clinic. Even they saw the child with infected tonsils or a fever they would give us some medicine to start with and then ask us to bring them back the next day so they can give them a better type of medicine.

Participant's Husband: Or they would transfer them.

Participant: Or they would refer them to the hospital and keep following up on them while they are there. It would be a hospital that this doctor is assigned to, for example. He would come and check up on the patient there.

Riham: He would check up on him.

Participant: He would follow-up on his case closely. They would just refer them to the hospital if a child needed an IV or a mask to help with breathing, for example. Here they don't do that. I have a child that suffers from asthma. Every time I would take him, they would give him some oxygen at the hospital. Here they do not have this thing. If a child gets sick or gets a fever, we have to treat them at home and bring their temperature down and not the doctor. We aren't able to easily communicate with them, to begin with. The problems with the language, and the appointments, and the rescheduling of appointments. We don't know when a child is going to get sick. How are we supposed to make an appointment in advance if we don't know when a child is going to get sick? This is not acceptable. All these are things we are struggling with.

(16:31)

Ahmed: I just want to focus on this point that you mentioned. It seems like most of the difficulties that you are going through is because you have lost hope in finding your kids the right treatment from doctors or hospitals.

Participant: Yeah, we got too tired of it. We started to deal with it at home because it is easier on us. Most of the time we would take them for a fever, they don't even let them in. They say go --. , if we took someone with us or we had an interpreter, they would say take him back home and make an appointment first. The soonest appointment they would give us would be 10 days or so. The best case scenarios were after three days. That is if Allah cured him of the home remedies we gave them. If they had a fever we would use a cold compress. If he had diarrhea we would feed him yogurt and garlic. We might boil some potatoes. Just like that. We would deal with it a home.

Riham: Earlier, you said that you lost hope in life itself.

Participant: I said that because I was feeling alienated. It was really hard during these first three months. We really suffered. Especially with not finding proper medical help for my kids. I have a lot of kids. Each one needs to see a doctor and we can not keep up. We got really tired of it.

Participant's Husband: We felt frustration [hopelessness].

Participant: Yes, like I told her. My daughter is feeling frustrated [hopeless]. All of us suffered psychologically. We did not experience this level of hopelessness after the war the way we felt when we came here.

Riham: Why do you think that is so? Why are you experiencing more frustration now than you did in Jordan?

Participant: Here, the stress with the appointments and the lack of proper medical care caused it. We had high expectations. We came here for this reason. We brought our son here in hopes of him getting proper treatment and to be cured of this illness. This is the main reason for migrating here. We did not get anything out of it. We did not see proper care from them. We could say that it is okay regarding the younger children. We could deal with that. However, when it comes to a more severe illness --. I know that the illness was due to what God wrote for us, but it was also due to a shot they gave him. We did not see proper care for him. This is not a good situation.

Riham: In your opinion, what does proper care look like?

Participant: We did not see them help him with his bladder issue or follow up with him closely and give him proper treatment. He has been suffering from this for six months. Three months in Jordan and three months here.

Riham: Can you explain to me what you mean by bladder?

Participant: The bladder, I mean his ability to urinate.

Riham: You mean so that he could urinate on his own?

Participant: No, I mean it is out of his control. He cannot hold it.

Riham: Oh, okay. You mean urinary incontinence.

Participant: It happens during the night and day.

Riham: Due to the illness.

Participant: Yes, from the paralysis. It happened to him first. He first had urine restriction then he became paralyzed. The next day his feet became paralyzed.

Riham: Excuse me, I do not mean to interrupt you but you said he was first crawling, and then he started using the crutches. Now, I am seeing him walk. Thank God he is walking now but he still not walking like a young man who is 18 years old but at least he is walking. In your opinion --.

(21:22)

Participant: It is not like any other young man. Yesterday he was about to get in the car --. His right one, is almost completely healed, thank God. His left foot went like this. I started crying when I saw it. When we tried to get in the car he held his foot like this and put it in the car. He used his right one to help him get in the car. He is prevented from living a life like other boys his age. When he would see his friends playing ball, it really bothers him. He is not able to run with them and play ball.

Riham: It is still early.

Participant: Thank God. If only there was proper care here. They made us travel from Jordan to come here just to get medical care. We have not found it here. We did not find any medical care, especially for him. They did not care for him at all.

Riham: I want to ask you the same question again because everyone has their own opinion. Describe to me what proper care means to you.

Participant: It means showing interest.

Riham: How can one show another person that they care or show interest?

Participant: By giving him treatment and following up with him. It’s been 20 days since he has last been to the doctor. They postponed his appointment. The neurologist postponed his appointment to the 23rd of the month.

Riham: You mean to see him with a short amount of time in between appointments. Not to keep him waiting for long between appointments.

Participant: Yeah, every 10 or 20 days, the neurologist sees him. It has to be a shorter time.

Riham: Okay, a shorter time.

Participant: One week he should see the neurologist and another week the urologist. His problems are neurological, urological, and internal medicine.

(23:23)

Riham: Okay. What do you think they can do to improve and make you feel like they are providing proper care for your son?

Participant: They can get us the MRI results and other test results quickly. Currently, they wait a month to get the results and give him medicine based on the results of tests they have done a month ago.

Riham: Okay so to shorten the time between the exams and the results.

Participant: Right now they did an MRI and ran some tests but did not explain to us the results. They did not give us a report or a diagnosis. Nothing at all. Is he going to get cured or not? Does he need treatment or not? We do not know anything. They did not explain anything to us. They do tests and then get the results a month later. On top of that, they repeat all the tests as if the ones that were done before were not adequate.

Riham: So the things you expected to happen did not happen.

Participant: Not at all. What I imagined in my mind and the reason we came here and left all our family, I did not find. I had hopes of seeing my son getting proper care and a cure. I did not see that at all.

Riham: How did you imagine it to be?

Participant: I thought there would be more medical care. His father has been also experiencing mental stress and his blood pressure is high now due to his son's illness. Now his father's health is also deteriorating. We both imagined it to be different.

Ahmed: Regarding these difficult circumstances, if you were in Jordan or Syria, who would you have turned to for help with your difficulties? Are you finding people here to help you overcome these difficult circumstances?

Participant: No, we are not finding. We are locked up in between four walls. Who would help us?

(26:23)

Riham: It appears like there are five.

Ahmed: If you were in Jordan or in Syria, who would you have turned to for help? I am trying to understand what things are missing here.

Participant: The doctors there understood us and helped us. It is not like here. The doctors there follow up on their patients every day.

Riham: How do they follow up? Do they call you by phone?

Participant: Yeah, a lot of the doctors call by phone. Also, if I would go to them, they would say to come back the next day so they can follow up on them. They ask about the child and check in to see how they are doing. They do that every day.

Riham: Do they come to the home?

Participant: Yes. If a patient is not able to go there that is when they come to the house. In Syria more than in Jordan.

Riham: I am going to ask Ahmad’s question again. What are some things you need here that can help you feel more settle and also help you prosper here? What are the intangible things that are missing? Things that will help you prosper psychologically. Do you understand the question?

Participant: Yes, I understood. I don’t know how to answer you though.

Riham: It’s okay, think about it. We will wait patiently.

Participant: There are a lot of things that are missing. Emotional support is one. The hopelessness my son is feeling. He and his father are the most ones that are ill here. If the doctors could help us and follow up on their condition by giving them the results and the cure, that would help.

Ahmed: When a person is in their own country, they often have a community with shared values to help them.

Participant: For sure. They help when someone is ill. If they were to need a hand, all the relatives would be ready to assist. Here, there is no one.

Ahmed: You are not finding the Syrian community helpful with these matters here?

Participant: No, we did not see that. May God reward the Syrian community. They helped so much when we first arrived. But. They are not able to follow-up with us like a family member would.

Riham: Right, it's not like family.

Participant: It is not like an uncle or cousin would. Life here is difficult. Everyone has their own life. It’s not like how family drops everything for you. If a family has an ill child, everyone steps in to help.

Riham: Of course.

Participant: It’s a different environment.

Riham: Even the neighbors would help, right?

Participant: Of course. [Patient cries]The neighbor would check in with us in the morning and evening. They would knock and check up on how he is doing. They would ask if they ate or slept. Even though they aren't family, but they still ask. The family would come in and lend a hand. His cousins would help him. They lived with us in the house and helped him with everything. They supported him and helped with caring for him. They were always with him. They slept with him in the same bed and did not leave his side. Not even for a second. If one leaves, the other is there next to him asking him what he needs. They supported him emotionally.

Riham: Here, he does not have that thing.

(31:35)

Participant: He does not. Honestly, for three months they lived with him in the same room. They did not leave him for a second. If he needed to go to the hospital, they would carry him down. We were on the 3rd floor. They would carry him on their backs and would bring him down. This is when he started to crawl. Before that, the doctors used to come to the home to treat him. To do physical therapy with him. He did not have to go to the hospital at all. The doctors used to bring him the machines to the house and treat him there. His cousins used to carry him like this and the doctors would help him. On the days he needed to go to the hospital, they would bring him down the stairs and go to the hospital with him. Thank God. They would protect him and feed him with their hands. They would remind him to pray and supplicate to God. They would say, “Ahmed, hold on. God will cure you. Ahmed, you will get better.” That was all he needed. It was good for his psychological well being.

Riham: It was also good for your psychological wellbeing as well.

Participant: Of course. I saw that my son had someone to care for him if I were to go up to the kitchen to cook for his siblings. He had someone to care for him better than his own mother could care for him. They would take care of him better than I would take care of him.

Riham: Your heart was put at ease.

Participant: A lot. A lot.

Riham: You do not have these feelings here?

Participant: Not at all. The day that both he and his father were hospitalized, I fell to the ground crying. His father sent me a message saying he is going to the hospital. Mona from the organization came over to tell me that his father fell down in the charity organization and they carried him to the hospital. He had a stroke. This happened about a week ago. He [her son] had an incident here on the floor due to a neurological condition. I didn't know whether to go with my son or to go with his dad. I fell to the ground crying. I went with him to the hospital and left the kids here. I sent Ayham to go see his father at the hospital and the others stayed here.

Riham: They stayed with Mayadah.

Participant: [crying] They stayed with my daughter Mayadah and her older brother. There is nothing like having relatives. I spent 26 days at the hospital and could not see my kids [while they were in Jordan]. My sisters and my husband's sister stayed with them at home. Their grandmother was there too. They were all there to take care of them. I was with him along with his dad and his uncles at the hospital. Here we don't have this at all. Here it wasn't until the second day that I called the organization and someone came to take me to see Abu Ahmad. I stayed with Ahmad at the hospital until the evening. Thank God they discharged him then. He had a neurological condition and it passed. They didn't even give him a shot. We went in and came out and they did not even give him anything. They just made him an appointment. Even though he was having spasms and his feet were paralyzed. He could not walk. They had to take him in an ambulance. They did not give a cure. All that and they did not give him anything.

Riham: They didn’t draw any blood or anything?

Participant: No, they did not even touch him. They just made him an appointment for the 23rd of the month with the neurologist. He went back to his normal state after five hours. I was with him the whole time at the hospital and then we came home. After all this, this trial we experienced, I came back home with him and I couldn’t even see his father at the hospital. Even though his father was in a bad state. There was no one to take me to see his father. I just waited with the kids and I could not sleep that whole night because I couldn’t stop thinking about their father. I didn’t know how he was doing or what state he was in. This happens each time. This was the third time he has this shocking experience [literally means heart attack] happens. We spent time in the hospital with about 20 different people. We would sleep there, spend hours waiting, and follow up with the doctors about their condition. He stayed alone in the hospital and slept there alone. It wasn’t until the next day at noon that someone came to take me to go see him.

(36:10)

Riham: Who came to take you?

Participant: The organization [Catholic Charities]. I called them and they could not understand me. I was speaking in Arabic and the man speaks English. He understood that I wanted to go to the hospital because I said the word hospital. He came and took me there. I was able to go see Abu Ahmad. It had been more than 24 hours at that point till I was able to visit Abu Ahmad in the hospital as he was sick. This was a really difficult time. Isn’t this suffering? Is there a greater suffering? …[cries] Thank God. Life is really really difficult here. We are really suffering. This is the first time in my life I felt this level of suffering. From the doctors and the life here and from everything. From loneliness as well. Everything. Feelings of hopelessness and emotional suffering. It has all been a struggle. Thank God the most High [saying in Arabic].

Riham: Thank God. You really need to take care of your mental and emotional well-being.

Participant: How am I going to take care of it? I am dealing with high blood pressure. It’s been a week that these two parts are having spasms and hurting me. My neck is hurting me as well. Even the disc is unbearable. Thank God.

Ahmed: Is there anything on your mind that we did not cover?

Participant: Even if I spent two years speaking, I will not finish.

Riham: You only get an hour. I told you that. Your husband used up all the extra time! [laughs]

Participant: [laughs] Ok! I have an allergy as well. I spend the whole night sneezing.

Riham: Yeah, Ohio has a lot of allergens.

Participant: I am sick and have not benefited from here. My daughter has been suffering from stomach pain and I told her when we get to America, you will feel better.

Riham: Your daughter?

Participant: Yes. I told her she will get better when she comes here and she got her hopes up. We expected her to heal here.

Riham: Did she see a gastroenterologist?

Participant: She did not see anyone. When we told them that her stomach was hurting her, they would give a bag with warm water in it and tell her to put it on her stomach. They did not run any tests or examine her.

Riham: She had this before --.

Participant: It happened in Jordan. After her brother became ill. She used to constantly be sad about what happened to him and she would cry a lot. This was when he was in the hospital.

Riham: Did they tell you that it might be psychological?

Participant: They did not tell us anything at all. They did not even examine her.

Riham: Sometimes a person has --.

Participant: This one was sick and they didn’t care for him the way they should. How could they care for any other person? They just give us an appointment and some words. Appointments. Appointments.

Riham: Do you think that if you did not miss these appointments that it would be a different case?

Participant: It is the same thing. We did not get any clear results. Things are not clear at all. We are suffering because of the language, the doctors, being far from family, being far from the hospitals, and all this stuff. They would tell us to be patient. This one suffered from his molar teeth and that other one from her stomach. If one of the children gets a fever I might be able to treat it at home. However, those suffering from the pain inside, I cannot know what is wrong with them nor treat them here. I have to make an appointment and then they can go. A person would get better or might just forget about their illness by the time the appointment comes!

(40:46)

Riham: Maybe that is the point. That way they can know which illnesses are not going away on their own.

Participant: It keeps coming back though. It is not like it happens once and then goes away. My daughter’s stomach pain is constantly coming back. A tooth also doesn’t just heal on its own unless it has a cavity. If my sons molar tooth was infected, they should give him antibiotics. Instead of having it slowly going away on its own. They should give them medicine to ease the pain. A person cannot always bear the pain. They feel hopelessness when they have pain and cannot get medicine to treat it.

Riham: Okay so tell me about your daughter. You told me she has stomach pain. Did you take her to see a gastroenterologist?

Participant: No, we don’t know which doctor. We just take her to the appointments they made for us.

Riham: Is that a primary doctor?

Participant: I don’t know what type of doctor they took her to.

Riham: You weren’t with her?

Participant: No, her father was with her. A primary doctor, she says.

Riham: A primary doctor. Weren't you there? I wanted to know if they tried to refer her to a gastroenterologist and maybe you missed the appointment.

Participant: I don’t know. They did not give her anything. She told them my stomach always hurts me and she has indigestion. She told her to just put the warm bag of water on it. The pain has been there for a long time. Ahmad is really having a hard time with his urine incontinence. It is making him really frustrated. He is depressed. He is 18 years old but has to keep a diaper on day and night. Isn’t that wrong?

Riham: It is really hard.

Participant: It is really destroying him. He is embarrassed around his siblings at home. He is embarrassed by me and embarrassed when we go anywhere. He will get embarrassed in school that he has to take a diaper with him. Isn't this hard on one's psychological state? He is an 18-year-old young man at the beginning of his life. May God protect him and prolong his life.

Riham: Amen.

Participant: I came with high hopes that they will care for him and give him the treatment that he needs…a. and that they would take better care of him. I did not see any of that.

Riham: Did you think that he would heal right away?

Participant: I thought he would be given something at least. Healing will come from God but there is a cure for everything. I had hopes that they would give him medicine and give him proper care. I hoped they would do more imaging and evaluation. We did not feel like they did at all. It has to be better than this. Thank God for everything.

Riham: I am really sorry that this is your impression and your experience here. As doctors and as Americans, and as fellow brothers and sisters in Islam, we want you to feel safe and secure here and to be treated in a humane way.

Participant: We did not feel safe and secure here at all. When we first arrived we had shots fired on our home. We had to keep the windows and doors locked constantly. We did not dare to go out. Even when we needed to go out to get food, the kids would be too afraid to leave the house. They brought us to a terrible neighborhood and we had snakes at our door for days. If anyone would try to open the door she would lift her head up like this and start to blow at the door.

Riham: A what?

Participant: A snake.

Riham: Oh no!

(45:23)

Participant: Yeah, here is her picture. They kept telling us that it will go away soon but it didn't. It lived under our door and every time we would come it would stick its head out like this. We started to exit from the back door in the kitchen. Thank God that we found this house and we moved in immediately. Even when we were moving our stuff out we had to use the back door out of fear of the snake. We were afraid that it would get into our things or hurt one of the kids as we were moving the stuff. There were little bears. When my kids would open the doors, they would come to them. It was a small animal that looked like a bear.

Riham: A raccoon maybe?

Participant: I don't know. There were ants like this in front of the house as well. Ants everywhere. How could we feel secure after having lived like this for three months? My kids would not dare open the door out of fear of these wild creatures.

Riham: Oh my God. I just saw a picture of the snake.

Participant: This one was laying down.

Riham: Please take it away or else I will not sleep at night.

Ahmed: May God give you strength.

Riham: Is there anything we can do, in your opinion, so that the Syrian families that come after you can feel secure, hopeful, and cared for?

Participant: By not stuffing them in homes like the one they placed us in. Not in a neighborhood that is run down, dirty, and filled with drug dealers and animals. That is wrong. They would be happy and hopeful thinking they are coming to a new life here and then they get this treatment. The organization would throw them in houses that are dirty and in dirty neighborhoods. There should be an improvement in the interpretation services. They should have people assigned to take care of them. They should have people assigned to each family that understands the situations they came from. There was a lot of suffering at the beginning. Of course, we know that the beginning is always tough but it doesn't have to be to this extent.

Riham: This isn’t the only beginning in your life.

Participant: This was the hardest stage I have ever experienced in my whole life. I am forty years old and I have never experienced anything as difficult as those first three months of coming here. We first went to Baadia [the Syrian steppe or the Jordanian steppe] with rockets and missiles…and when we would see a plane, we would get scared and hide in our homes or shelters. As soon as it would leave, we would all come out and resume our normal lives. We had our relatives there and we would eat together and get together but it was nothing like here. Here we had a bad neighborhood and people we do not know. It's a tragedy when a person first arrives here. On top of that, a person has left his family and his world. There are those that are coming for the future of their kids and others for a sick child that needs treatment. Imagine they come under these circumstances and they find other than they imagined. They picture aspirations and goals in their minds and come here to find something completely different. It's wrong to bring these families here and put them in houses like these. They need to care for them more than this. Sometimes they give us an appointment and we go and spend 6 or 7 hours stranded at the hospital.

Riham: That is wrong.

[49:47]

Participant: Sometimes they would say the volunteer did not come to pick us up and other times they would say that it wasn’t time for the appointment yet. Sometimes they say the interpreter is busy elsewhere. A person would waste a lot of time in the beginning “all for nothing.” He would not get anything out of it. Wherever he goes he does not get any benefit from it. A whole day gone. One needs to rest physically and mentally. We can’t waste a whole day waiting at the hospital for an interpreter or a doctor’s appointment. They reschedule these appointments constantly. Often we would go to these appointments with high hopes thinking that we will get something out of that visit. We are going with stomach pains and headaches hoping to leave feeling better. We and end up talking and the interpreter tells the doctor what we are saying. Most of the time he does not understand what we are saying and doesn't respond and sometimes they would just reschedule the appointment. It has been like that since we came. From the beginning. Allah is sufficient for us, and He is the best.

Riham: It is a different environment and system.

Participant: That is right. It’s a completely different system.

Riham: It takes time for a person to get used to a new system.

Participant: How can one get used to it. It has to be better than this. If this is their system, we will not be able to change it.

Riham: We can change some things. That is why I want you to tell me your thoughts and opinions. I want us to work together to figure out what to change. You mentioned housing. What else can we improve?

Participant: The medical treatment. The most important thing is a proper medical treatment for the refugees. It has to be better than this. It is true that they have a lot of refugees but in the beginning, they need time to adjust and get used to things. We still do not know anything. We are just going to these doctor appointments and have not gotten anything out of it. They need to care more about this matter, more than they are doing now. They should not put someone in the charity organization that is going to throw the refugees in terrible neighborhoods. A person that will not care for them and cause them to miss appointments. An organization that will not give the refugees their rights. They welcome us and place us in homes and tell us to pay $1000. Later on, people would tell us that it is not even worth $400 dollars. They would wrongfully take $1000 from us. A refugee leaves his home and his family behind to come here in hopes of making a better future for their kids. Abu Ahmad is ill and he is not able to support his children with these things. They cause us to miss out on these opportunities. They take all these opportunities away. They should try help refugees more than this.

Riham: You have a long life ahead of you.

Participant: God is the one that sends down the illness and He is the one that can cure it. A doctor is a means for the cure, though. The doctors and others as well. God made them a means to help the ill and those in need. Doctors have a responsibility. They are able to find the cause of the illness through exams and tests. Medicine is advanced now and the doctors know how to find the cure. They should try to help him more with his illness. His emotional state is destroyed. It is wrong. He is still a young man.

Riham: May God cure him.

Participant: Amen. In Jordan, a doctor told him that he will find himself fully cured before six months go by. Of course, the cure comes from God but this doctor clearly said this. We have fallen into a state of hopelessness because we came here against our will almost. They convinced us of things that we did not even find here. He had some doctors there that were following up on his case and ready to treat him.

Participant's Husband: We tried to go back.

Participant: We tried to go back so that our son could complete his treatment that he already started there with the specialists. They tell us we cannot by law. They call this the country of freedom. This is not the country of freedom! When we find ourselves in this situation, how can we feel free? They bring a person and force things on them. That is wrong. [cries] They have ruined our psychological wellbeing. They told us that they have brought us here for the sake of our children’s futures and to treat our son of his illness but it was all talk. It was all a lie. If they would take me back there now, I would go. I don’t want any of this because it is all wrong. It is wrong. Honestly, if they were to tell me that there is a one in a million chance that they would take us back I would go to it crawling just to go back. This is completely wrong what they are doing to us. [cries]

Riham: There is no power but from God [saying in Arabic].

(55:57)

Participant: They say this is all for the future of our children. This is not our society and they are not our people. This is not at all what we imagined. We did not even see a fraction of what we imagined. Even this child that is sick is saying he wants to go back to Jordan. Two days ago, he picked up his passport and started walking out. He wanted to go to the airport and go back home. Jordan will ban you from entering. They set it up in a way where they will not take the blame for us leaving. They do not want to take responsibility. They destroyed this young man's future. He still has a long life ahead of him. They have protected themselves so that we do not complain about them. They were the reasons we migrated. God is sufficient for me and what a great Being to rely on [saying in Arabic]. They destroyed the future of a whole family. There are nine people in this family. All this happened because of a shot taken for medical reasons. Now we have to carry him around. God is the one that cured him. They gave us hope that he will continue receiving treatment here but they lied to us. This is all a lie. The whole purpose of the migration is for this reason. They assured us that the doctors there would be communicating with the doctors here, but it was all just talk. The migration was because of the doctors. God is sufficient for us over them [saying in Arabic]. They promised us with things and did not keep their word. Everything that was told to me and my husband, I did not see. Look what happened to us. They want to ruin our children's future. They were telling us that our children do not have a future in an Arab country. Supposedly they do not have an education. Yes, they do! They were studying in Jordan and doing well in school. It is not like how it is here. Our children's future depends on this thing. We thought they would have a better future than this.

Riham: Is it too early to tell? It has only been three months and one day.

Participant: No it is not too early. We cannot handle living a life like this. It is hard for them.

Riham: It is very difficult.

Participant: It was clear from the beginning. We knew from the first impression we got. They are going to schools filled with corruption. We will be responsible if anything were to happen to them. We won’t get into this topic since it is not related to the medical field. We are not acclimating to any of this. We are trying to acclimate to it and pick ourselves up [improve our mood] for the sake of our children but we are unable to. Not a day goes by that we do not cry. Abu Ahmad, my older son, and I sit and cry at least four or five times a day over all this. Abu Ahmad gets high blood pressure and needs to take medicine to bring it down. My son that is sick, however, has a short temper now. This is new on top of the physical ailment. He suddenly gets angry. His life might get ruined even more than it is now.

Riham: When some people find themselves in a difficult place, they try to do things to cope with it. For example, they might write down three positive things that happened that day. They might do this every night before they go to sleep. Of course, we pray every night and day to God. Did anyone talk to you how to do things like this? These things don't come naturally to us, we have to learn how to use them during difficult times in our lives. Do you understand what I mean? For example, I learned about this writing exercise that I mentioned during my adult years.

(60:44)

Participant: When we go through a hard time we just get up and pray to our Lord. We don't know how to write or read or anything. Our knowledge is limited to that. If something bothers us, we stand up, make ablution [ritual way for purifying oneself before prayer], and pray to God the Most High [Arabic saying]. We might read the Quran as well.

Riham: Nothing compares to turning to God, of course.

Participant: Reading the Quran is healing as well. It will put one at ease. That is what we do to feel better.

Riham: If you were able to reach out and get help in dealing with your psychological well-being, how would you feel about that? It is a form of science and art. What if someone could help you find ways that help you? When I would find myself under a lot of stress, I go running, for example. Everyone has something that they can do to help them cope.

Participant: I have responsibilities. If someone is part of a huge family, they cannot do that. When I am upset, I just turn to prayer and crying.

Riham: You could use this time that you would spend crying on something that could put your heart at ease.

Participant: What kind of thing?

Riham: My field is not psychology, it is emergency medicine. I don’t know if I am expressing myself clearly.

Ahmed: Would you be open to seeking help from mental health professionals that could help you deal with your difficult circumstances?

Riham: As in coping mechanisms.

Participant: If our life was easier we would not need a psychologist. We just want them to make life easier on us.

Riham: Life is difficult though. Isn't that right? Life is on and we cannot change that overnight. What coping mechanisms can we use to deal with it? There are things that are out of our control, right? There are some things that are written [fate]. There are exercises that a person can learn so that they and their families can practice them.

[indistinct]

.

Riham: You said that yesterday, you were feeling uplifted. Can you tell me what happened yesterday to make you feel that way?

Participant: We took the kids to the park and we took tea with us. Our kids had a game at school and they went to watch it. We took the young kids to the park. It is true that we saw people different than what we were used to but it made us happy to see our kids happy. The young kids were happily playing on the playground. This helped us feel better.

Riham: May God fill your hearts with joy and may He make all your matters easy on you. God is Great. Do not forget that God wrote everything.

Participant: There is no God but Allah.

Riham: Everything that you lost and everything that you gained. Praise be to God.

Participant: That is true but a person thinks and does his part.

Riham: Is there anything else before we end?

Participant: No.

Riham: Okay, thank you. It’s been our pleasure.

Interview 6A

Ahmad: Today is July 12, 2016, and Riham and I are with participant 6A.

Riham: My name is Riham Alwan and I am with Ahmad Beydoun here. We are with a father of seven children. We will be starting our conversation now, God willing.

Ahmad: Alright, we’re going to start with a general topic. How has your experience been with the practice of medicine here in Cincinnati? You’ve been here for a year and a half now, right?

Participant: Yes. Thank God, it’s good.

Ahmad: Do you, your wife, or your kids have any health issues?

Participant: My wife. She has some issues with her eyes and pre-diabetes.

Ahmad: Pre-diabetes, okay. How often are you in need of visiting the doctors or hospitals here in Cincinnati?

Participant: Often.

Ahmad: Often? Is it due to anything specific?

Participant: It’s because of my children and my family and so on.

Ahmad: Okay. You mean for the vaccines and whatnot?

Participant: Yes.

Ahmad: Okay. Have you been to the clinic before to get the vaccines?

Participant: I went maybe twice or three times.

Ahmad: How was your experience in clinic?

Participant: It was very good. Yes.

A; How were you communicating with the doctors?

Participant: By using an interpreter.

Ahmad: Who would provide this interpreter?

Participant: He would be there at the hospital.

Riham: While speaking to some other refugees, a common complaint we would hear about the interpreters is that their dialect might be hard to understand at times. Do you feel that way as well?

Participant: The interpreter would be difficult to understand if it’s over the phone! Over the phone… When an interpreter speaks on the phone – excuse my language- but it is despicable.

Riham: Not only is his dialect difficult to understand, but also his whole speech?

Participant: The whole thing is despicable. The language. The attitude.

Riham: Explain what you mean.

Participant: They would say, "What are you not understanding me!?". When I speak to the doctor in person…even though I don’t speak English…The doctor, she would at least understand what I am saying because of my body language, but over the phone, it's hard for the interpreter to truly understand. He would sometimes shout at me and say, "what you don't get it?!"

Riham: That’s how he would talk to you?

Participant: Yes. Just like that. Every single interpreter on the phone is extremely terrible.

Riham: Did you ever complain to the doctor? I guess you can’t complain because the interpreter will not translate your words.

Participant: All of them are terrible. I advise all of them to not interpret over the phone. Yes.

Riham: You don’t feel at ease with them?

Participant: It’s not a matter of not feeling at ease. It is lowly and despicable. The experience is terrible.

Participant's Wife: When we ask them to repeat their words, they don’t repeat them.

Riham: So if you tell them, “excuse me, I did not understand. Can you please repeat it once more?”, what do they say?

Participant: If I say I am sorry can you please repeat the answer, he would shout, “what’s going on my brother, you weren’t listening to me? You can’t hear!”

Riham: Oh my!

Participant: I can’t even explain to the foreign doctor in front me that this interpreter is speaking to me that way. If he was sitting in front of me --.

Riham: They would understand your body language and from the gestures and sounds.

Participant: Every interpreter over the phone is terrible. Just terrible.

Riham: Okay. What about the interpreters that show up in person?

Participant: Good. Very good.

Riham: Always?

Participant: Always.

Riham: Okay, that's good.

Participant: I have interacted with several interpreters, both male, and female, in person, and they were great. I had personal experiences, and they were really good.

Riham: Okay. That's really good. So regardless of which country they're from, you aren't facing any difficulties?

Participant: Yes, regardless of which country they are from. They are all really good. The ones on the phone, however, are really terrible.

Riham: Where did this incident take place? The one in which he spoke to you that way over the phone. I am asking because I know the name of the company that we use at the hospital. It's called Pacific Interpreters. I use it every day with people that speak a language that I don't understand.

Participant: It happened with me --.

Riham: Was it in the hospital or at different places?

Participant: It happened to me twice in a row at the refugee resettlement agency. Three times it --.

Participant's Wife: One time at home.

Participant: When was it at home?

Participant's Wife: When that man came --.

Participant: Yes, it happened once at home. Another three times in refugee resettlement agency. Three times in Dayton, I think. The place where we did the interviews for the food stamps.

Riham: Oh with the government agency.

Participant: Yes, with the government agency. Even though the American man was really polite and kind, the interpreter wasn't.

Riham: It's only the interpreter.

Participant: Yes, only the interpreter. The interpreters over the phone, whether it be male or female, were just terrible.

Riham: That's unfortunate.

Participant: Yes, it's really unfortunate. The phone interpreters are just terrible.

Ahmad: Just to clarify, did you face any of these problems with the phone interpreters at the hospital?

Participant's Wife: No, in the hospital if they were provided in person, they were really good. If they're over the phone, they were not.

Ahmad: So even at the hospital.

Participant's Wife: Yes, even at the hospital. They would say something like this, "Listen, the doctor is about to say some things. I am not going to repeat it. If you hear it then good, if not then too bad".

Ahmad: I see. So even at Children's Hospital?

Participant: yes.

Riham: Yeah. It seems like it's the same company they use. They most likely all use the same company because it's a large company.

Participant: All the interpreters.

Riham: Yeah it's ideal in person. It's easier on the doctors as well as on the patient.

Participant: All the interpreters are --. For example, you would hear the doctor speaking and speaking and speaking. The interpreter would then sum it all up in a word or two. It doesn’t make sense.

Riham: Yeah, they do that often. We get really bothered when interpreters do that.

Participant: It happened to me and to a friend of mine. They were here conducting an interview with me. For a whole year.

Riham: A whole year?

Participant: Yes, they were interviewing me for a whole year. They were journalists.

Riham: Oh, journalists. Okay.

Participant: They had some questions for me. They brought with them some interpreters. I said so many things and later found out they didn't relay it to the journalists. I asked them why they didn't translate all of my speech. [indistinct]

Riham: So your words were lost in translation.

Participant: Yes, they were lost. My words were lost.

Riham: Yeah, the language barrier can really make a person feel estranged in a new land. It makes one feel like they don't have any --.

Ahmad: Any power.

Riham: Yes, any power.

Participant: When it comes to the language --. If one does not know the language, it's as if they are deaf and mute.

Riham: Yeah, exactly.

Participant: It makes one feel deaf and mute.

Riham: Yeah, it's really hard on one emotionally.

Participant: A person would feel unaware of everything around them. Don't get me wrong. Let's say there's a watch for $90. If I knew the language I could get it for $30. I would change my whole lifestyle. In terms of comprehension, I don't comprehend the language.

Riham: Are you trying to learn?

Participant: I am trying so so so hard. There is absolutely no comprehension.

Riham: Alright so tell me how they are teaching you. I heard many stories about what the teachers there were like and that they weren't really good.

Participant: No, no my sister. It depends. When it comes to an old man, it's difficult to learn a new language. If you tell them you aren't picking up the language they ask you why not. How can you not pick up the language, they ask. My family doesn't know the language. My wife is at the fifth-grade level and I am at the sixth grade. Her language is good. She can read, she can write, and she can converse. She can manage by speaking. When we go to the lesson at the mosque, every Sunday, she's really really good with the language. She can memorize. I can't comprehend the language, but she can.

Riham: Can I suggest something to you? It could be because of how your wife, as she told me, befriended a lot of women when she first arrived. When a person learns a language, there is a certain amount that one can pick up in the classroom, but then there is a huge part of learning that comes from having everyday conversations with others.

Participant: I am not able to speak. She is able to comprehend. I went to more lessons than she did at the organization. The English lesson the organization offered.

Riham: Yeah, I mean in regards to your work. You have the opportunity --.

Participant: I understand what you mean. She went with me for two months to the organization. She comprehended a lot but I did not. She would play something on a CD, on television, or on her phone. If she hears a word she would memorize it and pick it up quickly. I, however, will memorize it one day, then completely forget it. I go to work and come home and listen to the CD. I don't comprehend it. At work, I understand them. I understand them really well. I understand 100% but I can not speak.

Riham: Oh, okay. It might just need time. At least you understand what they are saying.

Participant: Yeah, I understand them. If someone speaks to me, I understand what they want from me. I just can't speak.

Riham: It just needs time, that's all. Everyone has their strengths. For example, look at how I speak Arabic. It's scary. My brother, on the other hand, if you hear how he speaks, it's amazing. He's a teacher and teaches Arabic. Everyone has their own mental strength. Some people are strong in language learning. They can learn four or five languages quickly while others need more time. Be patient and go easy on yourself.

Participant: Thank God

Riham: Yeah, thank God. It's good that things are going well at work. Tell me how your work situation is now.

Participant: Work is going really well. If a person does not work here, he has no life.

Riham: Not just here, right? It's like that back in your country as well, right?

Participant: No matter which country one is in. If a person does not work, he has no life.

Riham: Yeah, of course.

Participant: If you don't work who is going to provide for you? No one is going to pay for you to live. We are forced to work. There are children. There are expenses and bills to pay. What is one to do?

Riham: Yeah, that's true. Like many men that first got here, you spent some time without work, isn't that right? This goes for both men and women of course, but it affected the men more. Can you tell me a little about how your mental state was and how you were able to cope with that situation?

Participant: I spent you could say--. When I first got here it was like the honeymoon phase.

Riham: Of course, always.

Participant: The first month was like the honeymoon phase. It was like paradise. However, heaven without people isn't to be entered, as they say. It's nice but it's not nice at the same time. One doesn't have a neighbor that checks on them. You'd stand in the street and wish someone would just walk by and say, "Assalamu alykum." All you would wish for is that another Arab would knock on your door just to say hello. There isn't. If you need to go get some things, groceries, and don't have a car, there is no one to call? It becomes a psychological problem. I started to go out for a walk and cry. I would sit and cry. I broke. I could not take it anymore. They told me I needed to go to the organization. They said I would have to go by bus. I told them I would go by bus then. It was a long way. I would leave my home at 9 am and arrive at the organization at 12 pm.

Riham: Oh my! Three hours?

Participant: By bus.

Riham: Three hours on the bus

Participant: If you're just five minutes late, you'll miss the bus and have to wait an hour. And I had to change buses. I told them, in this case, I don't want to take the bus.

Riham: Yeah, it's hard.

Participant: I don't want to attend the lesson in this case. I am not benefiting from the lesson!

Riham: You would lose two hours.

Participant: I would spend three hours on the bus! Not to mention the type of people I would see on the bus and the type of illnesses. I told them I am not going anymore. I stopped going. Then they sent me a message saying you can come by bus but using a different way. A shorter way. I told them I didn't mind if it was a shorter trip. The short route they sent me on would take only 45 minutes. That's really good. I started going frequently by bus. Then I told them to find me a job. I didn't care what kind of job, I just wanted to work. They then found me a job at Chick-fil-A. I worked there for about a month or so. I used to go walking there. Half an hour each way. It wasn't bad. At Chick-fil-A, the owner was an American. She told me she could find me a job at a church.

Riham: At the time you were still working at Chick-fil-A.

Participant: Yes, I was still at Chick-fil-A. She said she would find me a job at a church. I said sure. We went and had an interview at the church. They said they would pay me $15 an hour. The job would include cleaning, mowing the lawn, painting, moping, and sweeping. Basically, everything. We thought it was good. I filled out the paperwork. They then told me in a few days they would get back to me. So I told my Arab friends. I told them that I found a job at the Church and on and on. One of them told me to “stop right there and do not dare it”. He said he would find me a job. I told them okay. He came over and said that he has a friend, a friend from childhood, from school, that can give me a job. A job that would pay $16 an hour. I asked if they knew the guy. They had been here for 20 years or so. At that point, I had only been here for four months. We went to the Indian restaurant and met with the guy. They both came to an agreement. He said $16 an hour. He said, however, there is one rule. There is no break. Not even on the weekends.

Riham: That means you would work every day.

Participant: Every single day. From 10 am to 10 pm. He also said he has five other workers and needs me to bring them with me. They were other workers that worked for him.

Ahmad: So you would bring them to work and take them home.

Riham: Oh, you would be their transportation?

Participant: Yes, in this situation… sorry to go on a tangent. In this situation, the Arab community had secured a vehicle for me. So I would have to drive the other workers.

Riham: So your job was to pick them up.

Participant's Wife: He started then to leave at 6 am in order to pick them all up.

Participant: I would take them all to work and bring them back home.

Riham: Would they pay you for the gas?

Participant: No, no, no.

Participant's Wife: It was supposedly included in the $16 an hour.

Participant: I told him to give me my paycheck. I need to pay my rent. He said okay. He gave me $1200 cash and he gave me a check for --.

Participant's Wife: That was after working for a while.

Participant: Yeah, I had been working there for a month when I asked him. He gave me a check for about $300. Basically around $1,600 total….[pause]… I told him to give me my full paycheck. He would say, "what?! What is this?" I told him okay okay. He clearly didn't understand me. I called my friend to translate for me on the phone. I told him I needed my money. I need to pay my rent, the car payments, and the electricity bills. He talked to him. I asked him what he said. He said he would call him back shortly. It was a Monday. Anyway, he called me back and said, “I am really sorry Abu Hasan. I am indebted to you", he said. [pause] I was afraid at this point. I was scared. I saw from the way he looked and acted, that he wasn't going to pay me. I asked him what in the world happened. He said there was a misunderstanding. "We misunderstood him", he said. I said how is that possible? He said that the offer was $1,600 a month. I asked how can that be.

Participant's Wife: Turns out that for all his work he would only get $1,600.

Riham: How did it start off as $16 an hour and end up at $1,600 a month?

Participant: Yeah, can you believe it?

Participant's Wife: So for 40 days of work he only got $1,600.

Participant: I don't understand English but you two understand English. My friend understands English just like you two. How did he possibly misunderstand him?

Riham: Yeah, how do the numbers just change like that?

Participant: Of course, I did not have any contract or paperwork to prove that! I said to forget it. I will leave it to God. God will replace my loss for me. I am not going to work though. I stopped working. I stayed at home without work for maybe two or three months.

Participant's Wife: That comes out to less than $4 an hour

Riham: That comes out to about $3 an hour.

Participant: Yeah something like that. Something really pitiful. It was hard work. So I sat at home for 3 months or so. I found a job at a restaurant that was only once a week.

Riham: You couldn't go back to Chick-fil-A? Remember how you told me you started out there?

Participant: At Chick-fil-A --. I did start out there but…I would work and…it was clear. There are no customers, I am not needed.

Riham: Oh I see. So there wasn't a lot of work to do.

Participant: Yeah there wouldn't be work to do and I would just stand around waiting. They would send me home. Thank God. I know found a job at a music store. I have been working there for about a year and a month now.

Riham: Thank God. Are you feeling more at ease now?

Participant: Yes, thank God.

Riham: So for the difficult time you experienced in between jobs, can you tell me what some of the things are that you did to cope? How did you help your wife and children cope?

Participant: The most important thing is for a person to be honest with others before being honest with themselves. That's the most important thing. If you're honest with others you will be honest with yourself, with your family. I want to strive. Find me a job I would say. There will be days that I get tired and don't want to work, but I have to work. Or I would be told that I could find a better job closer to me that would pay more. I have to be consistent, stay at the same job, not try to be pulled left and right for different opportunities. The most important thing is for a person to be honest with others.

Riham: Yeah.

Participant: Now at my current job, my co-workers are all Americans. I am loved by them all, thank God.

Riham: That's great, thank God. God makes things easy.

Participant: Thank God.

Ahmad: How did you feel after you experienced what you did when working at the Indian place? I am sure it must have been really difficult.

Participant: It taught me about myself. Please excuse me for saying this but I said every curse word that exists. I cursed every person and everything.

Riham: Did the kids get cursed at too?

Participant: Everyone got cursed at. Imagine how it would feel to have all the hard work I put in to go to waste. I would be slicing onions and meat and other things for hours. This hand right here was injured for about 15 days. I cut myself with the knife.

Riham: Did you go to see a doctor when that happened?

Participant: No, why would I go? I bandaged it. Imagine someone not compensating you for all your hard work. On top of death, they are re-burying you [a saying implying life was already rough and it worsened]. …Thank God. It passed.

Ahmad: What was the most thing that helped you through these challenges?

Participant: Patience. A person needs to be patient. There is nothing else that can be done. Pray to God. Ask Him for forgiveness.

Riham: You said you used to go for walks.

Participant: Yes, I used to go for walks.

Riham: Did you feel like you used to walk more and exercises to cope?

Participant: No not really, I didn’t exercise.

Riham: You didn't feel like it?

Participant: I can't really exercise because of my foot. It has a --.[indistinct]

Riham: What is it? Like a rod?

Ahmad: Yeah.

Riham: So that means you broke it before?

Participant: No, no. I had an injury. Shards. Metallic. From an explosion.

Riham: Oh alright, so that's why you can't exercise.

Participant: Yeah if I exercise it gets worse.

Riham: Do you feel pain while working?

Participant: Currently no, I don't feel pain, thank God. It's fine. When I go out to walk, I go out to calm down and relax.

Riham: You kept up the walks?

Participant: Yeah, I kept it up. The period in which I was working, I would go out and walk.

Riham: So till now --.

Participant: No.

Riham: Why?

Participant: No my job requires a lot of walking as it is.

Riham: Oh, so you feel tired.

Participant: Yes, my job requires a lot of walking.

Riham: So we spoke earlier about the importance of exercise for health. Especially for your kids. Your wife told me that there wasn't a gym or *** for them to go to but do you encourage them to exercise or anything like that?

Participant: I encouraged them a lot. At school, I even encouraged them to join the soccer team.

Riham: Oh, that's really good.

Participant: Yeah. I really encourage them.

Riham: So they would play soccer.

Participant: Yeah. Every time we go to the mosque they would play there as well.

Riham: That's really good. Is there anywhere nearby you could take them?

Participant: Their mother takes them.

Riham: Okay, that's really good. May God protect her.

Participant: She would take them during the day. Now that she has a car and I have a small car at work as well… it works out really well, thank God.

Riham: Thank God. That's really good. Congratulations.

Participant: Yes, while I am at work she takes the kids out.

Riham: We spoke earlier about the detailed questions regarding what home prevention or remedies you use as a father to promote your children's health. Can you tell me about the means you use and where you learned them from?

Participant: So we use olive oil. If someone sprained or pulled a muscle, has back pain or joint pain we use olive oil. Massage it in. We use some herbs and such as well. Thank God.

Riham: Herbs you mean.

Participant: Yes, herbs. Ginger as well. Especially in the winter. Ginger is really good. It warms you out.

Riham: So when you were younger did your grandma or mother or maybe other family members use these remedies or did you find yourself using them more here?

Participant: No, it's the opposite actually. We used them a lot more back home. Back home, a lot.

Riham: Why'd you stop then?

Participant: With every generation that passes these things get forgotten as they say.

Riham: So in your opinion the more a person is distant from their homeland the more they forget herbal remedies?

Participant: Absolutely.

Participant's Wife: It's not just that we forget. In our homeland, things were more blessed and had more flavor. Everything we used to eat was organic and fresh. Here it's not like that.

Participant: Back home if you would walk next to someone who sells herbs and oils…and order a little bit of this and that he would have it all. You could find anything you wanted there. If one doesn't go to the store often they would order a little bit of everything and keep it at home. It's all useful and beneficial.

Participant's Wife: We used to have fresh milk --.

Participant: Fresh milk and fresh meat. We had a field. Our veggies were fresh from our own garden. Everything is fresh, unlike here. From the farm directly to our plate. You find it frozen here. Here when we buy meat we don't know where it's from. What is this halal.

Riham: God knows where it's from.

Participant: They tell you it's halal but no one has seen the way it was prepared.

Participant's Wife: Even in Syria we wouldn't buy it frozen.

Participant: Yeah, in Syria no one eats meat that was frozen. They don't sell it that way either. It's all fresh. For example, I would want to buy a whole chicken. I would order it from the butcher. He would then slaughter it, weight it, skin it, clean it and put it in a bag for me.

Riham: Here in Cincinnati?

Participant: No, no.

Riham: In Michigan, in Dearborn, you can find the same exact thing. I was telling your wife that I was from Michigan. Ahmad's family is also from there. It's as if you are in an Arab country there. Honestly, I don't like this type of butcher that kills the chicken right in front of you…I don’t go because I get grossed out. My mom goes. Do you know this butcher?

Ahmad: No, I don't know him.

Riham: Yeah, right in front of you they slaughter a live chicken. It's on Miller Rd. They do it all in front of you. I don't like it but my mother does.

Participant: That's the way it should be! To say “Bismillah” before the slaughter.

Riham: Yeah but being raised here we don't really like these things.

Participant: When you get it frozen only God knows where it came from. We went to Chicago. Not Michigan yet.

Riham: You would like it if you visited. All the signs are in Arabic. The butchers, the grocerer, the bakery, the herb shops, all just like back home. Everyone speaks in Arabic, an area called Dearborn. Even for me..coming to Cincinnati, was a challenge. I felt foreign here. How do you feel about coming here to a foreign land?

Participant: When I would go to the organization…I would tell them about our state.

Riham: What do you mean?

Participant: For example, they would explain everything to us. They drew two lines on the board for us. They would say that we were now at the top. That we are currently feeling really happy. Then they said that we were going to go all the way down to the second line and start crying and feel sad. Then they explained that we would be climbing back up one step at a time. These lines represent our experience. There was an interpreter with us. I asked him, which America was this that they were talking about? They would bring a refugee then after three months tell him to go and manage on his own? Where is the logic in this?

Riham: It's not logical.

Participant: I am going to ask you the same question. Imagine I took you and placed you in an Arab country for one year. Let's say one year and not three months! I give you one year. Will you be able to manage on your own? No, absolutely not. How am I supposed to manage on my own here? Us Syrians are now scattered all over. We have filled all the corners of the earth. When it comes to seeking asylum, America is known to be the worst place for a refugee to end up. It is the worst.

Riham: You know this fact by speaking to other refugees that have sought asylum in different countries or is it just based on your experience?

Participant: No. I am speaking based on the experiences of my friends, my relatives --.

Riham: They are in different countries now?

Participant: I have them all over. I have some in Australia, in Germany, in Sweden and all over. They are all told that they are prohibited from working! If you're a refugee, you are not allowed to work.

Riham: How would they manage living there then?

Participant: Everything is provided for them.

Riham: For how long?

Participant: Some have it up to two years and some until they learn the language. They focus on learning the language.

Riham: Wow. That's very different.

Participant: In Austrailia, a refugee is not allowed to work if he did not learn the language.

Riham: So if someone doesn't learn the language, what happens to them?

Participant: Everything will be provided for him. For example, I have been here for about a year. I can understand the language but I cannot speak. But I can get by. A year later. After two years of being here, I think I would know enough to get by. It's a reasonable time.

Ahmad: You're also working so it's hard to feel at ease or find the time to study and learn.

Participant: Exactly. At the beginning, when I arrived, I would wake up and BANG BANG in my head, all the thoughts and stress. But now. I have no time to think or anything. I wake up in the morning and need to get to work. After work, I need to get home. That's the only thing on my mind.

Ahmad: You have no time for anything else.

Participant: I don't. Here a person isn't able to breathe.

Riham: Okay so when you chose the country, did you know --.

Participant: No, no there is no choice.

Riham: You didn't get to choose?

Participant: No. They speak to you and inform you that they arranged for you to go to America.

Riham: Oh, so they choose?

Participant: They choose.

Riham: Even if for example, you told that you had a brother in Australia?

Participant: No.

Riham: It won't make a difference.

Participant: No. They would say that currently there is a chance to go to America. That's all they tell you. If you don't want to go, then don't go.

Riham: You would miss your chance of leaving then?

Participant: Yes, you would miss the chance. You won’t get the chance to leave.

Riham: That's not easy on one emotionally either. You are a refugee, to begin with and then you feel like you are forced to do something and then forced when you arrive.

Participant: It's not easy. Don't think that they tell you how long all this would take. They would just tell you that you are leaving. They don't give you time. They don’t tell you how long you will wait to travel.

Riham: Oh, they don't tell you everything.

Participant: No. We would be going and coming back frequently. Interview after interview. They would request you come at 7 am and you don't leave until 5 or 6 pm. The whole process wasn't easy The process to leave! It took over a year. They ask you questions about every single moment from the moment you were born till now, today!

Participant's Wife: They would interrogate us. They are afraid of bringing bad people into their land. They have a point.

Riham: The point that I want to make clear was whether or not everything was explained to you. For example, regarding the three month period.

Participant: Everything was explained.

Participant's Wife: They didn't say that we had to learn the language during that time and then we will be on our own covering all your expenses!

Participant: No, no, no. We remember at the time that the only thing on our mind was our children. I didn’t think of myself. We thought that if we came here they would have the chance to study and prosper. My own self. I did not think of myself. If I were thinking of myself, I would wonder how I would make it here with the language barrier and such. It didn't even cross my mind to think of the language barrier! All I was thinking of was my children. I wanted to bring them here to study. Thank God, that part is good. It's really good.

Riham: Thank God. What are some things you hope your children would attain from living here? You mentioned the education aspect. What else?

Participant: God willing my children study and turn out to be great. Hussein has set his mind on Dentistry.

Ahmad: God willing.

Riham: God willing.

Participant: This son as well. This son…

Riham: What does he want to become?

Participant: A doctor, God willing. Their grades are really good, thank God. All of them.

Riham: May God protect them. Parents really sacrifice a lot for their children.

Participant's Wife: This one [points to son] has been doing well in school even in Syria. He would always rank 1st. He was in first grade and excelling! He had excellent grades.

Riham: That's great. What do you worry about for your kids?

Participant: The schools. The schools. I am afraid. I worry about certain things they might face in school. Things may not turn out well. The friends they make. We hope they make good friends. If they befriended the wrong person they might get lost. I worry about this. It scares me.

Riham: Okay, the friends. Is there anything else? Your wife was telling me that you didn't like your kids to ride the bus alone. Is that right?

Participant: Yes.

Riham: What are you afraid of?

Participant: I didn't like the bus. I rode the bus a considerable period of time. When I would ride the bus I would see all types of people. People that weren't good. They way they looked. Their actions and words were bad. They weren’t good people on the bus. We didn't want them to see the things we saw.

Riham: What are some things you are doing with your wife at home to ensure your children don't fall into the things you don't want them to fall into? Like to befriend the wrong person as you mentioned, for example. Or to fall into things like drugs, alcohol and so on. I am mentioning these things that most parents fear for their children.

Participant: As for me, I like to have a close relationship with my children. That way they come and tell you everything that goes on in their lives. Anything in their lives. Especially if they have an issue. So I can guide them what to say or not to say. Whether it be a boy, or a girl, if they befriended the wrong person, they will get ruined.

Participant's Wife: Even if my daughter would tell me there is a classmate at school that is lazy. If she befriends her, she could make my daughter lazy as well. If they do drugs or aren't hard workers they might want to bring down those that are better than them.

Riham: That's true. Out of jealousy?

Participant's Wife: Exactly.

Riham: So you try to get close to your kids and have a good relationship with them.

Participant: Yes. Thank God, I built a friendship with my kids.

Riham: Do you take them to work with you?

Participant: Hussein is very eager to start working. He keeps asking us.

Riham; They are young men now.

Ahmad: They have the passion for it.

Riham: May God protect them.

Participant: Hussein will tell you he wants to go to Michigan now. [laughs] He will tell you I want to go work in Michigan! You made him desire it.

Riham: Have you explored jobs for teenagers? Did you ask about jobs for kids that have a lower age requirement? It can be something like a golf caddy or a dog walker.

Ahmad: There are some jobs your children can legally work at now.

Riham: For example, If the girl wants she can be a babysitter. She doesn't need to be a certain age legally.

Participant's Wife: No, there is a law. We went to Meijer and asked. They said to go to Kroger because they usually hire younger kids. We went to Kroger, and they said a child needs to be at least 15.

Riham: How do you keep them entertained then?

Participant: Here. They entertain themselves here.

Riham: There are seven of them. I am sure they keep one another entertained.

Ahmad: Okay what do you feel the Syrian refugee community needs here? What things do they need?

Participant: Things?

Ahmad: Is there anything missing you wish was provided when you arrived?

Participant: I don't wish for anything.

Riham: How about needs. Not just things.

Participant: Or needs. When it comes to things, everything can be bought later. The person who works can buy things. They can buy anything. The organization supposedly furnished the house. How did they furnish it? By putting these two sofas. If I saw these on the street I wouldn't even take them. Everything else is available thank God. Little by little a person gets the things they need and want.

Ahmad: Are there any intangible needs that you wish were provided? That may have made your lives easier?

Participant: No. It won't make a difference.

Riham: What about programs? Like I am thinking more in terms of programs rather than things or money. Programs geared at children and families to help them during their transition. What do you think?

Participant: They should. Legally they should have programs that meet the needs of the children.

Riham: If you were to design such a program, for children, what would you include in it?

Participant. As a program for my kids? Monetary benefits. In Canada, each child receives $450 a month till they turn 16 years old.

Riham: Yeah.

Participant: When you take a child into a store, they're going to need clothing. They like a pair of jeans or a shirt…shoes, a toy even. I would want to get it for them. But. There needs to be assistance.

Riham: Okay so monetary assistance, clothing, and work to keep them busy as I heard you say earlier.

Participant: Of course.

Riham: Okay your son also suggested a program for drawing. I would like to hear examples more like that.

Participant's Wife: If they have a talent, they should be able to work on their talents.

Ahmad: So you are saying programs to foster these talents and provide them with skills they need to be able to work with them in the future.

Participant: Yes. Programs to help their mental state and to help them make good use of their time.

Riham: Of course. If one makes good use of their time they feel better psychologically. Do you have anything else on your mind or any suggestions that we did not speak of? Regarding the topic of health for your kids.

Participant: No, it was a good conversation.

Riham: Or anything else that a refugee might need in terms of health?

Participant: No, everything was covered thank God.

Participant's Wife: The eyes and the teeth are not all covered though.

Participant: Yes, insurance does not cover teeth completely.

Riham: Oh, I see. What do you mean it doesn't cover it completely?

Participant: For example, if the bill is $1000 only $500 would be covered.

Riham: When I asked you earlier, in the other questions, about medical expenses that are not covered completely, you said there weren't any expenses.

Participant: Yeah this one isn't covered. The dentist. Extractions.

Riham: So you did find difficulty in this cost?

Participant’s wife: I had extractions and the cost wasn’t covered. And the glasses.

Riham: The eyes and teeth then. Well, your son will become a dentist, God willing, and that will cover it. The other son can be an ophthalmologist, and you will be covered someday! [laughter] Thank you so much for your time.

Participant: No, thank you for your time.

6B Interview

Ahmad: We are with participant 06B. Today is July 12, 2017. I am with Riham Alwan.

Riham: We are here with a mother of seven kids. She has been in this country for a year and a half now. We will now begin.

Ahmad: Let's begin ma’am. Can you tell us a little about your experience with the medical system when you first arrived?

Participant: It was really good when we first arrived. With respect for the kids,it was good for the kids, especially. Not so much for the adults, but great for the kids.

Ahmad: Lets back up a little. Why did you decide to come to America?

Participant: We went as refugees from Syria to Jordan. In Jordan, they spoke to my husband about an opportunity to migrate. They told him an opening was made for us in America. He told them okay we will go. Then, we spent about a year and eight months doing interviews prior to coming here.

Riham: How long were you in Jordan for? It must have been a long time.

Participant: A little over two years.

Riham: Oh, wow.

Participant: They kept interviewing us for about a year and eight months. We were also delayed because I was pregnant with Yusuf. It was prohibited to allow a pregnant woman to fly. They waited till I gave birth. Four months after my delivery they called us again.

Ahmad: Okay. How was the medical situation in Jordan?'

Participant: It was excellent, thank God. Honestly, we had no complaints when we were in Jordan. When we came here, that changed. All the diseases descended on us.

Riham: May God protect you all. What happened when you came here?

Participant: In Jordan, I was doing really well. When I came here, I developed diabetes and became anemic.

Riham: What else?

Participant: In Jordan, it appeared that Yusuf had a normal head. When we came here they told us that it wasn't and that he had extra fluids in his head.

Riham: Yeah?

Participant: They said maybe it's normal or maybe it's not. They wanted to observe it longer. They did a scan and then said this. They did another scan when he was six months old and said it was still there but did not grow in size. They will do another scan when he is three years old. They said if it stays the same size, then we will not worry about it. However, if it increases, God forbid, then we will discuss then.

Riham: I see. What made you think there was anything wrong with his head, to begin with?

Participant: I didn't think anything at all. I was taking him to see a doctor regularly. Especially when we first arrived. We would go every two months for his vaccinations and check ups. She used to measure his head and she saw that the way his head was growing was unusual. It's supposed to be like this but she saw that it was going this way [shows with her hands]. It wasn't normal she said. The doctor said we should do a scan. After the first scan, they said they found fluid in his head. It seemed like he had pressure over his eyes according to the scan. We then took him to an eye doctor. Thank God, he said there is nothing wrong with his eye! Later, however, they told us we need to do another scan to check the fluid levels. Thank God, it was still the same. The water hadn't increased. She said if God forbid it grows then a problem will arise. Now, however, everything is fine now. They said perhaps when his head grows the fluid will too so they need to wait till he becomes 3 years old to re-scan.

Riham: So when this happened you had to go from one doctor to another, different subspecialities. Did you find any difficulties with transportation or knowing which doctor to go to?

Participant: In terms of the transportation, we have a group set up from when we first arrived. May God reward them. They were about 5 or 6 American women that take turns taking us to our appointments. They would even take care of making the appointments and everything. I don't talk to the doctors myself or make the appointments. They would do it and tell us. Tomorrow we have an appointment for the vaccines. They called and told us we will come and pick you up for your appointment. They would take the kids and get them vaccinated. We went to a see a neurologist for my son’s head. They did an MRI and then sent us to another doctor to fully examine his physical abilities and walking and movements and such.

Riham: Yes, ah like a developmental pediatrician?

Participant: Yes. And they sent us to an eye doctor and one to test his hearing. The results from all these were all good, thank God.

Riham: Thank God.

Participant: When he turns three we will see again, God willing.

Ahmad: Was this group helping you from the time you arrived?

Participant: Yeah. They would help us more than a family member, more than a sibling or a cousin, they would help. They helped so much.

Riham: That's great.

Participant: They did not leave us on our own from the time we arrived here till now. The woman made an appointment for my children's vaccines on her own and called to inform us about it. We're going tomorrow. My son Hussein went to school for the last month and he was vaccinated. We knew they vaccinated them but I don't know which vaccines they were. Yesterday, she went to the school and brought their paperwork. She found that they don't need to be vaccinated again because they already had them done before.

Riham: What is the name of this organization? It is unfamiliar to me. Are there also other Syrian refugees being helped by them or just you?

Participant: Who?

Riham: You mentioned this group of four or five women that help you with certain matters. What is the name of this group?

Participant: They are just women doing it on their own. When we first arrived, one of them came because she heard from a friend of hers about a family coming with seven children. They all wanted to help. Do you know what I mean? They took us where we needed to go and took care of our appointments.

Riham: Do they speak Arabic?

Participant: No.

Riham: How are you able to communicate?

Participant: At first we were communicating by using the phone but now we are able to understand one another. We can speak to one another.

Riham: That's really good.

Participant: Yeah, it's better than before.

Riham: Do they help other refugee families?

Participant: Yes, they help other refugee families but they aren't Syrian. Every Syrian family that comes has people assigned to help them. If any Syrian tells you no one is coming to help them then they are lying! Honestly, even the refugees. When I would go to my lessons for the women, the other families would be dropped off by foreigners. They bring them and take them back home.

Riham: So every refugee has someone assigned to taking care of them.

Participant: Yes, they find a few people to take care of them.

Riham: Who are these people? Is it Catholic Charities, the organization?

Participant: The organization would know about the families that arrive. Who supports the organizations? The churches do.

Riham: Yeah. When you came it was at a different time than most refugees. Now I am hearing people come through Catholic Charities.

Participant: Yup that's it. Who supports them? The churches and the donations that come from the churches. When help is needed, the church announces it and people volunteer to help. So now every Syrian here has people taking them around and assisting them. All of them. Participant 5B has people that are helping her. I know that woman and her husband. There are other people that I know as well that go and study in their church. For example, there is also Abu Ahmad. They also have seven kids and… their son is sick. They also have people that take them around. There is Mayadah as well. She gave birth yesterday and also has people that take her around. They take you places and bring you back home. As soon as one is able to stand on their own two feet then they stop. As soon as you're able to get around and have a car then they don't drive you anymore. When my husband wasn't working, I would go with him.

Riham: Your husband has a car?

Participant: Yes, thank God.

Riham: He already knew how to drive?

Participant: No he has been driving for 20 years.

Riham: Oh, okay so he knows how to drive.

Participant: Yes, he knows how. I also got my license here but I didn't know how to drive before that.

Riham: Okay, that's good for you. You learned.

Participant: Yes, I learned and got my license. There was a person from the group that taught me how to drive. The mosque also helped with three lessons. Each was an hour long. They also helped.

Riham: That's really good.

Participant: Yeah, but the mosque knows these people that are helping us. They would reach out and request that he teaches us. He is around 60-65 years old. He is the husband of one of the women that comes and helps us. I would invite them over and they would come. I would throw them parties, cook dinners. If one of my kids has a birthday party, then they show interest in coming. You know they love Arabic food so I have them over for lunch or dinner. They all come over with their spouses.

Riham: My God bless your hands.

Participant: You as well. One of the women has five children and she brings her kids with her.

Riham: That's really nice.

Participant: Yeah. If someone is kind to you, you have to be kind to them. We're forced to. You know as the saying goes, "Sweet words bring a snake out of its [indistinct]". What they are doing for us is more than what a family does for one another!

Riham: Can you say it again because I didn't hear it.

Participant: They say, "A kind word brings out the --.

Ahmad: It brings the snake from its cave.

Riham: Oh, the snake from its cave.

Ahmad: I am sorry not cave, I meant its den.

Riham: Okay I understand now.

Ahmad: Sweet talk.

Participant: You don't even know. They tell us you are like our sister and our friend. No one is forcing them. They say that they hope Syria will have peace and everyone will be able to return. Except for you, us. "We don't want you to go back", they said. We told them that we would take them with us if they want! SO they are really really good people.

Riham: May God preserve your friendship.

Participant: Yeah, it developed into a friendship between us.

Riham: That's really nice. Can you tell me a little bit about --. I know your situation is a bit different than other refugees I speak to that have been here for only a month or two. Can you tell me when you started to feel friendships forming, safety, and that people around you were willing to help? How long did it take for you to start feeling this way?

Participant: Honestly, I felt it from right when we arrived.

Riham: From when you first arrived.

Participant: Yes, right when I arrived I felt this way. They would take us and bring us and do so much for us. Excuse what I am about to say but no one is obligated to do anything for anyone. They aren't obligated to do anything for us. They are doing it for the sake of God. From the time we came till now people are telling us that these people are helping us only to take us to the churches and convert us to Christianity.

Riham: Yeah.

Participant: That's what they are telling us.

Riham: Who is telling you?

Participant: A lot of Arabs.

Riham: Oh, Arabs. Okay.

Participant: Yeah they are telling us that they aren't coming to help us because of our difficult state but rather because of your kids. That in the future they are going to come and take us to the church and convert us to Christianity. These five families attend a wealthy church. There was even a time when my husband couldn't pay rent for like two months or so. The mosque was helping him at the time. They told him we will talk to the church and see if we can find you a job. Like cleaning the bathrooms in the schools.

Riham: There is nothing wrong with that.

Participant: Yeah. He said he would rather work this type of job than stand around asking others for money. So he went for an interview. They agreed to $15 an hour. That's excellent.

Riham: Yeah it is.

Participant: He told him he would get $15 an hour and if he works on Saturdays and Sundays then it is considered overtime.

Riham: Wow.

Participant: Yeah he said they have events and such and if he works on Saturdays and Sundays then it's considered overtime. They told him he will be happy working there and such. Honestly, between me and you, we were overjoyed with the offer. Then we met with some Arab families and they started telling him not to do it. You know when we were the first arrived; we were the only refugees.

Riham: Yeah.

Participant: All the Arabs here said they didn't want him to go work at the church.

Riham: Why does it concern them?

Participant: They kept saying that they aren't really trying to help us but rather want to take our kids and convert them to Christianity. Honestly, my husband was not convinced. He said he wasn't a child and won't be easily manipulated by others. He thought what they were saying was nonsense.

Ahmad: Yeah.

Participant: They also sent some Arab women to come and talk to me. I told them there is nothing wrong with it. They started saying they had a friend that owns an Indian restaurant and he wants to pay $16 an hour. They said isn't that better than $15 an hour and working at a church. He said he would take my husband there. My husband eventually gave in. He said since they found him a job, and it'll keep them on good terms he accepted. If we accepted the job at the church they were going to find us a house around there since it's 45 minutes away.

Riham: Oh, it's far.

Participant: Yeah it's far. My husband is now working in Kentucky though!

Riham: Oh wow.

Participant: It's an hour and a half commute.

Riham: Oh no.

Participant: Yeah. They said if the church is far then we will rent a house out there for him. So that his job isn't too far from his home. So the Arabs started to say that they want to take him and move him and I don't know what. That's when my husband decided it’s better to stay here. All this was considering this Arab man and the owner of the Indian restaurant –He is from Syrian, Homs, but he was born in America. The Arab man said he is very close to the owner of the Indian restaurant and all. They told him we have someone to work for you. He said he can work there on the condition that he works Saturdays and Sundays as well. From 6 am till 12 am.

Riham: Oh no!

Participant: My husband said it is okay. He is content with the fact that he can get us some financial security. So he can take care of his kids. He didn't complain at all. He started leaving at 6 am and coming back at midnight. He would wash the dishes. You know how that is. This kind of work.

Riham: Of course. It can cause back and hand pain.

Participant: Yeah, he kept working like that for a month. Not getting paid. Finally, he called his friend and said he didn't get paid yet. So he told him, don’t worry I will talk to him. So the man finally wrote him a check. He gave my husband $1,200.

Riham: That means he didn't give him the right amount.

Participant: $1,200. He spent 40 days working there and telling him I have bills to pay. I have rent, electricity bills and water bills. His friend who is supposedly a lifelong friend of the owner talked to him. The owner then said I still have to give him 200-300. He replied that's not enough, the man worked for 40 days and didn't even have Saturday or Sunday off. All that for $1,500. He said that's all he gets from me. Turns out he is paying him $5 an hour.

Riham: Oh no!

Participant: If he doesn't like it he can leave. He said he is washing dishes all day, “why would he pay him $15 an hour. It's $5 an hour. Who does this man think he is?!” ….tsk tsk tsk …All this happened because he didn't get him the job through the organization, he did it the Arab way. After 40 days, he only got $1,500. On top of that, he says if you like it then stay, if not, then you can leave. My husband of course refused. He also knew that he couldn't complain or anything because there was no contract between them. There is no paper to prove anything. He just quit at that point. He spent two months unemployed.

Riham: That's terrible.

Participant: Yeah. After two months he said he wanted to go back to work. We tried to get him the job at the church back, but it seems like they were a bit upset. They went and spoke on his behalf and he didn't take the job. I think they were offended. The priest found someone else in his place. He missed out on that job. All this because of the issue of Arab and non-Arab and things like that. Can you imagine that if someone is scammed by someone from the church they would pay money from their own pockets to repay the debt to that person rather than let the debt go unpaid! My husband told them that he didn't know English and cannot speak or read it. How can someone who knows English, referring to the restaurant owner’s supposedly a lifelong friend, the Arab man, misunderstand the $16 an hour and full-time offer? His friend said it’s true that he said all that…but now the owner is saying that we misunderstood him. He now says that someone who washes dishes should not be getting more than five dollars an hour. All that hard work for 40 days was lost. All those hours.

Riham: That is unfortunate. Where did he find a job after that?

Participant: After that, he was unemployed for two months. The Rahma organization from the mosque --.

Riham: Yeah?

Participant: The owner in Kentucky is also Syrian. The caseworker from Rahma talked to him and told him that my husband had been unemployed for two months and has a family and that they paid for our rent this month, and he is not able to find a job. The man said he will give my husband a try for a month. If it works out then he will stay and if not then they will not continue. He said this to prevent any hard feelings. My husband went and he told him the same thing and that he should try it out for a month.

Riham: What does he do for work now?

Participant: Washes cars.

Riham: Oh okay. In Kentucky.

Participant: Yeah, in Kentucky.

Riham: It's at the same job then. He ended up staying.

Participant: Yeah. Thank God, he's been there for a year and two months now.

Riham: Thank God.

Participant: The owner liked him and my husband ended up staying in the job.

Riham: Yeah. These types of things you mentioned, like the problems with bills and the job situation, they can really weigh heavy on you.

Ahmad: These things can really cause stress.

Riham: They can cause stress for you and your husband.

Participant: When we first arrived my husband was of healthy weight. If you look at him now he's like a stick.

Riham: As in he lost weight?

Participant: No, he didn't lose weight. It was from the stress and worries.

Riham: You said he was heavier.

Participant: Yeah he was heavy and now if you see him he is a stick.

Riham: Like he lost weight.

Participant: Yeah, he lost weight but he wasn't dieting like some people would do. He did not lose weight on purpose.

Riham: Oh, I see….so due to the circumstances he lost weight.

Participant: From the burdens and pressure. From the exhaustion. Now whether it is snowing or sunny he works outdoors. Those that are educated their jobs are behind desks and --.

Riham: Yeah, but he has to work outdoors.

Participant: Yeah and especially since our situation in Syria was good. I don't mean it was a little good, I mean it was great. We had been comfortable.

Riham: Thank God. What was your husband doing in Syria for work?

Participant: Anything that crosses your mind.

Riham: Okay.

Participant: For example, we had two gardens [fields] adjacent to one another. One for my father and one for us. He and his father used to buy and sell cars. They would buy and sell houses.

Riham: Oh, so a businessman.

Participant: Everything. Motors and his brother owned a supermarket that he also worked in. Anything that crosses your mind, he worked in it. [Indistinct]. We had a house on the second floor, and we were renting out two apartments on the first floor. We also built a terrace during the war and thought the Uprising would end within a month or two. We also had 2 million Syrian pounds that we bought a huge land with. We thought the war would end soon.

Riham: That's what everyone thought.

Participant: We had investments and expected to gain from them. We had many homes we rented out. We had an income….the Uprising didn’t end. Then we left it all in Syria and went to Jordan. The situation was much different there. By a lot not a little. Let's leave it to God. Thank God everyone is in good health.

Ahmad: Thank God.

Riham: Thank God.

Participant: The most important thing is good health.

Ahmad: Regarding these difficult times you have all been through, how did it affect your psychology? What are some things you try to do to improve your emotional health?

Riham: As parents and then as children.

Participant: As parents, it’s due to wanting our kids to get an education.

Riham: No, as to how did it all affect your mental state?

Participant: Of course it had a huge impact.

Riham: Can you tell me a little about it?

Participant: What do you mean?

Riham: As in do you get anxiety?

Participant: A lot of it. It would make one cry. A person would be in a very difficult state. There are a lot of things here that are very different than what we are used to. It's even different than Jordan. For example, when we went to Jordan we didn't feel the way we feel here.

Riham: It's foreign.

Participant: It's very foreign. In Jordan, I had my siblings and he had his. We had our relatives there as well. We didn't feel the way we felt here when we first arrived. Especially since when we first came we didn't know a single Arab person. We spent two months here before we met any Arabs.

Riham: Really? Not a single one?

Participant: Not a single one! Why? Because we were told that we weren't allowed to meet Arabs. They would only bring us the interpreters.

Riham: The interpreters wouldn't tell you that there is a community here?

Participant: No, they weren't allowed to. It was against the rules. A Catholic Charities person would come with us and leave with us. It was prohibited for them to talk to us. Later when we asked them they said we weren't allowed to talk to you. "We are only able to translate", they said. “I can only translate verbatim.” They couldn't speak to us regarding other topics.

Riham: Right, because that is their job.

Participant: Because we were the first refugees, and they were worried about our safety. They didn't even inform the mosque at that point. They didn’t want us to be associated with the mosque. One woman was telling me how they heard about a Syrian refugee family coming and when she went to inquire, she was told it wasn't any of her concern. She said she was scolded and kicked out and told to stay out of it. Then coincidently, as this organization took us shopping one day at sams--.

Riham: Sams?

Participant: It's a store called Sams Club. No, I am sorry, it's called Jims. Jumbo Jim’s [Jungle Jim’s].

Ahmad: Yeah, I have been there.

Riham: I haven't been there yet but I heard of it.

Participant: So after two months of being here we told them we wanted some bulgur. As you know. It is one of our staples but we did not find it.

Riham: Yeah, of course, I know it. I go to Michigan for these things.

Participant: Yeah. There is nothing here. We need bulgur, and we need the Egyptian rice. They told us that Jungle Jims has Middle Eastern things like that. They didn't know what the bulgur was so they asked if anyone speaks Arabic there. Then a Turkish woman came. She didn't know any Arabic, but she came to ask us where we were from. We told her we were from Syria. She went off running. Then a young man came named Basil. He was from Palestine. May God preserve him for his mother. The Turkish woman had called him to come help us.

Riham: That's really nice.

Participant: He introduced himself and said, “I am Basil from Palestine. You won’t find any of those things here,”…He told them they should take us to the Halal Market. The people with us said no no we can't. That we didn't have permission.

Riham: Why?

Participant: I don't know. So no one can know we were there. It isn’t allowed. We said please, we want someone who speaks Arabic. We were so happy when we saw Basil!

Riham: Yeah, of course.

Participant: We couldn't take it anymore. Basil asked for our address but they refused to give it to him. It wasn't allowed, they said.

Riham: Oh no.

Participant: We said [whispers] “Basil please follow us from afar.” He did. He followed the cars that brought us and he saw where we lived. He then informed people at the mosque.

Riham: Oh my.I don't understand. You are in a foreign land and all you want is to interact with someone that knows your language.

Participant: They were worried about us. The mosque then sent some people over to our house. They were three women. As they were coming, someone from Catholic Charities saw them and went and informed the organization. My friend later told me that they received a call from the organization questioning their visit and telling them they were not to return again. They told them they were not allowed to be near us.

Riham: It's a free country, supposedly. Everyone is able to go and come as they please.

Participant: We spent four months not seeing any of them. We asked them why they didn't come around and they told us what had happened to them. That they weren't allowed to come near us because of the organization [Catholic Charities]. One time there was a conference at the mosque, and when the mosque called to say they wanted us to attend the event, the Catholic Charities organization said we will bring them. But. They didn’t take us. They didn't even tell us about the event!

Riham: Okay so if your husband wanted to go pray Friday prayer, where would he go?

Participant: They took him. One of the men took him. They took him to Clifton mosque though.

Riham: Okay so he didn't meet anyone there?

Participant: No. He said people were going to pray there, and how was he supposed to approach them.

Riham: Was he going every week?

Participant: No not every week. If the man was free he would take him. For example, he would say he was free this Friday. He would take him and wait outside for him till he finishes to take him immediately after prayer.

Riham: Okay.

Participant: He took him twice to Clifton. Only there.

Riham: Eid didn't come during that time?

Participant: No, it didn't.

Riham: Yeah, Ramadan was in the Summer.

Participant: It was in July.

Riham: Yeah, that is right.

Participant: By then some Syrian refugees had come.

Riham: So you became a big group.

Participant: No, then Ibtisam came. Then Um Maher came. Do you know her?

Riham: Yeah, but I have heard of her.

Participant: Then two more came. That's when they started telling people. Now every time someone comes they tell them about the mosque. If a Syrian family comes they requested that people from the mosque go and greet them.

Riham: Yeah, that's better.

Participant: They saw that the mosque was helping us. When we first arrived, they had only given us a couch here and a couch inside. These couches if you were to see them on the street you would not take them. They also put a table and some chairs for us. If you only saw the condition of these chairs. On top of that, they charged us for them! Then the Ihsan Mosque came --. Do you know the Ihsan mosque?

Riham: Yeah. You mean Rahma.

Participant: No. Ihsan.

Riham: Which one is Ihsan? The one in West Chester?

Participant: No, I honestly don't know where it is.

Riham: Mason?

Participant: No, it's the Mason one.

Riham: How many Mosques are there?

Participant: There are four.

Riham: I only know three. I guess I don't know the Ihsan mosque.

Participant: There is Mason, West Chester, Ihsan, and Clifton.

Riham: Yeah, then maybe I don't know Ihsan. Is it far?

Participant: Yeah, it's far. From here it's far.

Riham: Okay.

Participant: So at this mosque, the Egyptians joined together and got me this for example [points to furniture]. These things over here as well are from another group of Egyptians. We got rid of all things that were here when we first arrived. The women came and told us to throw them all away. They said they would buy us everything we needed brand new. It will come from people that donate specifically for those in need. We would then go to the mosque and choose. Everything you see here, they provided it. Thank God. The organization [Catholic Charities] just furnished something to get you to sign a paper and make you pay for it.

Riham: Yeah, I heard about that before.

Participant: Yeah even the way they bring you. For us, it was $955 for each person. How many people are in my family? We are nine. Right, when we came we saw that they were charging us $5,000 for the home.

Riham: What?! $5,000!

Participant: The rent was $1,250. Security deposit was also $1,250. Then rent for 3 mo.

Riham: Security deposit? You're supposed to get that back though.

Participant: Yeah, but no one gets it back here. They said no one gets it back. So they told us we need to pay the $5,000 back that they paid for us. All the furniture..even though it was garbage…the furniture inside came out to $1,500. Once you sign the papers you are indicating that you are no longer getting this money. When we first arrived we couldn't even get food stamps right away.

Riham: Yeah, you have to wait a long time.

Participant: You have to wait a while till you're able to get it. If you wanted to buy groceries or what not you had to buy it all. When they took us to Jim’s, we had to buy it all.

Riham: Okay, when we are discussing physical health, finding the right types of food for you is important. The bulgur, the rice, the olives, the halal meat are some examples of foods you use as a Syrian.

Participant: I didn't find it at first.

Riham: The question is what you do when it comes to ensuring your children get nutritious food?

Participant: I didn't really understand the question.

Ahmad: You were telling us about how when you first arrived you found it hard to find some of the groceries you were looking for as well as the food stamps issue. What are some things that you did to ensure your children received nutritious foods and maintained good physical health?

Participant: We were sitting at home.

Ahmad: Okay. How about regarding the food? What were you able to cook at that time? Were you able to feed them fruits and vegetables?

Participant: Yeah, we used to buy fruits and vegetables. When we first arrived they took us to Kroger. The next time they took us to Mejier. And Wal-mart. We bought from there but they both didn't have any Middle Eastern groceries.

Ahmad: Okay.

Participant: And when we first arrived, we bought a few things with us.

Ahmad: Oh, okay so you brought some with you.

Participant: Yeah, we did. It was permitted for us to bring things… except any oils and such. The white powder was also prohibited. So we brought with us some things. I also brought some kitchen supplies with me from there.

Ahmad: Okay. So even without food stamps, you felt you could still ensure your children get proper nutritious food?

Participant: They gave us $500. From our own money.

Ahmad: Oh, okay.

Participant: Yeah it was from our own money. In a form of a card. They told us to make do with it until we get the food stamps.

Ahmad: Okay.

Participant: We managed and went and bought stuff. However, we were a bit surprised by the prices at first.

Ahmad: Did you find any difficulty using the groceries they have here to make something nutritious?

Participant: No, because there are somethings I make from scratch and some things I buy. It depends. For example, the grape leaves dish cannot be made except by using middle eastern groceries.

Riham: You don't know how to pick the leaves from outside?

Participant: No.

Riham: Even though my Arabic language isn't great, I am still an Arab. And a Syrian. I know how to pick them. Then you boil them!

Participant: Yeah, that's true. Except you don’t boil it. [laughs] You just need to place it in hot water and soak it.

Riham: Clearly you see that I don't really know. [laughs]

Participant: No, you're great. Thank God for good health. That is the most important thing.

Riham: Thank God.

Participant: If a person has all the wealth they need but are not in good health then they would rather give it all up for good health.

Riham: Yeah, of course. What are some other things you did that promote your children's good health? For example, do you let them play outside or at the gym?

Participant: No, honestly, I have not been good at that. I regret this. When we first arrived we couldn't go because we didn't have a car. When my husband got a car from a group of Arabs. They all pitched in to get him a car. Some were Egyptians and some were Palestinians. They bought him a car. 26 families got together. They put a donation box for the Syrians. Now when any Syrian comes, they buy them a car from the money in that fund.

Riham: God willing.

Participant: Any Syrian that comes they get a car from the money in that fund. During the time we came, however, we were helped by 26 families with the car. My husband would go and come with the car. It wasn't available. If my kids signed up at the gym someone will need to go pick them up. It's wasn't possible. There wasn’t extra time to use the car. Maybe next year it can be an option.

Riham: God willing. What are some other ways you help them cope and adjust to their new life here? How do you help them deal with their feelings?

Participant: I help by ensuring they stay successful in their studies. We don't want this to go to waste. When we decided to come here, all we were thinking about is our children's education. In Jordan, there wasn't an option for Syrians to get educated.

Riham: It wasn't?

Participant: There is but it's a lost cause. It's either you pass or fail and the teachers in Jordan are really tough on the Syrian students. Really tough. Everyone has it different in their country. We are used to a different system. You can't go up to a teacher in Jordan and question why they hit your kid and tell them they don't have a right. He would respond by saying, "if you don't like it, then don't send your kid to school here." For example, my boys here. One was in 6th grade and the other one was in 7th grade in Jordan. Can you believe that one day my older son was talking to his cousin in class and the teacher came and he slapped him right here? He felt pain and a bruise for over four days.

Riham: Oh no, and you couldn't say anything?

Participant: What are you going to say? You have no right to say anything. I let them go for two months then I stopped them from going. They had the boys between grades 7-11 in one place. You know how boys can be. They separated the Jordanian boys from the Syrian boys. And the Syrian boys will be waiting outside with— you know…

Riham: Yeah they are boys.

Participant: Problems arise between them.

Riham: They might fight.

Participant: They would bully them and fights would break out. No one would be with them either. The Jordanians would mock the Syrians and say “Why are you in our country? Why are you coming here?” ….We decided since we are going to America we don't want this type of education. Why would one go through all that for some education? To be emotionally abused for this education? No. For a whole year, they did not go to school. I took them out.

Riham: That's sad. Do they face any bullying here?

Participant: No, not at all. It's excellent here. The teachers are also excellent. Excellent. This daughter right here, during her last day in kindergarten, you should see how much she cried. She would go to the same school as the girls, but it's called kindergarten. You should see how much she cried because the school was going to end for the year! We don't have teachers that hug and treat the students kindly like this in our country. In our countries, the teachers think since they are teachers they don't need to show any compassion to the students. They would not use words of endearment. Here they are different though. They hug the kids and make them love school. [Translator’s note: hug implies putting arms around someone, sometimes figuratively, as in supporting the child]

Riham: Yeah, of course. There needs to be a certain type of treatment.

Participant: Yeah. They cause them to love school. Last summer the kids had to attend summer school by bus. My husband did not allow them because he knew what the busses were like. He used to go to Catholic Charities for the lessons by bus. He would leave in the morning at 8 am and come home at 2 pm. Since he used to take the bus, he saw all kinds of things on there. He wasn't willing to lose his children over a bus ride. The vice principle lives in our neighborhood. She said she was willing to pick them up and bring them back home.

Riham: That's really nice.

Participant: She spent a month taking them and bringing them back home. A whole month for summer school. They are really kind. We were even going to change schools because there are some students at this school that aren't good. After there was an increase in Syrian refugees here and they started attending the same school, they were able to speak in Arabic with one another. The teachers started complaining.

Riham: Why?

Participant: It hinders their learning when they speak in Arabic. They shouldn't. It keeps them from progressing in English.

Riham: Oh, so they can learn English.

Participant: Yeah. Now, thank God, their English is excellent. It's not perfect but it's excellent. They told us that Hasan and Hussein were doing much better in English before the other Syrian students came. They started to converse with them by phone and at school so they aren't as good as before. For example, my oldest son had all A's at first. After the other Syrian students came he started getting B's. The teacher told us. This one is still an A student, praise be to God. The teacher explained that Hasan started to speak in Arabic a lot with the other Syrian students…and the cell phones they’re playing on. They said it's prohibited at school but as soon as they master the English language they can speak as much Arabic as they want. They are in a class specifically to learn the language.

Riham: The ESL class most likely.

Participant: Yeah they bring all the refugees together there.

Riham: Yeah.

Participant: The Albanians, the Mexicans, the Syrians --.

Riham: Which school is this?

Participant's Child: Withrow

Riham: Withrow, okay. It's a big school.

Participant: Yeah it is really big. Then we wanted to change schools because they had some bad students. They told us we weren't going to find another school that has ESL classes. Because this class teaches students that don't know English.

Riham: Yeah.

Participant: Withrow is the only one here in Cincinnati that has this ESL class.

Riham: Right.

Participant: We said that's fine. Hopefully, later we can put them in a different school after they learn English.

Riham: Yeah, God willing.

Ahmad: God willing. Let's continue our conversation. You were saying that when you were in Jordan you didn't have any health problems. You also did not come to America because of health problems.

Participant: Right.

Ahmad: I don't know if you if know this but many refugees are coming here due to medical problems.

Participant: I know. Especially now, they are bringing everyone that has medical problems.

Ahmad: Okay. Yeah, they are bringing them because the Cincinnati Children's hospital is here. How much did you interact with the hospitals and doctors here? Other than the vaccines.

Participant: Every 15 days or so I go for my daughter due to her speech problems. Shes not able to speak well. I also go to follow up on my sons head problems as well as the vaccines of course. I have Anemia and Diabetes. I used to go every month but now I was told to come back after three months to redo the tests to see if there is any improvement.

Ahmad: Okay, and the transportation is all taking care of by that group you told us about, right?

Participant: That's correct. They make our appointments and drive us to the appointment and everything.

Ahmad: When you are dealing with the doctors here, are you finding any issues with the communication? Trouble understanding what they are trying to tell you, for example.

Participant: No. Let me see. There is one eye doctor that I went to. The insurance covers one visit per year. When I was in Jordan they told me I needed to have eye surgery. When I came here I brought with me the results and what not. The first year of being here I went to my appointment and told them what the doctors had told me. He said he will prescribe for me some new glasses and that I should wear them for a year. Next year he will test me again and see if I need a lens implant surgery [Cataract?].

Ahmad: Okay.

Participant: This year they changed our insurance. My doctor from last year did not accept our new insurance. So I went to a new doctor. When I got there he tells me that the interpreter canceled. The interpreter wasn't going to come. He said this counts as your visit because I am only allowed one a year. I started to call some of my friends from here. No one answered. Usually when the translator cancels they would call someone to do it over the phone. This particular clinic, the eye clinic, they told me they didn't have that service. He didn't even have a phone at the clinic. I called five different people and no one answered. I tried to tell him what the doctor said from last year. He said he will prescribe for me some new glasses. I was able to understand that part. However, when it came to details of the eye and the devices used from last year I didn't understand it. The google translate on the phone sometimes gives you the wrong translation. I tried to tell him and he said he didn't understand anything. I missed out on that appointment. All he did was prescribe me some glasses and say he will see me later. The insurance didn't even cover those glasses. Apparently, only one is covered every two years. Since I got one last year I would have to wait till next year to get a new one. This was a huge problem for me.

Ahmad: Okay. Did you have any other incident like this in which the interpreter didn't show up?

Participant: Yeah, it happens but then there are phones used as back up.

Ahmad: Okay, I see.

Participant: They would call and get an interpreter. This doctor just didn't have one.

Riham: You told us about your son that had a problem with his head. Do any of your other children have medical problems?

Participant: No, thank God.

Riham: Thank God. You mentioned that you only went to the hospital once, is that right?

Participant: Which hospital?

Riham: Children's Hospital.

Participant: No, I go to it frequently. All my kids go there.

Riham: Oh okay but as for the emergency room, it was only once.

Participant: Yes, just once.

Riham: Okay, then tell me about when you went to the emergency room. Do you remember what happened to you?

Participant: Honestly, when I went is was with my husband. At that time my husband because my son had an allergy.

Riham: Which son?

Participant: Yusuf. He had an allergic reaction on his chest.

Riham: Oh was it asthma?

Participant: They called it an allergic reaction on the chest, not asthma. Like a light asthma.

Riham: Yeah.

Participant: It happened to him in Jordan as well. He would get a high fever and his chest .. and he would turn blue when he coughs.

Riham: Oh his color changes.

Participant: Yeah he would turn blue in this area. We took him to the doctor and she gave him some medicine. We then brought him back home but he didn't get better. His fever came back and was really high so that's when we went to the hospital. When we went there to the hospital, we got lost. We walked and walked and walked. We didn't know where to go.

Riham: It's really big.

Participant: It was very big and we didn't know where to go. The kids were home alone and we didn't know where to go in the hospital. We came back and called someone from the group of women. One of the women then took me and we went around 4 pm and didn't come back home until 9 pm. We told her we needed to go to the hospital. She's from Cincinnati but I don't know her name. I used to go take classes in the organization. My husband used to go as well. That day he was sick. We took him to the doctor. I put three of my kids at the daycare that is close by. They told me if I put my kids there I need to either be studying or working. I told them I wanted to learn the language first before I consider working. While my husband and I were in the class we got a call from the daycare. They called Catholic Charities and told them that my young son had a high fever. He wasn't eating or drinking. Al they did was tell my husband here is the address. They said here is the address, take it. They didn't even offer to take us. Imagine, we have never been there, and we were worried.

Riham: At least to show you where to go.

Participant: Catholic Charities isn't a good organization at all. My husband and I went and got lost. We went all over and even forgot where we parked the car. We left home at 11 am and came back at around 3 pm. We didn't even accomplish anything during that time. Then one of the women from the group she asked the others if anyone was available to come. One of them came and she was around 70 years old. She has 7 children. Her youngest child was my age. She came and took me and my son. She warned us that it was going to be a while. We left at 4 pm and got back home at 9 pm. We stopped by and picked up the medicine he needed to take. They gave him a shot and said he had an ear infection**.** They then flushed out his ears [irrigated his ears]. I didn't really agree with this procedure. I felt like the problem was in his chest. In Jordan when it happened they ended up giving him a shot and then he got better. Here they said it's so infected that they wanted to irrigate it. I didn't want them to do that. The problem here is that if you tell them you don't want to do something they tell you that it will be your responsibility if anything happens. If you don’t agree, you sign paperwork saying it is your responsibility.

Riham: Can you please repeat this last point? I didn’t understand.

Participant: They said the young --.

Riham: The doctor told you that there was an ear infection --.

Participant: There was an interpreter there.

Riham: Okay. Then what happened?

Participant: She said there was an ear infection and that they wanted to irrigate it.

Riham: Okay so that they can remove the wax.

Participant: Yes, the wax. If I didn't agree to it then I would have to sign that I would take responsibility for it.

Riham: Did they make her sign AMA? Is that what she's saying?

Ahmad: I don't know if it's AMA but it would be her responsibility they said.

Riham: I have never heard. Were you about to leave the hospital at the time?

Participant: I didn't even say anything. She just explained it all to me. She said that if I don't permit them to irrigate the ear then it would be my responsibility, not theirs.

Riham: Yeah

Participant: Because the child was burning up and they gave him two shots, irrigated his ears, and prescribed him some medicine. They gave us a small device to put at home --.

Riham: It sounds like bronchiolitis. Were you at Children's?

Participant: Yeah.

Riham: In the Emergency Room?

Participant: They took us to several rooms. They first took us to a room where we waited for a bit. They took measurements. And then they took us to another room --.

Riham: It's most likely triage. Okay, I understood what you were saying. Since this was the first time you've been to an emergency room in American, tell me a little bit about your experience.

Participant: My experience when we went in was excellent but when we were outside it wasn't good. Please don't mind me saying this but in our country when we call an ambulance for someone, that person is able to see a doctor right away! If I call an ambulance, an emergency, I don't want to sit and wait five hours while my son is burning up. No offense but this fever isn't a light matter. It's frightening. I went thinking it was an emergency room. That people were going to be there to ready to help when I arrive. Not to sit and wait as if there was nothing wrong or no fever. God forbid if the fever of a child gets too high – it hits the brain!

Ahmad: Of course. it's frightening.

Participant: It's not good here. It's really not good. Since you have this high level of care for children, if you want to make it better you then you don't leave a child waiting long in the emergency room. If it wasn't an emergency no one would bring their child there.

Riham: So you spent more time waiting than you are used to.

Participant: A lot of time. Even in Syria, I mean in Jordan they opened an Emirates hospital. It was free for the Syrians. There was an objection at the time that the Syrians were getting treated differently. They had an emergency room. In Jordan, when the same thing happened to my son I immediately took him to the emergency room. They call you in immediately and the doctor comes right away. The doctor saw him and checked his temperature. There are people there waiting specifically for urgent cases and are ready to help. It is called emergency afterhours. Here? since I am forced to come, I shouldn't be made to wait 5 or 6 hours just to call me in. Even after they called me in they examined him and then said the doctor will be here soon. I didn't like that at all. I went for the first time and hopefully for the last time. I hope I never go again. I didn’t like the experience. This thing is called EMERGENCY. It is not just 9-1-1 ambulance. It needs to be a doctor and a nurse waiting to take care of each child. The fever it can hit the brain. So many kids, they were injured by high fevers!

Riham: It is a different system here than back home.

Participant: What is that? A fever can really affect the head. A lot of children developed problems from having high fevers. There was this one lady that I know. When she came she refused to stay. She's a gynecologist here. She went up to the receptionist and told them that her daughter had a fever. When they told her she had to wait she refused and ended up taking her daughter and leaving. I didn't like this at all.

Ahmed: Yeah.

Participant: If a person calls 911, they say it is not an emergency. This case is not an emergency.

Riham: What, in your opinion, comprises an emergency?

Participant: I saw a woman sitting holding a bandage over her sons bleeding head. His blood will dry before he is even seen. This, this scares me. A Fever. That is a very urgent matter. It hurts the entire body, the brain. It really hurts children. And a cough. If it his face turns blue, under his eyes are blue, that is urgent. I am not a doctor, please excuse me. But I know when a face turns blue, the lungs are failing. They are not able to breathe well. Something is wrong. They are not getting air. The under eyes become more blue. I am brining my son in an emergent state, and why should I wait for 4-5 hours? I would have left at home!

Riham: Yes.

Participant: I had my daughter here. I picked her up from school. She had fevers, chills. She was hallucinating. I called one of my friends on the group. My friend called the clinic, the doctor, and told me “this is crazy!” I can’t believe the doctor tells you that you need an appointment. The child is sick. This is crazy. To have an appointment.

Riham: And where do your children go for primary care?

Participant: We used to go to the one…far from here. Where the refugees go. But now we go to health group. On Montgomery rd. We got vaccinations there. The first time we went there was at the beginning of this year. Tomorrow we're going back for more vaccines.

Riham: Okay.

Participant: This will be the second time we go.

Riham: Okay, that's great. Okay, did you like the doctors there?

Participant: There is one doctor and she is excellent.

Riham: Oh wonderful.

Participant: They had 4 vaccinations at a time. It was hard on them. So she told us to return again in 6 months. So now we have an appointment tomorrow. We will continue the vaccines.
 Thank God. Tomorrow we will be going at 1 pm God willing.

Riham: That's really good.

Ahmed: A bit ago, we were discussing mental health, and how these experiences have affected you all. Many refugees have discussed feelings they have had, some depression and anxiety, would you be able to speak about your experiences and how you dealt with your emotions?

Participant: Yeah we went through very hard times. And even my husband…Actually. We can talk but please stop recording. It's not personal but please don't record this part.

Riham: Of course. We will turn it off.

Interview 7A

Time (7A.I) – 0:00

Ahmed: Today is July 17, 2017. I'm Ahmed Beydoun and have Dr. Alwan here with me. We're at a Syrian family's that has six kids here in Cincinnati. Okay. So, Abu Mu'adh, let's start from the beginning. When did you come to Cincinnati?

Participant: We, God willing, were here on December 14, 2016.

Ahmed: 2016. And you came—. Where did you come to Cincinnati from?

Participant: We came from Egypt. We spent a night in New York, and from New York we came directly here.

Ahmed: Okay. Could you tell me why you came to Cincinnati?

Participant: Honestly, we came here to Cincinnati—. Of course, we came and presented my son's file. He's sick. Because I was struggling in Egypt because of the medical treatment.

Ahmed: Okay.

Participant: And I presented his file to the UN, that “I want to medically treat my son and travel.” And they [chose for me/moved me] here to Cincinnati because of the children's hospital. They told me “It specializes in your son's condition.” And that's the reason why.

Ahmed: Okay, so you came here to the United States to Cincinnati to treat your son.

Participant: Yes, that's right.

Ahmed: Could you tell us more about your son's condition and the circumstances that led to your coming here?

Participant: Honestly, I was struggling with my son—. We struggling with his sickness in Egypt because he was getting a fever. They told me in the end that they gave up on his sickness. They told me “Your son's disease is the cause of this. It causes a fever. It causes—.” But there was, for example—. Now I know his disease has to do with the brain and nervous system [another way of saying “neurology”]. I know this disease, but the problem is that he gets tonsil infections [tonsillitis]. He gets stomach infections [gastritis]. He gets urinary tract infections [cystitis]. Those things are treated alone. So, over there, they weren't treating him. I wanted to come here to see about these things.

Ahmed: Okay.

Riham: I'm going to ask you a question. I know the situation, but so I can remember it when reading the text—. Tell me a little about what your son's disease is. What do you know about your son's disease?

Participant: My son's disease—. In Egypt they described it to me as a wearing away of the white tissue. It's a wearing away in the white liquid—. The white tissue is the white liquid that's in the brain. The reason for it, they said is that he gains his strengths/abilities to a certain age then goes back and loses them. In fact, my son really was like that. My son started walking. He started talking. He started playing. Then little by little he started to regress. Little by little. Yea, so he lost his hands. His lost his mouth's movement. He lost his legs' movement. He lost the ability to speak. Little by little, as they told me, his abilities started to decrease little by little.

Riham: When—? How old was he when you first felt that he changed, that there was a problem?

Participant: My son, from when he was little, used to have convulsions [“tashannuj”]. Like in the first six months, he had the first convulsion. Later, after a year or 10 months, he got a second convulsion. Then they started to increase. He would convulse then return to normal. It was normal with nothing wrong.

Riham: Does “tashannuj” mean a seizure or paralysis?

Participant: “Tashannuj” is—

Ahmed: What do you mean by “tashannuj?”

Participant: May God's peace and blessings be upon our chief, Muhammad—what was “tashannuj?”

Riham: You mean like this?

Ahmed: Okay, okay.

Participant: Now, “tashannuj” means he does like this then goes unconscious.

Riham: Then he goes unconscious.

Ahmed: So seizures. Okay.

Riham: Okay, so a seizure.

Participant: Yea, and—.

Riham: Okay. Did they used to give him medication for this?

Participant: They used to give him medication, but they explained his disease to me in Syria—may God reform them and guide them—. I mean, they didn't discover his disease. They said it was just a surplus of electricity [This term is used as a euphemism in the Arab world meaning “epilepsy”]. That's it. They said, for example, his tonsils are infected. He's getting a fever. He's getting—.

Riham: Because every time he would have convulsions he'd have—.

Participant: That's what would happen.

Riham: Yea.

Participant: Like, we—. I mean, we were lost. We didn't know. Because sometimes his disease would cause the fever.

Riham: Yea.

Participant: And sometimes because of the fever his sickness would increase. So, he used to—. I mean, I would take him to the doctor, and they would tell me, “He does not have a fully developed immune system. Like, when he reaches seven years old, his immune system will be fully developed, and this thing will go away, God willing.” That's how—. I mean, they were hoping that it was a surplus of electricity that was causing him to have convulsions. Yea, but it turns out he has a brain disease. We didn't discover it in Syria. I mean, nobody discovered it. Until the end of the—. When we left, he started—. Like I told you, the first year he had convulsions. Then he got more. Then every six months. Then it started to increase. But when he used to have convulsions he'd go back to normal. There was nothing wrong. So before we left Syria, his convulsions started to increase. So—glory be to God—by chance, I have a friend whose kid is like that. He said, “No, you have to take him to a neurologist.” I took him to a neurologist. She said, “What you've described about your son means he must have a brain disease. We have to take images [this could mean an X-ray, MRI, CT scan, etc.] of him.” But—glory be to God—we were going to travel to Saudi Arabia after a few days, so we didn't get a chance to make it. We didn't take any images. In Saudi Arabia they took images of him, and they did brain imaging/brain mapping [not sure what is meant here]. It seems that they didn't discover anything in Saudi Arabia either. They told me, “There's nothing.” But the kid's condition was getting worse and worse. The convulsions were increasing. So they started in Saudi Arabia—. I mean, I wasn't in Saudi Arabia. I was in Syria, and they were in Saudi Arabia. He started to walk and fall. I would be speaking to them by phone and find that his speech was regressing. So I was asking his mom, and she told me, “He's getting more and more convulsions. He will be walking, and then suddenly his leg will give way, and he'll fall down.”

Riham: How sad.

Participant: He starting not being able to control his leg. He started little by little. So in those six months, they got a little more. He was really attached me and got affected by my absence. I mean that made them increase even more. When we came to Egypt—. At that point I had been away from him for six months. When I saw him after six months, it was like really the boy is totally different.

Riham: Within those six months.

Participant: And right when I saw him I knew that there was something. I mean, I immediately took him to a neurologist, and he ordered an MRI. It turned out from the images that he has a wearing away of the white cortex. We did [another/an magnetic] image. He said it's in the white cortex, but they said he can be treated little by little. It also seems that they didn't know what disease he had. From that time we started giving him “debekine” and “tretam,” and they used to increase it and increase it. There was a doctor who started increased the “tretam” and the “debekine” and—.

Riham: Sorry, what is “debikan?”

Participant: “Debekine.”

Riham: Oh, Depakene. Okay.

Participant: And “trepital.” Here they call it “trepital.”

Riham: Oh, Trileptal.

Participant: Trileptal.

Riham: Okay, those are medicines for convulsions.

Participant: For convulsions, yea. He said, “Your son has to take them and not stop.” His convulsions started to decreases. He started to give me a program, telling me “Come back after a month.”

Riham: Yea.

Participant: He would say how much—. He would tell me—. I would tell him, for example, “They're decreasing.” And the doctor completely understands the situation to the level that in the latest period he would tell me, “Your son doesn't have any convulsions anymore” or “They're very few.” And it happened just like he said. When he increased his Trileptal to 4 cm, his convulsions decreased completely. But the problem is that we used to suffer. He would get a fever. When he had a fever, his condition used to increase. I mean, he used to go back to normal, but every time he used to—. I mean above—. I mean, he's taking the medication, and he's not getting any convulsions, but he's losing—. Like, for example, suddenly I'll find that he's not able to move his hand. “Honey, hold this.” He's not able to. “Hold this.” He was no longer able to walk. He started to crawl. So these progressions continued. They didn't stop. The convulsions stopped, but the progressions in the reverse continued until we reached a point where the boy [missed/lost] everything. Then one time, we had a lot of heat in Egypt, and he got an infection in his throat or something else. Honestly, we didn't know. His temperature went up to 40. It was more than 40. He had a really strong convulsion and lost consciousness. Yea, so I took him to the hospital. I mean, he lost consciousness. He spent a while, a few hours, then—. Yea, then—. It was the second time then. Then another time it happened, and he lost consciousness for days, ten days. He lost consciousness for about ten days. I mean he was in a coma. I took him to a hospital in Dimirdash in Cairo, and he spent about ten days there. And when they discharged him, they said, “Your son is going to stay like this. Like, that's it. He's going to remain unconscious.” When we left, about the time when we were on the way or the first day we arrived, right when I took him to my brother's place, the boy woke up. He woke up by himself. They said, “He's going to remain like this,” but he woke up by himself. Yea, but I started to really struggle because of—. One time he had a—. So, over there they studied his condition and said “Your son is going to always stay like this. Like, his disease causes him to lose consciousness. It's what causes his temperature to go up and down.” The thing I suffered from the most was when my son had urinary retention [ischuria]. He was unconscious and wasn't able to feel too much. So I took him to the hospital. They inserted a catheter and relaxed the boy and everything. He stayed there for about—. This was a children's hospital in Mansurah, after it. Yea, so they inserted a catheter, relaxed him and everything, and after four, five days they said “We need to discharge your son from the hospital.” I told them—. And his temperature was approximately 39 or 40. I told them, “How are we going to discharge him and his temperature is like that?” They said, “That's how your son's disease is. The temperature nerve or the temperature storage”—or I'm not sure what they said—“failed because of his disease. That's the reason why. Your son's temperature will go up and down by itself. Like, there's no stabilization.” I mean, the ability to control the body's temperature and all that has failed in his body. I told them, “But you told me there's a urinary tract infection, and you inserted a catheter. Doesn't he need shots?” They said, “Yea, here take some syringes, but give them to him at home. Try them out. If this is the cause, his temperature will go down. If it's not the reason,

Time (7A.I) – 10:00

Participant: your son is always going to be this way.” I took him home. Of course, we couldn't stop using the cold wet cloths. The fan was facing him, and I brought him the medication. I wanted to treat him myself. We gave him the medication for a week, and really it turned out that the cause was these medications—. Its cause [the fever] was the urinary tract infection [cystitis]. And thanks to God's favor upon us, he was healed. He was healed, his fever went down, and his urinary tract infection was healed. He went back to normal. Then he spent about a year like any other kid. Just like any other kid, he would get a fever and is aware, but his condition was increasing and increasing. But this thing, like, you're discharging him from the hospital and telling me that he's going to stay like this? Like, you're discharging him, and he has a temperature of 39? That's something I really suffered from in Egypt. That's the reason why I started to think about traveling, taking him abroad and getting him medical treatment. We know that there's more advanced medical treatment in western countries than our countries. Honestly, that's the reason why I started to think about coming here. And when we came here, I was surprised when they told me that this disease doesn't have a medicine, that he has a new disease, which a doctor discovered 15 years ago in the Netherlands. And the disease—. They told me my son is one of 100 in the world with it.

Riham: Oh, wow.

Participant: Yea, “Your son,” they told me—. Because there's a doctor in the Netherlands researching it—.

Riham: Does anybody know about this disease in America?

Participant: In America, I don't know, but they said—.

Riham: I mean, are there other children like him?

Participant: I don't know if there are others like him, but she said—. The doctor here said “We're in touch with the doctor, and we'd also like to get some information from you about your son for him to research the issue.” Because I requested from them—. I told them that—. I told them, “We're not—. “We believe that ''There is no disease except that it has a cure. The one who knows it knows it, and the one ignorant of it is ignorant of it.'” That's one of the sayings of the Messenger, may God's peace and blessings be him.

Riham: May God's peace and blessings be him.

Ahmed: May God's peace and blessings be him.

Participant: So I told that to her, to the doctor. She said, “What you're saying is right. We're researching the issue.” I also told her, “What's a proof of this saying is that there are a lot of diseases that you said 'don't have a cure when they were first discovered, but later they found a medicine for it, and people were treated.” So I told her, “I hope you would research the issue.” I mean, I told her, “You might not find it in my son's lifetime, but for other kids—.”

Riham: Yea, good.

Participant: “you have to research this issue.” So the doctor got really happy and said—. Later she told me, “We—. This is a new disease, and there's a doctor that discovered it in the Netherlands.” And she's the one who told me the story after I told her this. Sshe told me, “We're doing a research study with him. Llike, he's researching and wants to know what information there is about your son and about all the kids. He wants to see how the disease is progressing so they can find a medicine for it or—.”

Riham: Yea. Okay, but that means it's good that this doctor—. Is this the neurologist you're talking about?

Participant: The neurologist.

Riham: What do you think—? I mean, when I first met you, you all might have just arrived.

Participant: Yes.

Riham: You had come to the ER.

Participant: Yes.

Riham: And at that time, maybe you had [come to/been admitted to] Children's Hospital, right?

Participant: Yes.

Riham: When you arrived in the country, what did you think? Tell me a little bit about your experience when you arrived in the country and how you felt.

Participant: Honestly, when I came here to this country, frankly—. We have someone responsible for us called a “case worker.” He's responsible for us. So, I told him—. He even saw that I have a sick kid with me. They even sent a escort with him on the plane from Egypt to here, someone specially assigned to my son because of his condition. So I told him, “We want to take my son to the hospital as soon as possible.” He told me, “Now, as long as his condition is good, we're moving your paperwork along until the social security and whatnot comes out.” He said, “Unless there's an emergency situation.” Then a week or ten days after we arrived here, it happened that he had a temperature and little bit of urinary retention [ischuria], and my son had a high temperature. When I took him to the ER was the first time I saw you. Honestly, when I entered the hospital, they received us well, and I was surprised, thank God. I was worried about the language and worried about things. An interpreter came, and the situation was good, thank God. They took a look at him. And I was happy that they started with him from zero. I mean, they started with tests and started with—. They can know what—. They even took new images and everything. They asked me, and I told them, “I requested something, and they were doing something else by themselves.” I told them, “I want to know more about my son's disease, and I want to know what measures are taken.” I expected to find him a medicine here, but I was surprised that they told me, “Unfortunately, honestly, it doesn't have a medicine. The problem is it's a genetic disease.” And my son inherited a gene that—. They said—glory be to God—every child of Adam has two genes, which are

Riham: Copies—glory be to God—yes.

Participant: copies, of course. This gene that's responsible for the wearing away of the white liquid—. He took two genes, one from me and one from my wife, and both are afflicted with this disease. His white liquid is not increasing, and the white liquid is responsible for movement and is responsible for everything.

Riham: I see what you mean.

Participant: That's the reason for it. Even—.

Riham: That means it's hereditary.

Participant: It's hereditary. That's what they discovered.

Riham: Yea.

Participant: They even got worried about the other kids. We did tests for all the kids because they said—. Even in Egypt they told me, “Two and a half out of 10 of your kids are going to be this way.”

Riham: Two and a half out of 10? Wow.

Participant: Yea, and they saw my kids and said, “We have to do it for them.” Frankly, I was really nervous—. There was a time for two months when I was nervously waiting to hear the results before they called me—. I mean, it was the best phone call I've ever received in my life, and it was the most beautiful moment since coming here.

Riham: Thank God.

Ahmed: Thank God.

Participant: They said, thank God, the kids don't have anything. I have a son called Abdur-Rahman who has one of the genes.

Riham: Okay.

Participant: But they said he won't be affected at all. I mean—.

Riham: God willing.

Participant: We say that he's carrying the disease, but he's not affected because one gene does the thing [is dominant?]. The other gene is just in case [recessive?]. Like what—.

Riham: Thank God.

Ahmed: Thank God.

Participant: Thank God. Like, I calculated it out of 10. We have six. In fact, we really do have like one and a half right now approximately.

Riham: Yea, that's right.

Participant: I mean, their results in Egypt were on point just like they told me, and here it was on point again.

Riham: Yea.

Participant: It turned out that I have a kid with two full genes, and one kid with a gene. I was happy because I found the information to be in exact agreement. Yea, and the information was correct about these things. Yea, but, thank God, they told me, “Your son won't suffer with—. Your other son won't be affected with anything.” Yea, so I was really happy with the tests and these things. Like, a person gets relaxed when they see people—. That's the reason.

Riham: Okay. Thank God. Did you find any problems when speaking with doctors or understanding these things? Because it's clear that these aren't easy issues to understand.

Participant: No, honestly, I didn't find any problems. Didn't I tell you we sat for a time and—. I mean, I sat for a time—. One time I was asking a question, and the doctor said—. This is when the specialist came. I mean, I was asking her a question like “What's my son's condition? Explain it to me.” So she said, “That's a bit of a difficult question for me. Let me call the primary care physician.” Then when she called her, she explained to me what the white liquid is, what his disease is, and—.

Riham: With an interpreter.

Participant: Of course, with an interpreter. No, I mean, with regards to the interpreter, they really put us at ease. They bring one for us a lot.

Riham: So your experience with the interpreters—.

Participant: No, thank God, they're good. They're all good. Anytime there's a situation, they bring an interpreter for us.

Ahmed: Did you face any problems with them?

Participant: No, thank God, with regards to me, I didn't face any problems with them. Whatever I say they translate, and everything was appropriate. Because I spent 10 days with my son in the hospital.

Riham: Oh, ten days.

Participant: Yea, we spent ten days in the children's hospital. I started to see interpreters on a daily basis and see doctors everyday in the morning. I used to see them just about everyday. Yea, so I started to have a lot of experience with his disease and I saw all the circumstances.

Riham: Thank God.

Participant: Yea, thank God. And until now they said it doesn't have a disease [perhaps he misspoke and meant “cure”], but we believed in the Master of all creation. It's possible at any moment for the Master of all creation to cure him.

Riham: God is great.

Participant: His organization is such that—. “After they have decayed.” [A reference to 36:77-83 in the Qur'an] We're seeing him and counting on, God willing, God to cure him.

Riham: Amen.

Ahmed: God willing.

Riham: So tell me about your other children. How has your experience been?

Ahmed: How has your experience been with the health care system?

Participant: Honestly, thank God, no, the other kids are normal. There are no problems. They're like any other kids. They go to get their vaccinations. They're going to—. I mean, there's no struggles, frankly. For me, there are no struggles with anything, thank God.

Riham: Thank God.

Ahmed: Thank God.

Participant: We're treating them, and everything is good with them, thank God.

Ahmed: Thank God.

Riham: Did they start school immediately when they arrived?

Participant: No, honestly, by chance, when we came—. Just like I was telling you, December 14 was a break. I mean there were holidays. There was a delay until they were enrolled in school. It was another month and a half or a month until their were enrolled. The little ones didn't catch up a lot with the language [English]. I mean, Mu'az is the smartest of them by a little bit. They didn't catch up because they studied for three months or two and a half months then it happened to be a vacation, too. That's the reason why.

Ahmed: You mean they're not going to school now?

Participant: Now in the summertime there's no school. Because they told me, “You have to drop them off and pick them up.” It's hard for me. I mean, we're—. Frankly, we're—. The things we're struggling with—. I mean, here they told us that—. Here it became clear to us that the whole family has to work. They have to. And really, here for somebody to manage things, we found that the father works, the mother works, the children work. What we're used to in our country is that the father works, and that's it. It's enough for the whole family. The kids are focused on their studies, and the mother is in her home. In Egypt it was the same thing. Like, when I went to Egypt, I started working. So, here we found that the whole family has to work to—.

Time (7A.I) – 20:00

Participant: I mean, to live a normal life. But here we're—. Like, my wife can't work. My wife is a—.

Riham: A nurse.

Participant: A nurse. I have friends in other states. They told me the mother—. They should bring you someone to be with your child or the other solution is for the mother to stay with the child and they give the mother a salary because she's not able to work. Do you know what I mean? Now, the mom—. So I asked them about this here. They told me, “This service isn't available in Cincinnati.” I mean, the case worker that's in charge of her. Because now if my wife wants to go out and work it's hard for her work. She's with my son and is with two young kids. That's the one thing we're struggling with. It's like she has to always be with him, and we aren't able to leave my son in the hospital. We're used to always being with him. I mean it's not a problem. But to put him in a hospital so that she can go and work or whatnot—. No, we don't like that. I mean, we're not able to—.

Riham: Did they suggest you put him in a hospital? Is that what you're saying?

Participant: Honestly, one guy—the guy whose place I'm working at—put his son in a hospital.

Riham: You mean a nursing home?

Participant: Yea. They said that they'll put him there. I told him I'm not able to—.

Riham: Like a children's home/day nursery.

Participant: Like a children's home/day nursery where they take care of the kid.

Riham: Yea.

Participant: Yea, well if we sit here, we bring him down from his room. If we go upstairs we take him up. We don't like to—.

Ahmed: For him to stay alone.

Participant: I mean we're—. I mean we're—. Our idea is that the kid is living with us. The kid's thoughts are with us. Now, we know that he only hears. Like if we make a clamor, he gets startled. We know that he hears. That means that he hears us. He's sitting with us and hearing us. We can't leave him at all. Except if he falls asleep. I requested the device [baby monitor?] we had in Egypt. We put it here so that if he falls asleep upstairs I put a device for him so that if he cries, yells, throws up, or does anything we sense it.

Riham: Like a camera with sound?

Participant: We had one with sound.

Ahmed: [Indistinct]

Participant: The one they brought me here has a camera with sound.

Ahmed: Good.

Riham: That's really good.

Participant: Yea, so they put it for me with a camera and sound—may God reward them. They did really well in taking care of things. It really helps us relax because when we put it upstairs—. Because sometimes he sleeps and doesn't get up until noon, 12 PM or 1 PM. So we put the device for him and come downstairs. The mother—. We see—. When he wakes up we immediately bring him downstairs. We don't like to be away from him at all, Ahmad. In addition, we feel that he's living with us. When he's not with us, we feel—glory be to God—that someone is missing. Honestly—.

Riham: Glory be to God.

Ahmed: Glory be to God.

Participant: We're used to him. It's not something normal. We imagine that suddenly he got the disease, but he, for sure, says—. If we have to leave him it's a really difficult situation. We can't be without him. Like, I have to go to work, but my wife is always with him. Even when my wife and I have to go to the doctor like we have a dentist's appointment or something, we don't leave until Mu'az is home. Like, I've made Mu'az absent from school four or five times so he can sit with Ahmad. And when I enrolled Mu'az in school I told them, “My son is often absent. We have special circumstances.” That's why.

Riham: Did they help you with this? The school?

Participant: Yea—. Honestly, we don't like to make him absent a lot, but I told them, “We don't like it either, and my son doesn't like it, but there's a special circumstance that—. We have a boy. We have to have someone with him.” Even my wife—. If they were going to send us a nurse to our place, her mind wouldn't be at rest. Sometimes they tell us, “There are some nurses that come to homes and stay.” She doesn't like that. She likes to be with him.

Riham: Her mind is not at rest about what?

Participant: Like when they come and sit here, she likes to—. She feels like “I abandoned my son” a little bit. But, no, on the contrary, they're really doing us a great service, the nurses—.

Riham: It's a different perspective.

Participant: Yes.

Riham: Like, they see it like they're helping you, but you see it like you're abandoning your son.

Participant: They're helping us. They really are helping us, but in our view we've abandoned our son.

Riham: It's a different environment [culture?], of course. We have a different environment.

Participant: Even the boy—. For example, when we came here, his awareness was better. When he used to go to the hospital, his psychology would change.

Riham: Glory be to God.

Participant: I used to tell them in the hospital—. The nurses would see him—. I would tell them, “Look what happens when I tell him we're going home.” I used to talk to him and tell him, “We're going to see Mama now.” I would feel that the kid would become aware/enlivened a little. I would tell them, “We're going to see Mama and your siblings now.” I mean, he's used to sitting at home and hearing his siblings' voices while they play and fight. I mean, that's what he hears and whatnot, but when he's at the hospital, honestly his psychology changes—glory be to God.

Riham: Glory be to God.

Participant: Now his awareness is about—. It's a little poor, I mean. I mean, he's not sensing too much, not expressing his emotions too much. Again, it's because of his disease, too.

Riham: When someone has a chronically ill child, there's a lot of pressure on the heart, on the mind, and there are psychological pressures. Tell me what you do to take care of your psychology.

Participant: Honestly, I'm going to tell you something. Because of God's favor upon us, our belief in our Master is great, and we know that everything has been determined by God. We say, “This is what God has decreed, and He does whatever He wills.” We have believed in the Master of all creation, and, God willing, He will heal him. We know that this is a test from God. If we're content with this, we have rewards from the Master of all creation.

Riham: God willing.

Ahmed: God willing.

Participant: And the boy—. I mean, we say, “Glory be to God, why is he different from his siblings?” For example, his siblings are playing, and he's not. Of course, this is not for no reason. The Master of all creation knows more about his condition and is more knowledgeable about everything. We have a lot of faith in that, thank God. That's why we aren't bothered or get sad either. Sometimes, a person's feels like “Why is it like this? How did he used to be and how did he become?” However, immediately we say—. Like, in that very same moment, we say, “Thank God.”

Riham: Thank God.

Participant: This is God's will. We can't say anything about that. Rather, we say that we are satisfied with it because a person gets rewarded for their content [with God's will].

Riham: Of course.

Participant: There are people without hands. There are people without feet/legs. There are people—people from our own families that we know—who are walking in the street then end up with no feet/legs.

Riham: Of course.

Participant: We—. Here we found out about one of our relatives who a shell [mortar] fell in front of then was without feet/legs. She lost a foot/leg, and they're going to treat the second foot/leg. I mean, for these things, we say, “Thank God in every situation.” I mean, there's no problem.

Riham: So you find that your religion and faith strengthen you?

Participant: Yea, our religion and our faith are what put our minds at ease because we have a really difficult situation. I mean, we have a—. Especially Ahmad For example, Mu'az fights a little bit with his brother. The others fight with him a little. Of course, they're just siblings, and the little ones fight with each other everyday except for Ahmad. He used to like everybody, and everybody used to like him. He was a peacemaker.

Riham: Glory be to God.

Ahmed: Glory be to God.

Participant: We say, “Glory be to God. [Indistinct]” We say, “Glory be to God, this is God's will.” We don't get sad, thank God. That's how it is. But this is a difficult situation for us. How we used to be—. I mean, Ahmad was really, really beautiful. His beauty was really beautiful. Everyone in the family used to talk about his beauty. Yea, so, thank God, we're content with God's decree.

Riham: Thank God.

Ahmed: Thank God. Thank God. Can you tell us a little bit about the circumstances you faced here that aren't related to medicine? For example, how was your coming to Cincinnati? How did you find the city? How did you find your place [house or apartment]? All those things.

Participant: Honestly, the problem is that—this is from God's favor—I honestly didn't face any problems, thank God.

Riham: Thank God.

Ahmed: Thank God.

Participant: I came here—. I mean, the people who came before me faced problems. They didn't know anybody. They didn't—. Yea, they told us the story of how some people [Indistinct]. So, we came here—. They give us a “welcome.” I don't know if you know the story or anything.

Riham: The “welcome” is the sum of money.

Participant: The sum of money. So the caseworker is responsible for it in the beginning. We came to this place here you see immediately. He had rented it out for us and put us a little bit of furniture, but when we arrived in the airport the people who welcomed us were some of the most kindhearted people. One of them was Abu al-Bara' 'Abd as-Salam Trabulsi. One of them was Ashraf Tarabulsi, Abu 'Abd ar-Rahman.

Riham: Yea, he's really respectable.

Participant: Until now I don't know—. I treat them like my brothers, and they treat me like a brother, thank God.

Ahmed: Thank God.

Participant: They came there, and it was like my own brothers were waiting for me [in the airport]. We came here—. “What are you missing?” Everyday they used to come here—. Imagine, the first week—. They had brought us everything, food, drink, and everything, both they did and the organization, may God reward them.

Ahmed: May God reward them.

Riham: May God reward them.

Participant: Yea, but sometimes I'd be in need of bread. I'd be in need of—. My son or daughter would want milk. That was a problem I had. As much as they'd deal with me like brothers, I'd find it a little bit difficult [literally, “heavy”] [to tell them of my need]. As you see, my place is a little far form the malls, is far from—. I mean, my son tried to go to Kroger alone—the closest place—and it took an hour.

Riham: An hour of walking?

Participant: About an hour of walking. When we came it was cold. There was snow, too.

Riham: Of course!

Ahmed: [Indistinct]

Participant: So that's how we struggled, but on top of that, thank God, honestly I'd give them a call, and they'd come to me at 10 PM or 11 PM. They'd bring me bread. They'd bring me—. So, these days I tell Abu al-Bara'—. I tell 'Abd as-Salam. I tell him—. I tell him, “How is it that I used to tell you, 'I'm in need of some bread,' and you used to bring it to me and there's about 25 minutes between our places?” He used to tell me—. May God reward them.

Riham: That's how brothers are.

Participant: Yea, honestly. Honestly, they're like brothers, and they're people

Time (7A.I) – 30:00

Participant: that helped us a lot [literally “stood with us”], thank God.

Riham: Thank God.

Ahmed: [Indistinct]

Participant: And they helped us, may God reward them, to—. I, thank God, have been driving since Syria and used to have a car.

Riham: Oh, that's good. Okay.

Ahmed: Okay.

Participant: So they helped me [whem I only had?] one or two lessons to get to know the streets because, you know, it's a new city and—.

Ahmed: With a car.

Participant: Yea, and they helped me get a driver license. They also, may God reward them, got us a car.

Ahmed: May God reward them.

Participant: Yea, and I became—. All of my matters became good. I started to go and come. Within two months I had a car. I mean, I had a car and a driver license.

Ahmed: That's good.

Participant: Yea, thank God. I mean, our situation was good. Yea, thank God. The only problem we're struggling with now is the language. God willing, we'll maybe—.

Ahmed: [Indistinct]

Riham: God willing.

Participant: Because it's really necessary, and I'm determined [to learn] it, thank God. God willing, I'm learning. I mean, I'm better than my friends by a lot. Like they came five months before me and six months before me, and, thank God, I speak better than them.

Riham: Thank God.

Ahmed: Thank God. Good for you.

Participant: God willing, I'm going to persist in learning, God willing. I'm trying to—.

Riham: May God provide you with relief.

Participant: Amen, for all of us.

Riham: So what I'm hearing is that the community and your religion are the things that you find give you strength and give you the possibility to live happy, right?

Participant: Yea, yes.

Riham: Is there anything in your view that helps refugees when they arrive, that helps refugees when they're bothered by difficult situations [literally “have a shortness of breath”], when they find depression?

Participant: Honestly, let me tell you something. When a person—. The most important thing—. The most important thing is that a person is not wronged.

Riham: Is not wronged.

Participant: When a person gets wronged—. When there's injustice, no matter who it's from, a person feels bothered [literally “has shortness of breath”) and that the whole world is against them. Once they don't have any injustice in their matters—. Once their matters are the same/equal, just like others, for example, you'll find that they live in happiness. I mean that there are no problems. And especially the first moments when they arrive and have someone to assist them like I told you how the community came to me and assisted me a lot. And, may God reward him, I really thank my case worker a lot. He was on point about his appointments and on point about everything. It was to a level where he told me, “You're my friend.” He goes—. Until now he visits me every once in a while.

Ahmed: [Indistinct]

Participant: Like, “I like to come over and check up on you.”

Riham: He's from Catholic Charities?

Participant: Yea, Catholic Charities. Yea, there are some of my friends, who are really struggling with their case workers. Yea, but as for me, I wanted to mention him [my case worker] because he's a man that does a lot of good, may God reward him—.

Ahmed: May God reward him.

Participant: regarding the appointments and everything and what I have with doctors and what I have—. He is—. He is great and a hard worker. So he also really made me feel relaxed. Like, I heard—.

Riham: Like he didn't let you miss appointments. He didn't let you forget—.

Participant: No, he didn't let me. He didn't let me.

Riham: And transportation—.

Participant: And for transportation, he would come and get me and make sure I had a ride. I even told him, “I don't like for my wife to go out with you alone.”

Riham: Yea.

Participant: So when it was my wife's appointment he started to either send a lady instead of him, his co-worker—.

Riham: Yea.

Participant: a caseworker like him—

Riham: Yea. Like he would send a woman.

Participant: Yea, either he would send a woman and even at times when I was home I would go with them or—. I told him, “I don't like my wife to go out alone.” He also used to respect these conditions.

Riham: That's good.

Participant: Although he told me that here in America “It's normal.” I told him, “Honestly, it's not normal for me.”

Riham: Yea.

Participant: I mean, I like to adhere [to my culture or to my faith].

Riham: Yea.

Participant: He also respected all of these things.

Riham: That's really good.

Participant: Until I got the car and I would—. When I got the car, he told me, “Now that you have a car, you can go to your appointments by yourself.” I told him, “Just take me the first time just so I can know where to park the car.” He also started to take me and tell me, “Here,” and tell me, “There.” He taught me a little, and everything is good, thank God.

Riham: How many months did he continue helping you in this manner?

Participant: He is obligated to help me for three months, 90 days. On the 90^th^ day he comes and has me sign what remains of the “welcome.” He gives me papers that say, “This is what I paid for you, electric bills, the place [house or apartment], and everything.” These things are known to us. After 90 days he no longer has anything to do with [a responsibility for] anything. However, until now, anything I need—. I get papers. We get mail. Sometimes I don't know what it means. I mean, we don't know how to translate. Or if we get it translated, they tell me I have to ask—. If someone from the community translates it for me. They tell me, “You have to go back to this person.”

Riham: Yea.

Participant: “He's responsible for these things.”

Riham: You mean so he can explain it to you.

Participant: So he can explain it to me, and sometimes he also needs to fill out paperwork for me. Yea, so, thank God, I go to him until now, and he comes over.

Riham: That's really good.

Ahmed: Thank God.

Riham: Yea, thank God.

Participant: Yea, no, thank God.

Ahmed: I have a question. Your family has been through difficult circumstances, especially your kids, who are young. They also went through circumstances most kids don't have to go through. What do you try to do with your family or with your kids to ease their psychology [or “calm them down”], to not let them get depressed, and such? To improve their psychology?

Participant: Honestly, we have been through very difficult circumstances in Egypt. It was during the worst of my son's illness, when he started to gain and lose everything, when he had his fever and we took him to the hospital, and he stayed there. During that, my wife became pregnant with Aisheh. And we used to—. In Egypt we used to struggle with my son, Ahmad—. He used to—. Now here we are giving him Valium, may God reward them. It really helps him feel comfortable [literally “relaxes his nerves”] because sometimes he feels pain—. There have been many times when she was up late at night rocking him.

Riham: How sad.

Participant: I used to stay up with her—me and her together—then wouldn't be able to go to work in the morning. I told her, “This isn't going to work. I have to go to work. You stay up at night. Then in the daytime, the kid comes—.” Watch out, honey. “Then I'll come home from work 6 PM to 10 PM.” Honestly, we really suffered with this, but it was a favor from the Master of all creation, a favor from the Master of all creation. I'm rocking him from 10 PM to 12 AM. My shift is in the evening, and she would have rested during that time. Then she takes over again at night and rocks him. Yea, so, thank God, we consider this accounted for [in terms of good deeds] by the Master of all creation. Those were the most difficult days we experienced. And, thank God, when my friends came over they would tell me—. I would tell them, “No matter how difficult things are, we feel that our lives are normal.”

Riham: Thank God.

Participant: I mean, this is a favor from the Master of all creation. I mean, may God make it easy on us.

Riham: Of course.

Participant: I mean, if I sit down and explain it to you, you're going to say, “That's a really difficult life! Very, very difficult!” As much as I am explaining it to you and more, quite honestly, but when we lived through it, we found it really normal.

Ahmed: [Indistinct]

Participant: Now we sit down and imagine, saying, “How did we used to sit down and adapt?”

Riham: Glory be to God.

Participant: But, thank God, God made it easy on us. God made it really, really easy on us. They were difficult days. Now here we're giving him Valium, which is calming him down, because his disease really is painful on him.

Riham: Yea, yea, yea.

Participant: He has to take—. Sometimes, something happens where his hands get tight like this.

Riham: How sad. Yea.

Participant: And, of course, that's it, because he doesn't has no control over his movement. He doesn't have a movement.

Riham: Yea, yea.

Participant: So something happens where his hands get tight like this, and—

Riham: [Indistinct]

Participant: for example, during those times he feels pain.

Riham: Of course.

Participant: He—. One day, two days—.

Riham: Yea, spasticity.

Ahmed: Spasticity.

Participant: Now, for example, we're giving him Valium, so he's calming down a little bit [literally “relaxes his nerves”]. I mean, he gets loose a little bit.

Riham: He relaxes.

Participant: Yea, thank God, that's something really good.

Riham: Okay, how about your other kids? Sometimes when kids go to a new country, a new school, don't speak the language, and whatnot and their brother is also sick, you find that they're a little bothered [literally “have shortness of breath”]. What are the things that you do with your family to help make things easier on them?

Participant: Honestly, the kids actually did miss out on a little—. I usually take them out to play a lot. I told you before that our relationship is strong and social [Indistinct]

Ahmed: [Indistinct]

Participant: My family's house has a lot of land, houses. The whole family comes and plays a lot. During travel, we—. Because of being foreigners away from home, there started to be a little bit of distance between families. We'd have to stay at home. Frankly, they were deprived of a few things. I'm not able to conceal this from you. But that's a normal thing. That's how our life is. When we came here, we also found this difficulty until they went to the schools and relieved themselves of it a little bit. Thank God, I try to take them out—. Here we go out in the community. They give us an English class for an hour on Sunday, so all the Syrian families get together. Every one of the kids now has friends. They go and play with each other.

Riham: Nice.

Participant: Because they—. Because we sit down for about one or two hours. The kids play with each other. We, the adults, meet together, the men and the women [separately] have fun together. And, thank God, we also now have relationships with each other. We go visit each other, like—. And we made a group. We help each other. Whoever needs something—. I mean, may God reward them.

Riham: Very good. You mean on the phone? WhatsApp?

Participant: On the phone, yea. Yea.

Riham: Yea.

Participant: And the kids, as I'm telling you, frankly, they missed out on a little. They were wronged a little, but our situation is that we have a son, who is, unfortunately like this. I mean, we can't take them out on a vacation or day trip whenever or whatnot. But, thank God, sometimes, any opportunity we get, we take them because we say, “What did the other ones [kids] do?” [As in, it's not their fault.] Yea, so we take them, but not like we used to before because we have a situation, but, thank God, now we sometimes we take Ahmad. I mean, we take all of them on a day trip. We have a way to help him relax a lot with the sitting. We take them. Like, about two weeks ago—. I have a friend in Michigan. We spent a whole day in Michigan.

Riham: Oh, that's really nice!

Participant: Yea, we went from the morning. We were three families that went together.

Riham: Three families.

Participant: Every family was in their own car. We went to Michigan at 5 AM and got back at 1 AM.

Riham: That's really nice. Where did you go in Michigan?

Participant: Honestly, we went to—. We stayed in—. We were going—. They wanted to go a little bit to the markets. They wanted to go to the—. Because they told me they have a—.

Riham: “The Arabic Area.” [East Dearborn]

Participant: Yea, and we went to a lake. We sat on the lake for about two or three hours.

Riham: That's really nice.

Participant: My friend took me there.

Riham: In Detroit?

Participant: Yea, Detroit.

Riham: Yea.

Participant: Then we went to have lunch in that Old Damascus restaurant [probably Damas Cuisine and Catering in Farmington Hills, MI].

Ahmed: Yea, I went to it. In Dearborn, right?

Participant: The one with the Damascene atmosphere.

Ahmed: Yea, next to the [Indistinct].

Riham: Where's that?

Ahmed: I broke my fast there in Ramadan.

Time (7A.I) – 40:00

Riham: I didn't know about it.

Ahmed: I think it's new.

Participant: Yea, it's new.

Riham: It's new?

Participant: It's really, really nice.

Riham: Is it delicious?

Ahmed: And their food is really delicious.

Participant: Yea, honestly, it's really delicious.

Riham: Where is it?

Participant: It made us feel like—.

Ahmed: I'll ask my family and send it to you.

Riham: Yea.

Participant: It made us feel the exact same atmosphere of Damascus.

Riham: Yea, good, that's really nice.

Participant: We ate Damascene dishes, and he designed a Damascene atmosphere. [Indistinct]

Ahmed: [Indistinct]

Participant: He put windows in the walls and lined the walls with stones.

Riham: Oh, really nice.

Participant: It was organized in a—. He even had a performance [men dressed in traditional Damascene clothes that come out with daggers, shields, swords, and dance to traditional music] when we were sitting.

Riham: A performance, too?

Participant: Yea, we were sitting down, and the performers entered.

Riham: Then you all must have been really amused.

Participant: We had a really great time. Yea, honestly, in the restaurant and the lake where we sat we had a really great time. The reason for the trip is that I have a friend in Michigan—. I mean, this is an old friend from Syria.

Riham: Oh, okay.

Participant: So he beat me here by about six months. So I've been planning with him the whole time. I came to Cincinnati, and he went to Michigan.

Ahmed: Glory be to God!

Participant: I told him, “We'd really like it if we could get together to see each other.”

Riham: Yea.

Participant: So I went and saw him in the—.

Riham: That's really nice.

Participant: No, it was a really nice day. I try whenever there's an opportunity to go and take the kids out. Thank God, I do really well in that regard.

Riham: Thank God.

Participant: And the kids all know that their brother is—. Thank God, they all like him.

Riham: Yea, good.

Participant: Even his brother that is older than him, Abdurrahman, I tell him, “Sit down, honey.” He sits down and reads him stories. He talks to Ahmad a little bit, although he tells me, “Dad, I like to speak with him.” He sits down.Thank God, they take care of him.

Riham: May God preserve them for each other.

Participant: But there's also something we're struggling with, which is the issue of [extended] family. Frankly, my wife is really used to [being with] her family, and it's now been six years since she last saw them. I'm applying now—. We're used to putting all the files we do in my name. We went to Catholic Charities, and I told them, “We'd like to do a reunion for her family.” They told me, “Honestly, if the file is in your name you can't do it for them now. If it's not in her name until she becomes a citizen, in five years.” Yea, so I made the file in my name.

Riham: What do you mean by file?

Participant: Our file for entering the country, Form I-94.

Riham: Yea, but doesn't she have her own file and you have your own file?

Participant: No, we're one family and have one file.

Riham: So with the last name “Arnaut,” you mean?

Participant: Yea. I don't know what they told me. They said, “If it were in her name, we would've brought them.” So now—.

Riham: Why does it make a difference? Aren't they her relatives? They're your relatives.

Participant: Yea, no, a reunion is for the father and mother.

Riham: Yea.

Participant: Or for the spouse or the child, as in first-degree relatives.

Riham: Only first-degree. So if, for example, her sister—.

Participant: Her father and mother, for example, are my father-in-law and my mother-in-law. No, it has to be one's [own] father and mother, for example.

Riham: How sad.

Participant: Yea, so I applied for them a second time. I'm going to a second interview to see what's going to happen because new decisions are coming out. I don't know what they are. I'm really, really hoping if only her parents can come here, really, really hoping. That's the only problem I'm trying to work on—. She's bored [literally “her creation is tight”] and even thinking of traveling for them, but we came here because of an illness. That's what we're finding hard. Because if we have to wait five years until she becomes a citizen before she can see her family—. That's what we're finding difficult.

Riham: That's tough.

Participant: Even for my family and whatnot, we're used to, as I told you, getting together everyday and whatnot. Yea, but, thank God, I—. I have a [a situation?] with a sick kid, so that's it. We're being patient, thank God. But that's the one thing that we found difficult as foreigners away from home.

Riham: Of course.

Participant: Or something that we're not finding a replacement for or something that we're finding we can't compensate for its loss. But as for everything else, like I'm telling you, the community or the kind people—. Like, glory be to God, sometimes when a kind person tells you a nice word or treats your kindly, you start to like the whole country because of them. Thats honestly what happened to us. Thank God.

Riham: Thank God. You're welcome here and like family.

Participant: Thank you. Thank you.

Riham: Thank *you*.

Ahmed: Are there problems you faced or things you found that we haven't talked about yet?

Participant: Honestly, right now, I don't remember anything, but the good things are a lot, thank God.

Ahmed: Thank God.

Participant: The problems are few.

Ahmed: A few?

Participant: Yea, honestly, the good things are a lot.

Riham: Is there something regarding your health, your wife's health, or your kids' that you wish would be better or a project you thought of that might make things easier for other refugees?

Participant: Honestly, as for us, we're good. As for my wife, as I told you, poor lady, she has to sit with the kid, but in her foot/leg is—. Her foot/leg was sprained like this in Egypt for a year and a little. Yea, so until now she can't stand on it a lot and can't—. I took her here to seek medical treatment. They put her a—.

Riham: A cast.

Ahmed: A cast.

Participant: It's like a cast, a foot/leg that she kind of puts on, and she didn't get better. She needs physical therapy and whatnot. That's the one thing we're struggling with a little bit. We want to take her to—. Well, it's hard. Going and coming back is hard for us, especially for my wife. I come and go. So she needs medical treatment, and—. She tells me, “Honestly, it's hard for me to come and go and whatnot.” Because they might give you an appointment during the middle of the week, in the middle of—. I'll be at work. Yea, of course—. That's what's happening. As for everything else, it's all good, thank God. Our other kids' health is all good, thank God. There's nothing wrong with them.

Riham: Thank God.

Participant: Like I was telling you, everything has to do with my wife, so she can go out. She's sitting down and bound up in—.

Ahmed: In her taking care of Ahmad?

Participant: Yea, that's the reason. Thank God, everything is good.

Riham: So if they help you with the nurses—. What I heard from you is that you don't feel safe/at ease if a nurse comes, and your wife leaves, even to walk outside or—. Do you know what I mean? For a little change of scenery. Because it's hard for someone to be with a child for 24 hours.

Participant: That's true.

Riham: If it wasn't any child—.

Participant: That's a good point, but we currently—. Quite frankly, when we go out all together we've planned the whole outing so that Ahmad is not bothered. Either, in most situations, I take my wife and leave Mu'az and Ahmad from the kids and sometimes take the kids and leave my wife with Ahmad.

Riham: I got you.

Participant: But as for—.

Riham: They take turns.

Participant: They take turns. I have to take my kids and come and go.

Riham: Yea.

Participant: I'm waiting for Mu'az until, may God bless him, is able to get a driver license. Then they'll start going out, and I'll take over the shift [at home]. There's no problem with that.

Riham: God willing.

Participant: Yea, but we're used to this. There's no problem.

Riham: Thank God. Thank God.

Participant: But if there is other assistance, like what I was telling you about someone coming over, honestly, she didn't agree. I told my wife. I told her, “Let's see if you're okay with someone coming over from the nurses.” She didn't agree. She said, “No, I don't—.”

Ahmed: It's hard for someone to leave—.

Participant: It's hard. I told her, “For example, let's say we really have to do something, if we call them ahead of time and tell them, 'We have an appointment.' That way there's a—. Maybe we have to do something.” She doesn't agree, but when there's a need, we have to do it.

Riham: Yea, yea. Yea, yea.

Participant: Now, if Mu'az goes to school, and she and I have a doctor's appointment together, we need someone to stay with the kids. So what's been working with us sometimes is either we try to make the appointment at 3:00 PM or 3:30 PM until after Mu'az comes home from school or—. Honestly, a few times, there was a [scheduling] conflict and maybe we missed an appointment or a few? Sometimes there's a conflict, but that's the only thing. Maybe if it's something important, we'll need to. Thank God. When we have to do it, we have to do it. What are we going to do?

Ahmed: [Indistinct]

Participant: I mean, may God reward them.

Riham: Amen.

Ahmed: Abu Mu'az, how's your work? Did you find any difficulty here in Cincinnati?

Participant: Honestly, I like to be committed to work. I have a lot of friends, thank God, who are also working. And I—. Through th e organization, they found me some work, too. They wanted to make me work here and there. The problem with the organization is that no matter what work they have, they want you to work with them. I told them, “No, I have my own profession. I like to work with stucco decorations, which I've been doing for 27 years.” I told him, “To make me work in a restaurant or in cleaning—.” I told him, “It's hard on me—.” I mean, “It's not hard for me to do. I can do anything.” I told him, “But it's not good since I have experience then throw out 27 years of experience to do something else.”

Riham: What's the experience? Sculpture?

Ahmed: With gypsum.

Participant: With decorations.

Riham: Plaster. Plaster, like with—.

Participant: Decoration.

Riham: Like interior decorations in homes.

Participant: Design and whatnot.

Riham: Design, yea.

Participant: Yea, decorations. Like, for example, I brought some pictures with me to show my work [like a portfolio].

Riham: That's really nice.

Participant: Yea.

Riham: That doesn't require the language [knowing English].

Participant: No, it doesn't need the language. There's no need to know the language, but, as I told you, I'm working little by little on the language. Thank God, I insisted a little bit, so they found me work in construction with a guy.

Riham: Yea.

Participant: But most of his work is with paint. I have a little bit of experience with painting. Four of my brothers are painters, and part of our job when working in the shops—. In places [apartments or houses] is meeting with a painter, meeting with a health inspector. I mean, we have experience and an overview of all the work involved. So, I'm working with him. Most of the work is with paint. I work with drywall. I work with everything. Also, as you know, when someone doesn't have a license—. I have a permit to work, but I don't have a—. I don't know what you call the license you're supposed to get. First, I have to know the language. We get a license—. It makes a difference when someone—. I—. Now, he for sure has American workers from this country. For sure they're not getting paid like me.

Riham: But they're not supposed to be paid differently than you.

Participant: Because they—. No, you're supposed to have a license or something. I don't have a language degree or a license for—. It's knowing the language plus—. It takes—. Honestly, they told us about it in the educational session. They told us about it. I forgot what it was. It was like, “To be paid like the others you have to have a license to work,” they call it. Like, “They get you a license to work from the specialists in that profession.”

Riham: Oh, okay, like a “license.”

Participant: Yea, that's right. A license.

Riham: A license.

Participant: They said, “Even if you have experience and everything, if you don't have this license, he'll pay you whatever he wants. It doesn't—.” Do you know what I mean?

Riham: Okay.

Time (7A.I) – 50:00

Participant: So that's is another thing I'm determined to learn.

Ahmed: God willing.

Participant: And we'll see how it goes, God willing. But as for the work situation, thank God, when I started working with him, I told the guy I'm working with—. I told him, “Let me tell you something. I want to work full-time, but the problem is that I have a lot of appointments.” And from the time I started working until now—. It's been about three months since I started working. T here wasn't a time when I worked over 30 hours, and several weeks have passed when I've only worked 20 hours or about 15 hours because I can't work. He told me, “The work is there for you when you want. You can work 40 hours, but you can't because you're not free.” I told him. I told him the condition from the beginning and said, “I take a lot of time off because I have a kid. I mean, I have a lot of appointments.” Like, for example, last week I think I had three appointments for Ahmad.

Riham: So are you able to combine your appointments so that you won't miss a lot of work?

Participant: Honestly, I'm missing a lot of work. Like I'm telling you, I'm not working more than 20 or 30 hours. Maybe once or twice I hit 30, but you can pretty much say that 90% of the time I work between 20 and 25 hours.

Riham: So can you stack up your—. Can you tell the doctors in the hospitals, “I don't want to miss any work. If you want to give me any appointments, make them on Monday all right after each other.”

Participant: For me to take off Monday?

Riham: For example, I mean. Either Monday, Wedesday, or Thursday so that you miss one day and not miss three days for three appointments. Do you know what I mean?

Participant: I got you. Honestly, I sometimes make the appointments later in the day. I'm trying as much as possible, but when they're necessary appointments and whatnot, I try to tell them, “Put them on Friday.” Because I also don't work on Friday because I have prayer and have—.

Riham: Yea.

Participant: I only work four days.

Riham: Okay.

Participant: Monday, Tuesday, Wednesday, and Thursday.

Riham: So, if you made all of your appointments on Friday.

Participant: Yea, when I'm trying as much as possible to make them on Friday, they're great. Honestly, sometimes really important appointments are coming, and they tell me, “It's not possible.” For example, his appointments in a week on Monday. If it's moved to Friday he'll missed two more weeks [i.e. two weeks pass instead of one] or three, especially for teeth. Especially for—. For teeth, they give me each appointment after a month or a month and a little.

Riham: Yea.

Participant: I tell them, for example, “I don't want to.” They then delay it more. I mean, whatever I'm able to take , I'm trying to—. What I'm not able to, I'm okay with—.

Riham: Are you finding that you have to wait a lot to find an appropriate appointment?

Participant: Other appointments [with a different time], yea, honestly. Yea, thank God in every situation.

Riham: Are there things you do at home to, for example—. Like, you spoke to me about teeth a few times. Are there things you do at home or learned a long time for someone to take care of their teeth? Or to take care of their body?

Participant: Yea, thank God, as for brushing our teeth and whatnot, thank God, we're taking care of these things, thank God.

Riham: Is there another material, an herb, or something like that you use? Do you know what I mean?

Participant: Honestly, I brought a miswak with me [a teeth cleaning twig from Salvadora persica tree, known as "araak" in Arabic], but I'm getting lazy. However, the miswak makes it so we don't need anything else. When we first came, we thought we might not find it here, so I brought some with me, but, honestly, I've been lazy.

Riham: Yea.

Participant: It's neglect, quite frankly.

Riham: Not just your teeth. Tell me about the other things. Like, do you find—.

Ahmed: Natural remedies, for example. Do you use them at all?

Participant: No, honestly, in our place [apartment or house] here we don't. Thank God, frankly, we found a difference between the air in Egypt and the air here. Like, today in particular, my kids were saying, “Thank God—.” Over there we used to—. Every month, every two or three weeks a kid used to get the flu/catch a cold then the rest used to get sick like them, too. Yea, so, thank God, there's a difference with the natural air. The air in Egypt was quite frankly a bit difficult, and hot and—. Yea, so here it made a difference with us, thank God. Since coming, they've maybe only once had the flu/a cold, and, thank God, until now they've been good.

Riham: Thank God.

Ahmed: Thank God.

Participant: Of course, we take herbs and whatnot. These are normal things, and we're like anybody else.

Riham: Yea, of course.

Participant: I mean, there's nothing special about us or specific that we take.

Riham: And what are the things that make you say, “I have to go to the ER or have to make an appointment with the doctor?” What are the things that make you immediately think, “I have to go see a doctor about this?”

Participant: Honestly—.

Riham: You told me fevers.

Participant: As for us—. As for my son, right when he gets a fever—. He has a fever today, but they told us he took a vaccination about a week ago. They said after a week he'll no longer have a fever. As for the kids, when they first get a fever—. Because we're struggling with Ahmad's situation, frankly fevers really terrify me.

Riham: Yea, yea.

Participant: For those, I immediately take the kids to the hospital. If there's a fever, [Indistinct] I'll take a look at him. Right when it goes above 38 C, I immediately take them. The temperature.

Riham: Is there anything else?

Participant: No, thank God. If there's anything that's an emergency, we'll go in for it, but if there's nothing that's an emergency then—.

Riham: Like, I'm asking you so that I can know what things you consider emergencies.

Participant: No, I mean, for example, if a kid falls really hard and something happened. For example, he fainted—.

Riham: He tore his skin—.

Participant: Yea, good.

Riham: Or—.

Participant: For example, big injuries. That's what I mean by emergencies. Things that are basic, so immediately I think about taking them. But, thank God, everything else is—. For fevers, I immediately take them in. As for me, I really get afraid of fevers. Or maybe if he gets a fever at night, I say, “Wait until the morning. Let's see.” I take them immediately. Except if they've been vaccinated. Then we know that there's going to be a fever because we've gone through a lot of phases of vaccinations, and all the kids are still getting vaccinated until now. So they're getting fevers, and that's something normal. But Ahmad, when they vaccinated him, they said he'll get a fever, but, honestly, we have to keep our eye on him.

Riham: Yea, you have to.

Ahmed: You have to.

Participant: Yea, if he gets a fever—. Because a fever doesn't help his disease at all.

Ahmed: You told us.

Participant: Yea, honestly.

Ahmed: Did you find any problems with figuring out how the health system here works? Because it's really different than our country, right?

Participant: Yea, a lot, honestly. The problem is that—.

Ahmed: Can you tell us—.

Participant: here everything is slow. Everything is done slowly. Like I took my son once, this same one here, Ahmad—. I took him. They told me that with his disease his bones become brittle, very soft. So really, I'm putting him into the car—. I swear to God, I'm really, really careful. One time I'm putting him in the car, and his foot/leg bent and broke. About two weeks ago I took it [the cast] off. They told me it's a hairline fracture, not broken badly. So I'm putting him in, and suddenly—. I was really bothered with putting him into the car. So his foot/leg bent. I looked. “Where's his foot/leg?” It turned out his foot/leg was bent. I was really, really bothered. And he cried! He cries when he has extreme pain. He cried, honestly, so I said, “For sure there's something.” I took him to the doctor. She said—. The next day she said, “It's hurting him when I touch him on his leg. We have to take x-rays.” We got x-rays for him, and it turned out that there's a fracture. They said it's a hairline fracture. We put a cast on him for two weeks, and, thank God, we took it off. The doctor told me, “There's no need for a cast, but just keep an eye on him more.” So when I took him to get his cast, he was also sneezing a lot. When he was sneezing, the hose came out of his nostrils—that hose that they give him as a feeding tube. Yea, so it came out of his nose. I told myself, “I'm in the hospital anyways.” I asked them to replace it, not that they put it back in because it had passed the—. It had come out a lot. The doctors in the hospital taught me how to put it back in if it comes out and how to put one in for him. I also have experience because even in Egypt I used to put him this tube. I would see how the doctor used to put it in and whatnot, so I learned how to do it. So when they—. By the time I put it in, he got a cast, and within half an hour or whatnot—. But it honestly took about four hours for them to put the tube in for them to transfer me to the ER then for a nurse to come then I don't even know what else. I mean, just for this thing it was very, very slow for them. Yesterday my wife was telling me—. She told me, “Honestly, Ahmad also—. It came out—. He also was sneezing a lot yesterday. I also took him to the park a little. He sneezed a lot, and the hose came out.” She said, “It all came out.” Yea, so, thank God, I put it in for him. Honestly, it was the easiest time it was put it in for him. I put it in myself. I used to be afraid—. I used to be afraid that—. I'm not a doctor. I might be putting it in, and it goes into the airway, God forbid. But here when the doctors and nurses told me, “If necessary, you can put it in.” it's like they gave me confidence [in myself]. Do you know what I mean? I said, “That's it. That means I'm not punishing the kid.” Yea, so I found myself that my heart felt at ease with doing it, and I put it for him yesterday. Honestly, I really did help him feel more relaxed and at ease than the nurses that used to put it for him and the doctor that used to put it for him in Egypt.

Ahmed: Great job.

Participant: Yea, thank God, because it was really, really easy yesterday.

Ahmed: Thank God.

Participant: And the nurse that I told you put it in for him in four hours put him the hose and said, “I have to withdraw so something can come out of his stomach. She put it in and was sucking and sucking [i.e. aspirating using a suction syringe], but nothing came out. She had to—. She told me, “Honestly—.” She listened to him with the stethoscope to see that it's there inside, but she said, “I have to make sure. Let's take an image.” We also took him to take images [ultrasound?] to see if it's in the right place or not. So yesterday, right when I put it in—. I used the syringe—. I was sucking [aspirating], and it immediately came up [the aspirate] with me. It was a proper [Indistinct], thank God.

Ahmed: Thank God. May God make it easy.

Participant: Yea, thank God. Thank God.

Riham: May God reward you! You're troubling yourself!

Ahmed: [Indistinct]

Participant: [Indistinct] the serving table that's next to you.

Riham: God bless your hands. Is there sugar though?

Participant's Wife: Yea, there's sugar. Do you want some?

Riham: No, I really love sugar..

Ahmed: She always loves sugar.

Riham: How many spoonfuls?

Participant's Wife: One and a half. You also see.

Participant: Put some extra. Put more sugar.

Riham: I love sweet things.

Participant: Honestly, we have a big serving table.

Time (7A.I) – 60:00

Participant: but the little one pushed it, and the glass came out.

Riham: How sad! Hopefully she didn't get any glass in him?

Participant: No, no, thank God.

Riham: Thank God. May God bless your hands. We've taken a lot of your time, Abu Mu'az. Are there any other questions?

Participant: No, there aren't.

Riham: What do you think?

Ahmed: Is there anything else?

Riham: Yea, I think that was everything.

Ahmed: Okay.

Riham: Anything else—? Before you shut it off—. Is there anything else you think we can help refugees with—? Thank you very much, may God bless your hands. That we can help refugees with regards to children's health?

Participant: Honestly, I'm going to tell you something. Now, I'm telling that through God's favor, God has made it easy on me, but I sense some struggling from my friends, quite frankly.

Riham: Yea, so tell me. I can talk with people—.

Participant: Now, my friend—.

Riham: Yea.

Participant: Like, I have a friend here—. In the org—. His son doesn't have anything wrong with him. So when we want to travel—. When refugees travel from country to country, there are vaccinations they have to take. They're giving his son a vaccination, then the boy became paralyzed by taking the vaccine shots. I don't know if you know him or not.

Riham: Yea. Yea, I know him.

Participant: Yea, so now the guy is struggling—. I don't know. Maybe he whines/complains a lot or whatnot, but he's struggling. He needs a medical report that—for his son, but they're not giving him a medical report. So I don't know his situation, but I'm talking about something I heard from him.

Riham: Yea.

Ahmed: Okay.

Riham: But—.

Participant: I told you about my situation. I know my situation, and I've lived through it.

Riham: Okay.

Participant: But as for others' situations, I'm just telling you what I was told—.

Riham: But what suggestions do you have? For example, you're hearing from your friends that they have this problem, this problem, and that problem. In your opinion, what is something that, for example, is coming easy to you that you wish would be applied for someone else? Do you know what I mean?

Participant: I got you. Honestly, nothing specific is coming to mind. As for me, thank God, as I'm telling you, everything is good. Everything is great, and there are no shortcomings with regards to Ahmad, thank God. I wish it were like that with all people.

Riham: Of course.

Participant: Now, anything I request—. Thank God, the nurses come every Wednesday to see my son.

Riham: Okay.

Participant: Every Wednesday they come to see my son. They examine him. They see him. And I'm following up with StarShine.

Riham: Oh, yea, yea, StarShine are great!

Participant: Yea, those people. So anything that we're missing, bags or whatnot, they send it all here for my son.

Riham: Good.

Participant: Anything I need—. Thank God, everything is good for us, thank God.

Riham: Thank God.

Participant: And we wish it were that way for everybody. We don't like for it to be just special to us, and, thank God, it's generally that way for all people.

Riham: Yea. Thank God.

Participant: Yea, thank God.

Riham: Okay, thank you very much for the [Indistinct].

Ahmed: Thank you very much for the [Indistinct]

Participant: May God reward you.

Riham: For your thoughts—.

Ahmed: And—.

Time (7A.II) – 0:00

Ahmed: Today is July 17. We're still with Participant 7A. Okay.

Riham: Finish the story.

Ahmed: Yea, finish the story.

Participant: Now, with regards to—. There's just one thing about appointments. The other day we had an appointment. Someone was going to come over. Who was with him?

Participant's Wife: [Indistinct]

Participant: Honestly, I don't know who.

Participant's Wife: [Indistinct] interview [Indistinct] the children

Participant: The problem is when they sent me letters to confirm, I don't know that I have confirm it for them like that. I mean, if they give me an appointment—

Riham: Okay.

Participant: I like to just immediately record it in my calendar/planner here. Do you know what I mean?

Riham: Oh, yea.

Participant: Do you know what I mean? They send me a letter to confirm, either yes or no. So maybe I didn't send it out. Maybe he tells himself, “He didn't confirm the appointment,” so he doesn't come, but I would've had already left my work and came here.

Riham: How sad. So that's a language problem.

Participant: It's a language problem. He spoke with me in English. My son spoke with him and knew how to manage. He told him, “I'm coming to you on Monday or whatever day it was.” We specified it and came. Then they send letters to us to confirm the appointment. I didn't know what to do so didn't send it back. There are others I don't send it to. I mean, hoow many appointments are established—? But as for—.

Ahmed: Okay.

Participant: But, I mean sometimes things like this—. The school, the kids—. They send us a lot of letters. Or they call me a lot. The most difficult thing that I remember right now is when he calls us by computer. I mean an automated message.

Riham: Oh, to remind you of appointments.

Participant: Yea to remind me or appointments or to tell me something for the school, for my kids. They call me and tell me “This is the children's school—.” They speak to us in—. I'm neither able to call them and tell them I need an interpreter—. And they speak to me without—. If it were a text message, I'd be able to give it to a translator. I know some—. Or I'd be able to show it to my friends to tell me what it means.

Riham: Yea, yea.

Participant: I'd wish that instead of that robot talking to me, they talk to me with a text message.

Riham: Okay.

Participant: Do you know what I mean? That's something I struggled with.

Riham: Yea. That's actually a really good idea.

Participant: It's really good, but it's not just me. My friends—.

Ahmed: It's not hard.

Riham: Yea.

Participant: It's not just me. My friends are really struggling with it.

Riham: Yea.

Participant: When a robot is talking to us, for example—. When they're talking to us with a computer, they sometimes tell me “If your son is—,” “If you're Mu'az's father, press 1. If you're the father of the other one—.” I'm sorry. “If you're not his father, press 2.” Whatever it was, press 1.

Riham: Yea.

Participant: That's something we're struggling with. Instead of a robot speaking to me with a computer, for example, either they speak to us with an interpreter or they send us a text message. I'm able to translate a text message. I'm able to send it to one of my friends.

Riham: Yea.

Participant: I mean, that's something—.

Riham: [Indistinct] Or even your son can—.

Participant: He can even read it to me.

Riham: He can even read it to you.

Participant: That's one idea I thought about that.

Riham: Honestly, that's a really nice idea because it might not be hard—

Participant: Not at all.

Riham: but nobody thinks about it. Because they might be thinking “He can't read English,” but they can—.

Ahmed: [Indistinct]

Riham: I mean, they can—. You can copy it and put it immediately.

Participant: Exactly. The problem is that this number calls you, and you're not able to call it back.

Ahmed: [Indistinct]

Riham: Yea, okay.

Participant: For example, this number calls you. It can call but can't accept calls.

Ahmed: Okay.

Participant: Being able to understand or having my son speak with them isn't working out.

Ahmed: [Indistinct]

Participant: Yea, honestly, that's something we're struggling with. But as for other things—. With regards to the children's hospital, I mean, they get me an interpreter. There was also something else. For me, I don't like for someone to come that's a—. I mean, I don't like to take my wife to a male doctor. I like to take her to a female doctor.

Ahmed: Okay.

Participant: At the same time, I don't like to go—. One time I took her—.

Riham: She's probably not comfortable.

Participant: She's not comfortable, and even here they bring an interpreter with them. During this last period, it happened to be that they brought a male interpreter with them. I mean, may God reward them, I told them, “If that's the case, I want to cancel the appointment.”

Riham: Yea.

Participant: Yea, they also told me, “Okay, that's it. We're not going to bring a male interpreter with us anymore.” They canceled on him and told him, “We're only going to bring a female interpreter.”

Riham: For you or for your son?

Participant: For when they come here for my son. Sometimes—. If I'm here, it's not a problem.

Riham: Yea, but when your wife is alone—.

Participant: When I'm not here [home] and my wife's alone—. Frankly, when the female nurses are coming on Wednesdays, and a female interpreter is coming along, she's becoming a like a friend to her.

Riham: Yea, that's really nice.

Participant: She sits down and talks with them. Then she doesn't become too shy to say anything. There are some things she's shy about saying in front of a man.

Riham: Yea, of course.

Participant: When it's a female interpreter and—. I mean, they're all female nurses that are coming. When a female interpreter comes, she's herself around her and speaks naturally and with comfort. She says, “They came like this. They said that.”

Riham: Yea, that's nice.

Participant: If it's a male interpreter, every thing's totally different.

Riham: Yea.

Ahmed: Okay.

Participant: No male interpreters are apparently going to come anymore, thank God. This was a problem I had, and they almost completely solved it for me.

Riham: Solved it for you.

Ahmed: Yea, thank God.

Riham: You solved it.

Participant: I solved it—. No, they told me “If this thing happens with you, let us know.” I told them, “Honestly, I'm bothered by this.” Then they brought a male interpreter another time. Then I brought it to their attention. They said, “If it happens again, let us know.” Then I went back and informed our social worker, and she did a great job and didn't have any shortcomings, may God reward her. She told me, “There's an appointment tomorrow. I'm going to cancel it, but we might not always be able to secure an interpreter. It might be via phone.” I told her, “It's not a problem at all.”

Riham: At least he's not in front of you.

Participant: Exactly. By phone—. Actually, by phone we mostly get females. Yea, thank God, things are going well.

Ahmed: I have something I remembered. How has your experience been with the phone interpreters?

Participant: No, thank God.

Ahmed: Did you have to use them or—?

Participant: It's really excellent. Even when I call the hospital and have—. Or if I have anything, I have the interpreter's number. He speaks with—.

Ahmed: Okay, that's great.

Participant: And for me, thank God, there were three phone calls in which they spoke to me in English, and I was able to manage, not a lot but a little. But I mean I understood them, thank God.

Ahmed: Okay.

Participant: I started. But my wife, because she's at home—. I mean, I'm going outside and interacting with people. I'm going out. I started a little bit. My oldest son knows, thank God, but my wife, frankly, isn't able to speak.

Ahmed: Okay.

Participant: Yea, like I'm telling you, my wife is missing a lot of things. It's true that she's sitting here [as in “remaining at home”]. She's missing out on a lot of things, but we're forced, I mean, thank God.

Riham: May God reward her.

Participant: May God reward her.

Ahmed: I'm going to go back and stop the recording. Thank you.

Interview 7B

Time (7B) – 0:00

Riham: Okay, today is July 17, 2017. We're with a mother that has six kids. And she has—. She has been here for seven months. Okay? In Cincinnati.

Ahmed: 'Umm Mu'az, when did you come to Cincinnati?

Participant: In the month of—. In the middle of December.

Ahmed: In the middle of December? And can you tell us why you came to Cincinnati?

Participant: Honestly, we applied for migration for my son's medical treatment, and there they transferred us over to Cincinnati.

Ahmed: In Cincinnati? So that means you didn't chose it.

Participant: No. They chose it for us. Then, I don't know. We heard from people that they see what place is best for a kid's medical treatment and send them there.

Riham: Okay.

Ahmed: Okay. Tell me about your feelings about moving from a country that has an Arab society to a country that has a foreign society and foreign language.

Riham: Because you're coming from Egypt. Like, you went from Syria to Egypt. You stayed for a long time, but, of course, you understand the language [there].

Participant: Yea, that's right. Now, it's hard, and since coming I've been worried [literally “eating my worries about him”] about my young children in terms of—. I mean, I don't want them to forget their language. [I want them to] learn Arabic. But, at the same time, the thing that I liked, that motivated me to come here was my son's medical treatment. That thing kind of surpassed the other things. But, at the same time, until now I want my children to learn Arabic.

Riham: You're going to teach them, right?

Participant: Now, it's hard for me to teach them. I have a lot of work [things keeping me busy]. I'm trying, but it's a difficult topic/issue.

Riham: Like, when you say “teach them,” what does that mean?

Participant: I mean I want to teach them how to read and write.

Riham: Reading and writing.

Participant: Yea.

Riham: Like grammar/rules.

Participant: Yea, yes. Now, they know how to speak.

Riham: Thank God.

Participant: And I've told them, as much as they speak, as much as they learn in the schools, there's no speaking English at home to—.

Riham: Preserve.

Participant: preserve their language.

Riham: Okay.

Participant: But that doesn't teach them how to read to read the Qur'an, to know the matters of their religion, I mean.

Riham: Yea. Are there any schools or anything for Arabic or the religion [Islam] or the Qur'an that they can enroll in?

Participant: Now, there are schools but private schools. Not schools for everyone. Private schools get a little expensive now for us being that we're new to the country.

Riham: Yea.

Participant: Yea, it's a little bit hard on us. Despite that, people are helping us, may God reward them.

Riham: Like, there aren't free classes? In the mosque or anything?

Participant: Now, in the mosque, we're going and taking English. They said there is, but my kids are young. So there only taking for the older ages still.

Riham: Those classes are only English classes.

Participant: For English, they have it for all ages. We're even taking English.

Riham: Yea, but I'm asking you: Are there free Arabic and Qur'an classes for children?

Participant: I heard that there was, yes, but my kids' ages are still too young—.

Riham: But your son—.

Participant: Like, my son is six years old. The other is three years old. They're still young.

Riham: And you have one that's—what is it—12 and another 16.

Participant: Yea, the 12-year-old knows how to read and write. I'm not worried about him.

Riham: Oh, you aren't worried about him.

Participant: Exactly.

Riham: I got you.

Participant: Yea, there are the two young ones, and the young sister, too. I mean, those are the ones I'm worried about.

Riham: Yea. They still don't write anything, right?

Participant: No, I have Hamze. In Egypt—. In Egypt, he had started preschool.

Riham: Oh, he is now six years old.

Participant: I mean, he started learning the letters.

Riham: Yea.

Participant: Then we had to travel, and we had to leave, so, I mean, he didn't get to learn [because of not having enough time to do so]. That's also something we struggled with. It's hard on us.

Riham: Yea, of course it's hard because it's a foreign environment [culture], and, at the same time, I mean, it's something really dear to you [literally “close to your heart”] that you don't want to let go of.

Participant: Exactly. Yea, yes. And, I mean, I have hope that I'll return to my country one day.

Riham: God willing!

Ahmed: God willing.

Riham: God willing, if it's written [destined] for you.

Participant: So all my kids can preserve their language and religion, God willing.

Riham: You—. When we were talking with your husband, he told us about your son, and he told us a little about his condition and whatnot. And, of course, the whole reason you came here is for his health. At the same time, you've been—. For how many years have you not you seen your family?

Participant: It's been about six year.

Riham: Like, your mom and dad?

Participant: Yea, yes.

Riham: And your siblings?

Participant: Five and a half years for my siblings.

Riham: That means—. We count the half.

Participant: Yea, because when I left my family—. When I left Syria, I went to my siblings'.

Riham: Oh.

Participant: My siblings were in Saudi Arabia.

Riham: Oh, okay, okay.

Participant: I stayed with them for six months then went to Egypt.

Riham: Okay. And the—. I mean, it's hard for someone to be away from their family.

Participant: Yea, yes, and we're used to always be around each other. Even us siblings always being together. So this separation was a bit difficult on us.

Riham: Really difficult.

Participant: Exactly. When a person has a situation like this—. I mean, I had my son's situation, and—. Like, nobody from my family is close to me. I mean, that was also difficult on us. There's nobody to keep you company, to be near you—. I mean, from your family.

Riham: Okay.

Participant: I mean, to raise your spirits. That's also something difficult.

Riham: Very difficult.

Participant: Yea, yes.

Riham: What do you use to kind of accompany yourself or strengthen yourself in this situation?

Participant: With supplications. I mean, I pray and am always supplicating [Indistinct] for the Master of all creation to answer the supplication one of these days.

Riham: God willing.

Participant: God willing.

Riham: Is there anything else that helps you?

Participant: No, like—. Of course, life distracts. Kids distract a little and whatnot. And life is, glory be to God—. Like, the Master of all creation is—. I mean, gave us a difficult test, and this thing—. Despite it, He is helping us, thank God.

Riham: Thank God.

Participant: Like when I think, “How did I leave through these things?” I find it very difficult. It's like, “How did I endure these things? I don't know.” That's all, glory be to God—. The Master of creation made it easy on us.

Riham: Glory be to God. Your husband told us that when you arrived there was a community that welcomed you and helped him. In your opinion does the community help you? Or is your sense of community something that strengthens you and helps you adjust [helps you cope] with the circumstances you're experiencing?

Participant: Yea, of course. When somebody sees someone supporting them [literally “standing with them”], they feel like someone like their siblings are with them. When we arrived, may God reward them, even community started sending their women to us [as in, “women starting visiting us”].

Riham: Nice.

Participant: Despite that, until now -- there's someone from the community responsible for us. She tells us, “If you need anything you have to let me know.” And she's always in touch with us until now.

Riham: Thank God.

Participant: That's something that makes a person feel at ease, of course—. When they see someone from their country or an Arab that is concerned with them. I mean, they feel a little—. Honestly, may God YUQADDIRUNA, dear God. May He make our circumstances easy on us.

Riham: Amen, dear God. Honestly, being a foreigner is really difficult.

Participant: Yes, very, honestly. My family lived in Saudi Arabia for about 20 years. From a long time ago. Like, I was born in Syria but was raised in Saudi Arabia.

Riham: Ah, okay.

Participant: But when I got married, we went down to Syria. And my family—. I mean, I'm the last of my siblings to get married, so [Indistinct]—.

Riham: Are you the youngest?

Participant: Now, there is someone younger than me. My brother was, I mean. But we girls got married first.

Riham: Yea.

Participant: So, after my family got me married, they didn't let themselves leave me and go to Saudi Arabia, so they came with me, as in, to—.

Riham: Nice. So they followed you then!

Participant: Yea.

Ahmed: [Indistinct]

Riham: You're their baby.

Participant: Yea. I'm the spoiled one.

Riham: Yea, the spoiled one.

Participant: So from that time, I never separated from my family. So that separation was hard on us for all this time.

Riham: [Were] they living with you at home?

Participant: No, but for the first period of my marriage, I lived in the same neighborhood as them. Then I moved to my home. Then I started going over to my family's and even sleeping over some days. I mean, my husband's mom is old. He would go sleep over at her place. I would also spend the day at to my family's. I mean, in addition, marriage didn't stop me from separating from my family or keeping away from them [I think she meant “seeing my family and spending time with them”]. I mean I stayed with them at the same time [i.e. that I was married].

Riham: But that situation has now changed a lot.

Participant: Yea, yes. From when the incidents started—. In the country we even started—. I was in the country at one point and couldn't see them for a month.

Riham: Wow.

Participant: Yea, they were in a place, and I was in a place. We couldn't reach each other.

Ahmed: Okay. Well, were they in “al-'Ada” [a neighborhood?].

Participant: They were—. No—. The incidents started in “al-'Ada,” and they went and stayed—. They stayed on Baghdad Street at my brother's in-laws'.

Ahmed: Okay, Baghdad Street.

Participant: Exactly, and I stayed with my brother-in-law's in “Sahnayah” [a neighborhood?].

Ahmed: Okay.

Participant: So we couldn't leave [Indistinct]. We ended up in the same city but far from each other.

Riham: Glory be to God.

Participant: Until now there are a lot of people like that. It's really hard for them to get in touch with their family in the same city.

Riham: Do you have a phone you use to be able to speak with them or not?

Participant: Yea, I talk to them online, thank God.

Riham: Good, thank God.

Time (7B) – 10:00

Participant: But now, my mom and dad are in a situation where they've been left alone. All their kids emigrated. So they're now staying alone. So this thing has also made us really worried about them [literally “eating our worries about them”].

Riham: Of course, because they're staying alone.

Participant: They're alone. The whole family, relatives, are close to them, but, of course, one's kids are different.

Riham: Yea, of course. In these circumstances when they changed, you are saying that you struggled with these things. What are the things that you talk with your children about to help them adjust here or help them relax, psychologically speaking?

Participant: Now, I tell them “The language, learning the language, schools—. I mean, that's going to be your future. You're going to have a future. If we return to our country, the future is—. I mean, having a language [knowing a foreign language] there has become something basic. So you're going to have a language [know a foreign language]—.” This is like a huge treasure for them, in their lives. Yea, God willing, dear God.

Riham: God willing.

Participant: God willing, we're able.

Riham: Is there anything you do at home as a mother—. Not just talking with them but, for example, things that you do to preserve their emotional and physical health?

Ahmed: Especially kids, when they go through difficult circumstances they [Indistinct] emotions, and it could affect them in a negative way. Do you do anything at home to help them? To help them deal with these emotions that are [negative]

Participant: Now, we try as much as possible to have them go out, even if just here outside. If they go outside and have a change of scenery, they calm down a little. When we first came, for a while when their brother was in the hospital, for the approximately 10 days, they had a lot of stress on them. There were no schools [in session], and they wouldn't see anybody here, so the situation was hard on them. I used to take them a little bit outside.

Riham: It was even during the winter.

Participant: Exactly. It was wintertime. Yea, it was hard, but when they started school, I told them, “Tomorrow you'll start school. You'll meet new friends.” They were even asking to go back to Egypt in the first period. “Our friends are there.” I told them, “Once you start school, you'll meet friends, and life will change a little.” Thank God, after a little, they got distracted a little with school.

Riham: Thank God.

Ahmed: Do you go with Ahmad to his appointments in the hospital?

Participant: No, his father goes.

Ahmed: His father goes with him?

Participant: Yea.

Ahmed: Did you ever have to go to the hospital?

Participant: Yea, when he was in the hospital, I used to go and visit him.

Ahmed: Okay.

Participant: There in the hospital.

Riham: How did you get to the hospital when—.

Participant: One time there was a man—. He's the one from the community. He came and took us, me and my kids. One time they also sent me someone from the community. A lady came and took me to the hospital.

Ahmed: Okay.

Riham: So when you got to know the community, they helped you with transportation?

Participant: Yea, for something like the hospital, they used to help me. One time, a lady even came and took me if I needed things [groceries], and she brought me things [groceries], being that my husband was in the hospital. When we first arrived, my kids lost weight, so she brought me drugs [medicine] for the kids. She helped a lot.

Riham: Was this lady a volunteer or—?

Participant: In the community.

Riham: Oh, in the community. May God reward her.

Participant: Amen. Yea, honestly. Yea, may God reward them. They helped us a lot in such a situation.

Riham: Very good. Do you have to speak with doctors or make appointments for your son?

Participant: Now, sometimes when my husband is at work, if something hard happens with Ahmad, I call the hospital and see—. I tell them about his condition. If he needs an appointment, they make one for him. Yea. Or they come over to see him.

Riham: Okay. Do you find any problems when speaking with them on the phone?

Participant: No, they told me—. They gave me an interpreter's number to call and said he would transfer me to the department I wanted.

Riham: Immediately.

Participant: Yea.

Riham: And do you have any problems knowing which department or which doctor or anything or?

Participant: No, now, I request the department responsible for him, Starshine, and I tell them his condition. Then they transfer me to the proper department. Yea.

Riham: I got you.

Participant: Yea, but one time I called, and I waited for 10 minutes until they transferred me to the department.

Riham: Ten minutes.

Participant: I mean, how I saw it was that sometimes my son's condition gets dangerous.

Riham: Yea.

Participant: For something like that, it's hard for someone to wait all of that time on the phone.

Riham: There is—. We're hearing that—. I don't know the Syrian or Egyptian health care system. Actually, I was born here, but tell me—. People are saying “We're waiting a lot here in America.” Did you feel like that or not?

Participant: Do you mean in Syria and Egypt?

Riham: I mean, if you're comparing. Between here and there.

Participant: Now, here we wait more, but we see results. Like, me, honestly, when I was in Egypt, and what happened to me in Syria when they discovered my son's disease, I mean, I lost hope in Arab doctors. They don't have any concern whatsoever for children, for these difficult conditions like my son's. It's like they see that it's a difficult situation and whatnot. It's like they see a condition and say “That's it. This is difficult.” It's like they lost hope and just leave it. That's something I didn't find here, even though I was afraid that I was coming to a foreign country. “How am I going to give them my son?” No, but, thank God, I found that they were very responsive with us.

Riham: Thank God. So do you feel safe [trust/at peace] with your doctors?

Participant: Yea, thank God.

Riham: Thank God.

Participant: Of course, we put our trust in the Master of all creation. He's the one, who strengthens us with this, and gives us more hope. At times I try to—. I feel that I'm fooling myself or something, but when I supplicate to the Master of all creation and read Qur'an I feel a piece of mind. I mean, I go back and feel reassured more.

Riham: Thank God.

Participant: Thank God.

Riham: So you find your religion helps you the most.

Participant: Yea, yes. Yea, in my separation from my country, I am far from my family and—. I get days where sometimes I can't talk to anybody. Yea, but with supplications and whatnot, I relax, thank God.

Riham: Thank God. May God comfort you.

Participant: Amen. May God protect you from harm. May God comfort everyone.

Riham: Amen.

Ahmed: Can you talk with us more about how your psychology was affected. Because you experienced—. You are experiencing difficult circumstances, and you have to stay with your son all the time at home. As you said, you don't get a lot of opportunities to go out. Your family isn't with you. How has that affected your psychology? And I just want to add a question. How is that affecting your kids' psychology?

Participant: Now, the kids, thank God, are not affected too much. They're distracted by play and whatnot. I mean, they don't feel too much. But sometimes I get mentally fatigued. I feel my psyche is exhausted and whatnot, but my husband takes me out. I keep Mu'az with him, and have a change of scenery then, thank God, I manage.

Riham: May God preserve you for each other.

Participant: May God protect you from harm. Thank God.

Riham: Tell me about how your health is here in comparison to how your health was in Syria, in Egypt, and here.

Participant: Now, in Syria, thank God, as a condition, it's true that I didn't used to struggle with anything. In Egypt, I struggled. I had a sort of asthma. I wasn't able to—. I mean, I was treated but didn't get better through it. Yea and I struggled with it more because I was sick and was seeing my son sick, too.

Riham: How sad.

Participant: And pregnant at the same time. I mean, that was really hard on us, but, thank God, after I came here, things got better. All that's left is the thing with my foot/leg here. And I've left it alone thinking it might heal with the passing of time.

Riham: Tell me about your foot/leg a little.

Participant: My foot/leg—.

Riham: God willing it'll heal, but let's hear your foot/leg's story!

Participant: I was in Egypt and had given birth a month earlier. It was the first time I leave the house after giving birth.

Riham: Yea.

Participant: So it twisted, and I fell down on it. So, I should have gone to get it x-rayed, but I started worrying about how I'm going to go and leave my kids at home, and it's hard. And if they put a cast on it, I won't be able to see my kids. So I said, I'll handle the pain. So I went and started treating it myself at home. After about a week or 10 days I started to be able to walk on it.

Riham: How did you treat it?

Participant: I brought—. He brought me an ointment from the pharmacy and a band. I tied it, and I sat for a while. He started helping me, and my son started helping me until I started to be able to walk on it. Yea, thank God, then it started to hurt me. At times, [Indistinct] on it a little. It would get better for a while, but when I would stand for too much it would start hurting me again. Yea, but now, it's hard. Now, here Ahmad has gotten a little bigger, so I struggle with it when I'm holding him especially when I'm bringing him downstairs, so I get really afraid that—.

Riham: You fall.

Participant: I'm suddenly not able to balance or something and fall with him. That's the only thing. And I—. They always bring him down. I don't bring him down

Time (7B) – 20:00

Participant: because of this thing, but sometimes I have to. But those are rare things, I mean. Most times, they bring him down.

Riham: Were you able to preserve [look after] your foot/leg here?

Participant: Now, when I went to treat it, I put the foot/leg that they gave, the plastic one. They told me for a month. Yea, I was able to put it on, but I didn't benefit, I mean. They said, “You need physical therapy.” But going to physical therapy—. It's hard for me to go to physical therapy every day or a few times every week. That's hard on me. So I said—. I left it. “With time it'll heal by itself.”

Riham: Okay. Why is it hard on you?

Participant: Now, it's hard when my husband is at work. There's no sometimes—. Like, he's going to start giving up more of his work to take me to get treated, and I'm not able to go out. Someone has to stay—. When I leave, someone has to stay with my kids, too. Like now, my son doesn't have school, but soon school is going to start. And treating nerves takes a long time. It could maybe take me months. Yea, so I managed to get by without the whole thing.

Riham: So that's it you managed to get by without your foot/leg? God be with it!

Participant: I said, “God willing, with the passing of time—.”

Riham: God willing.

Participant: The Master of all creation will heal me.

Riham: May God heal you.

Participant: May God protect you from harm.

Riham: God willing.

Participant: Thank God, and as for everything else there's nothing. It's true that—.

Riham: They got closer.

Participant: No, thank God, a month after I arrived I got better myself even without any treatment.

Riham: Thank God.

Ahmed: Thank God.

Participant: Yea, thank God.

Riham: Were you able to see a doctor when you arrived here and to treat yourself and your kids or did you find [have to wait] a long time?

Participant: Now—.

Riham: Not Ahmad. The other kids.

Participant: As for the kids, there was nothing wrong with them. I mean, necessary [things]. So, I left the appointments they scheduled. Like, I didn't need to do anything quickly.

Riham: I mean, how long did they stay—? Just give me an idea. Until they saw a doctor?

Participant: Maybe around two months. At first we got busy with Ahmad, and—.

Riham: Of course, because immediately—.

Participant: the hospital.

Riham: he was admitted to the hospital.

Participant: Yea, he was admitted. The appointments weren't scheduled quickly. Our appointments took a while until they started. And until now my kids have appointments for their teeth and their appointments aren't done yet. But there's nothing necessary. I mean, that needs to be quick.

Riham: What are the things that you feel are necessary to see doctors or the hospital?

Participant: For me or for my kids?

Riham: Both.

Participant: Now, the things—. A fever. I—. We get afraid of fevers. These are things that I can control, but when I see there's no results I can immediately think of the hospital and the doctor, with regards to kids. But as for me, I can manage. I can take pain. I'm the type of person that doesn't like seeing the doctor. Like, I don't go until it's absolutely necessary, and it becomes really hard.

Riham: You see? Two came to your house so that you'd go. You don't like us if we came in front of your face.

Participant: No, for me to go to a doctor—. I mean, I neglect the matter a lot. Like, this issue. Yea, and you're more than welcome.

Riham: Thank you. Thank you. Thank you very much for welcoming us into your home.

Participant: May God protect you from harm. You're more than welcome. Honestly, it's our pleasure.

Riham: May God preserve you.

Ahmed: A while ago, I remember Abu Mu'az said that Kroger is about an hour from here.

Participant: Yea.

Ahmed: Are you finding a problem buying food, securing suitable nourishment for your kids? Are you finding a problem, for example—. As you know, the food you find here is different from the food available in our country. So can you tell us a little bit about food and cooking?

Participant: Yea, now, as for when Mu'az went, he [Ahmad?] was in the hospital, and we didn't have a car. I needed some things, and I was too shy to ask from the community, so he told me, “I'll go.” So, he had to go, and we weren't expecting such a distance.

Riham: How sad.

Participant: Yea. But it's maybe the one time he went. Yea, other than that, thank God, but we get a little scared from the meat issue and the chicken. I mean, for chicken, until now I sometimes cook it, and the kids get disgusted. Like, we throw it away.

Riham: Why?

Participant: We can't eat it. Like, sometimes we get blood inside. No matter how much we clean it, you still find blood inside.

Riham: Do you find that the meat here differs from the meat you're used to?

Participant: Now, in Egypt and Syria, we didn't used to experience that. I mean, nothing like that used to happen. Like, here—. We started—. We've been lessening chicken dishes. We have been worried about it. But as for meat, we buy it from the halal stores, the Arabic stores, and don't worry about it, thank God.

Riham: When you buy the chicken from the halal stores, you find the same problem?

Participant: The same thing, yea, yes.

Riham: So you're decreasing the chicken then?

Participant: Yea, we're lessening. We get, for example, [chicken] breasts, but we don't get—.

Riham: A whole chicken.

Participant: A whole chicken or whatnot, yea, yes.

Riham: You find it's different.

Participant: Yea.

Riham: Are there things you used to always use in your kitchen to be able to keep your kids' nutrition really good that you aren't finding here? Either herbs or a food you're used to?

Participant: No, thank God. Everything is good.

Riham: You're finding everything.

Participant: Yea, yes. Like, what we don't find in Kroger and these stores, we're finding in the halal stores.

Riham: Okay. And where is the store you're going to? Is it far from you, the halal store you're talking about?

Participant: Yea, the one we go to is in West Chester.

Riham: Okay, West Chester.

Participant: I mean, about half an hour.

Riham: Half an hour.

Participant: Or there's “Jerusalem,” [the name of a store] too.

Riham: Yea.

Participant: We also go to it. It also carries it.

Riham: Is it nearby?

Participant: I don't know. Maybe it's about 20 minutes.

Participant's Husband: Twenty minutes.

Riham: Like, it's not walking distance.

Participant: No.

Participant's Husband: Twenty minutes by car.

Participant: By car. Yea, they're all far.

Riham: That's really different from your country.

Participant: Yea, yes, we were used to, for example, if I need something, I go down to the store across the street—,

Riham: Yea!

Participant: the supermarket across the street.

Riham: Glory be to God!

Participant: Yea, here, there's nothing like that. You have to enter—. I mean, you have to go on a special trip—.

Riham: For—.

Participant: for these here things.

Riham: Of course, of course. Do you have any ideas about how someone can improve the environment to—. Or the health for Syrian refugee kids?

Participant: Now, here, the environment is healthy compared to our country with regards to the greenery that's here. So, I don't think there's anything. I mean, I struggled with my kids, whether in Syria or Egypt. My son, Abdurrahman, also asthma, his chest and whatnot, allergies. Here, thank God, we didn't struggle with that.

Riham: That's not the first time I hear that. Ohio is known for having very, very high amount of allergens..

Participant: Yea, [Indistinct] that a lady came that was also in Egypt, and she told me that here she found—. I told her, “I'm the opposite. I got better.”

Riham: Glory be to God!

Ahmed: [Indistinct]

Riham: Everyone's body is different.

Participant: Yea, yes. My son used to take what they call an asthma spray [inhaler] in Egypt. Thank God, here he didn't need it.

Riham: Oh, thank God. I couldn't—. Sorry, were you able to treat him to see a doctor for asthma or anything or—?

Participant: No, here, he didn't need to see any doctor at all. I had brought with me—. I said—. Until—. Because they had said it would be a while until they were able to take them to see doctors, so I brought some medicine with me just in case.

Riham: Okay.

Participant: But, thank God, we didn't need it.

Riham: You didn't need it.

Participant: No, not at all.

Riham: Very good. Have you ever gone to the ER or needed to call an ambulance?

Participant: No, thank God.

Riham: Thank God. If you need to, do you know how to understand the system? How to call? How to get there?

Participant: Now, we know how to call. Uh... 911. Yea, and someone answers, and we ask for emergencies, ambulance, or the police. Yea, we can tell them—. If we just tell them what we need, they come to us at home. Even when we came, they had put a piece of paper in every room with the emergency number on it.

Riham: Oh, very nice. So, 911 so you don't forget it?

Participant: Yea, yes. At that time they had told us, “Whatever happens with you, immediately call it.”

Riham: Okay. But you never needed it?

Participant: No, thank God.

Riham: Thank God. Thank God.

Ahmed: Let's return a little bit to Ahmad. With regards to medicine, was there something you were expecting to find here in Cincinnati and didn't?

Participant: Yea, now, we were expecting to find him a cure and didn't.

Ahmed: Okay.

Participant: Yea, but, thank the Master of all creation, that's what the Master of all creation willed for him, destined for him, and we have hope that, God willing, the Master of all creation will destine healing for him—

Ahmed: God willing.

Participant: and—.

Riham: God willing.

Participant: a doctor will discover the cure, God willing.

Ahmed: God willing.

Riham: God willing.

Ahmed: In your opinion, is there something you were promised and didn't find anything—. For example, did you know that Catholic Charities would only take care of you for three months? [Indistinct]

Participant: Yea, yes. They had told us. Yea, from when we first arrived they told us.

Ahmed: Okay.

Participant: Even over there, they did an educational session and told us that—.

Riham: When you were in Egypt?

Participant: Yea.

Ahmed: Okay. So did you feel you were ready/prepared when you arrived here? For the things you faced?

Participant: Yea, we had almost [completely] known things [what to expect].

Ahmed: Okay.

Participant: I mean, even the Syrians that would leave [for America] would communicate with us and let us know

Time (7B) – 30:00

Participant: what would happen to them.

Riham: that—.

Participant: That's something that helped us a lot.

Riham: That they did this and did that? And if you want such and such do such and such?

Participant: Exactly. Yea, they used to tell us and give us directions.

Ahmed: Okay.

Participant: How when someone first arrives what happens to them—.

Riham: [Indistinct] like what?

Participant: They used to inform us about everything.

Riham: What is something you heard about that helped you, for example? Anything.

Participant: Now, when they told us that the organization is going to receive you, and it's responsible for you. It finds you housing and furnishes it for you and stays with you for—. It's three months in some states and in some states—. Like, every state is different. In America every state provides different aid, and its laws are different. Like, every state differs from the other. We had heard about all of those things. [Indistinct] that the Arab communities help, too. They also had told us about that.

Riham: Do you have friends in the United States?

Participant: Yea, the Syrians here that got togther. We all—.

Riham: No, like, didn't you say, for example, the people who are telling you these things. Don't you know them from back home?

Participant: Now, those people, no. When they told us “[you're going] to America,” they started making WhatsApp groups—.

Riham: Oh, really nice!

Participant: [Indistinct] and start adding each other.

Riham: Really nice!

Participant: And they would provide information. Like, whoever is in this place would provide information to someone in Egypt or—.

Riham: So we can all get together.

Participant: Yea, not to get together. To give information so that the person leaving [to America] can know [what to expect].

Riham: Yea, yea, yea.

Participant: Because the one leaving is leaving without knowing—.

Riham: Of course.

Participant: anything about the country.

Riham: Yea, because the most difficult thing is for someone to reach a situation without being prepared for it, without knowing anything [about it], or thinking that its something and sees that its something else [Indistinct]—.

Participant: Yea, that's true. We were prepared for this. We had heard a lot of things.

Riham: Thank God. I [Indistinct]—.

Participant: [Indistinct] helped us, too.

Riham: I haven't heard that from any family. This is the first time I hear this, but that maybe helped you a lot.

Participant: Yea, yea. Yea, yea. That helped us with a lot of things.

Riham: I mean, a whole bunch because when a person doesn't know where they're going it's something else.

Participant: Yea, that's right. And here, too, the organization's people—I mean, the case worker—may God reward him, he helped us a lot, too. In addition. I mean, some people really struggled [working] with him [or “suffered because of him”], but we, thank God, didn't struggle. He used to move everything forward for us.

Riham: Is it the same exact person or does everyone have a different—. Does every family have a different person?

Participant: No, not every family. Every few people has [one]. Now, we Syrians here don't all have the same person.

Riham: Okay.

Participant: There are some that struggled. Even here there are some that struggled with [them/him]. No, but we, thank God, didn't.

Riham: But not from the same person.

Participant: No, not from the same person. He was good to everybody.

Riham: Okay.

Participant: Like, all whatnot we heard that he was good.

Riham: That's really good. Did you get to know people really well in the organization? Don't they do classes or something like that?

Participant: I didn't attend the organization's classes.

Riham: Because of your son.

Participant: Exactly. I didn't go. I didn't even go to the organization at all. If there was anything he wanted from me he used to come here, and I would sign the papers and everything.

Riham: Did they give you any trouble? Because I heard about people that would get threatened to have their food stamps and whatnot cut if they didn't go to the organization.

Participant: No, our case worker is the one who took responsibility for that, and he was the one who told them about our situation and helped us with this thing. And they didn't—.

Participant's Husband: The food stamp people decided not to take it away because she has a situation [special circumstances].

Riham: Yea, of course.

Participant's Husband: Like, as you said, they reduce [the benefits] for a person who doesn't go.

Riham: They reduce whatever [amount] it was.

Participant's Husband: But for us, in the beginning they told us she doesn't have to attend because she has a circumstance. She has Aisheh, and she has Ahmad.

Riham: Of course.

Participant: The food stamp people even gave us an interview, and they told my husband, “You have to work and whatnot” but not for me.

Participant's Husband: They even told me, “You don't have to [it's not a condition] that you [Indistinct]. Three days are enough.” They also know that I have a lot of appointments.

Riham: Of course. You have to drive and take them.

Participant's Husband: I have to take them and bring them back. They told me [Indistinct] three days in the organization it's enough. And if it weren't for the language [not knowing English] and I were able, I would do four or five days.

Riham: God willing.

Participant's Husband: And once they start working, there's no need for them to to go. They just have to tell them “I worked.” So I told them “I worked”—because they reduce our food stamps and whatnot—.

Riham: Your husband told me that they suggested [to him] bringing a nurse to help you at home. What do you think about that?

Participant: I refused—.

Riham: [Indistinct]

Participant: [Indistinct] I didn't want to—.

Riham: I know.

Participant: I mean, I didn't want to—. I'd feel like I abandoned the son that I came [to America] for—. I'm finding someone to take care of my son, and I leave him to, for example, go out, learn—. I mean, I didn't like that. I wanted to—. I mean, I wanted to see him myself. Even when he was still aware [neurologically sound], he was really attached to us, so I wanted to pay attention to the thing that he likes. I don't know if he senses us or not, but I sit down and talk with him. I act like he knows me and senses me. At times I even feel—. I feel his breath is like he's not comfortable. When I come and sit down next to him to talk, I feel like he calms down. I mean, sometimes I feel like his feelings are with me.

Riham: Glory be to God.

Participant: So I don't like to leave him. Even when I do leave him and go a long time he's still on my mind. Even one time—. One time when he wanted to take us to Michigan, I came and told the nurses, “Does traveling affect him [negatively]?” They said, “You can leave him with us for the time you're away.” I told them, “No.” I didn't accept that.

Riham: Even if it's only one day?

Participant: Yea, yes. I didn't accept that. They said, “We know you're not going to accept, but we said we'll still offer.”

Ahmed: [Indistinct]

Participant: But it's hard. Like, I don't like to be away from him.

Riham: Do you find it's easy to communicate with the nurses that come and help you?

Participant: Yea. They bring a female interpreter with them and whatnot, and they sit down and talk with me and everything. Thank God, they really take care of him, thank God.

Riham: Did you find anything you wish would change or improve? For example, your husband told me that sometimes they bring you young men—. I mean, male nurses or male interpreters.

Participant: Yea, they used to bring a male interpreter. When they bring an interpreter, it becomes necessary for me to talk, and I can't talk a lot and—. Like, I can't be comfortable speaking with him when it's a male interpreter. When it's a female interpreter, we talk and laugh and—. They would ask me about things, ask me about my situation. I mean, I felt like we were like friends.

Riham: That's nice.

Participant: Yea, yes.

Riham: You're more relaxed.

Participant: Yea, and being that I don't see a lot of people, I feel like I spoke a little and—.

Riham: You had a good time.

Participant: I eased up a little bit.

Riham: Yea, that's really nice.

Participant: Yea, like I relaxed a little.

Riham: So, how did you solve the issue?

Participant: Honestly, my husband told them that we're bothered [not comfortable] and to no longer bring a male interpreter. So the last time when she was with us the interpreter was via mobile phone. When they came over.

Riham: Do you find any problems with the interpreter via mobile phone?

Participant: No. They even asked me, “If you're not comfortable or anything, we won't bring it anymore.” I told them, “No, it's okay. It's not a problem [literally, “does not affect”].”

Riham: Good, thank God. Is their dialect easy [to understand] and all that?

Participant: Yea, yes. If I don't understand anything—. If I didn't hear or anything, they even go back and repeat it for me. Like, it's not a problem.

Riham: That's really good.

Participant: Yea.

Ahmed: Is there anything you have in mind, good or bad, that we haven't mentioned yet? That we haven't spoken about? That you want to talk to us about?

Participant: No.

Ahmed: We said that you never had to go to the ER, right?

Participant: No.

Ahmed: What are your expectations—. What do you expect from the ER if you go to it?

Participant: Frankly, I never thought about it.

Ahmed: Okay.

Participant: But I expect that it has good service. Like, based on what I see with regards to care and whatnot. I expect that it's something good, I mean.

Riham: So, what do you see [literally, “in your eyes”] as good service?

Participant: That they take care of the patient.

Riham: Okay, what does taking care of the patient mean according to you [literally, “in your eyes”], in your view?

Participant: Taking care means no matter what level they've reached [meaning, “no matter how bad the patient's condition is”] that they don't neglect them. There's no neglect. They see their condition, no matter how bad it is and how impossible it is. They are still humane.

Riham: How can a doctor show you they are humane? Or the nurse? Or the—?

Participant: Taking care—. Frankly, in Egypt I really struggled with this in their hospitals. Like, my son was, for example, that needle for the nutritive substance [IV] would come out—. The one they put for him to give him medications.

Riham: Yea.

Participant: So they used to struggle to find the vein.

Riham: Yea.

Participant: So when it used to close up or would come out, the period for his treatment would be missed. The first time would be missed then the second time, and they would neglect him out of laziness because they would sit

Time (7B) – 40:00

Participant: and wait for a nurse or doctor to put it for him. And they had him on the third floor. Even the doctors at times would be too lazy to come upstairs to us. So I would go downstairs, go upstairs, go out, and call them and only with great difficulty would they answer me. So that thing really made me weary. Even one time I was in a room full of kids, and the doctor entered and said—. I would wait every day for the doctor to give me an update on my son's condition. She entered and examined all of the kids with the other doctor. She came to my son and said, “What's this kid's condition?” She said, “No, no. Leave him, and come here.” Like, she [Indistinct]—.

Riham: [Indistinct] You understood her.

Participant: Yea, she's Egyptian.

Riham: What did you tell her after that?

Participant: I—. I—. Frankly, I was shocked. I sat down and started crying. I mean, I saw my son's condition like that, that they were leaving him [neglecting him] that much, but that thing used to affect me, too. It makes my own psychology tired [emotionally exhausted] …and my presence with him. So I didn't find that here, thank God. That's something I really struggled with in Egypt. I would see my son with a fever, and I couldn't do anything about it. It was out of my hands to do anything. I couldn't do anything. Only the doctor could do something about it. That's the thing that really made us most exhausted. Here, when we see that they care about him so much, we found a huge difference.

Riham: Thank God.

Ahmed: Thank God.

Riham: When you see that they're checking up on him a lot, when you see them a lot [literally, their faces in front of your face], when you see them taking care of him—I mean, examining him and touching him—those are the things that make you feel that they are taking care of him?

Participant: Yes, yes. They even, if they see him with an allergic reaction, they say, “When did this allergic reaction start?” Anything, I mean, if they see something on the skin of his hand, they immediately tell me. They tell me, “He needs such and such ointment.” Like they—.

Riham: They're precise or detailed?

Participant: Exactly, the last time during physical therapy she found something red on his hand and said, “What's this?” I said, “His skin is peeling.” She said, “Tell them to prescribe an ointment for him when they come to you.”

Riham: Were they able to prescribe one for him?

Participant: Yes, yes. They had already prescribed an ointment for him. I asked them about it, and they said, “Yea, you can use it.” I thought it was only for his face, but, no, they said, “You can use it for his body, too.” Even when we first came he was struggling with—. Like, he's always wearing diapers and whatnot. He was struggling with his skin peeling [diaper rash?]. Yea, but here, thank God, they were able to treat it. Here, they prescribed the necessary ointment. Over there, in Egypt, they don't tell me. I go out and start trying out different ointments. I try this ointment. I try that ointment. Yea, we really used to struggle with that thing. Here, immediately. In Egypt, he reached a condition where all the bones of his body were protruding because he got thin [or lost weight] that much because he was laying down so much so the place where the bones are would become red.

Riham: Decubitus ulcer.

Participant: Yea, yea. Here, they immediately put an ointment, put him a wrap bandage. And when we went home, they immediately sent the ointment and wrap bandage with him. Every time they come, they sit down and ask me, “Are there any areas? Is there any redness on his body?”

Riham: Did they teach you how to move him [flip him over] every little bit?

Participant: Yes, they taught me, and they even kept telling me that they're going to bring him a bed. I tell them, “The bed is big. I want something smaller.” But they said they only have big beds. So, in the end, they brought me a big bed. I put it upstairs for him.

Riham: Good.

Participant: Because they started telling me that he has to sit for a little bit for “rabakeh” [indistinct]. So we used to struggle a little bit with his sitting down, but now with the bed it's easier on us.

Riham: Yea, it seems that he has gained weight since the last time I saw him.

Participant: Yes, when we first came he was about 15 kilos. When we first came. And now, the last time we went, they told us he's 23.

Riham: Yea, wow, did you change the food or what?

Participant: They prescribed a special food for him. In Egypt, when we were with him—. When they used to give him the syringe, I used to ask them, “What's the food?” They used to say, “The same food you eat. Just blend [literally, “ground”] it for him.” So, I used to boil vegetables for him and make fruits and give it to him.

Riham: How did you used to give it to him? With the syringe?

Participant: There was a time where he used to use the syringe. So, we used to give it to him with the syringe. After a while, we felt that he started swallowing, so we took it out, and we started to give it to him by bottle.

Riham: Okay, by bottle.

Participant: Yea, but when we came here, they did an exam and found that it was also entering his lungs, so they went back and put him the syringe. And now we give the nutrition through the syringe. They prescribed a specific nutrition for him.

Riham: For him?

Participant: Yea.

Participant's Husband: Pediasure.

Riham: Oh, Pedialyte?

Participant's Husband: Pediasure.

Riham: Oh, Pediasure? Pediasure.

Participant: But now Pediasure is a little bit heavy on his stomach, so now they started giving him Pedialyte and Pediasure.

Riham: Oh, okay, okay. Both, you mean.

Participant: Yea.

Riham: When you're asking questions or want to learn more about his condition or his sickness, how do you learn more about it? Or how do you feel that you have—? Or do you feel that you have the information you need?

Participant: Yea, they told us how we should deal with him. They made us aware [literally, “awake”] of the situation. And they told us, “If anything is difficult for you, ask us immediately.”

Riham: So you don't feel you have questions that aren't being answered.

Participant: No, no. They immediately answer anything we ask.

Riham: Thank God.

Ahmed: I have a question about your son. In your opinion, what are the needs of Syrian refugees? What are the things we have to do that nobody else is doing?

Participant: Honestly, I don't know a lot because I haven't mingled [or “interacted”] with people a lot, but I didn't hear that anybody needed anything that wasn't secured for them. Like, for example, for me, if an organization can't secure something for me, the community does. Like, it's helping them a lot. So, I don't know a lot about things like that.

Riham: The question is because—. Just like how I told you the study is trying to find what the things are that we can solve. So, is there anything in your mind that you think can improve the situation for children here? Whether anything about schools or healthcare or doctors or anything?

Participant: Yea, honestly, I didn't struggle with these things, so I don't what the things are that they struggle with. I didn't hear from anybody except that family whose son became paralyzed. The mom said her son became paralyzed and improved, and the doctors didn't even offer one pill of medication. Like, her son got better by himself. But I told her, “I think that's really good” that her son didn't even need a pill of medication, but as a mom she wants to see a concern for her son because I went through that phase.

Riham: Of course, of course, that's why I'm asking you what is “being concerned” for you because everybody has a different point of view. Like, for example, sometimes someone sees concern as something different than another person.

Participant: Yea, yea.

Riham: Do you know what I mean?

Participant: Yea.

Riham: Like, someone can say, “I always want to see someone [literally, “someone's face”].” The other, “I want a pill.”

Participant: Yea. With regards to medicine and whatnot, I prefer that someone stays away from these things. If it's possible to do something other than medicine that's my preference because my son took a lot of medicine, and there wasn't a need for a lot of the medication he took. I found that that was something that was unnecessary. Even now, sometimes, for example, they give him a medication. I ask if it's possible to substitute it for herbs [meaning, “a natural remedy”] then that's better. Whenever he has the flu or constipation or whatnot, that's what I prefer.

Riham: What are the herbs that you know that help these things. Give me an idea.

Participant: With regards to my son, chamomile. They used to tell me about it when I was in Egypt because I used to struggle a lot [with dealing with it for my son]. With regards to constipation, they used to tell me chamomile or orange juice. And the last thing they told me was “shawandar.”

Riham: Oh really? “Shawandar” is a radish?

Ahmed: No, sweetbeets that they make sugar out of.

Riham: That they make pickles out of?

Ahmed: That's turnips. “Lift” is turnips.

Riham: Red.

Participant's Husband: The one that gives color.

Ahmed: But it's not a turnip.

Riham: Yea, it is.

Ahmed: A turnip is—. Okay, okay, but it's okay.

Participant's Husband: Yea, yea.

Riham: But it's not spicy.

[Translator's Note: “Shawandar” is a beetroot. “Lift” is a turnip.]

Time (7B) – 50:00

Riham: “Shawandar” is sweet. “Shawandar” is sweet, right?

Participant's Husband: Yea, yea.

Riham: Oh, it's not spicy?

Ahmed: “Lift” is spicy.

Participant's Husband: “Lift” is the pickled one. “Shawandar” is what is put on “lift” to dye it.

Ahmed: I forgot what “shawandar” is in English.

Riham: Maybe we don't have it in America.

Participant: No, no. It's there because we bought it.

Riham: Show it to me later.

Ahmed: I'll show it to you.

Riham: What else? Chamomile, beets, what else?

Participant: Oranges. These things. That's what I used to use. Those things. Herbs with regards to the chest, coughing. We also use herbs.

Riham: Very good.

Participant: Yea, I used it a lot for my kids there because I used to struggle since they didn't use to give him medicine. He even used to start coughing with mucus. It used to stay in his chest, and he wasn't able to cough and take it out.

Riham: Of course.

Participant: So, I used to give him things that would take it out for him—

Riham: That would help him?

Participant: Yea.

*Husband brings in vegetable*

Riham: Isn't that a turnip in English?

Participant's Husband: Not “lift.” This is sweet.

Ahmed: This is sweet.

Participant's Husband: There are some people who boil it and eat it like this. It tastes sweet.

Riham: Maybe the one that I know is long?

Ahmed: It looks just like that, but the other one is red.

Riham: Oh.

Participant: This is red.

Participant's Husband: It's really dark.

Ahmed: But this one [indistinct].

Riham: That's the one I know. That's something else?

Participant's Husband: This is “lift.”

Participant: They look similar, but the other one is white. This one is red.

Riham: And you can find this in the store?

Participant's Husband: What you're referring to as “lift” is white inside.

Riham: Sugarbeet!

Ahmed: Sugarbeet.

Riham: Do you know why we don't have it? Because we don't have it in Michigan.

Ahmed: Oh.

Riham: This is known in the south.

Participant's Husband: We have it here.

Riham: But I haven't seen it.

Participant's Husband: Now “shawandar”—. “Lift” is made into pickles. Restaurants and whatnot put a dye with it. It's good.

Riham: My dad makes pickles. I know it. He puts turnip. What is it in Arabic? “Lift.” Because we don't have from this.

Participant's Husband: “Lift” is pickles, but factories and whatnot make it red. It's white. “Lift” is white. They put a dye with it.

Ahmed: A dye.

Riham: So it becomes—.

Participant's Husband: But if you put “shawandar,” it gives its true color [red]. This is just a dye, and the same one also becomes pickles, and it tastes good.

Riham: And what does this help with [“shawandar” or beets]?

Participant's Husband: It dyes.

Participant: It's good for anemia, for constipation.

Participant's Husband: For constipation.

Riham: For constipation.

Participant: We boil it and then blend it [literally, “ground it”] then give it to him.

Participant's Husband: His basic nutrition is milk and dates. We boil the dates for him and then we take the date juice and mix it with milk. That was his main meal. In my view, this food is what used to help him.

Riham: Yea, because it has sugar in it.

Participant's Husband: Because dates have complete vitamins.

Riham: A lot.

Participant's Husband: All of it. Completely, I mean. Now, I went on a diet here for about a month. I was just eating dates and drinking milk.

Riham: Wow.

Participant's Husband: Yea, I mean, sometimes I used to eat a few salad, but milk and dates are enough to not need anything else.

Riham: Glory be to God.

Participant's Husband: A lady came from the organization, and I said, “What should I eat so that it's everything [has enough nutrients]?” I said, “How about eating dates?” She said, “You need milk, too.” I said, “What about dates and milk?” She said, “Nothing will happen to him.”

[Translator's Note: It is not clear if she was quizzing him, asking about his son, or himself.]

Riham: Glory be to God.

Participant's Husband: This is the lady responsible for nutrition. We know that dates and milk—.

Riham: It's known.

Participant's Husband: We have a story for it—.

Riham: They used to break their fast on it.

Participant's Husband: We have a story about it, if you want to hear it.

Riham: Yea, yea, of course.

Participant's Husband: There was a guy—. My maternal aunt's husband used to always tell us this story, God have mercy on him. He died.

Riham: God have mercy on him.

Participant's Husband: He said there was a king a long time ago. If he wanted to kill people, as in execute them, they used to be given a choice. How he used to execute them was that he used to say, “What food do you like?” For example, they used to say, “I like beans.” So he would say, “Feed him beans every single day.” After a month or so, he would die. After a few days, a week, that's it because the stomach is not getting all the types of vitamin or whatnot. So a person would die because he doesn't have enough sugars or whatnot based on what he likes. He would go and get someone else, and every time he would get someone else he would say, “What food do you like?” He would say, “I like eggs,” for example, so they would keep feeding him eggs until he died, no matter who it was. This was until someone came, and he asked him, “What do you like?” He said, “I like dates and milk.” So a month, two months, a year, two years went by, and the guy wouldn't die! It's a strange story I wanted to tell you that dates—. And the Messenger of God, peace and blessings be upon him, said, “A house with no dates in it has a hungry family.” Glory be to God. We always hear, as Arabs, that the West has figured it out—. That dates have all vitamins, starchy foodstuffs, and proteins—. All of it is in dates.

Riham: Yea.

Participant's Husband: The Messenger of God, peace and blessings be upon him—.

Riham: Peace and blessings be upon him.

Participant's Husband: said, “Eat in odd numbers.” And they found out that if you eat odd numbers [of dates, for example] you get a complete nutrition—. It gets balanced out. If you add one, it gets unbalanced. Add another one, then it gets balanced again. You get a complete nutrition from dates.

Riham: Glory be to God.

Ahmed: Glory be to God.

Participant's Husband: I want to say something with regards to schools. The older one is smart. In Syria, he would be at the top of his class. Over there it was easier for him. We went to Egypt, and again he was working energetically and actively again, but here he tells me at times in school, “We sit down a lot.” And he says, “I feel bored.” So, I'm afraid that he's going to regress here in his studies. So, we're asking other people. We put him in Withrow [spelling?] School. So, I'm asking other people, and they're saying that, for example, their son is in 10^th^ grade, but he's taking the 11^th^ grade curriculum.

Riham: Yea.

Participant's Husband: I want to find out what the ways are, if there are any sort of ways to—. How can I help my son? He displays energy with his studies, and he's just sitting there. He says, “Dad, I answered all the problems and just sit there.” I tell him, “Look, son, ask how you can move forward [progress].” Now, in Syria we know how it is. In Egypt, we know how it is. Here, I'm really struggling with this. So, if there's any way you can tell us how or something like so my son doesn't regress—. Because this thing causes regression. I want him to feel full of energy and study. He even told me, “Dad, I want to see if there's another school.” I don't even know if they let us go to another school or what.

Riham: You mean transfer to another school?

Participant's Husband: Because maybe this is just for refugees because everyone who goes there is a foreigner. Like, it has—.

Riham: To tell you the truth, I don't really know a lot about schools because Ahmed and I aren't from this area. We don't know the schools really well. In America, by law, it's according to your address, so if you're in an area based on your address they put you in a school, but it seems like certain schools are being specified for refugees so that they can bring certain help.

Participant's Husband: For the language. It's because they focus on the language. We don't have a problem with that, but if there are ways for us to learn how he can improve or move forward—.

Ahmed: Have you tried to ask the caseworker? Honestly, I asked him, and he said, “They're all the same.” Does he mean “all of them” as in all the schools or all the schools [we, as refugees] have to go to? Because they told us not all schools are the same.

Riham: No, they're not all the same.

Participant's Husband: For sure he probably means all the schools we have to go to.

Riham: So did you speak with the school itself? Were you able to, for example, meet with the teachers?

Participant's Husband: No, no, honestly, I wasn't able to.

Riham: Or principal to tell them, “I'm finding out my son isn't studying a lot. It seems like he needs to be challenged.”

Participant's Husband: Honestly, I'm waiting. If it continues, I'm going to go tell them. One of my friends told me, “You have to tell them, and they give him more advanced programs [study].” Because it's wrong for him to waste his time like that. He wants to learn. And they're sending me mail—.

Ahmed: Thank you. We're going to stop the recording. Okay?

Interview 8A

Riham: Today is August 15, 2017. I am with a young Syrian man who has three children. They’ve been here for about a year and two months and we’re going to start right now, God willing.

Participant: Hello.

Riham: How are you? How is everything going?

Participant: Thank God, everything is gong well.

Riham: We spoke a little about this topic before we started the recorder. Lets begin right now.

Participant: Go ahead.

Riham: Okay. I’m going to start by asking you how you feel your health and your family's health is controlled here in Cincinnati after arriving from the country which you came from.

Participant: Thank God, our health is good and thank God, Master of all creation, and it’s just that we have a few problems.

Riham: Okay.

Participant: What kinds of problems do we have? How can I tell you? For example, doctor’s appointments.

Riham: Tell me a little bit about doctor’s appointments. What kinds of problems do you have?

‘

Participant: For example, with regards to doctors appointments, some days like for example, yesterday I didn't take the young one, as in my young daughter to the doctor.

Riham: Why?

Participant: Because her mom was at work and I had work. Yeah, so who’s she going to go with and who’s she going to come back with?

Riham: Who made the appointment for her?

Participant: They’re the ones who made the appointment or her.

Riham: They? I’m sorry, please explain.

Participant: The clinic in [Indistinct]. So we didn't take her. And one time they referred us to a dentist in a hospital.

Riham: Cincinnati Children's Hospital, right?

Participant: Yeah, Cincinnati Children's.

Riham: Okay.

Participant: They referred us there for her teeth.

Riham: yes.

Participant: Yeah, so maybe we went maybe we didn't go, I don't remember. We went or we did not go and from the hospital they referred us to a clinic. They said the hospital doesn’t fix up teeth or something.

Riham: Maybe they sent you to someone who specializes in fixing teeth or something.

Participant: She had a tooth that was eaten up from the inside and reached the skin.

Riham: Oh, a root canal!

Participant: Yeah so they said they took it out.

Riham: Did they put her give her a new tooth on top of the old one?

Participant: No, thank God it stayed in its place.

Riham: Thank God. Okay.

Participant: They just took it out. After they took it out she said that she got a new tooth.

Riham: Oh, that’s really good. That’s your daughter that’s 11 years old?

Participant: My daughter that’s 11 years old.

Riham: Okay.

Participant: As for me, I am also struggling with the dentist.

Riham: Okay, so tell me about your struggle.

Participant: My struggle is that every time I go there, like for example, right now they took out my molar teeth. may God grant them safety, but then when they went to put them back in they told me they cant do it, because my insurance hasn’t kicked in yet. “You still don’t have permission from the insurance company”, they said..Like from inside here and here, do you see? There are no teeth. There are no molar teeth.

Riham: Did you have this problem with your teeth back home?

Participant: Yeah I came from my country with problems with my teeth. Yeah and I came here, and they referred me to a dentist. And he said this tooth has to go. He took it out. They took out five or six teeth, molar teeth, and then we started sending to the insurance company to put the teeth back in. Until now it’s been like six, or seven, or eight months and nothing.

Riham: Do you have health insurance?

Participant: I have health insurance but it doesn’t cover it.

Riham: You have to pay out of pocket?

Participant: I have to pay out of pocket?

Riham: And it’s expensive for you.

Participant: It’s really expensive. It’s really really expensive. I came from my country here with only 400 dollars. I came from Jordan to here and all I had was 400 dollars.

Riham: Yeah.

Participant: We came here and they rented a house out for us. They said it’s ready. They paid for “three months of rent. After three months they said “you take care of yourself”. So I started working and started paying for the house and paying for this.

Riham: Did you find any problems when you started looking for work?

Participant: Of course!

Riham: Did you find anything pleasing to you?

Participant: I found a lot of problems especially the language.

Riham: Okay.

Participant: The language. Now if I knew the language I could’ve been a driver for Lyft. I could’ve –. In our country we have this job where someone carries stuff.

Riham: Oh, you mean you drive around and make deliveries for things?

Participant: Yeah. It’s called “shoki”.

Riham: You mean using a forklift?

Participant: Yeah, exactly. If I knew the language, I could’ve done that.

Riham: Does that need a license?

Participant: I have a license.

Riham: Do you need to know the language?

Participant: You have to know the language. Now from Cincinnati, I got a license. Let me show it to you. They took me –. I told the organization that I’m a driver. They told me before we can line up some work for you we want to see.

Riham: That you do this for a living.

Participant: That I can work or no. This is the license.

Riham: The forklift workshop application.

Participant: When I went to this school –.

Riham: Yeah?

Participant: I go inside, yeah, inside they gave me an interpreter, may God reward them. They said, “do you know how to drive it?” I said “I know how to drive it.” They said “lets try you out and if you don't know we’re going to teach you”. I told them okay. They take by the hour. But they told me if you know how to drive it we wont take anything from you. We told them okay. Honestly, I got inside and I took it around and they said,“pick these up and put them over there and pick those up and put them over there.” After two or three nights they said “we want to make you a trainer for us.” For you to train people, as a teacher. Well I’ve had experience training people, syrian people. Ahmad Masla. I trained Ahmad Masla and two from Sudan. Two people from Sudan.

Riham: You mean refugees?

Participant: Yeah refugees. Because they told me you don't need a translator. Do you see what I mean? The interpreter said there is no need for me. They speak Arabic so you train them.

Riham: So they can work.

Participant: So they can work. I kept training them about 15 [Indistinct]. The company told him hey Mohammad, we will hire you but you need to know the language. Because they put the radio.

Riham: So they can speak with each other.

Participant: So they can speak with each other. For example, if they said, bring me this drum or anything or lift this refrigerator or what not and take it to that car or whatever. What. He’s not going to understand them. So I went and applied to another company. They also told me the most important thing is the language. I’ve had [Indistinct] work a lot where I am working now I also applied to get the license and they agreed. I went inside to the supervisor. He is sitting and talking to me and I don't understand him. I didn't understand what he was saying. So he said, “how am I going to hire you?”

Riham: So what do you do now?

Participant: Right now I work –. I fill up boxes with nutritious items.

Riham: I understand.

Participant: Yeah.

Riham: Tell me a little bit –. In the beginning you were talking about appointments. Let’s go back to that.

Participant: Okay.

Riham: The appointments they gave you, for example, your daughter’s appointment. You missed it yesterday. Okay, did you know that there was an appointment?

Participant: Yeah I knew. Yeah honestly.

Riham: How did you know?

Participant: Because they sent me a letter with the appointment on it.

Riham: Okay, but you don't understand English but you were able to understand.

Participant: Hey bring the paper so I can show you the paper. Give me the paper.

Riham: Just talk to me about the paper. Explain it to me. That you saw the paper –.

Participant: I saw the paper and I showed it to a lady. Now when I get an envelope in the mail, I don't open it. I wait until I find someone who can translate to Arabic from English.

Riham: Oh.

Participant: I give them the envelope, I have them open it and read it to me and tell me you have such and such and you have such and such and you have such and such. For example, yesterday I got some mail.

Riham: Yeah?

Participant: I didn't open it or anything. I'm waiting for someone to come and translate it for me. Look here. I don't know where this mail came to me from anyways.

Riham: Okay.

Participant: Show it to her. Now any mail that comes to me I don't open it, I keep it sealed.

Riham: Until someone comes.

Participant: Until someone comes and translates it for me.

Riham: So for medical issues, who can help you translate those?

Participant: There is a lady called Heyoum. They call her Umm Fahid, may God Reward her. Every little bit, like every Friday she calls me. She calls Umm Maher, my wife, and speaks with her. And my wife says we received a few letters in the mail so you can read them for us. So she says hey on such and such a day, I have time. So she stops by in the afternoon, reads them for us, translates them for us, and may God send His Peace and Blessings upon the Prophet, she –. Like for example, those papers over there have to do with the plane ticket. Like for the plane ticket, every single month, I spend 100 dollars. Do you know what I mean? So the other month I didn't pay for the plane ticket. Why? Because you need someone to help you write down the amount and where you’re sending it and where you’re –.

Riham: The check.

Participant: Yeah. So I missed it.

Riham: So in those times that lady who’s helping you, is she from the organization or –?

Participant: No, no. She was –. I got to know her here in the field of interpretation. She was going with us to interpret. Do you know what I mean? We had a doctor’s appointment so they would call the lady and she would go and interpret. So that’s her job and that’s how we got to know her and the lady is a kind a respectable person, and she serves others. May God reward her immensely. There wasn't a day that I called her except that she responded and helped me. But you can’t always call her. Why not? Because she has work.

Riham: Of course, of course.

Participant: But today I called her, I do not remember what I needed from her, do you know what I mean? And she called me back two hours later. She told me “I can’t, I have some people in the hospital that I am interpreting for so I can’t answer right now, I’ll call you back later.” I mean, may God reward her.

Riham: When you go to the doctor’s appointments, do they help you with interpreting?

Participant: There is an interpreter with us.

Riham: Always?

Participant: They find an interpreter for us.

Riham: So you do not find any difficulties with translation when you are in front of the doctor.

Participant: No, because there is an interpreter there. When there is an interpreter there, there are no problems.

Riham: When are the greatest problems you face with translation? By phone or by –.

Participant: By phone and with the mail and for example, when someone talks to me by phone. I don't understand him. I tell him “no English, speak Arabic”.

Riham: And then?

Participant: He closes the phone and says thank you.

Riham: Do they call you back?

Participant: They don't call me back.

Riham: So you don’t know what it’s about.

Participant: No, I don't know what it’s about. So the other week we got some cards and we had applied to the Green Card, right? So we got some cards in the mail and so I asked her to translate it for us and it was about the Green Card that our application had arrived and they were looking into it. There are a lot of offices that are useless, good for nothing, you go there and the employee doesn't know anything and isn't free to help you.

Riham: Yeah that’s hard

Participant: That’s really hard. It’s really hard.

Riham: So, bills so –.

Participant: With everything, with everything. Like maybe a few days later she tells you I am traveling. So you call them and they say hey I am going to travel for one or two weeks. Yeah what are you going to do, you’re going to leave the mail for two weeks waiting for someone to come and translate it for you?

Riham: Of course. So do you use any machine or device to help you translate?

Participant: No.

Riham: Like the phone that has –.

Participant: We use –.

Participant's Wife: Yeah, but it makes mistakes.

Participant: Yeah it makes mistakes.

Riham: When it says words it comes out all mixed up.

Participant: Yeah.

Riham: So you tried to do that before?

Participant: Yeah.

Riham: For example, the health problems among Syrian refugees and also in your family. Let’s start with your family. So if we were to say what are the biggest health problems that were seeing in your family. So you mentioned teeth.

Participant: Other than teeth, my wife is going to a doctor, a woman’s doctor [so that would be a gynecologist]. So she’s been going to this doctor for five or six months and he gives her medication and he tells her this medicine is going to help you get pregnant.

Riham: She’s trying to get pregnant?

Participant: Yeah and she’s not benefiting at all. And I don't know and some of them say your blood sugar is too high that’s why you can’t get pregnant and others say your blood pressure is too high so you can’t get pregnant and thats also a problem were facing. No, I want kids. That means I want kids. I mean I am planning on getting one or two more kids, God Willing.

Riham: Not everything is in our hands, right?

Participant: It’s in the hands of God.

Riham: Exactly.

Participant: In the hands of God.

Riham: So tell me about this problem. Of course I am going to talk to her but from your point of view your wife found problems when searching for a gynecologist or when searching for the medication she was looking for?

Participant: Yeah the problem is the –. So I look for the slides, the sugar slides, and sometimes I go to the pharmacy and they don't give it to me.

Riham: Oh, you mean test strips? Those are really expensive.

Participant: Those little strips for the sugar?

Riham: Yeah, they’re really expensive.

Participant: Well they don't give them to me.

Riham: What do they tell you?

Participant: They tell me the insurance doesn't cover it.

Riham: They’re really expensive.

Participant: Yeah.

Riham: Have they written you a prescription because you have diabetes?

Participant: Yeah, yeah I have a prescription from the doctor.

Riham: They tell you it doesn't cover it?

Participant: It doesn't cover it. Some days the insurance doesn't cover it

Riham: You have to pay out of pocket.

Participant: Yeah.

Riham: So the dentist, and the test strips you need for diabetes, those are out of pocket.

Participant: Yeah out of pocket.

Riham: Is there anything else that’s really expensive that you –.

Participant: No no no, the diabetes medicine, they give that to me.

Riham: Yeah the medication for diabetes but the test strips so you can see your blood sugar levels.

Participant: Yeah.

Participant's Wife: If they don't prescribe it to us they don't give it to us.

Riham: But if its prescribed to you, do they give it to you?

Participant: Yeah.

Riham: Okay now I understand you. So if the doctor prescribed it to you, you can go to the pharmacy and they give you the test strips.

Participant: Yes, exactly, they give it to me but if I run out of them and I go to get them they tell me no you have to pay for it.

Riham: You don't have something called a refill?

Participant: No, what do you mean?

Riham: Refill means, if the medicine finishes for example, and the doctor says that –.

Participant: That’s like what the cardiologist wrote a prescription for me for. When I first came to America, I had a heart catherization, he opened the artery and now there is a medicine that I take. I’ve been taking it for about a year.

Riham: You’re young to have heart problems. Did you have them before?

Participant: I got them in Jordan.

Riham: Did you get a stroke?

Participant: No not a stroke, its a kind of stroke. I had sugar in my arteries.

Riham: Okay, do you have diabetes?

Participant: No, sugar in my arteries. I went to my doctor there in Jordan and stayed in the hospital about 15 days and after I got out I had to come to America. So the doctor came and I told him hey doctor I have to travel to America. So the doctor said go to America, you know what I mean? And over there do the surgery.

Riham: So you didn't do it in Jordan?

Participant: No, he said, “do it there it will be better.” So I came here and… William whats his name… the case worker, I came here with some medicine. My medicine finished, yeah so I went to him, the guy in charge of us [caseworker], I told him I need this medication its really important.

Riham: Yeah for your heart!

Participant: Yeah so he said “No. We have to make a doctor’s appointment for you for him to examine you.” I mean, between me and you, until he believed hey does this guy really have it or not –. So I got really dizzy, I was with them there in the organization. I fell down on the floor so they called the ambulance. I went out and then they said –. In the hospital they put me there for about three or four days until I saw the cardiologist. The cardiologist said you need –. We need to open your arteries so they put me a stent.

Riham: Oh, they put you a stent. So they did a catherization and they placed a stent for you.

Participant: And they gave me a piece of paper.

Riham: To tell you what they did.

Participant: Yeah, here’s the paper. The doctor told me wherever you go, keep this paper with you because when you go out it beeps.

Riham: RCA [Right Circumflex Artery]. yeah wow do you see what I mean?

Participant: Yeah.

Riham: Did you meet a Syrian Cardiologist?

Participant: No, an American.

Riham: There is one that works at UC. He’s really good but he might have moved by now.

Participant: I don’t know.

Riham: You didn't get to know him.

Participant: No.

Riham: He’s a really great young man.

Participant: Aren't I telling you? The case worker came and I told him if I would've died, whose fault would that have been?

Riham: How long were you in the country when that incident happened to you?

Participant: Two months.

Riham: So you didn't see a doctor for two months?

Participant: No wait more than that, it was maybe three months!

Riham: So when you came to America, and this is something I am trying to understand myself, what I understood is that within thirty days of a refugees arrival they have to see a doctor.

Participant: No no no, we didn't see any doctors.

Riham: So maybe they’re making a mistake because if someone has a health problem like you they have to immediately see a doctor… because obviously they need the medicine.

Participant: Sorry to interrupt you but even when leaving Jordan, I told them I have such and such problems. So even in Jordan they referred me, the United Nations referred me to a doctor and he wrote them a report, and the report came with me with all my papers.

Riham: So in your papers it says that, “This man has medical problems?”

Participant: Yeah. Yeah and I brought the papers with me and I had everything.

Riham: After that incident happened to you.

Participant: After that incident happened to me thank God.

Riham: God protected you.

Participant: Yes, thank God. I told him I have some medicine with me and after the medicine runs out I get really tired.

Riham: Well before the medicine finished when you first arrived, of course when a person first arrives there are a lot of things but you told them, “hey I have to see a doctor, I have a heart problem?”

Participant: Yeah, yeah, yeah. I told them so they said okay lets first make an appointment with the family physician and when you go to the family physician you tell her I have such and such problem and then she refers you to a cardiologist.

Riham: Did you see this family physician?

Participant: Yeah, we saw her.

Riham: Did you see her before the incident where you fell down or after?

Participant: Yeah we saw her before the incident.

Riham: So that’s what I am asking about. When you saw that doctor, did you tell her, “I have to keep taking this medication?”

Participant: I told her and such and such and she said yeah I’ll make an appointment for you but now you have vaccines.

Riham: What do you mean by vaccines?

Participant: I mean right when you come to America they have to give you all these vaccines. She told me once you finish taking all the vaccinations, do you see what I mean, I’ll make an appointment with doctors for you. So she made an appointment with the dentist for me. I told her. I brought the medicine to her, and I showed it to her. And I told her when this medicine runs out, I get really really tired and she told me “No no I am going to refer you to a doctor.” She made an appointment for me to go and see her so she can refer me to a doctor. So in that time period between her referral and my appointment, this happened to me.

Riham: Oh, I understand you. So they started to help you with this problem but because you were waiting for the appointment –.

Participant: Exactly, for the appointments.

Riham: The medicine finished.

Participant: It ran out.

Riham: This doctor couldn't have refilled it for you?

Participant: She didn't allow it. She said “I won’t refill the prescription for you until you seek a specialists examination.” So I went to a hospital. The ambulance took me to a hospital.

Riham: Do you know the name of the medicine or not? Is it Nitro?

Participant: Come, come, come, bring the medicine lets show it to her.

Riham: Is it a small pill?

Participant: Here, put it for her.

Riham: So heart problems. What are the other problems?

Participant: The other problem is that I have back pain.

Riham: Back pain, okay.

Participant: I went to a doctor

Riham: Oh my God! How many bottles do you have?

Participant: That’s just that medication.

Riham: Oh, it’s Nitroglycerin. That’s what I thought it was.

Participant: Yeah, I have to take it under my tongue.

Riham: When you have chest pain.

Participant: When I have chest pain. I keep one here and I keep one in the car. Come show it to me. This is for blood pressure.

Riham: Do you find a problem understanding what the pills are and how to –.

Participant: Well these big pills here, here show these to her. There’s another bottle. No not this one. Sorry.

Riham: So how do you know which one to –.

Participant: So these pills are for back pain. I can’t take from these unless its night. At night, I take them. How do I use this? I don't know, I am just taking it like that.

Riham: If its name isn't mentioned, I can’t help you. It doesn't have its name on it at all.

Participant: He tells me, for example, take it. What I understood from the doctor and it’s not written. For example, the first day you take three pills, the second day you take four.

Riham: That’s right, but what is it? What’s the medicine?

Participant: It’s for back pain and this is for back pain. There’s another two –.

Riham: How do you understand? Because you have one, two, three, four, five, six, seven, eight, nine, ten, eleven, twelve, thirteen pill bottles. How do you know which one to –.

Participant: Sorry to interrupt you. There is a medicine that has the same bottle as this one.

Riham: So you know based on how the bottle looks? That’s how you remember them?

Participant: Yeah, that’s how I remember them.

Riham: So that means you’re pretty smart.

Participant: So for these -- for example -- I take them in the evening before I go to sleep and for these -- I take a pill before I go to sleep.

Riham: Can you write in Arabic?

Participant: No honestly I do not know how to write in Arabic.

Riham: I was going to tell you if you knew how to write you could’ve written it on the –.

Participant: No, I don't know how to write, but my wife knows how to write in Arabic. She studied. So this pill I have to take one a day, and this one is for my chest.

Riham: But you only have to take this pill when you feel pain

Participant: I get pain at times.

Riham: So some of them are the same thing.

Participant: This one is for the back. Do you see what I mean? This one I have to take a pill a day. One day I take it, one day I don't.

Riham: What did you say this is for?

Participant: Which one? Oh, this one is for cholesterol. That one, I have to take a pill a day so the stent doesn't close up. I have to take that one.

Riham: Okay

Participant: So I have to take this, it’s a blood thinner. I have to take both of them together.

Riham: So you just look at the pill and memorize it.

Participant: Yeah, exactly, that is all I do but this pill they gave to us. Hey open this for me,

Ruqayah. Yeah, but this pill they gave me, I haven't dared take it. Is it to thin blood? Can you take a look for me?

Riham: Yeah, that’s Aspirin. You have to take it everyday.

Participant: No, there is another one that they gave me. Where is it? This one. So I don’t dare take the other one. I’m only taking this one that I know.

Riham: It’s the same thing but it’s just a different color.

Participant: So it’s the same thing but it’s a different color?

Riham: Yeah. It’s the same pill, but it just looks different.

Participant: Okay, finally I understand!

Riham: Oh, this is really hard. So someone has to memorize each one.

Participant: I memorized them by their color.

Riham: By their color.

Participant: I know based on this one’s color that it’s a blood thinner. Do you see what I mean? But they gave us this one right now, and I don’t dare take it.

Riham: It’s the same thing like I told you. It’s the same thing.

Participant: And I was afraid to take one pill from this type and one pill from that type for something to happen to me.

Riham: And with all this medicine in front of you, you keep smoking. You smoked two cigarettes right in front of me.

Participant: Yeah.

Riham: Do you know how old you are?

Participant: I am 47 years old.

Riham: You’re 47 years old. You’re young. You have 13 pill bottles. You see all the problems that you have all because of you’re smoking. I mean come on [haram alyk].

Participant: I don't know what to tell you. You don't know what we saw. Whoever doesn't smoke, starts smoking. Leave it to God.

Riham: When did you start smoking?

Participant: I started smoking when I was about 20 years old.

Riham: You were young.

Participant: About twenty or twenty-five years old.

Riham: So you mentioned something. This is going to open up the discussion for something else. You said, “oh you don't know what we saw, we have to do something about it.” So smoking calms the nerves?

Participant: Yeah, and for me… what can I tell you. Honestly, I do not know what to tell you…

Riham: Just tell me, it’s okay.

Participant: You don’t know what things we’ve seen. The incidents. I swear to God it makes a person....let’s just leave it to God.

Riham: God is great.

Participant: God is great. There is no one greater than Him.

Riham: So tell me some things that you as a father and as a husband do to help yourself and to help your family to take care of your psychology, your mental health. Because, like you said, the incidents that you saw, are not forgotten.

Participant: No no, honestly, no no. Hardship is never forgotten. When someone –.

Riham: I really like that proverb. Hardship is never forgotten.

Participant: Hardship is never forgotten. What year was it? 2011, 2012, 2013, 2014, 2015, 2016, 2017. It’s been six years since I’ve seen my parents.

Riham: How sad.

Participant: We talk on the phone, but it’s been six years since I’ve seen my parents. I call them. They talk to me. And I have a brother that was martyred.

Riham: May God have mercy on his soul.

Participant: He left five daughters and a son behind.

Riham: How sad.

Participant: I mean we’ve gone through difficult times. We went through tough times. Really, really, really hard.

Riham: Really hard.

Participant: And in Jordan, they also messed up. They wouldn't give us papers to even enroll the kids in school. I mean thank God we’re still standing on our own two feet.

Riham: Thank God. How long did you spend in Jordan?

Participant: We spent three years.

Riham: So a long time, huh?

Participant: Yeah, about three years.

Riham: So tell me, what you do here, in America, to help your psychology, your mental health. Like when you feel –.

Participant: I go out and I work.

Riham: So what if there is no work today, what do you do?

Participant: If there is no work, I sit at home.

Riham: But what do you do with yourself if you are bothered, if you feel stressed –.

Participant: I go out.

Riham: Okay. You walk?

Participant: No, I don’t walk. I go and sit outside, I drink coffee, I smoke a cigarette.

Riham: You smoke a cigarette.

Participant: I smoke a cigarette.

Riham: Do you drink some tea?

Participant: I don’t drink tea. I don’t drink tea. Yeah, I drink some coffee.

Riham: Are there other things you can do too for your psychology?

Participant: Like what?

Riham: I am asking you, because everybody, how old are you? You’re 47 years old, you know yourself. You know what you need to do to relax, to cope.

Participant: When I am really bothered or angry or have a lot of stress ,I go out. It doesn't matter where, I just go out. For example, if I am at work and I get really angry, I just keep working. Then at work when I get angry, if someone says something to me that upsets me, I open the door and I keep walking! My wife, when she says things that make me really angry, I open the door and I keep walking. I walk out.

Riham: So that’s good. Everybody has something they do.

Participant: Yeah, I open the door, I start walking. I leave. I keep going.

Riham: Well what do you teach your kids? Because obviously your kids were moving, they went through so much, a different language, a different country. What do you teach them to do for their psychology?

Participant: I tell them, learn the language and study. Whatever you want. I am ready to give. As long as I am healthy, God willing, I am not going to let them be in need of any other person.

Riham: That’s great.

Participant: But if the Master of all creation, Allah, takes me away, they’ll have to take care of themselves.

Riham: But if you find them for example, your daughter got really bothered by something at school. Something that really bothered here. What are the things that you teach her to do?

Participant: I teach her that you know a person has to calm down and nothing is going to be solved by getting angry or getting stressed. But it will be solved with patience. All of it solved with patience. Yeah.

Riham: Okay. I know that time is running out but are there things that, herbs that you use or natural remedies that you want to help your children at home. For example, my mom, if I am angry, she would give me yogurt. She would say here eat some yogurt. I got sick of yogurt, but thank God, she’s right. So are there things you use as a family? Herbs or food or anything else? Or if someone is –.

Participant: No no no.

Riham: Well if someone is sick, coughing or anything –.

Participant: No, no, no.

Participant's Wife: If someone has a runny nose or cough, we give them herbs.

Participant: Yeah, herbs.

Riham: Okay.

Participant: If the kids get bothered, I take them –.

Riham: There are ten minutes left.

Participant: For example, if the kids are bothered I take them out. I take them on a trip.

Riham: Where do you take them?

Participant: Sometimes I take them to Heyam’s house. Over there they have a pool and a park. The kids go swimming, and they have a great time. Some days I go down there, do you see over there and I grill for them. We spend a good weekend together.

Riham: Are there needs you have that you wish your children had when you first arrived and now to help their psychology?

Participant: Yeah, honestly.

Riham: Tell me about it.

Participant: For example, how can I tell you? For them to go study, to spend their time studying. So they can play a game. Like for example, they spend the whole summer…just sitting at home.

Riham: You mean their free time?

Participant: Yeah in their free time. They just sit at home. They don’t go out. If I don't take them, they don't go out.

Riham: So you wish there was a program or something?

Participant: Yeah a program or something. Like a Summer program. A program with studying and knowledge and going out and –.

Riham: Okay. Anything else you can think of with regards to their health that you wish they had?

Participant: Thank God their health is really good.

Riham: They don’t have any needs other than their teeth?

Participant: That’s only Ruqayah. Thank God, yes.

Riham: And your children?

Participant: My kid’s eyes.

Riham: What’s wrong with his eyes?

Participant: His vision, it’s been like that for three or four months. We’ve been trying to get an appointment for his eyes to get checked up. He has been complaining of his vision.

Riham: Did they do a vision test?

Participant: No, no, they didn't do a vision test or anything.

Riham: Don't they give him a vision test at school?

Participant: I mean they’re on Summer break so I don't think they had a vision test. Hey, did you have a vision test?

Participant's Wife: For his eyes?

Riham: When your kids arrived to this country, did they make a doctor’s appointment for them to examine their health, from head to toe?

Participant: Yeah.

Riham: Did they find any medical problems or did you find any needs that they didn’t help you with?

Participant: No no, thank God they don’t have any problems.

Riham: Is there anything else? I know time is running out but is there anything else you want to tell me while the recorder is on?

Participant: No there’s nothing. Right now there is nothing.

Riham: Think. Are there any big problems This is your time, your chance, to tell me any problems that you have. If there is anything related to health.

Participant: No, thank God there are no problems but like I told you with regards to health, it’s like they gave me this paper. They said, here make a doctor's appointment. Yeah but if I call the doctor, what am I supposed to tell them? Now if I call the doctor and the doctor speaks to me, what am I going to tell him? I speak Arabic and he speaks English. I mean I am finding a really big difficulty with this.

Riham: So it is making appointments. That’s the biggest difficulty.

Participant: The biggest difficulty are the appointments and the language.

Riham: The language.

Participant: Yeah, the language.

Riham: So, we can’t learn the language easily. That’s hard. In your opinion, what can help you or what are the things you wish you had to help you learn?

Participant: If they had a program for us. Even once a week, two or three or four or five hours to study. Do you know what I mean?

Riham: They told me there are classes for other refugees.

Participant: At the mosque.

Riham: Before the ones at the mosque, weren’t there language classes in the resettlement organization?

Participant: What classes are you talking about? They don’t teach you anything in the organization. What classes? Yeah, in the mosque, every Sunday they gave you a class. It’s only an hour long, but you enter that hour and you meet all the other Syrians. You sit down and say, “Hey, how are you, how has your week been, what have you done?” And then the hour is over and then you say, “Goodbye!” Yeah, honestly.

Riham: You mean it’s not a class?

Participant: It’s our fault. It’s not organized. We need organization. We need a curriculum. I mean, I went there with Abu Abdallah, we sat there chatting and the teacher was looking at us.

Riham: So it’s your fault, then.

Participant: I told you, it’s our fault. So that’s what’s happening to us.

Riham: But it’s at least good so you can meet each other. It’s an opportunity.

Participant: Yeah, it’s an opportunity for us to meet. So we don't learn any English there, we just spend the time talking about what we’ve done. What we worked in this past week. What has happened to us.

Riham: I got you. So what’s a person to do?

Participant: You have to learn. We have to find a way to learn.

Riham: If someone, for example, sent you a voice text or text message about your appointment, would that help you or not?

Participant: What do you mean, in Arabic?

Riham: In Arabic or in English.

Participant: If it’s in Arabic that would help me.

Riham: Okay.

Participant: If it were in English, that wouldn't help me at all. Because many days I get phone calls, they try to speak to me, and I don't understand them… and they don't understand me.

Riham: But at least, thank God, you’re able to understand your medications.

Participant: Yeah, thank God.

Riham: And that’s really good. Don’t miss your appointments with the cardiologist.

Participant: Thank God, the big issue I have with regards to medical problems is my back. Because I went to an Arab doctor and he wrote a prescription for this medicine for me and he told me I have cartilage.

Riham: How are you finding these Arab doctors? Is an organization helping you or giving you a list of names of doctors who speak Arabic?

Participant: No, no, no. No, there’s nothing like that. In the mosque, I got to know this doctor.

Riham: So it was just by chance that you got to know him.

Participant: Yeah so I got to know him and I told him I have such and such problem. He said “Do you have insurance?”, I said “Yeah”. He said “Go to your doctor and have her refer you to me”. I said “ Okay good.” I went to my doctor and I told her “I want to see this doctor. Please refer me to him for my back”. She said “okay”. She referred me to him.

Riham: Good.

Participant: Yeah so they put me in a machine to take images of my back. They put me in there for maybe –.

Riham: Two hours.

Participant: No, it was maybe 30 minutes or so.

Riham: Yeah, it depends.

Participant: They put me in there for five minutes and I felt like I had difficulty breathing so they took me out.

Riham: They didn’t ask you? They’re supposed to ask you before they put you in the machine “Do you have difficulty breathing?”

Participant: They didn’t ask me that.

Riham: Oh, they can give you a medicine to calm you down.

Participant: How am I supposed to know, they didn't ask me. So I went out and told the doctor, such and such happened.

Riham: He can’t help you then.

Participant: Yeah, he told me, “I can’t help you, I need the image. I have to have it”. Yeah, and I am finding that a really big problem for me. Now they’re asking for an image and to go and what not. I don't know. That second time I went, I spent about ten minutes in the machine. Right?

Riham: Why don’t you tell him that you get a shortness of breath or anxiety. They can give you medicine to calm you down, that puts you to sleep.

Participant: I didn't tell them that.

Riham: That’s two times that you missed the opportunity.

Participant: Yeah.

Riham: So, God willing, the third time –.

Participant: Yeah, the third time, God willing, but we need another appointment.

Riham: Okay. So we just have two minutes left.

Participant: That other week, it was really serious, it hurt so much I couldn't get up from the ground. Now if I life a little bit, I have to go back home and need to wait a week.

Riham: I hope you find relief.

Participant: Thank you. Those are the medical problems that I have.

Riham: Thank you so much for your time.

Participant: You’re welcome.

Interview 8B

Riham: Today is August 15, 2017. I have a mother with me who has three kids. She has been here in Cincinnati for a year and two months. Now you pull out some trays for us! Okay go ahead, how are you?

Participant: Honestly very good, thank God, Master of all creation.

Riham: All praise and thanks are due to God. I am going to ask you some general questions and then we’ll talk more general together. In your opinion what are the biggest problems and medical needs that you are finding in your family and children?

Participant: With regards to medical problems, the biggest problem is just the appointments. Like for example. I don't know how to make a doctor's appointment if I need to go. That’s it, really.

Riham: Okay, so tell me. You’re saying you don't know a lot about doctor's appointments because you’re not used to making doctor's appointments?

Participant: No I got used to it. Little by little I got used to how to make an appointment. But sometimes they give me a number for me to call and make appointments. And sometimes they ask me things like, I don't know like they tell me –. They ask me, “which doctor would you like to make an appointment with?’ and I don’t know.

Riham: Oh, I understand.

Participant: So, “which *kind* of doctor do you want”, and I don't know that.

Riham: So it’s the language or –. Are you talking in Arabic or what?

Participant: So for example, my daughter helps me with the language. This little one here but when we get to that point, we don't know what to say. So yeah you can say it’s a language problem.

Riham: Tell me which appointment was this that you were trying to make?

Participant: I was trying to make an appointment for my husband in UC hospital. So we were trying to make the appointment, they answered the phone. They asked us, “Who do you want to make the appointment with?” I gave them the full name and everything but I didn't know the type of doctor. So they could not make the appointment.

Riham: What kind of appointment where you trying to make?

Participant: It was for my husband. The appointment was for my husband.

Riham: Like a general checkup or specialist?

Participant: No, a specialist.

Riham: For what?

Participant: Because he needed to do some tests.

Riham: Tests for what?

Participant: Long pause. Giggles. The doctor, the doctor wrote down that he has to get some tests.

Riham: Oh, that paper that you showed me….You want to get pregnant. You went to a gynecologist and you’re trying –.

Participant: I am trying to get pregnant.

Riham: You have a medicine and you’re taking pills. You’re doing tests then they want to send your husband to a specialist.

Participant: Yeah, a specialist so he can do some tests.

Riham: So that doctor, based on what little I know, I think is a urologist.

Participant: Exactly.

Riham: So they didn’t tell you the type of specialist to ask for…or make the appointment for you. They just gave you a number.

Participant: Exactly, they just gave me a number.

Riham: So what are you going to do with this number? I understand what you mean. How did you feel after that?

Participant: Honestly, I didn’t know. They said no it’s not going to work. So we shut the phone and it didn't work out.

Riham: Did you call your doctor and tell them what happened?

Participant: No I haven't gone there yet.

Riham: Okay when do you have your next appointment?

Participant: No –. In three months.

Riham: Do they think the problem is with you or with your husband? With regards to getting pregnant.

Participant: Honestly. I don't know the cause of the problem. I mean the doctor is checking me up and sees that everything seems to be fine. He was looking at my blood and sugar levels for three months. He gave me a medicine to take for two consecutive months and it didn't work.

Riham: Okay, how old are you?

Participant: I am 34 years old.

Riham: So you’re still young.

Participant: Mm.

Riham: So you want to get pregnant?

Participant: Mm.

Riham: So tell me a little bit about what your perceptions when you are going to the doctor. When you are trying to get transportation there. Are you having difficulty with these?

Participant: Honestly, sometimes I face a few problems. I get a little mixed up. I mean the interpreter that’s with me explains to the doctor but sometimes some words are either not being said correctly or he’s not explaining them to me correctly. It seems there is confusion.

Riham: For example?

Participant: For example the first time I was given the medicine I took it once.

Riham: Yeah.

Participant: So when I went back to the doctor he said, “No, you were supposed to take it for two months straight.” I said, “They didn't tell me I had to take it like that.” I got it and I took it, until the bottle finished!

Riham: Yeah.

Participant: And that was it. He said, “no you were supposed to take it for two months straight.” So when I went now he said, “no, you were supposed to take it for two months and then come back.” So –.

Riham: Yeah.

Participant: Now this next time I went back and he said, “no you have to keep taking it. Some people take it for a full year.” Frankly I told them I want an Arab doctor so he can understand my condition – and we can speak to one another.

Riham: So you can understand each other.

Participant: So I can understand what he wants and what he doesn't want because maybe the interpreter –. For example, the other time he was a male interpreter! To interpret for me.

Riham: That’s tough.

Participant: And frankly, I don’t like to be with a male interpreter. To be with a gynecologist and for him to interpret for me.

Riham: And is the gynecologist a male or a female?

Participant: No, a male.

Riham: So you were okay with the gynecologist but not with the interpreter.

Participant: Yeah, I didn’t like that the interpreter is a male. I like it better when it’s a female. It’s more proper I mean.

Riham: Yeah, yeah, yeah, because you're talking about private matters. Of course.

Participant: Yeah.

Riham: So were they able to refer you to an Arab gynecologist?

Participant: No they didn’t try but I want to go to an Arab gynecologist.

Riham: Did you ask them for a referral?

Participant: They didn’t have any Arab doctors over there.

Riham: Okay where do you go?

Participant: I go to –. What is it called? That hospital –.

Riham: Millville?

Participant: Yeah.

Riham: Okay, Millville. Remind me when we end God willing I will give you the number of an Arab gynecologist I know but he’s a male. Still he’s an Arab, he’s Syrian, and I know him he’s really good.

Participant: God willing.

Riham: Okay with regards to transportation, are you finding any problems going to the doctor or to the hospital?

Participant: No, most of the time I am going to that one in Millville. Yeah and sometimes I find that transportation is difficult. Some days I have to go and my husband has to go back to work, and I take a long time at the doctor. It could be two or three hours so sometimes I call someone I know so they can take me back home.

Riham: It’s far from you? You’re not able to walk to take the bus?

Participant: No, it’s far.

Riham: Did you try taking a bus?

Participant: No honestly I don’t know how to go by bus. I have never gone by bus or anything. I would get lost.

Riham: Yeah because it’s confusing.

Participant: It’s really confusing and the streets are really big.

Riham: So tell me about your children’s health here. Is it good or not good? Are you able to take care of their health?

Participant: No thank God, Master of all creation, their health is good. Whenever they get sick or are struggling with an issue, I immediately take them to the doctor.

Riham: Who do you take them to?

Participant: The same one for the health –.

Riham: In Millville.

Participant: In Millville.

Riham: Do you find that when you make an appointment and you take your kids, you find yourself at ease?

Participant: Yeah, honestly I find myself at ease.

Riham: So you understand them and they understand you.

Participant: Yeah some days they provide me with and interpreter.

Riham: In person or by phone?

Participant: Sometimes in person and sometimes by phone. Some days like this and some days like that. And if there is not an interpreter on site, they'll call. When it’s necessary to see them, they call me by phone.

Riham: Okay and what’s your experience with the interpreters? Tell me. Good, not good?

Participant: Honestly my experience has been good. Most of them don't have any issues but when I go with my personal problems, I like to go with a female interpreter.

Riham: You mean a female. In your opinion is there anything your kids need or you wished they had when they first arrived to help their health?

Participant: Honestly my kids –. For example, Maher. When he first got here he got a skin illness. I took him to the pediatrician and he remained being treated for a month. He got a skin condition and it went across his body. So she gave me four ointment tubes and I had to apply them twice.

Riham: What do you mean by ointment tubes?

Participant: Honestly, I have them. She gave us ointment tubes, oils, and cream.

Riham: What do you mean by ointment tubes?

Participant: She gave me ointment tubes.

Riham: To put on his skin?

Participant: Yeah to put on his skin and also a moisturizers.

Riham: A moisturizer, okay. So that was something new from when they first came to America.

Participant: Yeah.

Riham: They didn’t have that before they came to America.

Participant: No, they didn't. No my son here didn't have that. No, thank God he was cured by it.

Riham: Were your children's health good in Syria and in Jordan?

Participant: Yeah, thank God Master of all creation.

Riham: Okay so it seems from when I spoke to your husband, it’s his health that requires a lot of doctors.

Participant: And me too. I also have to do surgery now.

Riham: For what?

Participant: I have a hernia. I did it twice before.

Riham: It’s probably from pregnancy.

Participant: Yeah it’s from pregnancy but I’ve done it twice before. Once in Syria and once in Jordan.

Riham: You did the surgery and it’s still opening up?

Participant: Yeah, it’s still opening up.

Riham: How sad.

Participant: They just told me I am supposed to be pregnant and they do the surgery and it’s over. I have to do it because it has a really big size.

Riham: Does it really hurt you?

Participant: Yeah on cold days, it really hurts me.

Riham: Did you go to a surgeon here?

Participant: No.

Riham: Why not?

Participant: Because I still want to have kids, they told me it’s not worth it if I do the surgery and get then pregnant. Because I’ve done it twice already and I would do it the third time here but after I get pregnant.

Riham: Okay may God protect you.

Participant: May God protect you too. In Jordan they said they put a ru’a [a piece of cloth] in my stomach.

Riham: What does ru’a [a piece of cloth] mean?

Participant: I don’t know. This is a doctor's thing.

Riham: A paper?

Participant: No. I don’t know. The day I went –. I had it done in Syria with no issues but in Jordan they told me it needs a ru’a. I don’t know that’s what the doctors told me. We told them okay. Then when I went they told me it doesn’t need anything but now it needs a net.

Riham: I understand what a net is. It’s mesh. The other one, I didn’t understand.

Participant: The mesh is necessary now because this is the third time I have to do it.

Riham: This is the third time.

Participant: Yeah.

Riham: Get well soon.

Participant: Thank you.

Riham: May God protect you.. Okay, may God make it easy for you. Now when you have this problem and you talk to your doctor and you tell her about this problem, do you find that

you’re able to understand one another?

Participant: Yeah, for sure. For example, if I have a problem, I call her and make an appointment and see what the problem is.

Riham: Okay, thank God.

Participant: Thank God.

Riham: Okay, let’s talk about the things that you use at home that help you *maintain* your health, your husband’s health, and your children’s health.

Participant: Like what do you mean things I use at home?

Riham: Like as in what means do you use, herbs, food –. Tell me about those.

Participant: Honestly, I really do feed them a lot of yogurt.

Riham: Same as my mom.

Participant: Yeah, I like to feed them nutritious food. The most thing I like to feed the children is fruit. Yeah and meals. Two meals at least per day.

Riham: So you make sure you always give them two meals.

Participant: Yeah and I give them lots of fruit because it’s really good for them.

Riham: Okay good. Because our food is different than American food. Did you face any difficulty finding the vegetables and things that you need or are used to?

Participant: No, thank God. I didn't face any difficulty because everything is available here.

Riham: Okay, thank God. So you found the stores that have Arabic food and everything.

Participant: Yes I found them, thank God.

Riham: Thank God. Did you spend extra time looking for these things?

Participant: Yeah, I spent some time looking for the things I wanted and needed.

Riham: What are some things you found that surprised you?

Participant: Surprised with –. I wasn’t really surprised because I found everything. You can look and know what it is. At first when I wanted to buy yogurt, I didn’t really know how to. They have many different kinds of yogurt so I wouldn’t know.

Riham: Oh, I understand you, yeah. Do you make labneh [a type of yogurt] from scratch?

Participant: Yeah I make it.

Riham: You’re really good, praise be to God.

Participant: Yeah I make everything, thank God.

Riham: Do you make maq’doos [stuffed pickled eggplants] too?

Participant: Yeah

Riham: Okay next time you make it, just give me a small jar.

Participant: We’ll give you now. We have some left.

Riham: You really have some? I love maq’doos [stuffed pickled eggplants]. The homemade one is different than the one bought in the store.

Participant: Yeah, that’s true. No and labneh and everything alhamdulilah, we make.

Riham: That’s really nice.

Participant: Yeah.

Riham: We look for stuff like that in the store but you make them from scratch.

Participant: Yeah, I don’t like to rely on ready made food, I like to make it all from scratch.

Riham: Okay but are you finding difficulty to do that, take care of the house and everything, because you are working outside your home too?

Participant: What are we going to do. I found a little bit of a difficulty but no, thank God, I am ok.

Riham: Thank God. You told me you have work now that you’re happy with.

Participant: Yeah, thank God, I am happy with it.

Riham: Did you start working right when you arrived or –.

Participant: No, I didn’t start working right away. I have been working for three weeks now.

Riham: Okay. What were you doing before?

Participant: I would mostly spend it with the kids. When we first arrived they had many doctor’s appointments so I had to stay with them.

Riham: So you could take them –.

Participant: So that I would take them and bring them back. Take them and bring them back.

Riham: Okay, was there anything during that time that could’ve helped you?

Participant: Honestly, I would take them and bring them back. There wasn't anyone –. Who would help me?

Riham: But you don’t drive.

Participant: No, I don’t drive but their dad would take me and I would stay with them. I couldn’t leave them alone.

Riham: Of course they’re young.

Participant: I would have to sit with them till he came back.

Riham: Okay.

Participant: Honestly, I got exhausted.

Riham: Yeah, that’s why I was shocked. You were telling me there were four appointments but others would tell me they had many appointments. Maybe you forgot?

Participant: No, I told you there were many appointments. I had appointments for the vaccines and for the –.

Riham: Yeah, so how many?

Participant: How should I know? I am going to sit and count them?

Riham: Say ten or twenty then.

Participant: Ten or twenty appointments

Riham: Okay but you told me four. I wrote four on the other paper!

Participant: No, like appointments for the vaccines are one and when they’re sick thats two –.

Riham: Okay, I'll write 14 since the four is already written here.

Participant: Yeah, It doesn't make a difference.

Riham: What are the social problems that you found here that affected your health or your family’s health?

Participant: Social problems? Thank God, I don’t have any social problems in my family and such.

Riham: Social, as in the society in which you live in. Societal problems?

Participant: In the Society that we live in? Honestly, I enjoy calm environments. I am a homebody. I am educated and acclimated to life here. Thank God, the Syrian people have acclimated to life here.

Riham: Thank God.

Participant: Thank God, Master of all creation.

Riham: I am asking –. For example, some people are sometimes finding problems with lets say their neighbors or with the schools. The kids maybe are struggling the schools. Do you know what I mean? Did you find any problems like these?

Participant: No, thank God. The neighbors all keep to themselves.

Riham: Thank God. No one bothered you?

Participant: No, thank God no one bothered us.

Riham: Okay good. You didn't find it unsafe at home?

Participant: No, honestly. Thank God.

Riham: Thank God. Some people, as I was getting to know them, were placed in areas that are a bit scary. They had violence around them. Things like that.

Participant: Yeah, that’s true, that’s true.

Riham: You didn't experience anything like that?

Participant: No, thank God. As for us, sometimes we feel like the environment is a bit chaotic at night.

Riham: What do you mean by chaotic?

Participant: Honestly, I don’t know what happens in this neighborhood.

Riham: You mean screaming and yelling? Stuff like that?

Participant: Yeah kind of like that. But praise be to God, the security here is found everywhere. Filling the city!

Riham: What do you mean, as in the police?

Participant: Yeah.

Riham: That’s good, thank God.

Participant: Yeah, as long as we have the police around –.

Riham: You feel at ease.

Participant: Yeah, we feel at ease.

Riham: So you do not feel afraid if you’re out alone?

Participant: No, honestly I don't like to go out at night alone.

Riham: Okay.

Participant: During the daytime, yes I do go out alone. It’s normal.

Riham: You go out and walk around for example?

Participant: No, I don’t go out and just walk around.

Riham: Why not?

Participant: I don’t like going out alone like that and walk in the streets.

Riham: I mean to exercise.

Participant: There’s nothing like that expect sometimes I go out to visit an Iraqi lady that lives out front. Some days she comes visits me and sometimes I go visit her for an hour or so and come back.

Riham: That’s great. But you do not walk there to exercise?

Participant: No.

Riham: Okay, do you do any form of exercise?

Participant: Well some days –. There are [indistinct] where they put some exercise machines. Sometimes we find parks. Outside. My husband takes me and the kids. The kids play for a bit and I go there and exercise.

Riham: That’s really nice. Thank God.

Participant: Yeah, honestly, thank God.

Riham: You encourage them to play –.

Participant: Yes honestly, I encourage them. I like my kids to exercise.

Riham: Yeah.

Participant: And some days –. Yesterday I bought my oldest son some weights. He likes to lift so I said let me buy it for him.

Riham: Nice! So you bought him the weights.

Participant: Yes I bought him the weights.

Riham: That’s great. So they can spend time –.

Participant: Yeah because my kids are often stuck at home. They don’t go out. I don’t really feel safe letting them go out alone, you see.

Riham: So you don’t let them go out.

Participant: No and they don’t go out. If I am not with them they don't go out.

Riham: Why? Do they get scared?

Participant: Yes, they get scared honestly. [indistinct] there isn’t safety here, in the neighborhood, as they say.

Riham: Why?

Participant: Unless either the dad or the mom is with them.

Riham: Okay, because you don’t really know the people –.

Participant: I don’t know the people and who is coming close –.

Riham: Did you find that they have any problems with friends at school?

Participant: No, thank God. My kids don’t have this problem.

Riham: No one bothered them or such?

Participant: No, no one bothered them.

Riham: Okay then thank God. Because there were some kids that faced a lot of problems at school.

Participant: No, honestly. My kids didn’t deal with such problems at school.

Riham: Thank God. Is there something that you do at home –. I asked your husband this question so I am going to ask you the same question. Is there anything that you do as a mother or you say to your kids so that they can be at ease psychologically? Regarding everything they saw in their lives?

Participant: Yeah. When it comes to my kids I like to calm them down and teach them right from wrong.

Riham: Okay. How do you calm them down? Tell me.

Participant: I let them open up and share what they’re going through. For example, if one child fought with another or –. Like take my middle child, if on some days he argues with his friend, I like that he tells me. So I can teach them right from wrong. I’d say, “no it’s not right that you hit your friend or that he hits you, tell the teacher.” Because the teacher will resolve it for them justly.

Riham: Okay so if they are bothered you talk to them. Is there anything else you can think of that you wish they had that can help ease their stresses?

Participant: No, I also like that if some days, for example, they asked me for something or they need something, that I bring it for them. That’s it like whatever you need, for example, a laptop or such, I like to get it for them. So that they can be busy with it or learn something. So they can be distracted. So if they ask for things like that, I bring it for them.

Riham: Okay so what if you don’t have money to buy it for them? A laptop is like really expensive.

Participant: No, I bought them one that wasn’t necessarily really expensive.

Riham: You found something and managed to –.

Participant: I managed to, they played [indistinct] –. And yesterday, I don’t know what they call it, that thing that they play games with –.

Participant's Child: X-box.

Riham: X-box. I also don’t know much about these things.

Participant: Yeah this they plug it in the TV and they start playing with cars and planes, especially boys, yeah I told them to put it. Because my kids don’t go out to play in the streets at all. And where would I take them?

Riham: So you want to keep them entertained at home.

Participant: Yes, I want to keep them entertained and they calm down and play with it.

Riham: Okay, do you also keep them busy with other kids? Do they have friends?

Participant: No they don’t have any friends in the nieghborhood. From this neighborhood they don’t have any.

Riham: Oh, there aren't any kids their ages or what?

Participant: No, no.

Riham: They’re all older or what?

Participant: There aren't any. I don’t know anyone. They’re all Americans. They don’t mix with them.

Riham: It’s hard. Are there any Syrian kids in the same age group?

Participant: No not here. They’re far if you want to go visit anyone.

Riham: It needs a car.

Participant: It needs a car.

Riham: That’s hard.

Participant: Yeah it really is hard honestly. That’s why I like to bring them a ball or something so they can play as much as they want.

Riham: This adolescent age is hard to –.

Participant: Yeah, it’s hard to [indistinct].

Riham: Yeah theres a lot of hardship in it. May God make it easy on you.

Participant: Yeah, honestly.

Riham: Is there anything are worried your kids at this age might fall into?

Participant: Yeah, I get worried for them to –. Not all boys can be befriended.. I would like for a boy that has been raised well, to befriend my son.

Riham: Okay.

Participant: But there are many boys that are not all that. I wouldn't like for my son to become like them or to learn from their traits as we say. Yeah I like if I know one and how his parents raised him and how the boy is, I let my son play with him and speak on the phone.

Riham: Okay thank God.

Participant: Yeah, honestly thank God.

Riham: Okay, let me see. What are some things in your opinion that cause you to got to the hospital immediately? As in medical problems.

Participant: As for me, If I am going to go to the hospital immediately, it’s going to of course be in the case of an illness.

Riham: Okay tell me what you consider to be an emergency illness.

Participant: If ones child has a high fever or something like a burn happened –.

Riham: A burn or an injury –.

Participant: Like something happened suddenly, an injury, for example. Of course I would take him to a hospital.

Riham: Yeah.

Participant: and I would want an ambulance.

Riham: Do you know how to get to the hospital in case of this type of situation?

Participant: Like some days they told me to call the number and it would come [the ambulance].

Riham: But you didn’t memorize it.

Participant : 911

Riham: Okay then you memorized it! You told me before you didn’t memorize it. 911 yeah. The ambulance would come immediately to you. But of course if it’s a small injury –.

Participant: No, I would nurse it here myself at home.

Riham: You would nurse it here yourself.

Participant: But if it’s something one doesn't know how to of course it would need an ambulance and hospital.

Riham: Yeah, okay. Let me just –. There are a lot of questions we discussed so let me just see them quickly. Okay what are somethings that would cause you to take your daughter, for example, to see her primary physician. Something in between taking care of it at home and the hospital. What thing would make you decide to take her to the doctor in the clinic?

Participant: Like if I would take her to see her doctor it would be if they became ill.

Riham: Like what, for example?

Participant: Like if they caught a skin disease or they are complaining from something.

Riham: Okay so if they complain about something then that would make you take them.

Participant: Yeah, I'd take them to doctor and she would examine them and see what’s wrong with them.

Riham: Okay, we asked most of these questions. Did you find that the doctors here treat you well? Tell me a little about your experience with the doctors here.

Participant: No, honestly, they’re all good. How long have I been here? All the doctors were good. Their treatment is really good.

Riham: The nurses as well?

Participant: Yes even the nurses.

Riham: Did you find that the system here is different than your country?

Participant: Yes, honestly. In my country they don’t have a system like this.

Riham: For example I don’t know how it is in your country so inform me a bit. What’s different?

Participant: Praise be to God, here honestly, they really welcome you well. They have good sportsmanship in the way they deal with people. Some doctors –. Overseas, they really kill you with their treatment. The way they sit and interrogate you for two hours or so. That’s if they don’t turn you away.

Riham: Mm. So here it’s different.

Participant: Yeah, it’s different. The doctors here –. The practice of medicine is really nice here in Cincinnati.

Riham: Okay, thank God. That’s really good.

Participant: Yeah, thank God.

Riham: In your opinion what makes it better? I am hearing that its sometimes really good and sometimes really bad. So in your opinion, what do you think makes your experience with doctors or the healthcare system good? What are some things? Like do not just tell me that the doctors are good. Tell me what things make it good.

Participant: They are good in everything.

Riham: For example?

Participant: For example when they are examining, they examine the child really well. They make tests to see what the child is in need of.

Riham: So they draw blood.

Participant: Yeah, they test the blood and they test the urine so they make sure of what the illness is that the child has.

Riham: Mm.

Participant: It’s really good. Yeah, they don’t delay matters or bring in other matters. No, that is it, if the child is in pain, they have to know. They immediately draw blood, for example, so they can know and they test the urine. The practice of medicine is really good here, thank God.

Riham: Okay, thank God. So in your opinion they examine the child well with their hands?

Participant: Yes, honestly. They examine my kids with their hands and everything.

Riham: And also by performing tests.

Participant: And by performing tests. They perform general tests as well.

Riham: Okay and also you are able to understand from them what the problem is from the beginning to the end?

Participant: No, honestly they explain everything –. I don’t really know. For me, in the past, when my kids –. The middle child when he was playing in school he started to have blood in his urine. Yeah, so I don’t understand those type of things so I made a doctor's appointment. She requested some tests and they performed a lot of tests and everything and they said thank God, there isn’t anything wrong with him. She said “since you were playing sports, there was some pressure being placed on the bladder system”. So that’s why it happened.

Riham: So what’s the solution?

Participant: The solution is she said is that he wasn’t playing and now he’s playing here. That’s all, and thank God, it didn't affect him.

Riham: Thank God. Okay so you then felt at ease.

Participant: Yeah, a person with these kinds of things –. I got scared. She immediately sought out the problem and the cause.

Riham: So with the incident you just described, did you go immediately to the emergency room or to the clinic? Meaning the clinic where his doctor practices.

Participant: No I went to the clinic and made an appointment. I told them I really need an appointment because his case his urgent. So they made me an appointment and I took my son.

Riham: How many days did you wait?

Participant: Honestly, about a week.

Riham: That’s really good.

Participant: Yeah. I then told her--. Their doctor sees them frequently. She’s his doctor.

Riham: Okay good.

Participant: Yeah, I made him an appointment and took my son. As usual she ran some tests and examined him and thank God.

Riham: Thank God.

Participant: She told me “No” thank God. She started asking him how many times it happened and everything. She took from him the whole story. She then ran some tests. She told him, “what happened with you was some pressure on your bladder system since you’re playing sports.” Yeah thank God she told him it’s not an issue.

Riham: It seems like your doctor is really good, thank God.

Participant: No, honestly they are all good. Why would one lie? All the doctors’ treatment is really good here.

Riham: That’s good. For the research project, we want to learn whats good and whats not. The good will help us see what--.

Participant: I don’t know. When I take my kids I love to know everything. I like to ask for the whole story. Even she tells me at times “Good” –. I am asking questions.

Riham: Because you’re asking questions.

Participant: Yeah, I am. I am taking my son so I’d like to know everything that happened. So I ask a lot of questions.

Riham: Of course. And you feel at ease from getting their answers.

Participant: Yeah, I do feel at ease. I also like having the interpreter with me to know what is going on. Right or wrong?

Riham: Yeah, of course. Okay that is really good. So just to repeat what you have said to me. I am hearing you say that from the things you like are: that they hear the full story from the beginning to the end. When they ask you a lot of questions to understand the problem. When, the doctor examines the child really well himself with his hands and then uses further tests. Lastly, when they explain everything to you .

Participant: Yes, of course.

Riham: While using an interpreter of course. Without an interpreter, what happens?

Participant: If there isn't a interpreter?

Riham: Yeah.

Participant: It would be a catastrophe. If there isn’t an interpreter it would be a catastrophe.

Riham: A catastrophe.

Participant: Yeah, but they either bring one in person or by using the phone.

Riham: Do you prefer one over the other?

Participant: No, it’s the same. I get my questions answered both ways.

Riham: Okay it’s the same.

Participant: It’s the same.

Riham: What about their dialect? Do you find a problem understanding it?

Participant: Whose?

Riham: The interpreters’.

Participant: No, when I need I tell him to ask and he asks.

Riham: No, I mean the dialect of the interpreter. Sometimes they bring a Libyan one or a Moroccan one. Something like that didn’t happen to you?

Participant: No, it did. One time when I took my daughter, Ruqayah, to the Children’s Hospital, they brought me a Libyan.

Riham: Yeah.

Participant: No, I understand them.

Riham: Oh, you understand them. You’re smart.

Participant: It’s not that I am smart but yeah I understand them.

Riham: Okay, because me for example I can’t really understand them well.

Participant: No they are understandable, praise be to God. The ones they placed in the hospital are really smart.

Riham: Okay that’s really good. Is there anything that I didn’t mention? Something on your mind?

Participant: No, you did well. May God give you good health.

Riham: May God give you good health too. There isn’t anything at all?

Participant: No there isn’t anything.

Riham: Okay let me just review my notes and see if there are any more questions. Is there anything, either at the doctors office or at the hospital, that would improve your perception of medicine here and the system here?

Participant: There has to be something?

Riham: No, I am asking.

Participant: No, not really.

Riham: Nothing that can improve your experience here?

Participant: No there isn’t.

Riham: Okay I guess it means we’re done.

Participant: May God bless you.

Riham: You too! Take care.

Participant: Take care.

Interview 9A

Riham: Today is August 16, 2017. I am Riham with a father of five kids. He came from Damascus and has been here for four months. We will begin now. How are you today?

Participant: Thank God.

Riham: Thank God. Okay so just as we said earlier. It’s going to be like a conversation but I am going to also start by asking you some questions.

Participant: Okay.

Riham: Before we started the recorder you were telling me that you were bothered by the appointments. Tell me a little about these appointments.

Participant: Especially the appointments for the kids. The vaccines.

Riham: Okay, the vaccines.

Participant: At first we struggled because we didn't have a car. By the time we were able to set up the transportation, the appointment would get delayed. Then now, thank God, we have a car.

Riham: Why would the appointment get delayed?

Participant: He would choose not to take us. Because he would take us when he could.

Riham: So lets say you have an appointment. The paper has a time and date for your appointment, right? He wouldn't come and take you?

Participant: Yeah of course. Lets put the kids aside, my wife, has been waiting to go to her appointment and till now he didn't come to pick her up. It kept getting delayed and delayed and delayed.

Riham: Why would it get delayed?

Participant: The first three months was from their end. Then on the 21^st^ of last month we went and they said it was delayed to the 31^st^ of this month.

Riham: Okay, why was it delayed?

Participant: From their end they did that, I don’t know. They changed it.

Riham: For example, you would go to the clinic; they would tell you the appointment is canceled?

Participant: Twice they would say we informed you over the phone. I didn't understand them over the phone. I can’t speak English.

Riham: So you got there and took time out of your day and arrived at the clinic –.

Participant: Yeah and they told me the appointment was changed. It happened twice with us.

Riham: How do you feel about that?

Participant: And twice they told her it’s delayed. I am telling you it’s been four months.

Riham: How did you feel when that happened to you? Describe your feelings.

Participant: I feel like I am blind and deaf in this country. How would one feel? What more than that? I don’t understand them, and I can’t talk to them. If a good interpreter comes, then it’s good. A person can then talk. If he doesn’t come. There was a time I was there for an hour. I didn’t understand from them that the appointment was canceled. Then they brought me an interpreter. He explained the situation to me and said it was cancelled.

Riham: So you were sitting there waiting for an hour?

Participant: Yeah. I didn’t know whether to wait or go. Another time, they requested an interpreter for us over the phone, and he translated for us over the phone.

Riham: When they tell you that it’s been delayed, do they tell you the reason? Like you were late?

Participant: No, no, no. Just like that. Prior to that appt, a day or two previously, we were receiving some phone calls in English, but we didn’t understand a word. There was this one time, other than this time, we asked if they have an Arabic translator. They said no there wasn’t. He kept talking… but I didn’t understand what he was saying. That’s what happened to us. It was a dentist appointment for me. [Indistinct]. I went three times. They said there was an Arab doctor.

Riham: An Arab dentist?

Participant: Yeah

Riham: Okay.

Participant: They were taking too long to give me an appointment. Till now they didn’t give me one. I don’t know when to go or come. It’s been a month and a half.

Riham: Mm. Is that for the kids?

Participant: No, that was for me.

Riham: For you.

Participant: Yeah. There is something else. They performed some tests and they said you have high cholesterol. We will treat that and get an ultrasound of your kidneys and abdomen.

Riham: Why for your kidneys and abdomen?

Participant: My stomach has been hurting me constantly.

Riham: You feel pain in your stomach, ok.

Participant: The first month we came, he supposedly made me the appointment and till now he did not say when or anything.

Riham: You’ve been waiting for four months.

Participant: Yes. He didn't even mention it. Even one time, I was in the organization, Catholic Charities, and there was a translator and I asked him about it. He said, honestly, it seems like you have high cholesterol. I am going to make you an appointment to follow up. Till now, nothing happened. I have no appointment.

Riham: This type of thing can be frustrating.

Participant: Yeah of course. He still didn't schedule an appointment for me. Maybe now if he makes me an appointment, I won’t even be ready for it. One time they made an appointment for the kids and they talked to me on a day I had a job interview and told me to bring all the kids in that day. I told them, I have a job interview today. How can I bring them all in today? All five of them! I wasn’t available. I didn’t go.

Riham: So you missed the appointment.

Participant: No, I didn’t miss it. I was in the organization that morning and he asked if it was possible to bring them later today... I said I had an interview and can’t bring them in at 1pm. At 1pm I had an interview. That was for the kids’ vaccinations.

Riham: You mentioned earlier the transportation problem. You said that when you used to have an appointment at the hospital you used to walk there.

Participant: Yes, the hospital is a ten minute walk from our home.

Riham: So it’s nearby.

Participant: Yes it’s nearby. I used to take them walking back and forth. There is also a bus that used to pass by our house but we didn’t know how to ride it and where to tell it to drop us off.

Riham: It’s hard.

Participant: It’s hard. One day it rained on us. We walked out of the hospital one day and it poured on us. What are we going to do? The kids walked in the cold. What are we going to do? We basically took a shower in the street.

Riham: How sad. You were walking from the hospital.

Participant: Yeah the whole way.

Riham: So they didn’t help you learn how to use the bus?

Participant: No, they helped us once for ten minutes. They have courses at Catholic Charities. On different topics. The case worker came once with me and rode on the bus.

Riham: So he came with you once.

Participant: Yeah he rode with me on the bus and told me where to get off.

Riham: Yeah.

Participant: And it was just my luck that there happened to be four or five different busses along that same route. I didn't know the difference between them! Every day I used to ride a different bus and get off at the wrong stop.

Riham: That’s too bad.

Participant: I would get off and have to go back and ride the bus back to the original stop. I would wait and wait. Sometimes I would be late. I wouldn’t arrive until 930 am. And then they would mark me off for being tardy, at the organization. They didn’t tell me there were four other busses on that same route.

Riham: Mm. So they didn’t explain it to you right.

Participant: Yeah, for 15 days I would struggle that way. If I happened to get on the right bus thats great. If not I would only find out till it takes a wrong turn. Then I get off and wait for the right bus.

Riham: So they didn’t tell you which color bus for example. The blue one or the –.

Participant: They were all the same. They had numbers on them. He didn’t tell me which number. If he told me to memorize the right number. I would have memorized by bus number.

Riham: You would have memorized it.

Participant: After 13 or 15 days that this would happen to me, there was a woman named Mona from the organization, not sure what the name of it was.

Riham: Was it called Rahma? Or Catholic Charities?

Participant: Yeah. I told her what would happen with me. She looked it up for me and gave me the times and the number of the bus. After that time it didn’t happen to me again.

Riham: Okay good so it doesn't happen anymore and you learned how.

Participant: Yeah, I spent two months going back and forth on the bus.

Riham: So I am hearing you tell me that you are smart, and you are able to learn quickly but they are not explaining things to you clearly.

Participant: Exactly. It’s not an issue of not knowing the bus. They all looked the same. It just happened that there were four or five other buses on the same route and I didn’t know the name of the right bus. That was the problem.

Riham: So they also didn’t teach you how to use the bus that goes to the hospital?

Participant: No, they didn’t teach me to go to the hospital.

Riham: Okay. Then you received a car?

Participant: Yeah, I then got the car from the people from the mosque. May God increase their good.

Riham: Thank God.

Participant: Yeah may God bless them. Thank God we are managing. We go and come with it.

Riham: How did you learn how to get around?

Participant: Honestly, we had a group that used to take us around. We memorized the way. Every way I went, after once or twice, I learned instantly.

Riham: Good job. I have been here for a year and still get lost. I use my GPS.

Participant: No, I don't use the GPS.

Riham: Some people are good with directions and some aren't.

Participant: Yeah, I am telling you this area, around the downtown Cincinnati, I don’t need a GPS. Around 15-20 kilometers around here, I don’t need the GPS. Every place I went I memorized it. I went on I-75 and learned how it works. I know it now. Just by going out with people. Sometimes my friend would come to take me to the mosque and he wouldn't know the way. I would tell him go like this and like that.

Riham: Wow. Great job. Were you a driver in your country?

Participant: Yes, of course.

Riham: Oh okay. So you’re really good at driving.

Participant: Yeah, thank God.

Riham: That was your thing then. You used to do it?

Participant: Yeah.

Riham: Okay good. Thank God. I am going to also ask you –. You told me you also faced some issues with the pharmacy. Tell me a little bit about that.

Participant: Yeah. When they would give us a prescription, back in my country, we could take it to any pharmacy.

Riham: Yeah, okay.

Participant: Here they assign you to just one pharmacy. You can’t go to any pharmacy unless it’s the one they are sending you to. That’s a problem here for us. The Kroger pharmacy in [indistinct] is far from our home.

Riham: It’s far now from your home.

Participant: Yeah, of course it’s far. The other day my wife and my son went to the hospital. They gave them a paper. This mix up happened. The next appointment when we went I went with him and from the hospital we went and got the medicine. When I am not with them, that’s the problem.

Riham: So you told me that you went to a few pharmacies and –.

Participant: Yeah, the Kroger here is close to us. I went and they wouldn't give it to me. I didn’t know.

Riham: Even with the paper in your hand?

Participant: Yes, the paper was in my hand. They wrote on it and –. The doctors even told me before, “If you go to Kroger and they don't give it to you, let them call us.”

Riham: Yeah, yeah.

Participant: We didn’t know how to tell Kroger to call the doctor. I pointed at the number and told her to call. She wrote a mark and didn’t understand what I was saying. She kept saying go to a different Kroger. The other pharmacy did the same thing. They were for two different dates that were back to back. One on the 12^th^ and one on the 16^th^. We went to the doctor and explained to her the problem. She was a good doctor. The doctors here are all good. We can’t really say otherwise. Truly, the doctors here are good.

Riham: The problem, however, is –. What I am hearing you say is that the problem is with the pharmacy, and the transportation prior to getting a car, and the problem of the system. It’s as if you’re trying to tell me its an issue of organization and communication as well. Like when you told me your kids were given an appointment at 9 am and your wife was also at 9 am and your other boys had one at 11: 15 am. How is one supposed to work with that? He can’t keep going and coming. However, you are not able to explain this to them.

Participant: Yeah I am not able to tell them this. One time I went without an interpreter. I waited an hour and half there and they didn’t tell me anything. At the end, my son was with me. I told him to go see the issue and he only knew a few words.

Riham: The 14 year old?

Participant: Yeah. The man started to get upset. Especially –.

Riham: Who, your son?

Participant: No, no the one that records the names.

Riham: The one that sits at the desk?

Participant: Yeah. He got upset at our questions. I was sitting there for an hour and a half and no one told me when to come and when to go. I couldn’t understand if the doctor needed to leave or not. Finally, it turned out it was a canceled appointment. I was sitting waiting for an hour and a half there. They want to enforce rules on me and not on themselves?

Riham: Oh, now I understand what you’re saying. You were ten minutes late once so they canceled your appointment but when you waited an hour and half that one time they didn’t let you in.

Participant: Yeah, they told me they were overbooked and what not. Okay, why would it matter to me how busy they are? Right or wrong? They disregard how busy I am. When I was 10 minutes late, they enforced their rules on me… but when I would wait an hour and a half with my kids, who would get uneasy? I can’t keep them sitting there. A kid can’t sit for a minute. Imagine an hour and a half. That’s what bothers me. Every time we go we have to wait an hour or so before our turn comes. If it even come! Three times we missed our turn and once they took us all in. So out of five times that we went, we were only let in two of those times.

Riham: Yeah.

Participant: Last time regarding my daughter’s appointment. We were late ten minutes and they canceled it but I would be kept waiting an hour and half. That’s the system that I am telling you about. This is the order.

Riham: Yeah, that can be really frustrating. Especially when a person comes from a stressful place. You guys came from Syria?

Participant: From Iraq.

Riham: So you went from Syria to Iraq then from Iraq, to here?

Participant: Yeah.

Riham: So you’re going to a foreign land. For the second time. So these types of things can really depress a person.

Participant: Yeah, of course. Sometimes we say –. In front the kids we can’t say… but between you and I, if we would go to Somalia that might be better for us.

Riham: Why?

Participant: Wherever it may be it would be better than here! Here they put you under a lot of psychological stress. The house here is expensive. Our rent is $950. One time I got an electricity bill for $400.

Riham: Oh no! $400?!

Participant: Yes, $400 dollars. What was I to do?

Riham: What are you guys doing? $400 for electricity?

Participant: Yeah.

Participant's Wife: For the month.

Participant: For the month.

Riham: Is it from the air conditioner?

Participant's Wife: We don’t even put it on. This whole month we didn’t turn it on. We’ve been really hot.

Riham: How sad!

Participant: I am telling you. It’s a thing with this country. It is expensive. In Syria, we did not pay this amount for the whole year!

Riham: Yeah, of course!

Participant's Wife: Even in the refugee camps we did not deal with these problems.

Riham: Yeah, Oh my God.

Participant: Yeah, he was telling me something is not right. I was telling my friend and he looked at it and said no it was accurate. It’s not a matter of being accurate or not.

Riham: No it’s too much. $400 is expensive.

Participant: I am a refugee, and I am coming to this land and everything is making it more difficult on us. I am telling you that some of the system [rules] is enforced on us and some isn't.

Riham: Yeah.

Participant: That’s what I am talking about. I am a refugee. We would never have to pay the electricity bill before. At all.

Riham: But you were living in a camp, isn’t that right?

Participant: Yeah, that’s right. Everything was provided. I wish they would send us back there and we don’t want anything else from them. We don’t want America or the luxuries of America. What luxuries are these? Anywhere I would go if I paid a $1000 I would live the best luxurious life.

Riham: What do you mean?

Participant: Like look at this house right here. If I paid 500 anywhere else I would get the best house possible. I can earn more elsewhere too.

Riham: Yeah, of course. It’s an expensive life here.

Participant: If we knew that we wouldn’t have come here. Why would we put our kids through that? In this jail here?

Riham: Yeah.

Participant: It’s not like it became an easy life. No, just the opposite. There is more stress on us.

Riham: Because it’s an expensive country and it’s foreign to you and the language barrier and all those things.

Participant: Yeah, I am telling you. It’s really hard.

Riham: Okay so what are you doing to help your psychology, your mental stress? As a father, you have a different type of stress. That is why we interview mothers and fathers separately. You have different stressors. What do you do to help yourself?

Participant: Honestly, I am telling myself to be patient. It’ll pass. First year or two might be hard but slowly I tell myself it will get better. Feeling foreign. I am trying to make it easy on myself. To tell myself to look ahead. I don’t know what will happen tomorrow or after a month. I just, often, how can I tell you in Arabic? The problem is my Arabic is also weak.

Riham: Because you speak Kurdish. That’s better than me. You don’t know how to express yourself in it.

Participant: Yeah. I don’t know how to express myself.

Riham: Say it in Kurdish and then think about it. Then translate it to Arabic for me.

Participant: The problem is we are forgetting our language here. We can’t say it in Kurdish, English, or Arabic. We are losing our language….Ok. How shall tell you. [Speaks in Kurdish]

Participant's Wife: Like when one makes the road easier by putting hope in front of them… and they keep going.

Riham: Like you put a goal in front of you and you give yourself hope in reaching it. Telling yourself you’re going to reach it one day.

Participant: Yes, I tell myself, especially for my kids. In these four walls, this square. I don’t want them to feel it. The stress. Sometimes we have obstacles and problems…and if we don’t let it go, it begins to spill onto other things. And then it stays in our mind, and we spend a week or two stuck on the issue. (points towards his wife)

Riham: Are you referring to your wife? It is not just your wife. All women, we worry about things.

Participant: After an hour or two, we need to put issues aside. Take it out of our minds. Otherwise, we then take it out on the kids…become angry with them…with the wall…with a rock, anything. It’s better to put the issue on the side and keep going forward.

Riham: Yeah. Now everyone in their life finds and learns new ways to calm themselves down. So you are telling me that you, kind of, give yourself pep talks in a way. You tell yourself there is hope and I have goals and my goal may not be accomplished today but eventually one day it will. Is there anything else that you do? You go for a jog? Do you know what I mean?

Participant: By the grace of Allah, Allah gave me patience. I have patience. When I get angry. It lasts from a minute to about four minutes. Not more than that. Also, if someone wrongs me, I forget it in a minute or so. I smile soon after.

Riham: That means your wife is really lucky.

Participant: Of course she’s really lucky. God created me like this and He created her like that.

Riham: That’s why they say married couples are opposites.

Participant: Like she might sit 4 or 5 hours upset. Not say a single word. You can touch her and lift her up and she won’t say a single word! A small smile will take away my anger. It’s from God. Her father, may God have mercy on his soul was like that. She took after him.

Riham: May God have mercy on his soul.

Participant: He didn’t used to talk a lot. If someone cries, he wouldn't say a word. Some people are like that.

Riham: Yeah.

Participant: She’s one of that type. It’s a problem here.. but we have gotten used to it.

Riham: Thank God. Yeah after five kids you managed to deal with it.

Participant: Thank God.

Riham: Is there anything else that you can do like talk to your family overseas? Or by talking to your friends here?

Participant: Yeah.

Riham: Tell me?

Participant: When I talk to my family abroad, I calm down a lot.

Riham: You don’t feel more upset out of worry for them?

Participant: No, they also feel better if someone talks to them.

Riham: Thank God.

Participant: Thank God. Sometimes I feel homesick. Just yesterday, I talked to my aunt and her husband. They talked to me.

Riham: May God keep them safe for you.

Participant: Thank You. I told them the years I lived in another city in Syria…the five years I lived in Iraq, I did not feel homesick the way I feel here in these past 3 months.

Riham: Yeah.

Participant: 16 years are not like the 4 months here.

Riham: Yeah.

Participant: So there is a difference.

Riham: There is a huge difference.

Participant: This is what God wrote for me.

Riham: Are you able to meet people like you, Syrian refugees? Or even other Arabs?

Participant: Yeah of course. We have Syrian families living here. The mosque also introduced us to some. We go and visit one another. We go out together. We go to the mosque to see one another on Fridays and Sundays. Thank God.

Riham: So you feel that helps you with your homesickness?

Participant: Of course! If it weren’t for the community, for these people, we would be in a much worse state.

Riham: Of course.

Participant: It would have been more difficult. Thank God there are new Syrian families. There is Abu Bara and the people of the mosque. May God reward them. If it weren't for them, our situation would have been much, much more difficult. Sometimes I call Abu Bara to translate for me. If it weren't for that it would have been a problem honestly. He helps me. Sometimes when I went to the hospital, I did not think of Abu Bara or the people from the mosque. They slipped my mind.

Riham: In your opinion, is there anything that could have helped you more when you first arrived? Or even now? Because I am hearing you say there are some problems like the appointments, the pharmacy, and being able to understand the doctors over the phone. Is there anything that could have helped you with these problems? Things that could have increased your ease or relieved your stress?

Participant: Yeah of course. I didn't really understand your question.

Riham: Okay my question is what do you think could have helped you physically and emotionally adjust to life here. Not only to adjust but also to feel at ease at the doctors office and in terms of the appointments. Do understand what I am saying?

Participant: Honestly, let me tell you. No matter where one goes, to be able to settle in a new home, in a new country, it would take a year or two to full settle in. We are refugees coming to a foreign land. Millions have come. If we want to assimilate to this environment in a month or two, it won’t happen! I am telling you it won’t happen. So before we came we knew, it would take a year or two or three before we settle. One needs patience. Patience is everything. If you have patience it will work, if you don’t it will not.

Riham: Of course.

Participant: Sometimes we lose patience. The reason could be a child or a wife or something. These problems happen wherever one goes. We didn't experience the big problems thank God. The people from the mosque, if it weren't for them honestly, it would be a different situation! If it weren’t for the people from the mosque, we could be having a different conversation. They first came to the airport and brought our case worker who did not know a word of Kurdish or Arabic. They brought Abu Abdallah with them too. I forgot all my troubles! All the exhaustion I felt. All the stress, I forgot it.

Riham: They brought Abu Abdallah and Um Abdallah with them too?

Participant: Abu Abdallah and Amer. The hardship I felt from the two days of travel were all forgotten.

Riham: Because you felt at ease.

Participant: Yes, I felt at ease. When they brought us home, they also brought our luggage. Even when we got home we found the food ready, everything was ready. They asked us what we needed. A person feels at ease in this case.

Riham: Of course.

Participant: If it weren't for that, I would not know what would’ve happened to us.

Riham: Thank God.

Participant: Thank God. It’s all by the Grace of God.

Riham: May Allah increase you in all that is good. I don’t want to make you late because we have a minute left.

Participant: What time is it?

Riham: A minute till 2pm.

Participant: Okay.

Riham: Is there anything else you have on your mind that you’d like to share?

Participant: No, there isn’t.

Riham: Okay thank you for sharing your thoughts.

Participant: You’re welcome.

Interview 9B

Riham: Today is August 16, 2017. I am Riham with a mother who has been here in Cincinnati for four months. She came from a refugee camp. She lived first in Damascus then she went to a camp in Iraq then she arrived here. Okay. Welcome. Thanks again. Let us start with a point that we were on. We were talking to your husband, and you and I were talking about this topic. Being in a foreign land away from home is hard. And at times, people experience incidents that are challenging emotionally and physically. Sometimes people see things they never imagined they would see. What do you do to maintain a healthy psychology yourself? And how do you maintain your children’s wellbeing? First tell me about yourself then about your children.

Participant: When we first arrived I often felt like I did not have any control over myself. I felt like life ended for me. I would have these episodes. Especially when we first arrived. Even the kids used to sit and cry and ask “why did we come here and why did this happen to us.” I used to sit with my kids all day and we would cry. I would serve food, and they wouldn't eat. I wouldn't eat.

Riham: Did you lose weight?

Participant: Yeah, honestly, I lost weight. We lost a lot of weight. It was really hard on me and on my kids. When we first arrived our home was an apartment. They told us in this apartment our children should not be making any noise. My kids weren’t allowed to make any noise or be loud at all. I was so worried. I used to be very strict on them, all five of them.

Riham: How sad. It’s hard to constrain them all the time. You have five kids.

Participant: I have five and I would see them in front of me constrained. There were times were I didn’t know how to deal with it at all.

Riham: What would you during those times that you were upset? Would you scream and cry?

Participant: I would sit and cry and scream until I felt a little better. Thank God when we came to this house, the situation improved.

Riham: How long have you been in this house?

Participant: It’s been about a month.

Riham: Oh so it’s new! May God bless your home.

Participant: Thank you.

Riham: It’s really nice, honestly. I thought you’ve been here all along.

Participant: No, when we were in the first home… we were really upset with it. We were very upset with the first home.

Riham: Why were you so upset with it?

Participant: We had neighbors that lived below us. They were a family of four. At times they would scream late in the night around 1 or 2am. The police would even come. My kids would get afraid. We are not used to things like that. The kids would come and be afraid. We were also afraid. We would wake up at night and wonder “What would happen to us?” We’re not used to people like that. We are not used to life like this. After 3 months, the Catholic Charities caseworker said we were on our own. They said they had nothing more to do with us after 3 months. Then, there was a Syrian family here may God reward them.

Riham: Are they from the Rahma organization?

Participant: No. They are also refugees. One of them has been here for a year, and the other one for six months. This one over there is the house of Abu Khaled.

Participant's Child: Abu Muath.

Participant: Abu Muath. They saw this house for us. They said this landlord that we’re renting from, three other families rented from him. They told us this house was vacant and if we were interested they would tell the landlord to rent it to us. They would be close to us.

Riham: Oh they live close to here. I had wondered why I recognized the area.

Participant: Yeah. They saw this house for us and we saw it and we moved here. After that I can’t say that we were completely comfortable but it is much better than the first one.

Riham: So you felt that the way the house looked and the location of the house affected you psychologically?

Participant: Yeah. Now here I have a backyard. My kids can play outside and no one will bother them. Here we have neighbors on this side and on this side. Americans. Thank God they have not bothered us at all.

Riham: The previous neighbors bothered you?

Participant: The other ones as I was telling you at times would wake us up at 3am and the police would come. They would be screaming and shouting –.

Riham: They would get drunk and high?

Participant: I don’t know. We were too afraid to even look, to find out what was happening. This house, however, thank God no one bothers us.

Riham: Thank God. Did you feel like your kids were more at ease here as well?

Participant: Yes, my kids are also more at ease here. Thank God. I feel like they are much more at ease here than at that other first house. Emotionally they are better.

Riham: Thank God. What are some other things in your opinion that you feel have helped your children’s psychology?

Participant: In terms of my children's state of mind, I have my older son as an example. Sometimes I would be talking to him, and he would be screaming and crying and banging things. He would ask, “Why did you bring me here?” There he had his friends and he was used to it and no one was interfering with us. I didn’t know how to deal with that. Sometimes things would happen with my kids… that I didn’t know how to deal with honestly.

Riham: What were you able to do during those situations? You weren’t able to comprehend what was happening, but what did you do in response?

Participant: I would discourse with them. Tell them we were all in the same boat, all here together as a family. Hopefully, it will get better. When we arrived to the camps we were in the same situation. Although, thank God, we came over all together.

Riham: Thank God. Can you maybe encourage him to go and play sports or meet other refugees his age?

Participant: No, there weren't any other refugees. When the Rahma organization sent a woman over named Asma, it helped me so much. Even with my oldest son. She took my oldest son and introduced him to her kids and thank God. Then he had friends. This woman, may God reward her, has helped me so much. Her kids were playing sports, a ball team, and she helped my son join the team.

Riham: That’s really good. When one joins a team it helps them assimilate more.

Participant: Yeah honestly this woman has helped me so much. Her and Jackie. Till now, anytime I need anything she helps me. They did not come up short with anything that I needed. May God bless them.

Riham: May God reward her.

Participant: Amen.

Riham: Okay and what about your daughters. These things helped your son so what about your daughters?

Participant: As for my daughters, she also has a daughter the same age as my daughter. This woman, she came and took them to the mosque. She would take them to her house, and sometimes she would bring her daughter here. She found them friends from the same religion and same language. After that… I noticed my kids were doing much better psychologically.

Riham: As your husband said, being a refugee in a foreign land is hard but if one meets others like him with similarities or common values they feel at ease a little. Us as Arabs and Muslims, we do interfere in each other’s affairs, but we also at the same time…

Participant: Yes, this is our culture

Riham: It is what we are we are used to, and we still support one another. So what I have been hearing from other refugee families is that the Arab community here has really helped them adjust. Do you agree?

Participant: Yes, I agree. We are always there for one another as a community. As Arabs. We do not come up short.

Riham: We support each other. So if these other Arabs weren't here, what would have happened?

Participant: Without them, we would have all been lost. Especially when we first arrived. God sent them to us. We would have been lost. When we first saw them, I felt like I saw the whole world. In their faces, I saw the entire world. When they brought us home and brought us to the mosque, we felt completely at ease. Thank God. They helped us. We feel the same about all the other Syrian refugee families. Without them, we would have lost everything.

Riham: You guys are lucky because when you came, there were others here before you. The ones that came at first probably had a different experience.

Participant: Yeah. Thank God. I think we were the last family. Things have changed…

Riham: The environment kind of changed around here. A new president and –.

Participant: It did. May God make it easy. Life is all a test. These are all tests. God will make it easy. Us Syrians are really strong. We are strong.

Riham: That’s true. The Arabs are strong and the Syrians and the Muslims. May God increase us in strength. Okay please tell me a little about your experience with the doctors and the healthcare system here.

Participant: When we went to the doctors here –. My whole coming here was due to medical reasons.

Riham: Why?

Participant: With my last pregnancy I had kidney failure. I had been on dialysis.

Riham: You’re young though. How old are you?

Participant: I am thirty.

Riham: You’re thirty years old and you have kidney failure?

Participant: Yeah, it happened to me from my caesarian delivery.

Riham: Do you still do dialysis?

Participant: No they stopped the dialysis. I was in Syria, and they stopped the dialysis. I went to Iraq and they were treating me there.

Riham: Okay so from before you had this.

Participant: This problem happened to me during the events in Syria. I delivered my daughter during that time and I had a Cesarean section. There was excessive bleeding during the surgery and from that my kidneys failed.

Riham: Okay I understand. You probably went into hemorrhagic shock and had kidney failure subsequnetly. Okay.

Participant: And then we left Syria… to go to Iraq. We left because of this medical problem. The people migrated and so did we. How did it happen? I was in Syria taking this medicine. Then the medicine ran out. The doctors said there wasn't any left in the country.

Riham: Because you were in a camp?

Participant: No, this was before I went to the refugee camp. It was in Syria. I kept taking this medicine. As long as I live, I need to take this medicine. It ran out in the whole country. We searched the whole country. So we left Syria with the other migrants. We spent a year, yes a year, suffering in the camps when we arrived. We were living in tents. For three months we were sleeping on the grounds and in schools. They were bringing lots of food for everyone but we really suffered a lot. We were tattered. We suffered. The children did too. Then, they fixed up the camp, created an actual refugee camp… and made it livable. Till now I still say that I wish they would take me back to the refugee camps and not have to live the life I live here.

Riham: Why do you say that?

Participant: Life in the camps was better. My husband, thank God, had a car and everyday he would work by driving. He brought home his earnings and we lived on that day to day but I was comfortable. I was at ease psychologically. I didn’t have all this stress. Here we have so much stress.

Riham: Tell me about your stressors here.

Participant: Like for example the rent for this house. My husband is working and he still can’t pay it. The electricity bill that came that was $400. It’s hard. He works every day but he cannot afford it. Now they are telling me I need to go to learn the language, and I need to work. I have kids and I am sick. I can’t go work. I take medicine. I am followed by doctors. They know all the details of my life and they still pressure me. This is all pressure. Pressure on my mind. They are giving us a card to purchase food with it. This amount is not enough.

Riham: The food stamps?

Participant: The food stamps does not last us 20 days. I have kids. A person has to buy things and not let them feel like anything is missing. Sometimes we won’t even have enough to buy bread. That’s our situation now.

Riham: It wasn’t like that in the camps?

Participant: No, it wasn’t like that. Because in the camps my husband would work and everything was cheap and affordable. We didn’t have to pay rent, electricity or water bills. They would give me about $150 dollars a month to buy things. This amount would last me the whole month and more honestly.

Riham: Here it doesn’t even last you a week.

Participant: Here yeah the food stamps doesn’t even last me 20 days as I told you. Now they are giving us a $100 for every person in the house. Now my husband is going to start working so they’re going to cut my $100 and my husbands $100. It’s not even a lot of money… but they’re going to cut us off because my husband is going to start working. As a punishment?

Riham: But he isn’t even making enough.

Participant: He isn’t making enough and on top of that they are going to cut the food stamp amount in half. These pressures and stressors placed on a person are too much. When I was in the camps I didn’t have any of these stressors.

Riham: Yeah.

Participant: While living in the camp we weren't like this. I didn’t have to worry about financial issues every month. Here when Eid (Muslim holiday) came, my daughter said, “Every Eid you used to buy me new clothes. You don’t want to buy me any this Eid?”… That was really hard. For anyone. It’s hard to see my kids during Eid. We used to celebrate, even while in the camps, I could afford new clothes for them. Here I am not able to save any money.

Riham: You’re not able to save anything.

Participant: Yes I am not able to save anything. Here we are experiencing things like that. I am watching my kids go through these things, and I tell myself “Why did I put myself in this situation? Sick or not sick. Let me die there in the camps and not see my kids in this state.”

Riham: May God give you health.

Participant: I came here due to my illness. To get treatment for myself and in the process I made it difficult on my kids. I have lost my kids. I lost myself, the kids, my husband…and the whole family. The whole family is suffering because of one person. It’s really hard on me.

Riham: It seems like you are taking all the responsibility for this situation. It’s hard for one to feel responsible for five kids and their husband and all that happened.

Participant: It’s really really hard on me. When I first left Syria, I sought psychological help. I was seeing a therapist in the refugee camp. I was getting treated.

Riham: Did you go see a psychologist here?

Participant: Here, I have been requesting for four months to see a therapist. I keep trying. Even in the camp I was seeking therapy. Even in Syria, I had sought help. When I had left Syria, because of the incidents and the experience, my mental health had suffered. So I was being treated in the refugee camp. I was really suffering mentally.

Riham: Were you taking medicine?

Participant: I was taking medicine. Before I arrived here I stopped taking them. When I first arrived here I told them immediately, “I need to see a psychiatrist. I really need one.” Till now they did no provide one for me.

Riham: It’s been four months and you still didn’t see a psychiatrist?

Participant: Four months and I still haven't seen a psychiatrist.

Riham: And you told them you really need one?

Participant: I told them and I told them and I told them. There was even a time…I attempted suicide, but my husband stopped me.

Riham: What did you do?

Participant: That previous house we lived in, we were on the 2^nd^ floor. I had a balcony. I ..

Riham: You were going to jump?

Participant: I wanted to jump from the balcony and commit suicide. I was feeling like I couldn’t take it anymore. I told them I urgently needed one. Till now they didn’t bring me a psychiatrist.

Riham: When this incident happened to you, and your husband saw you, did he take you to the hospital?

Participant: No. My husband tried to help me by talking me out of it. He said don’t take it too hard on yourself and whatever God wrote will happen. No matter what we do, it will happen exactly how God intended. He said all we need is patience. Thank God that I have a very patient husband. I am so lucky He is really helping me. God sent me a patient husband. This incident I described to you and with my children, thank God, my husband is really helping me.

Riham: Okay when this incident happened to you. Did you talk to the case worker and tell them this happened to you?

Participant: Yes.

Riham: Okay what did they say?

Participant: They said they want to send me to see a psychiatrist.

Riham: Okay, where is this psychiatrist?

Participant: They took me once to this organization that does the language lessons. She just asked me a few questions, and she said we will make you an appointment with the psychiatrist.

Riham: How long ago did this happen?

Participant: It’s been a month and a half, and they still didn’t make me an appointment.

Riham: Do these suicidal thoughts still come to you?

Participant: Not since I came here, I really felt more at ease after coming here.

Riham: You mean “here”… to this house.

Participant: Yes, in this house. I felt more ease. Like I told you before, we have two Syrian families living nearby. They come visit often and we go to them. That really puts me at ease.

Riham: What I just heard from you though is really wrong. If a person is asking for help and saying they attempted suicide and still not receiving help it’s really wrong. It’s legally wrong.

Participant: I told them that I had an incident where I attempted suicide. I described the whole incident to them, and till now they didn’t get me to a psychiatrist.

Riham: God willing when we end this conversation, I would like to get you a number of someone you can talk to. Please give me the name of the psychiatrist they told you about. Because often times, what happens is that they might have made an appointment for you but it was not clear to you. It could have been a misunderstanding. Do you understand what I am saying? It’s been a month and half. You could have attempted suicide again during that time.

Participant: No they told me that they made me an appointment with this psychiatrist. Let me find it for you. Let me look. [long pause] Here see maybe this is it. Maybe my husband took it with him.

Riham: No this one is for the phone company. Okay remind me after to see it.

Participant: It was similar to this, the paper looked like this one… but they told me that it might take time before I get to see the psychiatrist.

Riham: Show me this paper if you find it because it shouldn’t take this long. If they didn’t even start you on medicine or treatment, the incident might happen again. I am concerned. Do you know what I am saying? If you ever feel these thoughts returning you need to call 9-1-1. Or let your husband take you to the Emergency Room. They would give you an appointment there. Sometimes they would start you on some medication immediately. Or at times they might admit you to the hospital for a day or two so they can help optimize your mental health. Did anything like that happen to you?

Participant: When I first arrived, my state wasn’t good at all. Especially because of my kidneys. I was hospitalized for 7 days.

Riham: Due to the kidneys?

Participant: Yes, due to the kidneys. I didn’t see a doctor for my kidneys. I had infections, in my urinary system and kidneys. Till now I didn’t see a kidney doctor (nephrologist). They just told me we want to treat the infections you were having. Apparently they treated it and after that I left the hospital. Then this incident happened to me about a week ago…. This episode returned. I would scream and cry till I passed out.

Riham: This happened a week ago?

Participant: Yes, a week ago. Then my husband called the ambulance and they took me to the ER. Then we went there and they didn’t tell us anything. My neighbor came there, to the hospital, and stayed with me. I felt more at ease. In the ER, I did not see a doctor nor did they tell me why this happened to me! Nothing!

Riham: No one talked to you?

Participant: No one talked to me. After getting off the ambulance they took some blood work, and we were sitting in a room. They told us to leave the room. We went out, me and my neighbor…and we sat. We waited for an hour or two. It turned late. It was nighttime, and no one was home with the kids. They then told us the blood work is going to take time. So my husband said in that case I am going to go home. He told us to call him if anything comes up.

Riham: That means you waited a long time in the emergency room that you couldn’t see a doctor.

Participant: It was about 2 hours. I was waiting in the room.

Riham: The emergency waiting room or the patient room?

Participant: No the patient room. I stayed there for 2 hours and didn’t see a doctor. They didn’t even tell me what happened with me and why it happened with me.

Riham: No one talked to you? Not even an interpreter?

Participant: No they didn’t talk to me. No interpreter. Then we went outside, and they told us the results need two hours or so. My kids are waiting at home so I told them I need to go home. I told them to call me when the results come out. I asked them to use an interpreter and call me with the results. I can come back after the results if you call me. Until now I don’t know the results or anything. I don’t know why this all happened to me. I don’t know anything. So many things happen to us here… and may God help a person. What is a person to do?

Riham: Yeah. What do you think would have helped you in that situation? First an interpreter of course. No one talked to you in Arabic, this I understood. What else?

Participant: No they brought an interpreter. They told us to come to the room till the doctor comes. The doctor never came. After that the interpreter didn’t say anything else to us. That is all the interpreter said.

Riham: So the interpreter didn’t interpret anything?

Participant: Well they didn’t bring a doctor for him to interpret anything. The doctor didn’t come and ask me what happened with me for example. Usually the doctor asks you a series of questions, but the doctor never came. They only sent an interpreter to tell me to go wait in the room till the doctor arrives. We went to the room and waited for the doctor. It took two hours so we left to go home.

Riham: Because it took too long.

Participant: Yes, because it took too long.

Riham: I had told you before; I work in the Emergency Room and sometimes the wait can be long. Some people wait an hour or two before they see a doctor. So you both left before you could see a doctor.

Participant: No, they took us out of the room. They told us to wait outside. We were in a room and they told us to go out and wait in the waiting room.

Riham: That’s strange.

Participant: And I didn’t see a doctor. That night, there was a big accident maybe. She said there were people there that have been shot, and they had a full room that night in the hospital. There was a lot of pressure in the ER. That was the reason that I wasn’t able to see the doctor…I understood this because the woman with me, she spoke some English and was able to understand the situation. I just never saw the doctor.

Riham: Because if you had found out why you passed out and why this happened to you, you could have perhaps received help then and there.

Participant: Yeah

Riham: Do you have a primary care practitioner?

Participant: They assigned one for my family, but I have not gone to see her.

Riham: You’ve never gone to see her?

Participant: No. They just gave me her name. When we used to go, they told me that we have one chosen a family physician for your family.

Riham: Where is she?

Participant: I don’t know. Till now, I don’t know the number and we weren't told where to go and so on.

Riham: Do you go to Millvalle? Its name is Millvalle?

Participant: No. It is a problem.

Riham: Where they administer the vaccines. Next to it. That’s what I am hearing from others, their doctor is there?

Participant: No, we didn’t go. It’s possible that they told us our doctor is there, but they didn’t give us her number or tell us that she’s our doctor.

Riham: Okay. That’s a problem .

Participant: It’s a huge problem.

Riham: Is there anything that could have helped your health and your children’s health. Of course, I can tell what they are but I would like to hear it from you.

Participant: Till now, they did not help me with anything. For me personally, they did not help me with anything.

Riham: Okay so what are the things you need that you are not getting?

Participant: I need a psychiatrist. Till now I have not seen one. I need a doctor for my kidneys. Till now I have not seen one. As I told you of my state. The reason I even came to America is for medical reasons, for my kidneys, and till now I have not seen any doctor.

Riham: The doctor appointments were are not made?

Participant: No, I didn’t have any appointments. This appointment that my husband was telling you about earlier is the one for my kidneys. The cancelled one. Every time we go to the nephrologist, they tell us the appointment has been canceled.

Riham: How are they canceling it every time?

Participant: I don’t know. Our case worker, even though, he is no longer working with us, he called my husband and told him I had an appointment in the hospital. We went there and we were sitting waiting for like an hour or two. After that they told us your appointment is canceled. We asked for the reason and they said we don’t know. We just know that it was canceled. No reason.

Riham: It’s clear that there is a misunderstanding.

Participant: We don’t know what the reason is. They aren't even providing a translator to explain to us the reason.

Riham: They aren't even calling you by phone?

Participant: No, only the case worker called us.

Riham: How are you able to understand the case worker?

Participant: Our case worker has an interpreter. He explains it to my husband.

Riham: Where do you record your appointments?

Participant: I record them on a book, so we don’t forget.

Riham: So you aren't missing the appointments?

Participant: No. My daughter arrived 10 minutes late to her appointment, and they cancelled the appointment. My husband said “Because you canceled this appointment due to 10 minutes I will not even come to the next one.”

Riham: So he got upset.

Participant: Yes, he got upset. So then they canceled the other appointment as well.

Riham: This appointment was for what?

Participant: The vaccines. Since coming here my kids only received one set of vaccines.

Riham: How many appointments do they need? Do you know?

Participant: No, they didn’t tell us how many vacccines they need. Now school has started and from what we know, they need all their vaccines before they can go to school…. Even in terms of their school, we don’t know which one they will be attending! The schools are open. Not even where the bus stop is. School started and my kids have not gone yet.

Riham: You don’t have a paper that explains it?

Participant: No, they didn’t give us anything.

Riham: How did you know that your 14 year old son has school today and which school it was?

Participant: Because when we first arrived here my son attended school for 20 days.

Riham: The other kids didn’t go?

Participant: No, because by the time we finished their paperwork, the school year ended.

Riham: Is there anyone you can talk to you so that you can register your kids in school.

Participant: I am going to first check with the case worker from Catholic Charities. If he is able to help me, then that’s good. If not, then apparently he does not have to.

Riham: Have you spoken to Jackie, the caseworker at Rahma?

Participant: No. Not Jackie. I am going to talk to Jackie and tell her what happened with me.

Riham: Jackie is a social worker. You’ll also feel more at ease once they start school.

Participant: First I will try again with my caseworker. If he does not help me, then I will reach out to Jackie. Last year they missed school and if they also miss this year it’s an issue. My youngest are really excited about going to school. They want to go. I am going to try to fix this problem for them.

Riham: God willing. Having too much free time is difficult. Especially for children.

Participant: Of course. What is one to do?

Riham: May God make it easy.

Participant: For everyone.

Riham: Amen. Are there any programs that in your opinion can enhance your children's well being?

Participant: The school will help a lot. If we had anything like that it would help so much. Remember that woman I told you about that used to come and take my kids out? When they would come back I would feel that they were in a much better mental state. I could see it on their faces.

Riham: Thank God. How about you?

Participant: Even for me. When she would take us out on Sundays, we would come back and feel much better. We would pray. We would see people.

Riham: You go every Sunday?

Participant: Yes.

Riham: They have a lesson every Sunday?

Participant: They have an English lesson every Sunday at the mosque.

Riham: Do you find that helpful?

Participant: It doesn't really help me… because it’s only once a week. When you come home you forget everything.

Riham: You don’t review what you learned?

Participant: When I am not feeling well… mentally and emotionally I am not in the mood to learn. I don’t have it in me to plan and decide to do this or that. The thing that is on our mind now is just to get a passport so that I can return. I don’t want to stay here, in this country. I won’t stay here. I have decided. My husband doesn’t share the same opinion. He says, since the incidents in Syria, the last five years, we have gotten lost. We don’t want to lose our kids too. He wants a future for our kids. We want to sacrifice what we can for our children. In the camps… they were going to school but I know it wasn’t that good for them. The level of schooling in the refugee camps, I know, was not good.

Riham: What are your hopes for the future? For your kids’ future?

Participant: My hope is that my kids –. I sit and tell my kids that if we ever go back they will be the generation that rebuilds that society. If we can go back to our country, these kids. They will do something great. I told them they need to go to school and become something. Become doctors or engineers or things like that. That is everything we need. We are here so… that you don’t get lost. So you can have a future. There is a Syrian family here that I see them at times, and I argue with the mother. I tell her to stop saying that to her kids.

Riham: What does she say?

Participant: She is destroying her kids. She tells her kids, “We came here and we don’t want to learn or go to school. We just want to return.” I also say I want to return, but I don’t say that in front of my kids. I don’t tell them to not go to school and not care..because I want to go back. I don’t do that to my kids. That woman is destroying her kids. I do not want to destroy my kids. When I see this woman, I feel like she is discouraging her kids. If I said these things, my daughter would say to herself, “Okay then, why would I put in any effort. If my own mother is telling me not to care.” Thank God, we don’t do this to our kids. We give them hope to make something of themselves. Thank God, till now our kids are –.

Riham: I asked you before if you studied. You did not get the opportunity before to go to school. So this is something new for your girls.

Participant: Yeah, I didn’t study. Until now It doesn’t matter how old a person is though. Times have changed. Things have happened….If a person doesn’t learn, get an education, it’s as if they are blind. I don’t want that to happen to my kids. I would like them to study and to become something. I want my kids to have a future, and a promising future. They are still young and have a future ahead of them. This country, however, really puts stress on a person.

Riham: It does.

Participant: If I was more at ease, mentally and physically, if I saw that there was enough help, in this country. If I saw that this house’s rent was affordable, if the government paid half and I paid half for my rent…and that we are getting enough assistance from the government, then I too would be motivated to learn. I would want to learn and be educated. Me. To grow myself as person. I have never been to school.

Riham: You’re still young.

Participant: Yeah. If I go back and study I too would have a future.

Riham: Of course.

Participant: However, a person’s mental health is not at ease. There is too much pressure and stress being placed on a person here. It doesn’t allow me to even think of something like that.

Riham: Yeah.

Participant: Even when it comes to the lessons on Sunday. I am really grateful to all those teachers that come out and volunteer to teach us. It’s not their fault. However, sometimes, we are not in a state to learn. I see…I swear… women sitting and crying in the lessons. Honestly. At times I see them sitting and crying. A person cannot help it. We are just flesh and bones, just nothing…and we have seen so much. A person imagines everything that has happened to them, questions why it happens to them. It is compulsory almost. Nothing is going to change the past. It happens a lot…. When I see a woman in front of me reading and crying. I also start crying. I start to remember things. I start to wonder why this and why that. I cannot focus on the lesson, on anything. Especially in this country. I have a sister in Germany. Thank God, I am talking to her and she is really happy and her kids are happy. They are making her life so easy in Germany. Why? Because they are making life really easy on the whole family.

Riham: Tell me how. I don’t know what they do there. I only know what they do here in America. Is it better in Germany and why?

Participant: Let me tell you why. They are giving a good amount of money to the kids. The government pays the entire house’s rent.

Riham: They don’t have to pay rent?

Participant: No, not at all. They don’t have to pay house rent. The groceries and clothing, there are cheap.

Riham: But things in Germany are expensive.

Participant: No, honestly, at times I tell my sister of some of the prices of things I pay here and she tells me that it’s not like that for her. It’s cheaper. For example, the government helps them more. There if a person doesn’t know the language they don't have to work. Here, whether you know it or not, you still have to go to work. It’s not like that there. My sister has been there for three years now. Both her and her husband do not have to work.

Riham: What do they do?

Participant: They are sitting at home and the government is paying for everything.

Riham: Did they give you a choice of which country to go to?

Participant: No they chose for us. I had no choice. If they told me from the first interview that it was going to be America, it would have been better. After the third interview they tell you where your name is assigned to. If they told me from the very first interview that I was being sent to America, I would have told them I don’t want to go. From the beginning.

Riham: Yeah because if you were able to choose to be next to your sister, that would help a lot. It is easier being near your relatives.

Participant: Yeah, if they told me which country do you have family –. My sister is there and my brother-in-law (husband’s brother) as well. We have lots of people there.

Riham: Oh, you have two families there.

Participant: Yeah. My sister is there, my brother-in-law and my cousin is also there. They are all there. If they told me to choose, we would have chosen Germany.

Riham: So they did not give you a chance to choose?

Participant: No. If they told us we would have chosen for ourselves. They don’t do that though. They choose for you. That’s what they do to people. They just tell us which country chose us.

Riham: Mm.

Participant: They didn’t tell us all the details of life here. They left many details out. They only told us that life here is difficult and told us a lot of things but they did not explain what would happen with the house and the rent and whatnot. If I knew all this, I would not have left the camps. Like I told you before, if I had a chance to go back to the camps, I would prefer refugee camps over life here. We really struggled here.

Riham: You’re not able to though.

Participant: No, we are not able to. Even the plane ticket in which we used to come here with is on us to pay back. It will take six years to pay off the tickets! How is one supposed to do that. They really suffocate a person and make it difficult on them. All the debt and expenses.

Riham: It’s a country of debt.

Participant: It’s a country of debt. Thank God, that as an environment it’s pretty. The natural beauty. Everything in it is pretty. There are a lot of things that are nice here. If only they don’t make life here difficult on us, it would be so much better. If they make it difficult on a person – [long pause] Foreign is foreign. I got married into a foreign land. In Damascus it was even foreign to me. Then I went to Iraq. Then I came here. I can handle what is foreign, differences. I look back and realize that I was able to put up with a lot. I handled a lot. This type of experience here, I have never experienced this in my life. These four months here have been unlike anything I have ever experienced before. The stress and burden it puts on a person. It’s too much. It’s not like I am one person. I am attached to my family. If one was alone, it’s different. When it’s a family that is tied down to you, it’s different. May God make it easy. I know He will make it easier. God wrote for this to happen. Thank God. If a person is a believer and is a Muslim we need to believe in everything. Thank God.

Riham: God willing it will be easier. There is a saying in English that hopefully this is the worst of your days, and they will only get better. Same thing like you mentioned about the refugee camps. It was very difficult at first, and then it got tremendously better. So you don’t know what is to come and what lies ahead.

Participant: Yeah, honestly. No one knows what is written for them in this world. What is one to do? Just this morning I was saying …all of this, it is out of my hands. What am I supposed to do? If I was in my country and was with my family it wouldn't have been this hard. It’s just that I am in a foreign land. I don’t know anything. It’s as if I was deaf. I don’t know how to move about in this land. I do not know how to move about. I do not know how to do anything. What am I supposed to do? Thank God we pray and we call out to God, and we are trying to be patient. We say that hopefully God will make it easy. There is nothing more we can do than that. Thank God. This is not something small. Faith.

Riham: It’s not. There are so many people that lost their faith.

Participant: Yes, honestly. Till this day I tell my kids not to focus on this land but to hang on to their faith. Thank God.

Riham: Thank God. May God make us all from those that hang on to their faith.

Participant: God willing.

Riham: Let me just see what other questions I have here that we didn’t discuss. You told me that you have been seeing doctors, nursed and hospitals. In your opinion, what is your expectation of the best experience?

Participant: How should I imagine?

Riham: Okay I am going to give you an example. Imagine yourself in a clinic or hospital, and what would you imagine to be the best experience? What elements would you want?

Participant: I am not sure how to imagine…

Riham: Let’s consider the same question about expectations. If I was asking about a restaurant experience. I would answer by saying, for example, that I walk in to the restaurant and smell something amazing. The waiter takes me to a clean table. He gives me the menu and speaks to me in a friendly, kind manner. The food doesn’t take too long to arrive. The food turns out to be delicious. Do you get what I mean? Use this same example, only I want you to apply it to the medical system.

Participant: The practice of medicine here… we don’t feel like they made it difficult on us. Yes, we had issues with the appointments. The appointments are challenging. But the actual doctors didn’t make it difficult on us. They were really friendly and gentle with us. The doctors are gentle. The nurses were really friendly. I was in the hospital for seven days and the nurses were really kind.

Riham: So in terms of the actual people you are not finding any problems. As in the doctor and nurses.

Participant: No, no I am not having any issues with them. They don’t make you feel like you are a foreigner. They still respect you and everything. I have no complaints about them.

Riham: So it’s the system that is difficult.

Participant: Yes, the system is difficult that they have here. They make it really hard on a person. In terms of their interaction with you, it’s really good. We didn't see otherwise. Truthfully.

Riham: Thank God. When you went to the emergency room –. You’ve been there twice, is that correct?

Participant: Yes, I have been there twice.

Riham: Okay what are some things that bothered you and what are some things that you liked about your experience?

Participant: When I went to the Emergency Room the first thing they do is register a person and everything. After that, the thing that is not good, is that when they take you to a room and you have to wait for the doctor. You wait and wait. They take way too long. This causes a person to leave and not wait for the doctor.

Riham: So the time it takes to wait for the doctor. That’s the thing that bothers you.

Participant: Yes, the time it takes to wait bothers me. It is too long. Sometimes a person goes at 7am and they stay till 4 or 5pm in the hospital. Sometimes it’s not to even see a doctor. It might be for something simple like getting a test done and just waiting for the results. Even if the doctor was to see a person, they shouldn’t be waiting for him that long. It’s hard for a person to sit in a room waiting the whole time. Even mentally it is hard to wait so long. A person starts to say, “Why should I wait. I don’t want to see the doctor anymore.”

Riham: Okay so if they explained to you the process for example, will that make a difference to you? Like if they were to say that after the doctor orders some tests it might take the nurses half an hour to an hour to follow through on the order, be it a lab test or a medication. The nurse needs to draw blood till they send it to the lab, that might take 10 minutes and so on. Then in the lab, there are many samples that need to be run, and it may take X amount of time before YOUR blood is sent. And then the result is placed in the computer. Then the doctor needs to read it and interpret it. If they were to explain the process to you would it help?

Participant: Yes, if they explained all that to me it would help a lot! I am sitting waiting and I don’t know why I am waiting. After all that wait then they might come and say “Okay you can go home.” A person won’t even know why they waited all that time only to be told to go home. When I lived in [indistinct] camp I used to go to the doctor. They would explain everything to me. The results of the test in full detail. I have been here for four months and still do not know the results of my blood work. I used to monitor my creatinine and calcium levels. Here? I don’t know at all what I have, how I am doing. But when I was in Iraq, every week I would go. Sometimes I would stay in the hospital for seven days. That was when my health was in a really bad state.

Riham: Due to your kidneys?

Participant: Due to my kidneys. I also had a surgery at that time to remove the uterus. After the kidney failure I was told they needed to take out the uterus as well. Then I did that surgery in Iraq. They used to explain everything to a person. They would tell me exactly what medication I needed and why. Here? they give me a medicine and I don’t know what it’s for!

Riham: You are currently taking medication?

Participant: Yeah they are giving me medication now.

Riham: But you don’t know what it’s for.

Participant: I don’t know what each medication is for. When I was in Iraq and had that surgery to remove the uterus, they made me stop all medication. They told me because those medicines –. They had tied my bladder to my uterus. They sewed my bladder to my uterus. So when my monthly cycle would come, I had to stay in the hospital the whole time till it ended. Because it used to come out of my bladder.

Riham: Wait let me understand. They sewed them together then during the surgery.

Participant: Yeah, they sewed them together. They made a mistake.

Riham: That’s why you would get urinary tract infections.

Participant: Yes, that is why I would get infections.

Riham: How sad. That’s terrible.

Participant: For three years I was in this condition and they didn’t know that it was happening to me. They were just giving me medicine. For seven days I was taking medicine. They could not figure out the problem. I would get medicine.

Riham: Antibiotics then.

Participant: Yes, antibiotics and I would have to stay in the hospital till my menstrual cycle ended.

Riham: Why?

Participant: Because my period was coming out as urine and not where it’s supposed to come out from. I used to get a high fever and not be able to stand myself. Two nurses and two doctors weren’t able to hold me down. Because it was a unbearable fever. After three years, they decided to check using an ultrasound to find out what the problem was. Then they understood what happened. They were giving me medicine for my kidneys and it was affecting my kidneys...worsening my kidneys.

Riham: I see. How sad. May God give you good health.

Participant: Thank you. I stayed there till they took me in for surgery. When they brought me in the anesthesiologist came and said he is not going to drug me. The surgery doctor asked him why. He said because this woman has this issue with her kidneys. If I go forward with this surgery and drug here, this woman will not come out of the surgery alive. My creatinine level was 6 at the time. She will not handle the anesthesia, the doctor said. Bring another anesthesiologist because I will not do it, he said. The surgeon then said, that he was not able to do the surgery because the anesthesiologist doesn’t want to drug me. I stayed in that condition for a whole year. They refused to do the surgery. One time I went to visit my mom and she said that her neighbor was telling her about a doctor.

Riham: Your mother was in Iraq?

Participant: Yeah, my whole family is in Iraq now.

Riham: Oh, that’s why you felt at ease there.

Participant: Yeah all my family is there in Iraq.

Riham: They were in the camps as well?

Participant: Half of them were in the camps and half of them were living in the city. Then my father came and took me to see this doctor. He said he wanted to do the surgery then and there. He said he will take full responsibility. I told him I won’t do it today, I’ll do it tomorrow. The next day when I went the doctor gave me anesthesia and I was in surgery for four hours. They did regional anesthesia.

Riham: Oh no. So you were awake.

Participant: Yeah, I was awake while they were doing the surgery. After that surgery my state was much better. They kept treating me with medication for my kidneys. Right now both my kidneys only partially work. Before coming here by three months, the doctor told me to stop taking all my medication. He told me that the medication was affecting my kidneys. I stopped the medicine. When I came here, they started me back on it again. Every time I go they would give me more medicine. They don’t even explain to me what the medication is for, what it helps with. Till now, I don't even understand anything from the doctors.

Riham: That means you are currently in need of a kidney doctor and a gynecologist so that you can understand what surgery you had and what remains inside of you. Do you know if you have the ovaries, for example?

Participant: Yeah, they said they took one and out and left one in.

Riham: They took one out and left one in, okay. But you still have to follow up with a gynecologist.

Participant: The doctor who did the surgery said that I had to see a gynecologist every 2 moths.

Riham: Yeah, you have to see a gynecologist, a kidney doctor, a primary doctor, and also a psychiatrist. Those are the four specialities, that you have yet to see.

Participant: Yes, I didn’t see any of those doctors. I went and saw a urologist twice, though.

Riham: Okay, good. A urologist.

Participant: But those other doctors you mentioned, no I didn’t go to see them yet.

Riham: How about the primary doctor. Did you see her or not?

Participant: Which one?

Riham: A family primary care doctor.

Participant: No I didn’t. They told me I had one, a doctor assigned to my family, but I didn’t see him yet.

Riham: Yeah, you need to see him or her.

Participant: Yeah I didn't see them.

Riham: May God give you a lot of good. You have a difficult story. If a person is hurting physically, it also affects them mentally and vice versa. The two are intertwined.

Participant: Yeah, that’s what I am telling you. On top of all that, they are telling my husband that if your wife doesn’t come in to attend the lessons or doesn't attend, we’re going to cut all your benefits. It’s as if they are really giving us sufficient benefits. On top of that they want to cut them! Till now it’s all because of my issue with the doctors. Once I finish with my medical issues then I’ll go every day. If they don’t give us our benefits, my whole family will be affected. What is one going to do? They are pressuring us. So much.

Riham: I am going to stop the recorder now. Thank you.

Part 2

Riham: Okay, I apologize. Please continue.

Participant: I want to tell you something. Something happened with us with Jerusalem market. Sometimes a person has no choice but to go there. On the day before Eid, we went there. We needed some meat so my husband told the butcher what we needed. The butcher then started to shout at my husband.

Riham: What did he tell him?

Participant: He said, “You see me washing up and I am ready to close and you still order some meat”. My husband then replied that he didn’t say anything wrong to him. He didn’t swear or speak rudely, he just simply asked for some meat. Then the man continued to shout at my husband. We had a lot of things in our cart that we needed for Eid and my husband said lets leave. “We don’t want any of these things,” he said. My husband was crying. Tears were streaming down his face. He said, “I am telling you lets go.” We left…. There was also another time that the same incident happened at the Halal market. Same exact situation happened over the meat. There were some people standing, waiting. My husband knew that he had to wait his turn, but he wanted to first ask the butcher if the type of meat we wanted was available. Before he waited in line. He started shouting at my husband. He said get out from here, I don’t want to give you any meat. If you don’t want to wait your turn, I don’t want to give you meat. These two incidents, these things that happened to us brought tears to our eyes. I was crying on our way home. Never in my life were we ever treated like that anywhere in Syria and Iraq. This coming from Arabs like us that are supposedly our own people yet they treated us that way? Lots of similar incidences happened here with us…. We’re in a foreign land and we don’t really know how to come and go or from where to buy things. Everyone here is in a similar situation. They don’t know where to buy their groceries from. What is affordable and what isn’t. Where to go to find this or find that. What is one to do? We are forced to buy these things so we can survive. [long pause]

Riham: Is there anything else you wanted to add?

Participant: No.

Riham: Okay. Thank you very much.
